# Selective sweeps versus introgression - population genetic dynamics of the murine leukemia virus receptor *Xpr1* in wild populations of the house mouse (*Mus musculus*)

**DOI:** 10.1186/s12862-015-0528-5

**Published:** 2015-11-10

**Authors:** Natascha Hasenkamp, Terry Solomon, Diethard Tautz

**Affiliations:** Max-Planck Institute for Evolutionary Biology, 24306 Plön, Germany; Biomedical Sciences Graduate Program, School of Medicine, University of California San Diego, La Jolla, CA USA

**Keywords:** MLV, Retrovirus, Receptor, House mouse, Population, Introgression

## Abstract

**Background:**

The interaction between viruses and their receptors in the host can be expected to lead to an evolutionary arms race resulting in cycles of rapid adaptations. We focus here on the receptor gene *Xpr1* (xenotropic and polytropic retrovirus receptor 1) for murine leukemia viruses (MLVs). In a previous screen for selective sweeps in mouse populations we discovered that a population from Germany was almost monomorphic for *Xpr1* haplotypes, while a population from France was polymorphic.

**Results:**

Here we analyze *Xpr1* sequences and haplotypes from a broad sample of wild mouse populations of two subspecies, *M. m. domesticus* and *M. m. musculus*, to trace the origins of this distinctive polymorphism pattern. We show that the high polymorphism in the population in France is caused by a relatively recent invasion of a haplotype from a population in Iran, rather than a selective sweep in Germany. The invading haplotype codes for a novel receptor variant, which has itself undergone a recent selective sweep in the Iranian population.

**Conclusions:**

Our data support a scenario in which *Xpr1* is frequently subject to positive selection, possibly as a response to resistance development against recurrently emerging infectious viruses. During such an infection cycle, receptor variants that may convey viral resistance can be captured from another population and quickly introgress into populations actively dealing with the infectious virus.

**Electronic supplementary material:**

The online version of this article (doi:10.1186/s12862-015-0528-5) contains supplementary material, which is available to authorized users.

## Background

Host-pathogen interactions are an important driver of evolutionary processes and the characterization of their molecular basis is of prime interest in evolutionary biology [52, 56]. Murine leukemia viruses (MLVs) are extensively analyzed pathogens in mammals [31]. They were mostly studied in mice including analyses of their main receptor *Xpr1* (xenotropic and polytropic retrovirus receptor 1) [7, 29, 53, 61].

*Xpr1* is a highly conserved gene in metazoans and expressed in various cell types. The gene encodes a cell-surface receptor with eight annotated transmembrane domains which result in four extracellular loops (ECL) [53]. It belongs to the group of G protein-coupled receptors and has been shown to function in the export of inorganic phosphate [18]. XPR1 mediates infection of cells by both polytropic (P-) and xenotropic (X-) MLVs in a variety of hosts [9, 11, 30, 39]. XP-MLVs belong to the gamma-retroviruses and are distinguished according to their host tropism, interference pattern and pathogenicity [9–11, 20, 60]. Generally, MLVs can cause leukemia and lymphomas, but their pathogenicity is highly variable and dependent on virus strain and host background [31]. Sources of this variation are the generation of pathogenic P-MLVs (mink cell focus forming MLVs) by recombination events, genetic variability in the receptor binding domain of the viral Env glycoprotein and representation of pro-viral elements in the host genome ([6, 10, 11, 14, 20, 33]; [51]).

Co-evolution between XPR1 and XP-MLVs has been suggested in various studies based on lab strains and samples of wild-caught mice from scattered locations. Five MLV-restrictive alleles of *Xpr1* have been identified which, for example, lead to the resistance of *M. m. castaneus* against P-MLV infection or of some lab strains against X-MLV infection. Mutagenesis and functional analyses have identified XPR1 residues which are important for virus interaction [30, 38–40, 53, 57] and phylogenetic comparisons have suggested that the receptor has been under recurrent positive selection [60]. Accordingly, the currently available data suggests that there is functional variation among *Xpr1* alleles and that this could play an important role in the adaptation of mice to infections by XP-MLVs (reviewed in [31–33]). However, while there has been an extensive analysis of receptor and associated pro-virus variation in inbred strains and individual wild-caught mice of different subspecies [3, 4], information about receptor variation and its evolutionary turnover within a broad survey of wild populations is so far not available.

In this study, we use samples from wild caught mice derived from a number of populations. Samples were obtained in a way that ensures full allelic representation from a given area [24]. General population parameters and demographic models were previously assessed for these populations in various combinations [2, 24, 35, 36, 48, 54]. These studies had shown that the samples are suitable to identify selective sweeps and balancing selection through genomic signatures. In one of these studies [48] we found a major difference in haplotype diversity around *Xpr1* between local populations of *M. m. domesticus* from Western Germany and Southern France (Additional file 1: Figure S1). This suggested that a recent selective sweep had occurred in the population in Germany. Here we study the variability of *Xpr1* alleles and haplotypes in multiple populations from Western Europe, as well as a population from Iran and populations from the subspecies *M. m. musculus* from Eastern Europe. Surprisingly, we find that the low level of polymorphism seen in the German population is also typical for other populations and that it is the population from Southern France that has an unusually high polymorphism. Closer inspection and comparison of haplotypes across populations show that this is due to a recent introgression of haplotypes from Iran into the population in Southern France. We propose a scenario of frequent selective sweeps in *Xpr1*, possibly due to an ongoing co-evolution between receptor variants and bursts of infections, complemented by an introgression of receptor variants that convey resistance from other populations.

## Methods

### Mouse work

The animals used in this study are *Mus musculus*, a species that is not protected. Permits for catching them were not required at the time they were caught. Some specimens were caught on the properties of private landowners, with their oral permission to enter the property and catch mice. Mice were trapped in live traps, provided with food and shelter, by experienced personnel under the direction of DT. Trapping was only conducted at moderate temperature conditions, so that there was no danger for trapped animals to suffer from heat or cold. After trapping, mice were transferred into standard mouse cages containing food, water and shelter. Transportation to the laboratory, maintenance and handling were conducted in accordance with German animal welfare law (Tierschutzgesetz) and FELASA guidelines. Permits for keeping mice were obtained from the local veterinary office “Veterinäramt Kreis Plön” (permit number: 1401-144/PLÖ-004697).

The population samples used in this study were derived from previous trapping campaigns [24, 36, 48]. The study of viral particles in live animals involved dedicated crosses and collection of feces. It was assessed by the responsible animal welfare officer Prof. Schultheiß, University of Kiel, who is also the leader of the institutional animal welfare committee that discusses important topics of animal welfare regularly. Since the project did not involve any harm or stress to the animals, it was not considered an animal experiment that needs further approval of the governmental competent authority (MELUR) according to the German Animal Welfare Act.

### Mouse sampling

Samples from two subspecies and 11 distinct wild mouse populations were analyzed to assess patterns of allelic variation of *Xpr1* (see Table 1 for sampling locations). Samples were collected by Ihle et al. [24] and Linnenbrink et al. [36] following a sampling strategy designed to capture the variation in a local population and to avoid the inclusion of related animals [24]. One population in Southern France (Fra_MC_) was sampled twice with eight years between the samplings. All populations are represented in this study by 12 animals each, with the exception of Ger_CB_ that is represented by 11 animals.

**Table 1:** Population origin of wild-caught mice and population genetic parameters. *M. m. domesticus* populations from France (Fra), Germany (Ger) and Iran (Ira). MUS represents the two *M. m. musculus* populations

Population ID	Sampling location	Year	Variable sites/haplotypes	π per site (x 10^−3^)	Tajima’s D	Fu and Li’s F*
Fra_NA_	Nancy	2010	3 / 2	2.55	1.53	1.31
Fra_LO_	Louan-Villegruis	2010	3 / 2	3.00	2.25*	1.54
Fra_DB_	Divonne les Bains	2010	3 / 2	2.32	1.16	1.19
Fra_AN_	Angers	2009	3 / 2	2.89	2.08*	1.49
Fra_ES_	Espelette	2009	3 / 2	2.89	2.08*	1.49
Fra_MC1_	Severac le Château	2001	7 / 5	4.71 (2.60^a^)	0.86	0.27
Fra_MC2_	Severac le Château	2009	6 / 6	3.94	0.66	0.65
Ger_CB_	Cologne-Bonn	2010	3 / 2	0.54 (1.26^a^)	−1.73	−2.60*
Ger_SL_	Schömberg/Langenbrand	2010	3 / 2	1.72	0.21	0.88
Ira_AH_	Ahvaz	2003	3 / 2	0.49 (3.25^a^)	−1.73	−2.66*
MUS-CR	Czech Republic (Studenec)	2001	0 / 1	0 (1.42^a^)	n.a.	n.a.
MUS-Kaz	Kazakhstan (Almaty)	2001	2 / 3	0.48 (1.68^a^)	−1.20	−0.93

*significant at p < 0.05 level

^a^values in brackets refer to average π estimates for the respective populations from Baines and Harr [2]

Live animals for virus tests were taken from the wild-derived breeding stock at the MPI in Plön, which represent animals that had been originally caught according to the above mentioned sampling scheme and had then been kept in the stock over several generations, whereby the breeding followed a rotating outbreeding design (HAN rotation system – [44]) with 10 unrelated starting pairs. This design ensures a maximum degree of outbreeding [41] and maintenance of polymorphisms. Additional samples included ear punches of *Mus spretus*, *Mus spicilegus* and *Mus m. castaneus* individuals to complement the population sampling. To analyze variation in the receptor binding domain of X/P-MLVs and corresponding allelic differences in *Xpr1,* we collected ear punches and feces samples of Ger_CB_, Ira_AH_ and Fra_MC_ mice. The feces samples were stored at −80 °C for later RNA extraction and analysis of X/P-MLV variation. The ear punches were transferred to HOM buffer (80 mM EDTA, 100 mM Tris pH 8.0, 0.5 % SDS) and stored at 4 °C until DNA extraction and *Xpr1* allele determination.

### Mouse crosses

To test whether the identified virus variants occurred in the form of infectious particles or as transcribed pro-viruses, we conducted a cross-breeding experiment with animals from the Ger_CB_ and Ira_AH_ mice. The males were removed from cages as soon as the females were visibly pregnant. Before birth, the females were moved to fresh cages, and thus the pups were only in contact with the mothers. The feces samples were collected from the adult mice before and after mating, while the pups were sampled upon weaning.

### Extraction of nucleic acids

Extraction of DNA from tissue pieces was done by a standard salt extraction procedure. The tissue was digested with Proteinase K (1 μg/mL) in 550 μL of HOM buffer at 55 °C This was then mixed with an equal volume of 4.5 M NaCl and cooled on ice. 300 μL of chloroform was added and gently mixed. After centrifugation, the supernatant was precipitated with ethanol, washed with 70 % ethanol, dried and dissolved in TE buffer (10 mM Tris pH 8.0, 0.1 mM EDTA). Until further processing, DNA was stored at −20 °C and diluted to 5 ng/μL for PCR. RNA from feces samples was extracted using TRizol in combination with the PureLink RNA Mini Kit (Ambion®, Life Technologies, Carlsbad, California, US) following the protocol by the manufacturer. RNA samples were stored at −80 °C.

### RNA analysis

The RNA from the feces samples was reverse transcribed for later PCR and Sanger sequencing. The first strand cDNA synthesis was performed using the MMLV High Performance Reverse Transcriptase (Epicentre®, Illumina inc., Madison, Wisconsin, US). This was done using 350 ng of extracted RNA with oligo-dT-primers and following the manufacturer protocol. Afterwards, cDNA was stored at −20 °C.

### Microsatellite genotyping

We genotyped nine microsatellite loci within a region of 200 kb around *Xpr1* (Additional file 1: Figure S2). The forward primers were labeled with Hex at the 5’ end and four primer pairs were pooled per reaction. Care was taken that the pooled primer pairs yielded distinct product size ranges (Additional file 1: Table S1). PCRs were carried out using a multiplex PCR kit (Qiagen, Germantown, Maryland, US) in 5 μL final volumes and using 5 ng of DNA as template. Amplification conditions were as follows: 95 °C for 15 min followed by 28 cycles at 95 °C for 30 s, 60 °C for 90 s, 72 °C for 90 s with a final extension at 72 °C for 10 min. Afterwards, PCR products were diluted 1:20 in water and 1 μL was transferred to 10 μL Hidi formamide and 0.1 μL 500 Rox size standard (Applied Biosystems®, Life Technologies, Carlsbad, California, US). The subsequent denaturation step was performed with the following incubation times: 90 °C for 2 min and 20 °C for 5 min. Product sizes were automatically determined on a 3730 DNA Analyzer (Applied Biosystems®), and alleles were called using the GeneMapper v4.0 software (Applied Biosystems®). Alleles are listed in Additional file 1: Table S3.

### Sequencing

First, we identified variation in *Xpr1* alleles by sequencing parts of the coding sequence expected to be variable (Additional file 1: Figure S3), i.e. exon 4 and two putative extracellular loops (ECL3 and 4). For this purpose, five primer pairs were used (Additional file 1: Table S2) which amplify five fragments of the Xpr1 gene that are all within a 40 kb region (Additional file 1: Figure S2). Secondly, we analyzed the variation in the receptor binding domain of the surface unit of the viral envelope gene of P-MLVs (using the primers listed in Additional file, Table 2). PCR reactions for the amplification of exon 4, ECL3 and ECL4 from genomic DNA were carried out in 10 μL final volume with a multiplex PCR kit (Qiagen) and following cycling conditions: 95 °C for 15 min followed by 35 cycles of 95 °C for 30 s, 60 °C for 90 s, 72 °C for 30 s/90 s and 10 min at 70 °C for elongation time. Exo-Sap purification (USB®, Affymetrix, Santa Clara, California, US) was performed with the following incubation: 37 °C for 20 min and 80 °C for 20 min. Cycle sequencing reactions were done using the BigDye Terminator v3.1 Cycle Sequencing Kit (Applied Biosystems®). Reaction parameters were 96 °C for 1 min followed by 29 cycles of 96 °C for 10 s, 55 °C for 15 s and 60 °C for 4 min. The sequencing products were purified with the BigDye XTerminator Purification Kit (Applied Biosystems®). Sequences were generated on a 3730 DNA Analyzer (Applied Biosystems®). Independent base calling and analysis was done using CodonCodeAligner v4.0.2 (CodonCode Corp.).

**Table 2:** Numbers of microsatellite alleles for two loci (Xpr1_ms6 and Xpr1_ms8) sorted according to genotypes of *Xpr1* haplotypes for the Fra_MC_ and Ira_AH_ populations. The informative alleles are allele 265 for ms6 and allele 157 for ms8.

	Fra											IRA	
	I/I	I/Ia	I/IIa	I/II	I/III	I/IIIa	II/III	II/IIIa	III/IIIa	III/III	III/IV	III/III	III/IV
ms6													
263												1	
265			1		2	1	2	1	6	13	1	20	2
267													
269	2	2	1	1	1	1						1	
271										1	1		
273	1												
275							1						
277	1			1			1	1					
279													
281													
283				2	1								
ms8													
137	4	2		4		1	1						
153												3	
157			2		4	1	3	2	6	13	2	17	
161										1		2	

### Population genetic analysis

Because we only observed specific alleles together in the homozygous animals, we worked under the assumption that the five sequenced fragments from the *Xpr1* locus are linked. Haplotypes were phased manually and later reaffirmed using PHASE v2.1.1 [49, 50]. All haplotype phasing was consistent between the two methods, with the exception of sample Fra-MC_2501. Manually, we did not phase this individual. PHASE assigned this sample to haplotype IIIa and a novel haplotype, with only 50 % probability of these being the correct haplotypes. This sample was removed from any further analysis. All animals used for the haplotype analysis and their assigned haplotypes are listed in Additional file 2: Table S4.

Allele frequencies were calculated for all populations and their spatial distribution was visualized on a map made with NaturalEarth (http://www.naturalearthdata.com). Neutrality test calculations were done with DNAsp v5 [34]. The *Xpr1* region and *Vkorc1* region haplotype analysis on whole genome sequences was based on the vcf files from Pezer et al. [43].

The aligned sequences of the receptor binding domain in the surface unit of the viral envelope were inspected for SNPs and a phylogenetic consensus tree was calculated using MrBayes v3.2 [23, 46] using the unphased sequences.

### Availability of supporting data

The microsatellite genotypes, the reconstructed *Xpr1* haplotypes form the population survey and the viral RBD sequences are included in the supplementary files.

## Results

### Haplotype variation of *Xpr1* in wild mouse populations

To assess the allelic variation of *Xpr1* at the population level in the wild, we analyzed sequence polymorphisms of *Xpr1* from 11 house mouse populations (Table 1) and three related sub-species and species (*M. m. castaneus*, *M. spretus* and *M. spicilegus*). Based on previous data on the most variable parts of the gene, we focused the sequencing on the extracellular loops ECL3 and ECL4 of the protein (encoded in exons 10–13, Additional file 1: Figures S2 and S3) and included sequencing of exon 4 as an intracellular domain that is known to harbor variable sites ([26] - Additional file 1: Figure S3). All five sequenced fragments are located within a region of 40 kb and are treated as being derived from a single locus.

**Fig. 1:**
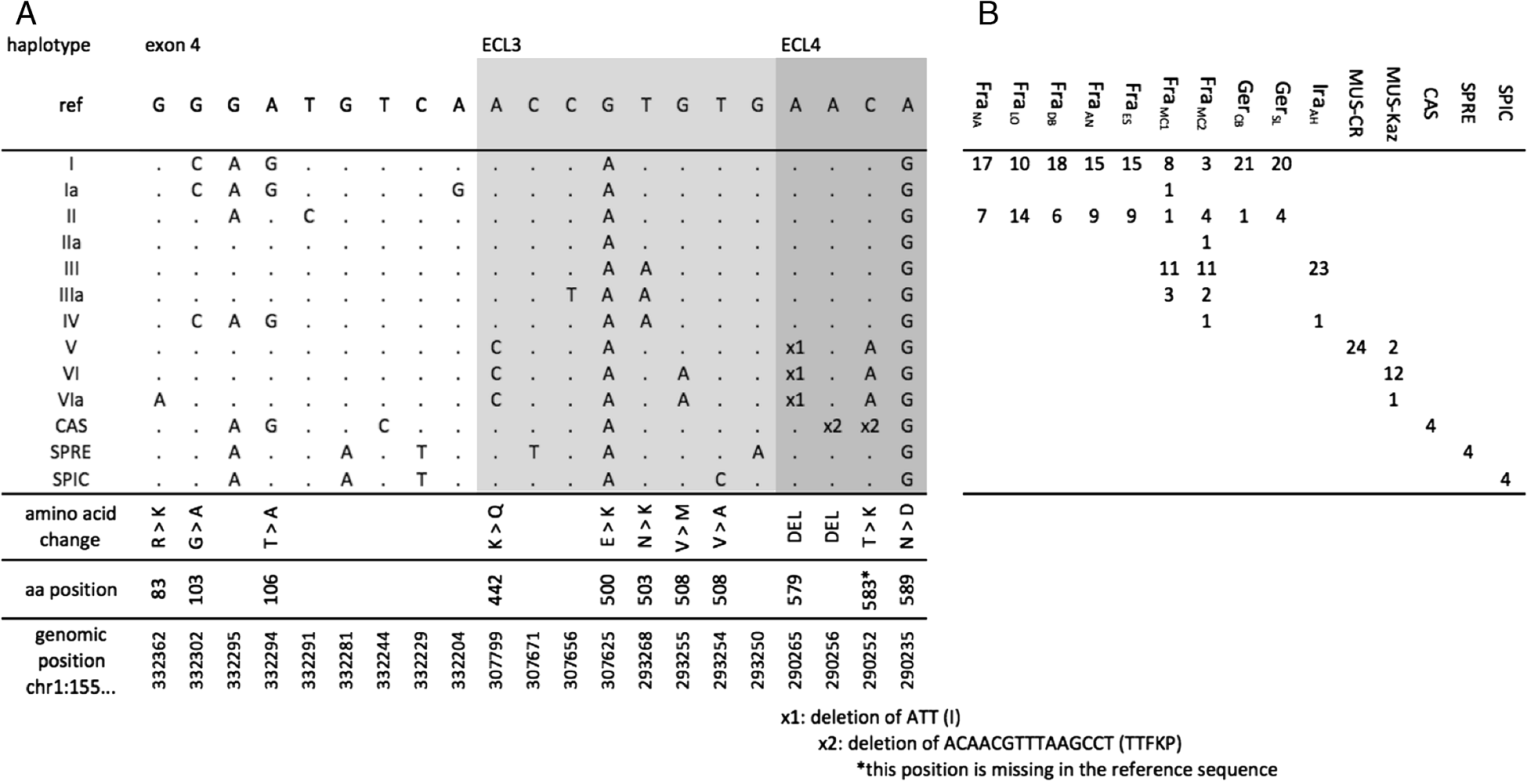
*Xpr1* haplotypes and their distribution in the populations. **a** Haplotypes - only variable sites are shown. The reference sequence (ref) is taken from the NCBI37/mm9 assembly; the genomic positions (bottom) refer to this assembly. Amino acid (aa) positions refer to the translated product. Nucleotides identical to the reference sequence are marked with a dot. The major haplotypes found in *M. m. domesticus* and *M. m. musculus* populations are designated by roman numerals. CAS, SPRE and SPIC represent the haplotypes of the sister species *M. castaneus*, *M. spretus* and *M. spicilegus*, respectively. **b** Distribution of haplotype numbers in the populations. Population designations correspond to those in Table 1. Note that Fra_MC1_ and Fra_MC2_ represent the same population sampled eight years apart. Suppl. Table S4 lists all haplotypes per individual

Between 11 and 12 individuals were included from each of the *M. m. domesticus* and *M. m. musculus* populations and two individuals each from *M. m. castaneus*, *M. spretus* and *M. spicilegus*. In these samples we found eight non-synonymous SNPs, nine synonymous SNPs and two indels. The two indels occur in *M. m. castaneus* and *M. m. musculus* and are located in the fourth extracellular loop (ECL4). Based on these polymorphisms, 13 different *Xpr1* haplotypes could be reconstructed (Fig. 1a).

**Fig. 2:**
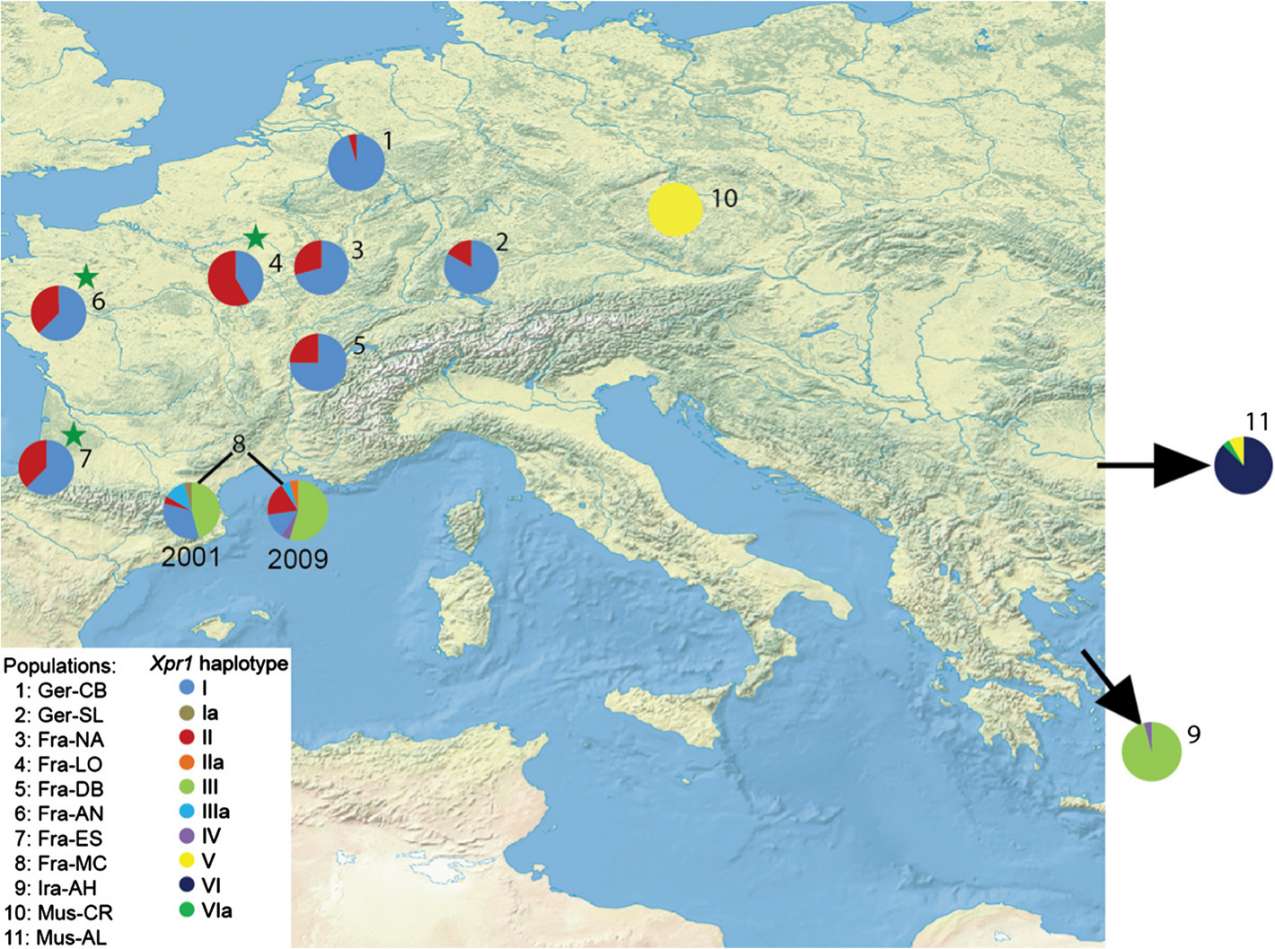
Map of population locations and haplotype frequencies. Haplotype assignments correspond to the designations in Fig. 1. Frequencies are depicted as pie charts. Green stars denote significantly elevated values of Tajima’s D in the respective populations. The background map was taken from the public domain map dataset NaturalEarth (http://www.naturalearthdata.com)

The identified *Xpr1* haplotypes varied in abundance and frequency in these populations (Fig. 1b). All Western European *M. m. domesticus* populations harbored haplotypes I and II but showed different frequencies of these two haplotypes, with the Ger_CB_ population being almost fixed for haplotype I (Fig. 2). Haplotypes I and II differ by two coding substitutions in exon 4, with each of them showing a single copy of a derived variant carrying a non-coding substitution (haplotypes Ia and IIa - Fig. 1a). The Iranian (Ira_AH_) population carries haplotypes that differ by a unique coding substitution in ECL3. This is mostly represented by haplotype III, but we found also a single copy of haplotype IV, which carries the ECL3 substitutions seen in haplotype III as well as the exon 4 substitutions of haplotype I (Fig. 2). The *M. m. musculus* population from the Czech Republic (MUS-CR) is fixed for haplotype V, which contains coding mutations in ECL3 and ECL4, as well as a deletion in ECL4. The ECL4 mutations are also found in haplotype VI, which is the most prevalent one in the *M. m. musculus* population in Kazhakstan (MUS-KAZ), but carries an additional mutation in ECL3. The three related species (*M. castaneus*, *M. spretus* and *M. spicilegus*) show separate fixed haplotypes but since they are represented by only two animals each, it may be possible that they harbor additional haplotypes.

This survey of wildtype populations shows that the predominant pattern is one where each population carries only a single or a most two XPR1 haplotypes with coding differences and a few rare additional ones with non-coding differences. Given this general background, the population Fra_MC_ from Southern France shows a stark contrast. We find a total of seven haplotypes in this population, three of which are present at elevated frequencies and confirmed in two separate sampling surveys (Fra_MC1_ and Fra_MC2_ - Figs. 1 and 2). Intriguingly, none of the coding haplotypes are unique, they constitute a combination of haplotypes I, II and III, with the latter one otherwise only found in Iran. In addition, we find unique low frequency derived haplotypes with non-coding substitutions, as well as one copy of haplotype IV, which occurs also as a low frequency variant in Iran. This suggests that the elevated diversity of *Xpr1* in the Fra_MC_ population could be due to introgression of haplotypes from Iran.

Table 1 lists overall nucleotide diversity π and results of neutrality tests for *Xpr1*. For π we compared the results with average values previously obtained for eight autosomal regions for some of the same populations [2]. Although our sequencing strategy for *Xpr1* was biased towards sequencing the most variable exons, we found that the nucleotide diversity is much below the average diversities found by Baines and Harr [2], with the exception of Fra_MC1_ (Table 1). This would be in agreement with the notion of repeated selective sweeps at the locus and a more complex scenario for Fra_MC_. Fu and Li’s F test is significantly reduced for Ger_CB_ and Ira_AH_, compatible with recent positive selection. Tajima’s D is significantly elevated for Fra_AN_, Fra_LO_ and Fra_ES_, which would suggest significant balancing selection between haplotypes I and II. However, the populations Fra_NA_, Fra_DB_ and Ger_SL_ harbor the same haplotypes but with an elevated frequency of haplotype I, which makes Tajima’s D non-significant for these populations, although they share the same haplotypes. Note that haplotypes I and II differ by multiple substitutions, i.e. are not directly derived from each other. Hence, the overall pattern is compatible with a scenario where these haplotypes arose in different populations and where the current populations represent an admixture of these two major haplotypes segregating across the European populations, with different admixture frequencies. Hence, the population parameter analyses support the previous inference of repeated positive selection on *Xpr1* [60], although other more complex demographic scenarios can not be ruled out.

## Recent introgression

The presence of the Iranian haplotypes in only one European population suggests a recent introgression. To further investigate this, we analyzed microsatellite variability and the presence of shared informative alleles around the locus. Microsatellites evolve so quickly that shared allele patterns would imply recent immigration rather than incomplete lineage sorting as a possible alternative explanation for the presence of an ancestral allele. We determined the alleles for nine microsatellite loci along the *Xpr1* gene (see Additional file 1: Figure S2 for locations) for all populations. Most allele spectra overlap and are therefore not informative for our question. However, at two loci (Xpr1_ms6 and Xpr1_ms8) the Iranian population shows a high frequency allele that is rare or absent in the other populations (Additional file 1: Table S3), apart from Fra_MC_. Further inspection of the Fra_MC_ animals showed that the animals homozygous for the Iranian *Xpr1* haplotype III are also mostly homozygous for the corresponding Iranian microsatellite allele with only a single additional allele in one locus each (Table 2). This observation indicates that the Iranian population (or a population that is closely related to it), would indeed have been the donor of this allele. Furthermore, this introgression must have occurred relatively recently. One can do a rough calculation of the age of introgression if one assumes that the two extra alleles found in animals homozygous for haplotype III constitute new mutations. In a previous study [54], we calculated that it takes about 1,200 generations for a new microsatellite allele to emerge at a frequency of 5 % in the Western European populations. Since the additional alleles occur at this frequency level (1 out of 14 = 7 % in the animals carrying the respective starting allele) and assuming about 3 generations per year, we can calculate that the introgression of the Iranian haplotype into Fra_MC_ would have occurred several hundred years ago. Hence, this rules out that the shared allele is due to incomplete lineage sorting, given that these populations separated several thousand years ago [12] (also see Discussion).

**Fig. 3:**
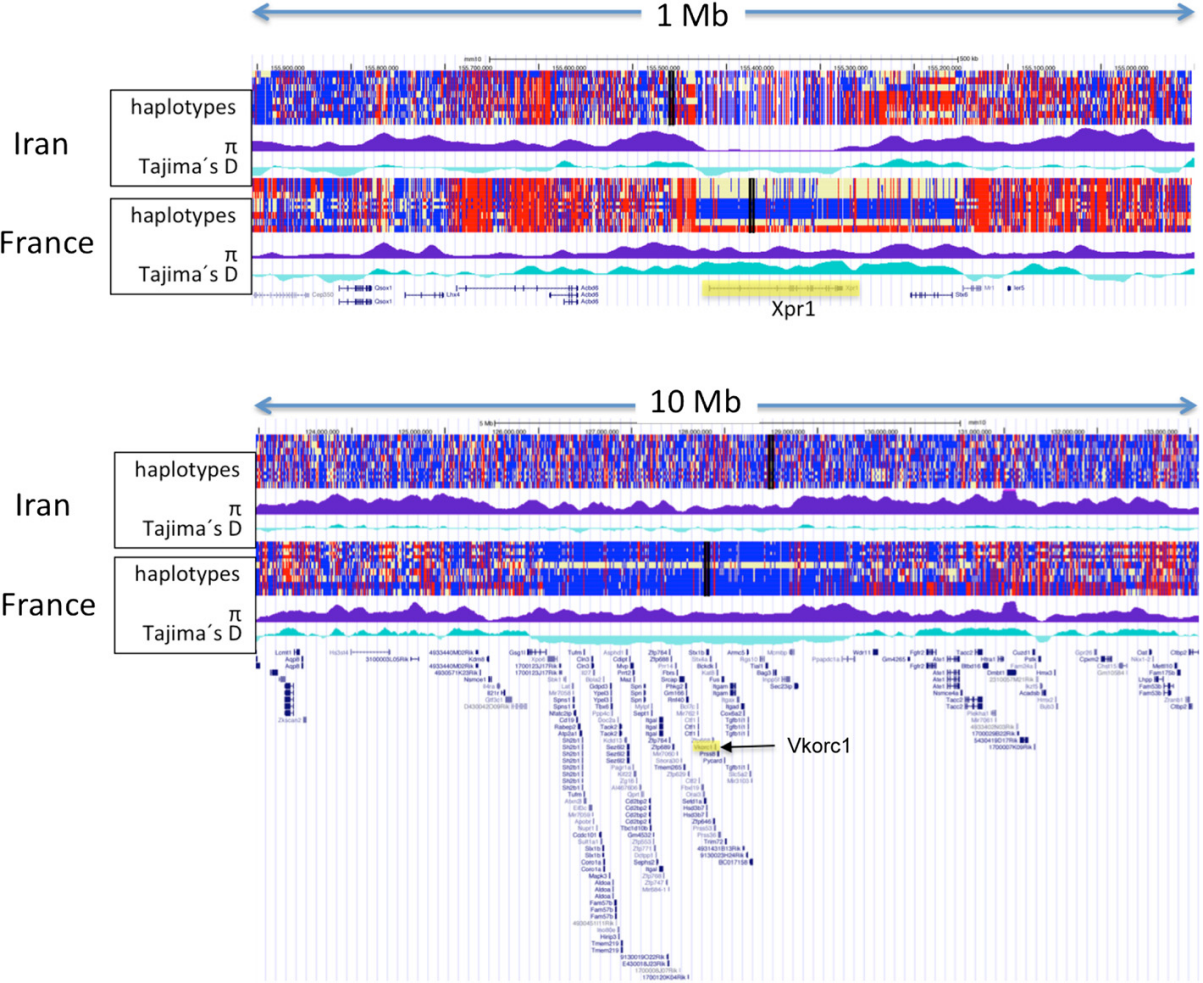
Views from the UCSC genome browser depicting SNP information derived from whole genome sequencing of samples from the Iranian (Ira_AH_) and French (Fra_MC_) populations. Comparison of the *Xpr1* introgression region (top - genome position chr1:154,900,000-155,900,000) with the previously described introgression of the *Vkorc1* region [47] (bottom - genome position chr7:123,000,000-133,000,000). Three tracks are shown for each population, the top one is based on SNP polymorphisms (vcf files) with the haplotype sorting function of the browser activated. The second represents the scores for nucleotide diversity and the third is Tajima’s D, both calculated across successive 1 kb windows. The scale is shown on the top, the RefSeq genes are at the bottom of each view. The focal genes are highlighted in yellow

To investigate this further, we have inspected whole genome re-sequencing data from a subset of the animals of the Iranian and French (Fra_MC_) populations [43]. Figure 3a shows the UCSC genome browser display of nucleotide variants (vcf file visualization) using the haplotype sorting function implemented in the browser [28]. The displayed window includes tracks for nucleotide diversity π and Tajima’s D for these data and extends to both sides of the *Xpr1* region (*Xpr1* marked in yellow). The sweep in the Iranian population becomes evident as a region of reduced π and negative Tajima’s D covering the extent of the *Xpr1* gene region. In the French population, the haplotype sorter identifies the region of introgression, with a size of a few hundred kb centering around the *Xpr1* gene. This region also shows an elevated π and elevated Tajima’s D, compatible with the introgression scenario.

The relatively small size of the region indicates that recombination has already broken it down to almost gene size, compatible with the time of several hundred years since the introgression event, similar towhat was calculated above. To compare this with a known very recent introgression event, we have chosen the same comparison for the genomic region surrounding *Vkorc1*. This locus was suggested to have adaptively introgressed into Western European mouse populations based on a haplotype that may have been derived from a *M. spretus* population [47] and that conveys resistance against the mouse poisoning substance warfarin. In this case, the onset of adaptive spread would have occurred only a few decades ago and it is indeed evident that the introgressed region is much larger, encompassing several Mb (Fig. 3b).

### Detection of P-MLV virus variants

Given that the *Xpr1* ECL3 receptor variants are relevant for infectivity of different MLV strains [40, 57, 59], we were interested in assessing possible viral variants associated with animals carrying alternative alleles at the N503K substitution in XPR1 (haplotype I/II versus haplotype III). We focused this analysis on sequencing the receptor binding domain (RBD) of the viral protein that interacts with *Xpr1*. We obtained the RBD variants by extracting RNA from feces of wild-derived mice representing the three *M. m. domesticus* populations Fra_MC_, Ger_CB_ and Ira_AH_ and sequencing the respective PCR fragments.

**Fig. 4:**
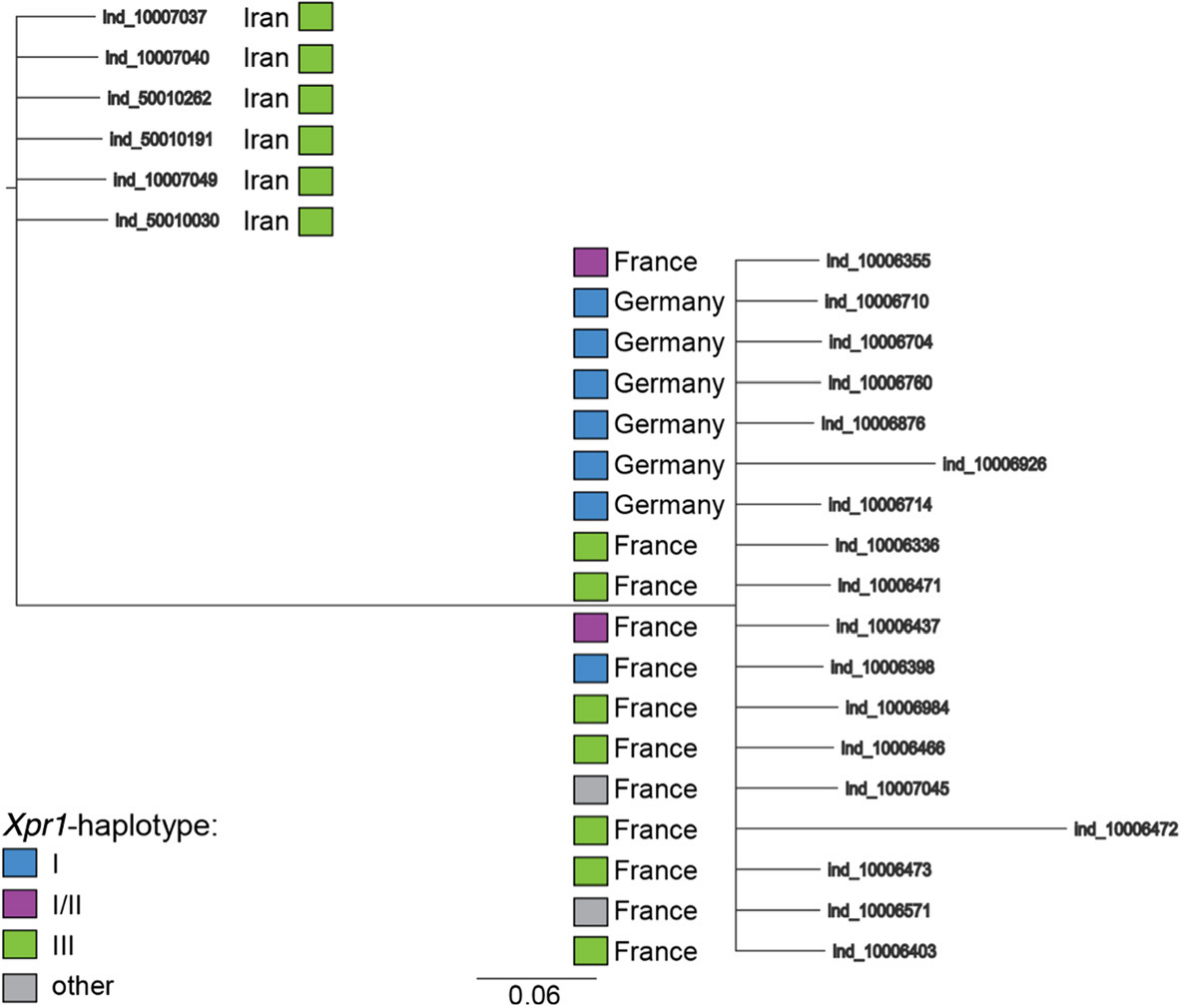
Phylogenetic tree of P-MLV RBD Env sequences from wild-derived mice. Population of origin and Xpr1 haplotype of the respective individuals are indicated next to the samples. The tree was reconstructed using Bayesian analysis of unphased sequences. Polytomies were collapsed

We obtained RBD fragments that were similar to the corresponding region of the MCF247 isolate [27] from 12 samples originating from Fra_MC_ and six samples each from Ger_CB_ and Ira_AH_. We detected 41 sites in the RBD sequence that were variable within or between populations (full sequences in Additional files, overview in Additional file 1: Figure S4). The variable positions were mostly represented by two nucleotides in each animal, suggesting that the transcripts originated from at least two different transcript variants. Phylogenetic analysis shows that the variants from Iranian mice are clearly separated from German and French samples. In contrast, the latter are not clearly separated and cluster independent of their respective *Xpr1* haplotypes (Fig. 4).

**Fig. 5:**
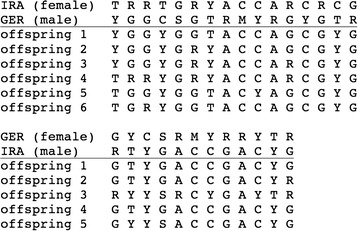
P-MLV RBD *Env* sequence alignments from the reciprocal crossing experiments. Only variable positions are shown, the parental animals are on the top, the offspring animals are on the bottom of each column. Note that different positions were polymorphic for the two pairs of animals. We do not number the positions, since there is no universal reference sequence, but the full sequences are provided in the suppl. Material. All offspring animals show a combination of the parental sequences. Nucleotide and ambiguity codes follow the IUPAC conventions

This observation raises the question of whether transcribed virus variants are tightly associated to their host genomic background, independent of the receptor type, or whether the transcripts originate from non-infectious pro-viruses integrated into the genome. We tested this alternative by setting up a reciprocal cross between Ger_CB_ and Ira_AH_ animals. Mates were tested for their virus variants before and after they were brought together. Each animal retained its own RBD sequence profile, i.e. the mating encounter did not lead to a transfer of active viruses. Males were removed before the offspring was born and females received new bedding. The offspring were then tested for their RBD variants and we found that a composite of the paternal types occurred in the offspring (sequences in Additional files, overview in Fig. 5). This argues against an infectious transmission via the females and favors the pro-virus transmission mechanism. Hence, we find no evidence for an infectious virus in the populations that are currently kept under laboratory conditions.

## Discussion

The overall pattern of *Xpr1* variation in house mouse populations traced here is compatible with the notion of a co-evolution between receptor and infectious viruses. This is also in line with previous inferences that were based on sequence comparisons between sub-species and species (reviewed in [31, 32]). Fourof the populations analyzed here are fixed or almost fixed for a single haplotype (Ger_CB_, Ira_AH_, MUS-CR, and MUS-Kaz) and their overall nucleotide diversity in the *Xpr1* region is 2-6fold lower than the average at other autosomal loci (Table 1). Most of the other populations harbor two rather distinct haplotypes at variable frequencies. This leads to a high Tajima’s D value in some of them, which can be explained by a segregation of two major haplotypes that have come from different source populations. Another possible explanation for the occurrence of two such major haplotypes is balancing selection, but given the overall pattern of recurrent selection, it would seem possible that these haplotypes have formed in independent populations and have come together either by a merging of populations or specific introgression. For the Iranian haplotype, a specific introgression into the Fra_MC_ population is indeed evident, making this latter scenario likely.

*M. m. domesticus* populations arrived approximately 3,000 years ago in Western Europe, most likely traveling across the Mediterranean through Phoenician ships from the near East region [12]. The *M. m. domesticus* population from Iran (Ahvaz area) is currently considered to be the most closely related source population for the mice that arrived in Western Europe [21]. Accordingly, the Iranian haplotypes in the Fra_MC_ population could also be remnants of this original colonization. In this scenario the Iranian haplotypes would have been retained in the Fra_MC_ populations but would have become lost in the populations that spread further across Western Europe. However, the fact that informative alleles at at least two microsatellite loci are identical between the Iranian and Fra_MC_ haplotype refutes this scenario. Instead, the evidence from newly mutated microsatellite alleles, as well as the size of the introgressed haplotype indicates that the introgression happened several hundred years ago.

Long-range introgression of haplotypes has also been shown to occur between populations of the two subspecies *M. m. domesticus* and *M. m. musculus* [48]. At least a fraction of these introgressing haplotypes seem to convey a selective advantage, i.e. have spread adaptively [48]. Introgression has also been observed across species boundaries between *M. m. domesticus* and *M. spretus* [37, 47]. Accordingly, it seems possible to speculate that the Iranian haplotype is also spreading adaptively in the Fra_MC_ population. However, because the two samplings 8 years apart did not show a major frequency shift, the spread may either not be fast, or the adaptive value has already been lost, since the infectious virus is not present any more (see below). Still, given the fact that none of the other population parameters available for the Fra_MC_ population have so far indicated an admixture with Iranian alleles [35, 36, 54], the rather high frequency of the Iranian *Xpr1* haplotype is unusual and would best be explained by invoking a significant positive selection coefficient associated to it. While it is difficult to proof this unequivocally, the assumption fits generally into the pattern of recurrent positive selection on *Xpr1* as we discussed above.

Co-evolution between viruses and the receptor could be invoked in driving this pattern. To date, *Xpr1* alleles were mostly described in a species or subspecies context of *Mus* while variation of alleles at the population level has not been studied in detail so far (for review see [31, 33]). The identified *Xpr1* alleles are characterized by several SNPs in ECL3 and 4 and *M. m. musculus* and *M. m. castaneus* carry unique deletions in ECL4. These deletions occur in most of the identified MLV-restrictive *Xpr1* alleles and have been shown to contribute to resistance phenotypes [40, 60]. One exception is the *Xpr1*^*p*^ variant which was found in *Mus pahari* and which represents a full-length receptor, mediating resistance to P-MLVs [57]. Wild-caught *M. m. domesticus* from the Americas and Europe were described as having a full-length *Xpr1* allele which was called *Xpr1*^*sxv*^ and is apparently permissive to all XP-MLVs tested [32, 59, 60].

In the Western European populations that are studied in this paper, most of the SNPs occur in exon 4, which codes for an intracellular domain of XPR1. This also includes the coding variant that distinguishes the two major Western European haplotypes I and II. We can currently not infer any functional effects of these polymorphisms, since they are not directly involved in the interaction between the ECLs and the RBD of the virus, but we can also not exclude the possibility that they contribute to allele-dependent variation in receptor function.

In contrast to the lack of knowledge about implications of the variation in exon 4, the variable sites in ECL3 and 4 have been shown to be determinants for virus entry or to modulate virus interaction [22, 40, 57, 59, 60]. The ECL3 coding variant at residue N503K that is characteristic for the Iranian haplotype had so far not been described in any other population or species. Accordingly, it has not been specifically tested for its effect on virus interaction, but it represents a possible site for N-linked glycosylation and was suggested to be under positive selection based on a comparison between different species [60]. Glycosylation is known to modulate virus entry in specific virus-cell combinations and many viruses use glycans or cell-surface glycoproteins as attachment molecules [32, 42, 58]. In the Iranian *Xpr1* haplotype the N is substituted by a K, i.e. glycosylation would not be possible. On the other hand, this position is a G in other mammals, including the rat (inferred from the species alignments in the UCSC genome browser, [28]), thus if this site can be glycosylated, it would be mouse specific.

We did not detect infectious virus variants in our mouse colonies and it is therefore not possible to test directly whether the receptor variants convey resistance. Generally, P-MLVs can become infectious if they are activated by ecotropic MLVs (E-MLV) which are known to cause disease in wild and lab mice, but use a different receptor [1, 25]. P-MLV activation involves recombination events which result in the formation of mink cell focus-forming MLVs (MCF-MLV). Those viruses often carry a P-MLV RBD and are oncogenic in some inbred laboratory strains [51]. Endogenous E-MLVs have not been found in *M. m. domesticus* so far [3, 4, 33] which means that the formation of MCF-MLVs would depend on re-occurring exogenous infections. These exogenous E-MLV infections have been shown to play a role in some wild mouse communities, for example in the mice sampled from California at Lake Casitas [15–17]. Yet, no recombination between endogenous MLV-DNA and the infectious E-MLVs has been detected so far in wild mice [5, 45]. Furthermore, wild mice have been shown to be quite resistant to disease induced by P-MLVs and if they do develop disease, general immunity and fertility seem not to be affected [16, 33].

The breeding experiment with wild-derived mice from Ger_CB_ and Ira_AH_ showed that the sequenced virus transcripts were not transmitted as infectious particles but were inherited in a Mendelian fashion, i.e. supporting the notion that they are derived from genomic pro-viruses. Furthermore, the phylogenetic analysis revealed that the separation of the RBD matches the mouse population history, i.e. the pro-virus divergence would have occurred after the separation of the populations. We searched the available genome sequences of house mice, including those of the populations under study, for possible inserts that could be the direct source of the P-MLV transcripts that we detected in the feces. However, an unequivocal assignment was not possible. We found, however, that the MLV inserts show a high turnover between strains and populations. Given that all re-sequenced genomes are usually mapped against the reference sequence of the laboratory strain C57Bl6, new inserts that could be the true source of the transcripts would not have been detected.

Although a causative role of MLV-induced diseases and corresponding resistance evolution seems a likely cause for the observation of recurrent selection at *Xpr1*, not all resistance effects may be mediated through changes to the ECLs. Either intracellular regions, such as the substitution found in exon 4, or expression changes may play a role as well. For example, it is known that virus receptors can be down-regulated by expression of virus ENV glycoproteins, probably to avoid super-infection of cells [13, 55]. Hence, the details of the regulatory response could also be subject to evolutionary resistance development in the receptor gene region. We note that such expression differences of *Xpr1* alleles were indeed identified in the comparison between the Ger_CB_ and Fra_MC_ mice [8]. It might be interesting to analyze the different receptor alleles in the context of their expression levels to gain a more comprehensive picture of *Xpr1*-mediated resistance evolution against virus infection.

Based on all of the available data, we envisage the following scenario for the evolutionary pattern seen in *Xpr1*. Changes at this locus may be driven by short bursts of infection possibly caused by the emergence of disease-causing E-MLV variants with a corresponding build-up of resistance. Although the resistance evolution is likely to also include other loci and genetic processes, it appears to drag along a new *Xpr1* haplotype, which then becomes the most frequent haplotype in the respective population. This would explain the patterns seen in the populations Ger_CB_, Ira_AH_ and MUS-CR with single major haplotypes (haplotypes I, III and V respectively). Secondary contact between populations with differently fixed haplotypes can then result in an exchange of these haplotypes, which would explain the situation in the other populations where two major haplotypes segregate. Among these, we found only four derived haplotypes with single-nucleotide changes, three of them synonymous, and all at low frequency (haplotypes Ia, IIa, IIIa and VIa). This suggests that there are only short phases of neutral accumulation of mutations before a new *Xpr1* haplotype becomes prevalent in a population. The Fra_MC_ population thus appears to represent a transition case caught in the act where a foreign haplotype has started to invade and thus creates a situation with elevated polymorphism. The specific spread of the Iranian *Xpr1* haplotype in this population suggests that it is or has been under positive selection, possibly because it conveys resistance to an infectious virus. However, we have not been able to detect such an infectious virus in our laboratory-bred wild populations, but we can not exclude that it is still present in the natural wild population. Note that only healthy mice were propagated in our colony, i.e. we would have actively selected against an infectious virus causing a disease. Alternatively, given that the introgression of the Iranian haplotype may have occurred a few hundred years ago, it seems possible that the acute phase of the virus infection is over even in the wild population and that we see only the remnants of the resistance evolution that has occurred during this phase.

Specific introgression of an immune relevant allele has also been found in Alpine ibex (*Capra ibex ibex*). In this case an allele of the MHC DRB locus from domestic goats has adaptively introgressed into the wild population of Alpine ibex to contribute to the polymorphism at this locus [19]. Since DRB is involved in pathogen recognition, it may well be that this has also occurred in the context of a transient virus infection.

## Conclusions

The XPR1 receptor is apparently subject to repeated selective sweeps in populations, even more than previously anticipated. We show that a receptor variant has specifically introgressed in one local population, revealing the evolutionary dynamics associated with this receptor. Our results point to regular cycles of retroviral infections and resistance development in wild populations. We therefore expect that allele introgressions related to the exchange of immune relevant genes would occur frequently.

## Additional files

Additional file 1: Table S1. Primers for microsatellite loci. **Table S2**: PCR and sequencing primers. **Table S3**: Allele frequencies of microsatellite loci for all populations. **Figure S1**: SNP haplotype tracks from the SNP survey in Staubach et al. [48]). **Figure S2**: Genomic overview of *Xpr1* on chromosome 1. **Figure S3**: Alignment of *Xpr1* haplotypes from the literature and the present study. **Figure S4**: Alignment of viral RBD haplotypes from the population survey. Xpr1 haplotype sequences from population survey. RBD Sequences of P-MLV variants from the population samples. RBD Sequences of P-MLV variants from the reciprocal crossing experiment (DOCX 1116 kb) Additional file 2: Table S4. Phasing of Xpr1 haplotypes per individual. (XLSX 97 kb)

## Abbreviations

ECL: extracellular loop
MLV: murine leukemia virus
Ms: microsatellite
P-MLV: polytrophic MLV
RBD: receptor-binding domain
X-MLV: xenotropic MLV
Xpr1: xenotropic and polytropic retrovirus receptor 1
