# Supplementary material for: Selective sweeps versus introgression - population genetic dynamics of the murine leukemia virus receptor Xpr1 in wild populations of the house mouse (Mus musculus)
Source: BMC Evol Biol. 2015 Nov 10;15:248. doi: 10.1186/s12862-015-0528-5 (PMC4641351; doi:10.1186/s12862-015-0528-5)
Supplement: Additional file 1: Table S1. — Primers for microsatellite loci. Table S2: PCR and sequencing primers. Table S3: Allele frequencies of microsatellite loci for all populations. Figure S1: SNP haplotype tracks from the SNP survey in Staubach et al. [48]). Figure S2: Genomic overview of Xpr1 on chromosome 1. Figure S3: Alignment of Xpr1 haplotypes from the literature and the present study. Figure S4: Alignment of viral RBD haplotypes from the population survey. Xpr1 haplotype sequences from population survey. RBD Sequences of P-MLV variants from the population samples. RBD Sequences of P-MLV variants from the reciprocal crossing experiment (DOCX 1116 kb) [file 12862_2015_528_MOESM1_ESM.docx]

**Selective sweeps and introgression - population genetic dynamics of the murine leukemia virus receptor *Xpr1* in wild populations of the house mouse (*Mus musculus*)**

Natascha Hasenkamp, Terry Solomon^#^ & Diethard Tautz

Supplementary Material

|  | page |
| --- | --- |
| Table S1: Primers for microsatellite loci. | 2 |
| Table S2: PCR and sequencing primers | 2 |
| Table S3: Allele frequencies of microsatellite loci for all populations | 3 |
| Table S4: Phasing of Xpr1 haplotypes per individual (separate Excel file) |  |
| Figure S1: SNP haplotype tracks from the SNP survey in Staubach et al. (2012). | 5 |
| Figure S2: Genomic overview of Xpr1 on chromosome 1. | 6 |
| Figure S3: Alignment of Xpr1 haplotypes from the literature and the present study. | 7 |
| Figure S4: Alignment of viral RBD haplotypes from the population survey | 7 |
| Xpr1 haplotype sequences from population survey | 11 |
| RBD Sequences of P-MLV variants from the population samples | 38 |
| RBD Sequences of P-MLV variants from the reciprocal crossing experiment | 42 |

Table S1: Primers for microsatellite loci. Primers were designed to allow amplification in four pools (indicated in last column).

| primer ID | sequence | mean product length | pool |
| --- | --- | --- | --- |
| ms1-F | ACAGCTGGCAATTTGGAGAT | 345 | a |
| ms1-R | AGTCAGCTGCCACAACACAG |  |  |
| ms2-F | GAGGAGGGAGGCTCTCAAGT | 240 | b |
| ms2-R | TCCTTTCTGTCACCAATGGAC |  |  |
| ms3-F | TGCAACTCCAGTAACGCAAG | 251 | d |
| ms3-R | GCCTCCTATATGGCCACTCA |  |  |
| ms6-F | TGAAGAAAATTGTCATGAACAAGAA | 266 | c |
| ms6-R | CCAGAGTCACAGTCACAAGCA |  |  |
| ms8-F | ATACAGGCGCCAAGTCCATA | 158 | a |
| ms8-R | GAGTGCCAGGGTTACATGGT |  |  |
| ms10-F | TCCATCACAAGGCTGTATGTG | 147 | b |
| ms10-R | AAACACCGGGCTATGAAATG |  |  |
| ms11-F | CAGCTGCCCAGAGGAGTAAG | 344 | d |
| ms11-R | TTTCATACCTGCAACCCACA |  |  |
| ms13-F | CCCTTTCCCTGGTCTTCAAT | 459 | c |
| ms13-R | CAAGGCTTTTGGAGGGTTTT |  |  |
| ms15-F | GAGGTAGAGCGCATGGATTC | 448 | a |
| ms15-R | TCCCCCAGCTCTACTCTGAA |  |  |

Table S2: PCR and sequencing primers for *Xpr1* genomic regions and the MLV RBD domain. The last column lists the PCR profile modifications.

| primer ID | sequence | elongation time (s) |
| --- | --- | --- |
| ex4-F | GGGCCAAAATGCTTTCTCTT | 30 |
| ex4-R | TGATTTCAATCTTTAGAGGATTCAGT |  |
| ECL3.1-F | TCCATAAGGTAGGCTTTGCTG | 30 |
| ECL3.1-R | TCTTGGTTTATGCTGGCAATC |  |
| ECL3.2-F | CACACACTGATGGGGAGTTG | 30 |
| ECL3.2-R | GCAAAGTCCAGGAAAGCAGA |  |
| ECL3.3-F | TGGGCACTATGAAGAATCCA | 30 |
| ECL3.3-R | GAGACCCCAGTCCATCTTGA |  |
| ECL4-F | AACGCTTCTCCATGAGTCTTTG | 30 |
| ECL4-R | GATCAGACTTGGTATAAGTGTCT |  |
| P-MLV-F | AAGGGCAGGAGTATCAGTACAACAT | 90 |
| P-MLV-R | GGGACGCGGGGCCCTATATTGAG |  |

Table S3: Allele frequencies of microsatellite loci for all populations

|  |  | Ger_CB_ | Ger_SL_ | Fra_AN_ | Fra_NA_ | Fra_LO_ | Fra_ES_ | Fra_DB_ | Fra_MC_ | Ira_AH_ | MUS-CR | MUS-AL |
| --- | --- | --- | --- | --- | --- | --- | --- | --- | --- | --- | --- | --- |
| Xpr1_ms1 | 333 |  |  | 0.10 |  |  |  |  |  |  |  |  |
|  | 337 | 0.25 | 0.30 | 0.45 | 0.45 | 0.33 |  |  |  |  |  |  |
|  | 339 |  |  |  |  |  |  |  |  | 0.05 |  |  |
|  | 341 | 0.75 | 0.70 | 0.45 | 0.50 | 0.63 | 1.00 | 0.96 | 0.39 | 0.05 |  |  |
|  | 345 |  |  |  |  | 0.04 |  | 0.04 | 0.04 | 0.14 | 1.00 | 0.96 |
|  | 347 |  |  |  |  |  |  |  |  | 0.18 |  |  |
|  | 349 |  |  |  | 0.05 |  |  |  | 0.52 | 0.36 |  | 0.04 |
|  | 353 |  |  |  |  |  |  |  | 0.04 | 0.18 |  |  |
|  | 355 |  |  |  |  |  |  |  |  | 0.05 |  |  |
| Xpr1_ms2 | 211 | 0.44 | 0.42 |  | 0.25 | 0.08 |  | 0.46 | 0.02 |  |  |  |
|  | 214 |  |  |  |  |  | 0.04 |  |  |  |  |  |
|  | 220 | 0.17 |  |  | 0.04 | 0.17 |  |  | 0.02 | 0.04 |  |  |
|  | 223 |  | 0.13 | 0.18 | 0.04 | 0.04 | 0.17 | 0.17 | 0.06 | 0.13 |  |  |
|  | 226 | 0.33 | 0.33 | 0.36 | 0.29 | 0.21 | 0.29 | 0.08 | 0.54 | 0.21 |  |  |
|  | 229 | 0.06 |  |  | 0.29 |  | 0.17 | 0.08 | 0.21 | 0.25 | 0.04 | 0.21 |
|  | 232 |  |  | 0.18 | 0.04 | 0.08 |  | 0.08 | 0.10 | 0.13 | 0.54 | 0.33 |
|  | 235 |  |  | 0.14 |  | 0.33 | 0.13 | 0.13 |  | 0.17 | 0.29 | 0.29 |
|  | 238 |  | 0.08 | 0.14 | 0.04 | 0.08 | 0.17 |  | 0.02 | 0.08 | 0.04 | 0.13 |
|  | 241 |  | 0.04 |  |  |  | 0.04 |  | 0.02 |  |  | 0.04 |
|  | 244 |  |  |  |  |  |  |  |  |  | 0.08 |  |
| Xpr1_ms3 | 240 | 0.33 | 0.25 | 0.40 | 0.23 | 0.13 | 0.04 | 0.18 |  |  |  |  |
|  | 244 | 0.25 | 0.20 |  | 0.27 | 0.42 | 0.21 | 0.05 | 0.02 | 0.08 | 0.96 | 0.71 |
|  | 248 |  | 0.10 | 0.25 |  | 0.08 | 0.50 | 0.45 | 0.10 |  |  | 0.29 |
|  | 252 | 0.17 | 0.15 | 0.20 | 0.45 | 0.25 | 0.25 | 0.09 | 0.06 | 0.04 | 0.04 |  |
|  | 256 | 0.25 | 0.25 | 0.05 | 0.05 | 0.13 |  |  | 0.58 | 0.42 |  |  |
|  | 260 |  | 0.05 | 0.10 |  |  |  | 0.05 | 0.10 | 0.38 |  |  |
|  | 264 |  |  |  |  |  |  | 0.18 | 0.13 | 0.08 |  |  |
| Xpr1_ms6 | 263 |  |  |  |  |  |  |  |  | 0.04 |  |  |
|  | 265 |  |  |  |  | 0.04 |  |  | 0.58 | 0.92 |  |  |
|  | 267 | 0.09 |  |  |  | 0.04 |  |  |  |  |  |  |
|  | 269 | 0.32 | 0.35 | 0.23 | 0.42 | 0.04 | 0.38 | 0.13 | 0.19 | 0.04 |  | 0.17 |
|  | 271 | 0.05 | 0.10 |  |  | 0.04 | 0.04 |  | 0.04 |  | 0.75 | 0.79 |
|  | 273 | 0.05 | 0.20 |  |  |  |  | 0.04 | 0.02 |  | 0.25 | 0.04 |
|  | 275 | 0.14 | 0.35 | 0.18 |  | 0.29 | 0.17 | 0.04 | 0.02 |  |  |  |
|  | 277 | 0.32 |  | 0.50 | 0.58 | 0.38 | 0.25 | 0.38 | 0.08 |  |  |  |
|  | 279 |  |  | 0.09 |  | 0.17 | 0.13 | 0.13 |  |  |  |  |
|  | 281 | 0.05 |  |  |  |  |  | 0.13 |  |  |  |  |
|  | 283 |  |  |  |  |  | 0.04 | 0.08 | 0.06 |  |  |  |
|  | 285 |  |  |  |  |  |  | 0.04 |  |  |  |  |
|  | 289 |  |  |  |  |  |  | 0.04 |  |  |  |  |
| Xpr1_ms8 | 139 | 1.00 | 1.00 | 1.00 | 1.00 | 1.00 | 1.00 | 0.92 | 0.27 |  |  |  |
|  | 153 |  |  |  |  |  |  |  |  | 0.14 |  |  |
|  | 157 |  |  |  |  |  |  | 0.08 | 0.71 | 0.77 |  |  |
|  | 161 |  |  |  |  |  |  |  | 0.02 | 0.09 |  |  |
|  | 165 |  |  |  |  |  |  |  |  |  | 0.25 | 0.04 |
|  | 169 |  |  |  |  |  |  |  |  |  | 0.54 | 0.71 |
|  | 173 |  |  |  |  |  |  |  |  |  | 0.21 | 0.17 |
|  | 177 |  |  |  |  |  |  |  |  |  |  | 0.08 |
| Xpr1_ms10 | 119 |  |  |  |  |  |  |  | 0.02 |  |  |  |
|  | 127 |  | 0.08 |  |  |  | 0.04 | 0.08 |  |  |  |  |
|  | 131 | 0.35 | 0.25 | 0.38 | 0.13 | 0.17 | 0.13 | 0.04 | 0.02 | 0.04 |  |  |
|  | 135 | 0.15 | 0.21 | 0.21 | 0.33 | 0.42 | 0.46 | 0.46 | 0.09 | 0.21 |  |  |
|  | 139 |  | 0.04 | 0.33 | 0.13 | 0.17 | 0.29 | 0.04 | 0.26 | 0.54 | 0.04 | 0.46 |
|  | 143 | 0.05 | 0.29 | 0.08 | 0.38 | 0.17 |  | 0.33 | 0.37 | 0.04 | 0.08 | 0.13 |
|  | 147 | 0.45 | 0.13 |  | 0.04 | 0.08 | 0.08 |  | 0.17 | 0.17 | 0.58 | 0.21 |
|  | 151 |  |  |  |  |  |  | 0.04 | 0.02 |  | 0.13 | 0.04 |
|  | 155 |  |  |  |  |  |  |  | 0.04 |  | 0.17 | 0.17 |
| Xpr1_ms11 | 330 |  |  |  |  |  |  |  | 0.02 |  |  |  |
|  | 334 |  |  |  |  |  |  |  | 0.02 |  |  |  |
|  | 342 |  |  |  | 0.13 |  |  |  |  |  |  |  |
|  | 344 |  |  |  | 0.04 |  | 0.04 |  | 0.02 |  |  |  |
|  | 348 |  |  | 0.05 | 0.04 | 0.04 | 0.04 |  | 0.07 | 0.04 |  |  |
|  | 350 |  | 0.10 |  |  | 0.04 |  |  | 0.07 |  |  |  |
|  | 352 | 0.13 |  |  |  |  |  | 0.05 |  | 0.21 |  |  |
|  | 354 | 0.06 |  | 0.10 | 0.08 |  |  |  | 0.02 | 0.04 |  |  |
|  | 356 | 0.06 |  | 0.05 | 0.08 | 0.38 | 0.21 | 0.14 | 0.02 | 0.13 |  | 0.08 |
|  | 358 | 0.38 | 0.15 | 0.60 | 0.33 | 0.29 | 0.29 | 0.14 | 0.16 | 0.25 | 0.23 | 0.04 |
|  | 360 | 0.13 | 0.10 | 0.05 | 0.17 | 0.17 | 0.08 | 0.18 | 0.05 | 0.21 | 0.23 | 0.08 |
|  | 362 | 0.13 | 0.35 |  |  |  |  | 0.18 | 0.05 | 0.04 | 0.18 | 0.17 |
|  | 364 | 0.13 | 0.15 | 0.05 | 0.04 |  | 0.17 | 0.18 | 0.11 | 0.08 | 0.05 | 0.17 |
|  | 366 |  |  | 0.05 | 0.04 | 0.04 |  | 0.09 | 0.11 |  |  | 0.25 |
|  | 368 |  | 0.05 |  | 0.04 | 0.04 | 0.17 | 0.05 | 0.11 |  | 0.05 | 0.13 |
|  | 370 |  |  | 0.05 |  |  |  |  | 0.16 |  |  |  |
|  | 372 |  | 0.10 |  |  |  |  |  |  |  | 0.23 |  |
|  | 376 |  |  |  |  |  |  |  |  |  | 0.05 |  |
|  | 378 |  |  |  |  |  |  |  |  |  |  | 0.08 |
| Xpr1_ms13 | 427 | 0.39 | 0.25 | 0.32 | 0.25 | 0.23 | 0.04 | 0.08 | 0.06 | 0.04 |  |  |
|  | 431 | 0.06 |  | 0.05 | 0.17 |  | 0.13 | 0.08 |  | 0.04 |  |  |
|  | 435 | 0.11 | 0.40 | 0.14 | 0.13 | 0.32 | 0.33 | 0.46 | 0.13 | 0.42 |  |  |
|  | 439 | 0.39 | 0.30 | 0.27 | 0.33 | 0.27 | 0.17 | 0.38 | 0.31 | 0.42 | 0.17 |  |
|  | 443 | 0.06 | 0.05 | 0.09 | 0.13 | 0.18 | 0.21 |  | 0.40 | 0.08 | 0.58 | 0.04 |
|  | 447 |  |  | 0.14 |  |  | 0.13 |  | 0.06 |  | 0.13 | 0.29 |
|  | 451 |  |  |  |  |  |  |  | 0.04 |  | 0.13 | 0.25 |
|  | 455 |  |  |  |  |  |  |  |  |  |  | 0.17 |
|  | 457 |  |  |  |  |  |  |  |  |  |  |  |
|  | 461 |  |  |  |  |  |  |  |  |  |  | 0.17 |
|  | 465 |  |  |  |  |  |  |  |  |  |  | 0.04 |
|  | 469 |  |  |  |  |  |  |  |  |  |  | 0.04 |
| Xpr1_ms15 | 441 | 0.20 |  | 0.35 | 0.15 | 0.04 |  |  |  | 0.05 |  |  |
|  | 443 |  | 0.32 |  | 0.05 |  |  |  |  | 0.05 |  |  |
|  | 445 |  |  | 0.05 |  |  |  |  |  | 0.32 |  |  |
|  | 447 |  |  |  |  | 0.08 |  |  | 0.56 | 0.09 |  |  |
|  | 449 |  |  |  |  |  |  | 0.04 | 0.04 | 0.14 |  |  |
|  | 451 |  |  |  |  | 0.04 |  | 0.04 | 0.06 | 0.23 |  |  |
|  | 453 |  | 0.05 | 0.05 | 0.15 | 0.04 | 0.25 | 0.29 | 0.04 | 0.14 |  |  |
|  | 455 |  |  | 0.10 |  | 0.08 |  | 0.04 | 0.02 |  |  |  |
|  | 457 |  | 0.23 | 0.20 | 0.25 | 0.33 | 0.33 | 0.08 | 0.08 |  |  |  |
|  | 459 | 0.40 | 0.14 | 0.15 | 0.05 | 0.25 | 0.13 | 0.04 | 0.04 |  |  | 0.08 |
|  | 461 | 0.35 |  | 0.05 | 0.30 |  | 0.17 | 0.17 | 0.04 |  | 0.88 | 0.92 |
|  | 463 |  | 0.09 | 0.05 | 0.05 | 0.08 | 0.13 | 0.21 |  |  | 0.08 |  |
|  | 465 | 0.05 | 0.09 |  |  | 0.04 |  | 0.08 | 0.08 |  | 0.04 |  |
|  | 467 |  | 0.09 |  |  |  |  |  | 0.02 |  |  |  |
|  |  |  |  |  |  |  |  |  |  |  |  |  |

Supplementary Figures


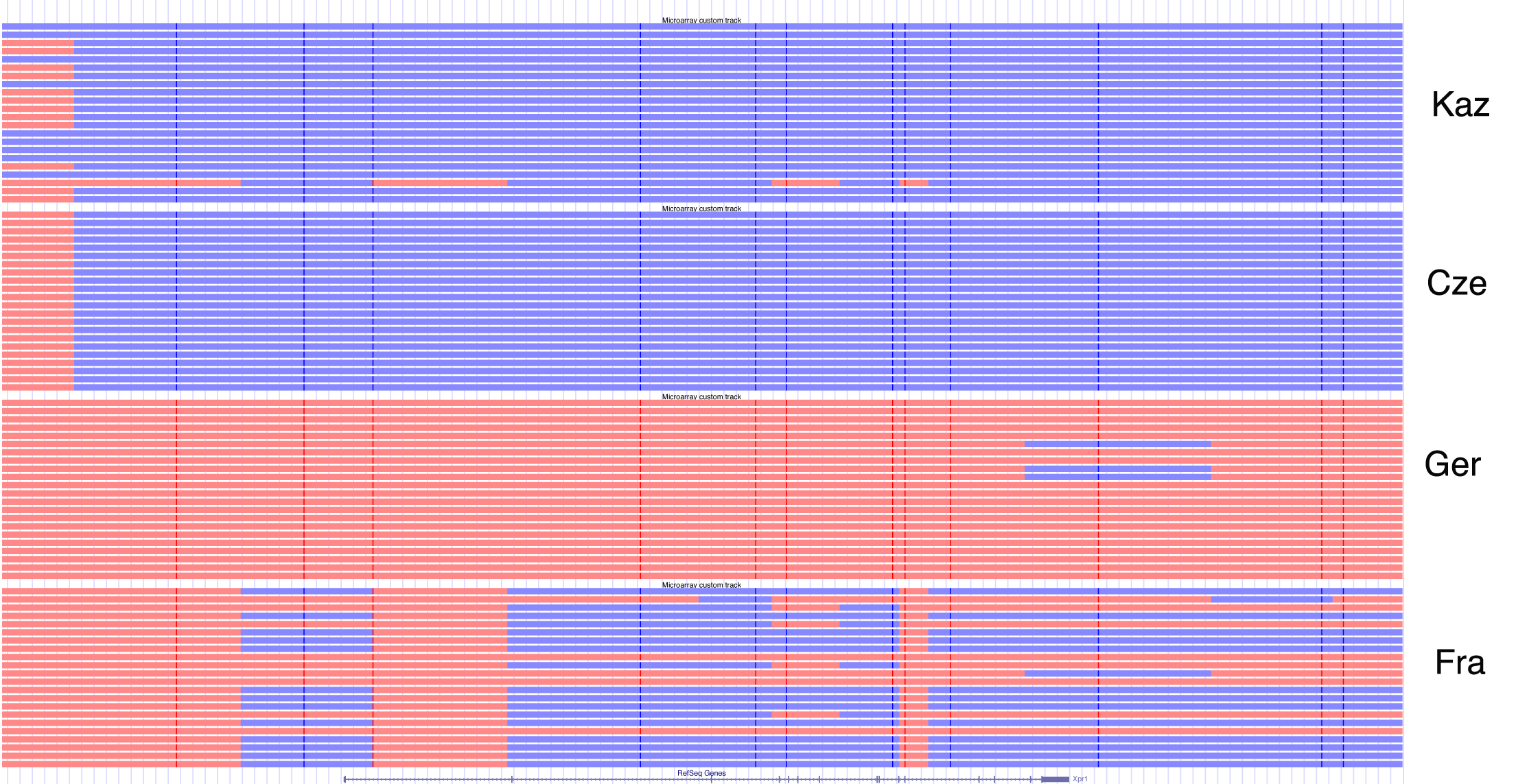


Figure S1: SNP haplotype tracks from the SNP survey in Staubach et al. (2012). The picture shows the genomic region around *Xpr1* (chr1:157057668-157331808 in the NCBI37/mm9 assembly) for the four surveyed populations (Fra and Ger = *M. m. domesticus* from France and Germany, Cze and Kaz = *M. m. musculus* from Czech Republic and Kazakhstan). Blue and red represent the different allele states, consecutive stretches of the same allele state suggest recent fixation of a given haplotype (see Staubach et al. 2012 for further description of this display mode). This is the case for the three populations on the top, while the Fra population at the bottom is polymorphic.


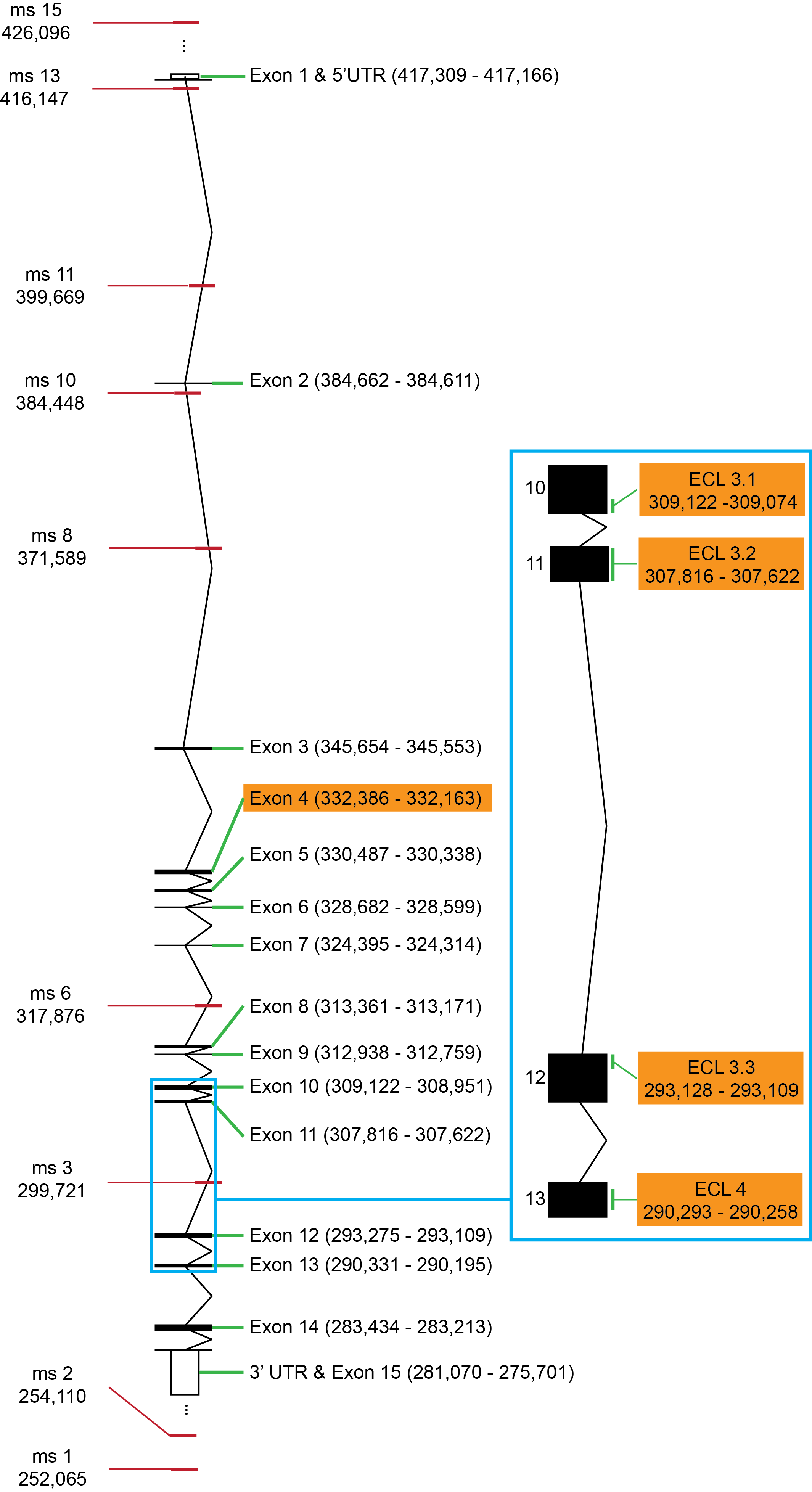


Figure S2: Genomic overview of *Xpr1* on chromosome 1. The exons are shown by black boxes, connected by lines that indicate the introns. The location of the microsatellite loci analyzed is indicated by red boxes. Their respective genomic positions are indicated next to the boxes. An enlarged view of exons 10 to 13 is shown as a box on the right.


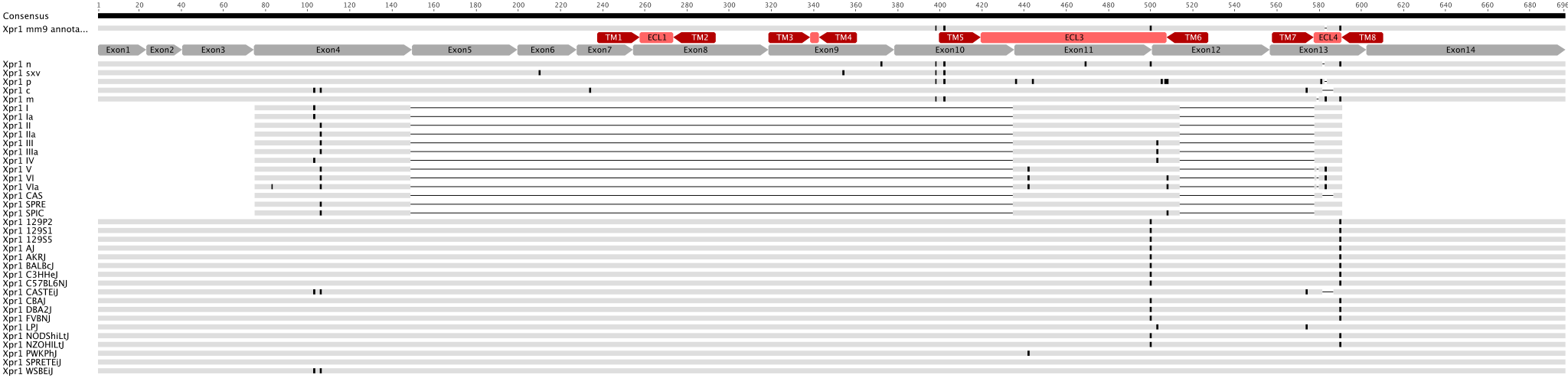


Figure S3: Alignment of *Xpr1* haplotypes from the literature and database (full sequences) and the partial sequences from the population samples in the present study (roman numerals corresponding to the haplotypes in Table 1). The exon structure of the gene is displayed on the top and transmembrane regions (TM), as well as extracellular loops (ECL) are marked by red boxes.


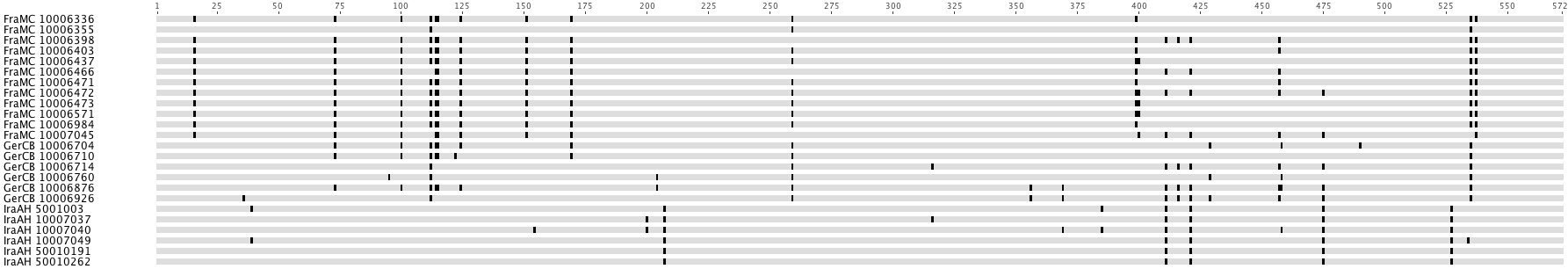


Figure S4: Alignment of viral RBD haplotypes from the population survey

**Xpr1 haplotype sequences from population survey**

>Fra-AN1301-1

GAGAAGCTGGCAGAGGCTCAGCGCAGGTTTGCTACACTTCAGAATGAGCTTCAGTCATCGCTGGATGTGCAGAAAGAAAGCAGTGcTGTCACagCAtTGCGACAgCGCAGAAAGCCAGTGTTCCACCTGTCCCACGAGGAGCGtGTCCAACATAGGAAcATTAAAGACCTTAAGCTGGCCTTCaGCGAGTTCTACCTCAGTCTCATCCTGCTGCAGAACGAACCAGAATTTTGCCACaAATATTCATATGGTGTGCGAGCCATTGTTCAATGTATTCCAGCTTGGCTTCGCTTCATCCAGTGCCTGCGCCGGTACCGTGACACGAGGAGGGCTTTTCCTCATCTAGTAAATGCTGGCAAATACTCcACAACTTTCTTCACcGTGACATTTGCAGCCCTTTACAGCACTCACAAACCTCCTCTTTCTCCAGAACAAAAtCACTCTGACACCgtGGTgTTCTTTTACCTGTCTATTACTGCTACAACGTTTAAGCCTCATGTTGGGGAC

>Fra-AN1301-2

GAGAAGCTGGCAGAGGCTCAGCGCAGGTTTGCTACACTTCAGAATGAGCTTCAGTCATCGCTGGATGTGCAGAAAGAAAGCAGTGcTGTCACagCAtTGCGACAgCGCAGAAAGCCAGTGTTCCACCTGTCCCACGAGGAGCGtGTCCAACATAGGAAcATTAAAGACCTTAAGCTGGCCTTCaGCGAGTTCTACCTCAGTCTCATCCTGCTGCAGAACGAACCAGAATTTTGCCACaAATATTCATATGGTGTGCGAGCCATTGTTCAATGTATTCCAGCTTGGCTTCGCTTCATCCAGTGCCTGCGCCGGTACCGTGACACGAGGAGGGCTTTTCCTCATCTAGTAAATGCTGGCAAATACTCcACAACTTTCTTCACcGTGACATTTGCAGCCCTTTACAGCACTCACAAACCTCCTCTTTCTCCAGAACAAAAtCACTCTGACACCgtGGTgTTCTTTTACCTGTCTATTACTGCTACAACGTTTAAGCCTCATGTTGGGGAC

>Fra-AN55-1

GAGAAGCTGGCAGAGGCTCAGCGCAGGTTTGCTACACTTCAGAATGAGCTTCAGTCATCGCTGGATGTGCAGAAAGAAAGCAGTGcTGTCACagCAtTGCGACAgCGCAGAAAGCCAGTGTTCCACCTGTCCCACGAGGAGCGtGTCCAACATAGGAAcATTAAAGACCTTAAGCTGGCCTTCaGCGAGTTCTACCTCAGTCTCATCCTGCTGCAGAACGAACCAGAATTTTGCCACaAATATTCATATGGTGTGCGAGCCATTGTTCAATGTATTCCAGCTTGGCTTCGCTTCATCCAGTGCCTGCGCCGGTACCGTGACACGAGGAGGGCTTTTCCTCATCTAGTAAATGCTGGCAAATACTCcACAACTTTCTTCACcGTGACATTTGCAGCCCTTTACAGCACTCACAAACCTCCTCTTTCTCCAGAACAAAAtCACTCTGACACCgtGGTgTTCTTTTACCTGTCTATTACTGCTACAACGTTTAAGCCTCATGTTGGGGAC

>Fra-AN55-2

GAGAAGCTGGCAGAGGCTCAGCGCAGGTTTGCTACACTTCAGAATGAGCTTCAGTCATCGCTGGATGTGCAGAAAGAAAGCAGTGcTGTCACagCAtTGCGACAgCGCAGAAAGCCAGTGTTCCACCTGTCCCACGAGGAGCGtGTCCAACATAGGAAcATTAAAGACCTTAAGCTGGCCTTCaGCGAGTTCTACCTCAGTCTCATCCTGCTGCAGAACGAACCAGAATTTTGCCACaAATATTCATATGGTGTGCGAGCCATTGTTCAATGTATTCCAGCTTGGCTTCGCTTCATCCAGTGCCTGCGCCGGTACCGTGACACGAGGAGGGCTTTTCCTCATCTAGTAAATGCTGGCAAATACTCcACAACTTTCTTCACcGTGACATTTGCAGCCCTTTACAGCACTCACAAACCTCCTCTTTCTCCAGAACAAAAtCACTCTGACACCgtGGTgTTCTTTTACCTGTCTATTACTGCTACAACGTTTAAGCCTCATGTTGGGGAC

>Fra-AN2601-1

GAGAAGCTGGCAGAGGCTCAGCGCAGGTTTGCTACACTTCAGAATGAGCTTCAGTCATCGCTGGATGTGCAGAAAGAAAGCAGTGcTGTCACagCAtTGCGACAgCGCAGAAAGCCAGTGTTCCACCTGTCCCACGAGGAGCGtGTCCAACATAGGAAcATTAAAGACCTTAAGCTGGCCTTCaGCGAGTTCTACCTCAGTCTCATCCTGCTGCAGAACGAACCAGAATTTTGCCACaAATATTCATATGGTGTGCGAGCCATTGTTCAATGTATTCCAGCTTGGCTTCGCTTCATCCAGTGCCTGCGCCGGTACCGTGACACGAGGAGGGCTTTTCCTCATCTAGTAAATGCTGGCAAATACTCcACAACTTTCTTCACcGTGACATTTGCAGCCCTTTACAGCACTCACAAACCTCCTCTTTCTCCAGAACAAAAtCACTCTGACACCgtGGTgTTCTTTTACCTGTCTATTACTGCTACAACGTTTAAGCCTCATGTTGGGGAC

>Fra-AN2601-2

GAGAAGCTGGCAGAGGCTCAGCGCAGGTTTGCTACACTTCAGAATGAGCTTCAGTCATCGCTGGATGTGCAGAAAGAAAGCAGTGcTGTCACagCAtTGCGACAgCGCAGAAAGCCAGTGTTCCACCTGTCCCACGAGGAGCGtGTCCAACATAGGAAcATTAAAGACCTTAAGCTGGCCTTCaGCGAGTTCTACCTCAGTCTCATCCTGCTGCAGAACGAACCAGAATTTTGCCACaAATATTCATATGGTGTGCGAGCCATTGTTCAATGTATTCCAGCTTGGCTTCGCTTCATCCAGTGCCTGCGCCGGTACCGTGACACGAGGAGGGCTTTTCCTCATCTAGTAAATGCTGGCAAATACTCcACAACTTTCTTCACcGTGACATTTGCAGCCCTTTACAGCACTCACAAACCTCCTCTTTCTCCAGAACAAAAtCACTCTGACACCgtGGTgTTCTTTTACCTGTCTATTACTGCTACAACGTTTAAGCCTCATGTTGGGGAC

>Fra-AN3101-1

GAGAAGCTGGCAGAGGCTCAGCGCAGGTTTGCTACACTTCAGAATGAGCTTCAGTCATCGCTGGATGTGCAGAAAGAAAGCAGTGcTGTCACagCAtTGCGACAgCGCAGAAAGCCAGTGTTCCACCTGTCCCACGAGGAGCGtGTCCAACATAGGAAcATTAAAGACCTTAAGCTGGCCTTCaGCGAGTTCTACCTCAGTCTCATCCTGCTGCAGAACGAACCAGAATTTTGCCACaAATATTCATATGGTGTGCGAGCCATTGTTCAATGTATTCCAGCTTGGCTTCGCTTCATCCAGTGCCTGCGCCGGTACCGTGACACGAGGAGGGCTTTTCCTCATCTAGTAAATGCTGGCAAATACTCcACAACTTTCTTCACcGTGACATTTGCAGCCCTTTACAGCACTCACAAACCTCCTCTTTCTCCAGAACAAAAtCACTCTGACACCgtGGTgTTCTTTTACCTGTCTATTACTGCTACAACGTTTAAGCCTCATGTTGGGGAC

>Fra-AN3101-2

GAGAAGCTGGCAGAGGCTCAGCGCAGGTTTGCTACACTTCAGAATGAGCTTCAGTCATCGCTGGATGTGCAGAAAGAAAGCAGTGcTGTCACagCAtTGCGACAgCGCAGAAAGCCAGTGTTCCACCTGTCCCACGAGGAGCGtGTCCAACATAGGAAcATTAAAGACCTTAAGCTGGCCTTCaGCGAGTTCTACCTCAGTCTCATCCTGCTGCAGAACGAACCAGAATTTTGCCACaAATATTCATATGGTGTGCGAGCCATTGTTCAATGTATTCCAGCTTGGCTTCGCTTCATCCAGTGCCTGCGCCGGTACCGTGACACGAGGAGGGCTTTTCCTCATCTAGTAAATGCTGGCAAATACTCcACAACTTTCTTCACcGTGACATTTGCAGCCCTTTACAGCACTCACAAACCTCCTCTTTCTCCAGAACAAAAtCACTCTGACACCgtGGTgTTCTTTTACCTGTCTATTACTGCTACAACGTTTAAGCCTCATGTTGGGGAC

>Fra-AN3001-1

GAGAAGCTGGCAGAGGCTCAGCGCAGGTTTGCTACACTTCAGAATGAGCTTCAGTCATCGCTGGATGTGCAGAAAGAAAGCAGTGcTGTCACagCAtTGCGACAgCGCAGAAAGCCAGTGTTCCACCTGTCCCACGAGGAGCGtGTCCAACATAGGAAcATTAAAGACCTTAAGCTGGCCTTCaGCGAGTTCTACCTCAGTCTCATCCTGCTGCAGAACGAACCAGAATTTTGCCACaAATATTCATATGGTGTGCGAGCCATTGTTCAATGTATTCCAGCTTGGCTTCGCTTCATCCAGTGCCTGCGCCGGTACCGTGACACGAGGAGGGCTTTTCCTCATCTAGTAAATGCTGGCAAATACTCcACAACTTTCTTCACcGTGACATTTGCAGCCCTTTACAGCACTCACAAACCTCCTCTTTCTCCAGAACAAAAtCACTCTGACACCgtGGTgTTCTTTTACCTGTCTATTACTGCTACAACGTTTAAGCCTCATGTTGGGGAC

>Fra-AN3001-2

GAGAAGCTGGCAGAGGCTCAGCGCAGGTTTGCTACACTTCAGAATGAGCTTCAGTCATCGCTGGATGTGCAGAAAGAAAGCAGTGcTGTCACagCAtTGCGACAgCGCAGAAAGCCAGTGTTCCACCTGTCCCACGAGGAGCGtGTCCAACATAGGAAcATTAAAGACCTTAAGCTGGCCTTCaGCGAGTTCTACCTCAGTCTCATCCTGCTGCAGAACGAACCAGAATTTTGCCACaAATATTCATATGGTGTGCGAGCCATTGTTCAATGTATTCCAGCTTGGCTTCGCTTCATCCAGTGCCTGCGCCGGTACCGTGACACGAGGAGGGCTTTTCCTCATCTAGTAAATGCTGGCAAATACTCcACAACTTTCTTCACcGTGACATTTGCAGCCCTTTACAGCACTCACAAACCTCCTCTTTCTCCAGAACAAAAtCACTCTGACACCgtGGTgTTCTTTTACCTGTCTATTACTGCTACAACGTTTAAGCCTCATGTTGGGGAC

>Fra-AN2901-1

GAGAAGCTGGCAGAGGCTCAGCGCAGGTTTGCTACACTTCAGAATGAGCTTCAGTCATCGCTGGATGTGCAGAAAGAAAGCAGTGcTGTCACagCAtTGCGACAgCGCAGAAAGCCAGTGTTCCACCTGTCCCACGAGGAGCGtGTCCAACATAGGAAcATTAAAGACCTTAAGCTGGCCTTCaGCGAGTTCTACCTCAGTCTCATCCTGCTGCAGAACGAACCAGAATTTTGCCACaAATATTCATATGGTGTGCGAGCCATTGTTCAATGTATTCCAGCTTGGCTTCGCTTCATCCAGTGCCTGCGCCGGTACCGTGACACGAGGAGGGCTTTTCCTCATCTAGTAAATGCTGGCAAATACTCcACAACTTTCTTCACcGTGACATTTGCAGCCCTTTACAGCACTCACAAACCTCCTCTTTCTCCAGAACAAAAtCACTCTGACACCgtGGTgTTCTTTTACCTGTCTATTACTGCTACAACGTTTAAGCCTCATGTTGGGGAC

>Fra-AN2901-2

GAGAAGCTGGCAGAGGCTCAGCGCAGGTTTGCTACACTTCAGAATGAGCTTCAGTCATCGCTGGATGTGCAGAAAGAAAGCAGTGcTGTCACagCAtTGCGACAgCGCAGAAAGCCAGTGTTCCACCTGTCCCACGAGGAGCGtGTCCAACATAGGAAcATTAAAGACCTTAAGCTGGCCTTCaGCGAGTTCTACCTCAGTCTCATCCTGCTGCAGAACGAACCAGAATTTTGCCACaAATATTCATATGGTGTGCGAGCCATTGTTCAATGTATTCCAGCTTGGCTTCGCTTCATCCAGTGCCTGCGCCGGTACCGTGACACGAGGAGGGCTTTTCCTCATCTAGTAAATGCTGGCAAATACTCcACAACTTTCTTCACcGTGACATTTGCAGCCCTTTACAGCACTCACAAACCTCCTCTTTCTCCAGAACAAAAtCACTCTGACACCgtGGTgTTCTTTTACCTGTCTATTACTGCTACAACGTTTAAGCCTCATGTTGGGGAC

>Fra-AN0801-1

GAGAAGCTGGCAGAGGCTCAGCGCAGGTTTGCTACACTTCAGAATGAGCTTCAGTCATCGCTGGATGTGCAGAAAGAAAGCAGTGcTGTCACagCAtTGCGACAgCGCAGAAAGCCAGTGTTCCACCTGTCCCACGAGGAGCGtGTCCAACATAGGAAcATTAAAGACCTTAAGCTGGCCTTCaGCGAGTTCTACCTCAGTCTCATCCTGCTGCAGAACGAACCAGAATTTTGCCACaAATATTCATATGGTGTGCGAGCCATTGTTCAATGTATTCCAGCTTGGCTTCGCTTCATCCAGTGCCTGCGCCGGTACCGTGACACGAGGAGGGCTTTTCCTCATCTAGTAAATGCTGGCAAATACTCcACAACTTTCTTCACcGTGACATTTGCAGCCCTTTACAGCACTCACAAACCTCCTCTTTCTCCAGAACAAAAtCACTCTGACACCgtGGTgTTCTTTTACCTGTCTATTACTGCTACAACGTTTAAGCCTCATGTTGGGGAC

>Fra-AN0801-2

GAGAAGCTGGCAGAGGCTCAGCGCAGGTTTGCTACACTTCAGAATGAGCTTCAGTCATCGCTGGATGTGCAGAAAGAAAGCAGTGgTGTCACaaCAcTGCGACAgCGCAGAAAGCCAGTGTTCCACCTGTCCCACGAGGAGCGtGTCCAACATAGGAAcATTAAAGACCTTAAGCTGGCCTTCaGCGAGTTCTACCTCAGTCTCATCCTGCTGCAGAACGAACCAGAATTTTGCCACaAATATTCATATGGTGTGCGAGCCATTGTTCAATGTATTCCAGCTTGGCTTCGCTTCATCCAGTGCCTGCGCCGGTACCGTGACACGAGGAGGGCTTTTCCTCATCTAGTAAATGCTGGCAAATACTCcACAACTTTCTTCACcGTGACATTTGCAGCCCTTTACAGCACTCACAAACCTCCTCTTTCTCCAGAACAAAAtCACTCTGACACCgtGGTgTTCTTTTACCTGTCTATTACTGCTACAACGTTTAAGCCTCATGTTGGGGAC

>Fra-AN1501-1

GAGAAGCTGGCAGAGGCTCAGCGCAGGTTTGCTACACTTCAGAATGAGCTTCAGTCATCGCTGGATGTGCAGAAAGAAAGCAGTGcTGTCACagCAtTGCGACAgCGCAGAAAGCCAGTGTTCCACCTGTCCCACGAGGAGCGtGTCCAACATAGGAAcATTAAAGACCTTAAGCTGGCCTTCaGCGAGTTCTACCTCAGTCTCATCCTGCTGCAGAACGAACCAGAATTTTGCCACaAATATTCATATGGTGTGCGAGCCATTGTTCAATGTATTCCAGCTTGGCTTCGCTTCATCCAGTGCCTGCGCCGGTACCGTGACACGAGGAGGGCTTTTCCTCATCTAGTAAATGCTGGCAAATACTCcACAACTTTCTTCACcGTGACATTTGCAGCCCTTTACAGCACTCACAAACCTCCTCTTTCTCCAGAACAAAAtCACTCTGACACCgtGGTgTTCTTTTACCTGTCTATTACTGCTACAACGTTTAAGCCTCATGTTGGGGAC

>Fra-AN1501-2

GAGAAGCTGGCAGAGGCTCAGCGCAGGTTTGCTACACTTCAGAATGAGCTTCAGTCATCGCTGGATGTGCAGAAAGAAAGCAGTGgTGTCACaaCAcTGCGACAgCGCAGAAAGCCAGTGTTCCACCTGTCCCACGAGGAGCGtGTCCAACATAGGAAcATTAAAGACCTTAAGCTGGCCTTCaGCGAGTTCTACCTCAGTCTCATCCTGCTGCAGAACGAACCAGAATTTTGCCACaAATATTCATATGGTGTGCGAGCCATTGTTCAATGTATTCCAGCTTGGCTTCGCTTCATCCAGTGCCTGCGCCGGTACCGTGACACGAGGAGGGCTTTTCCTCATCTAGTAAATGCTGGCAAATACTCcACAACTTTCTTCACcGTGACATTTGCAGCCCTTTACAGCACTCACAAACCTCCTCTTTCTCCAGAACAAAAtCACTCTGACACCgtGGTgTTCTTTTACCTGTCTATTACTGCTACAACGTTTAAGCCTCATGTTGGGGAC

>Fra-AN2301-1

GAGAAGCTGGCAGAGGCTCAGCGCAGGTTTGCTACACTTCAGAATGAGCTTCAGTCATCGCTGGATGTGCAGAAAGAAAGCAGTGcTGTCACagCAtTGCGACAgCGCAGAAAGCCAGTGTTCCACCTGTCCCACGAGGAGCGtGTCCAACATAGGAAcATTAAAGACCTTAAGCTGGCCTTCaGCGAGTTCTACCTCAGTCTCATCCTGCTGCAGAACGAACCAGAATTTTGCCACaAATATTCATATGGTGTGCGAGCCATTGTTCAATGTATTCCAGCTTGGCTTCGCTTCATCCAGTGCCTGCGCCGGTACCGTGACACGAGGAGGGCTTTTCCTCATCTAGTAAATGCTGGCAAATACTCcACAACTTTCTTCACcGTGACATTTGCAGCCCTTTACAGCACTCACAAACCTCCTCTTTCTCCAGAACAAAAtCACTCTGACACCgtGGTgTTCTTTTACCTGTCTATTACTGCTACAACGTTTAAGCCTCATGTTGGGGAC

>Fra-AN2301-2

GAGAAGCTGGCAGAGGCTCAGCGCAGGTTTGCTACACTTCAGAATGAGCTTCAGTCATCGCTGGATGTGCAGAAAGAAAGCAGTGgTGTCACaaCAcTGCGACAgCGCAGAAAGCCAGTGTTCCACCTGTCCCACGAGGAGCGtGTCCAACATAGGAAcATTAAAGACCTTAAGCTGGCCTTCaGCGAGTTCTACCTCAGTCTCATCCTGCTGCAGAACGAACCAGAATTTTGCCACaAATATTCATATGGTGTGCGAGCCATTGTTCAATGTATTCCAGCTTGGCTTCGCTTCATCCAGTGCCTGCGCCGGTACCGTGACACGAGGAGGGCTTTTCCTCATCTAGTAAATGCTGGCAAATACTCcACAACTTTCTTCACcGTGACATTTGCAGCCCTTTACAGCACTCACAAACCTCCTCTTTCTCCAGAACAAAAtCACTCTGACACCgtGGTgTTCTTTTACCTGTCTATTACTGCTACAACGTTTAAGCCTCATGTTGGGGAC

>Fra-AN1401-1

GAGAAGCTGGCAGAGGCTCAGCGCAGGTTTGCTACACTTCAGAATGAGCTTCAGTCATCGCTGGATGTGCAGAAAGAAAGCAGTGgTGTCACaaCAcTGCGACAgCGCAGAAAGCCAGTGTTCCACCTGTCCCACGAGGAGCGtGTCCAACATAGGAAcATTAAAGACCTTAAGCTGGCCTTCaGCGAGTTCTACCTCAGTCTCATCCTGCTGCAGAACGAACCAGAATTTTGCCACaAATATTCATATGGTGTGCGAGCCATTGTTCAATGTATTCCAGCTTGGCTTCGCTTCATCCAGTGCCTGCGCCGGTACCGTGACACGAGGAGGGCTTTTCCTCATCTAGTAAATGCTGGCAAATACTCcACAACTTTCTTCACcGTGACATTTGCAGCCCTTTACAGCACTCACAAACCTCCTCTTTCTCCAGAACAAAAtCACTCTGACACCgtGGTgTTCTTTTACCTGTCTATTACTGCTACAACGTTTAAGCCTCATGTTGGGGAC

>Fra-AN1401-2

GAGAAGCTGGCAGAGGCTCAGCGCAGGTTTGCTACACTTCAGAATGAGCTTCAGTCATCGCTGGATGTGCAGAAAGAAAGCAGTGgTGTCACaaCAcTGCGACAgCGCAGAAAGCCAGTGTTCCACCTGTCCCACGAGGAGCGtGTCCAACATAGGAAcATTAAAGACCTTAAGCTGGCCTTCaGCGAGTTCTACCTCAGTCTCATCCTGCTGCAGAACGAACCAGAATTTTGCCACaAATATTCATATGGTGTGCGAGCCATTGTTCAATGTATTCCAGCTTGGCTTCGCTTCATCCAGTGCCTGCGCCGGTACCGTGACACGAGGAGGGCTTTTCCTCATCTAGTAAATGCTGGCAAATACTCcACAACTTTCTTCACcGTGACATTTGCAGCCCTTTACAGCACTCACAAACCTCCTCTTTCTCCAGAACAAAAtCACTCTGACACCgtGGTgTTCTTTTACCTGTCTATTACTGCTACAACGTTTAAGCCTCATGTTGGGGAC

>Fra-AN0601-1

GAGAAGCTGGCAGAGGCTCAGCGCAGGTTTGCTACACTTCAGAATGAGCTTCAGTCATCGCTGGATGTGCAGAAAGAAAGCAGTGgTGTCACaaCAcTGCGACAgCGCAGAAAGCCAGTGTTCCACCTGTCCCACGAGGAGCGtGTCCAACATAGGAAcATTAAAGACCTTAAGCTGGCCTTCaGCGAGTTCTACCTCAGTCTCATCCTGCTGCAGAACGAACCAGAATTTTGCCACaAATATTCATATGGTGTGCGAGCCATTGTTCAATGTATTCCAGCTTGGCTTCGCTTCATCCAGTGCCTGCGCCGGTACCGTGACACGAGGAGGGCTTTTCCTCATCTAGTAAATGCTGGCAAATACTCcACAACTTTCTTCACcGTGACATTTGCAGCCCTTTACAGCACTCACAAACCTCCTCTTTCTCCAGAACAAAAtCACTCTGACACCgtGGTgTTCTTTTACCTGTCTATTACTGCTACAACGTTTAAGCCTCATGTTGGGGAC

>Fra-AN0601-2

GAGAAGCTGGCAGAGGCTCAGCGCAGGTTTGCTACACTTCAGAATGAGCTTCAGTCATCGCTGGATGTGCAGAAAGAAAGCAGTGgTGTCACaaCAcTGCGACAgCGCAGAAAGCCAGTGTTCCACCTGTCCCACGAGGAGCGtGTCCAACATAGGAAcATTAAAGACCTTAAGCTGGCCTTCaGCGAGTTCTACCTCAGTCTCATCCTGCTGCAGAACGAACCAGAATTTTGCCACaAATATTCATATGGTGTGCGAGCCATTGTTCAATGTATTCCAGCTTGGCTTCGCTTCATCCAGTGCCTGCGCCGGTACCGTGACACGAGGAGGGCTTTTCCTCATCTAGTAAATGCTGGCAAATACTCcACAACTTTCTTCACcGTGACATTTGCAGCCCTTTACAGCACTCACAAACCTCCTCTTTCTCCAGAACAAAAtCACTCTGACACCgtGGTgTTCTTTTACCTGTCTATTACTGCTACAACGTTTAAGCCTCATGTTGGGGAC

>Fra-AN2701-1

GAGAAGCTGGCAGAGGCTCAGCGCAGGTTTGCTACACTTCAGAATGAGCTTCAGTCATCGCTGGATGTGCAGAAAGAAAGCAGTGgTGTCACaaCAcTGCGACAgCGCAGAAAGCCAGTGTTCCACCTGTCCCACGAGGAGCGtGTCCAACATAGGAAcATTAAAGACCTTAAGCTGGCCTTCaGCGAGTTCTACCTCAGTCTCATCCTGCTGCAGAACGAACCAGAATTTTGCCACaAATATTCATATGGTGTGCGAGCCATTGTTCAATGTATTCCAGCTTGGCTTCGCTTCATCCAGTGCCTGCGCCGGTACCGTGACACGAGGAGGGCTTTTCCTCATCTAGTAAATGCTGGCAAATACTCcACAACTTTCTTCACcGTGACATTTGCAGCCCTTTACAGCACTCACAAACCTCCTCTTTCTCCAGAACAAAAtCACTCTGACACCgtGGTgTTCTTTTACCTGTCTATTACTGCTACAACGTTTAAGCCTCATGTTGGGGAC

>Fra-AN2701-2

GAGAAGCTGGCAGAGGCTCAGCGCAGGTTTGCTACACTTCAGAATGAGCTTCAGTCATCGCTGGATGTGCAGAAAGAAAGCAGTGgTGTCACaaCAcTGCGACAgCGCAGAAAGCCAGTGTTCCACCTGTCCCACGAGGAGCGtGTCCAACATAGGAAcATTAAAGACCTTAAGCTGGCCTTCaGCGAGTTCTACCTCAGTCTCATCCTGCTGCAGAACGAACCAGAATTTTGCCACaAATATTCATATGGTGTGCGAGCCATTGTTCAATGTATTCCAGCTTGGCTTCGCTTCATCCAGTGCCTGCGCCGGTACCGTGACACGAGGAGGGCTTTTCCTCATCTAGTAAATGCTGGCAAATACTCcACAACTTTCTTCACcGTGACATTTGCAGCCCTTTACAGCACTCACAAACCTCCTCTTTCTCCAGAACAAAAtCACTCTGACACCgtGGTgTTCTTTTACCTGTCTATTACTGCTACAACGTTTAAGCCTCATGTTGGGGAC

>Ger-CB17-1

GAGAAGCTGGCAGAGGCTCAGCGCAGGTTTGCTACACTTCAGAATGAGCTTCAGTCATCGCTGGATGTGCAGAAAGAAAGCAGTGcTGTCACagCAtTGCGACAgCGCAGAAAGCCAGTGTTCCACCTGTCCCACGAGGAGCGtGTCCAACATAGGAAcATTAAAGACCTTAAGCTGGCCTTCaGCGAGTTCTACCTCAGTCTCATCCTGCTGCAGAACGAACCAGAATTTTGCCACaAATATTCATATGGTGTGCGAGCCATTGTTCAATGTATTCCAGCTTGGCTTCGCTTCATCCAGTGCCTGCGCCGGTACCGTGACACGAGGAGGGCTTTTCCTCATCTAGTAAATGCTGGCAAATACTCcACAACTTTCTTCACcGTGACATTTGCAGCCCTTTACAGCACTCACAAACCTCCTCTTTCTCCAGAACAAAAtCACTCTGACACCgtGGTgTTCTTTTACCTGTCTATTACTGCTACAACGTTTAAGCCTCATGTTGGGGAC

>Ger-CB17-2

GAGAAGCTGGCAGAGGCTCAGCGCAGGTTTGCTACACTTCAGAATGAGCTTCAGTCATCGCTGGATGTGCAGAAAGAAAGCAGTGcTGTCACagCAtTGCGACAgCGCAGAAAGCCAGTGTTCCACCTGTCCCACGAGGAGCGtGTCCAACATAGGAAcATTAAAGACCTTAAGCTGGCCTTCaGCGAGTTCTACCTCAGTCTCATCCTGCTGCAGAACGAACCAGAATTTTGCCACaAATATTCATATGGTGTGCGAGCCATTGTTCAATGTATTCCAGCTTGGCTTCGCTTCATCCAGTGCCTGCGCCGGTACCGTGACACGAGGAGGGCTTTTCCTCATCTAGTAAATGCTGGCAAATACTCcACAACTTTCTTCACcGTGACATTTGCAGCCCTTTACAGCACTCACAAACCTCCTCTTTCTCCAGAACAAAAtCACTCTGACACCgtGGTgTTCTTTTACCTGTCTATTACTGCTACAACGTTTAAGCCTCATGTTGGGGAC

>Ger-CB15-1

GAGAAGCTGGCAGAGGCTCAGCGCAGGTTTGCTACACTTCAGAATGAGCTTCAGTCATCGCTGGATGTGCAGAAAGAAAGCAGTGcTGTCACagCAtTGCGACAgCGCAGAAAGCCAGTGTTCCACCTGTCCCACGAGGAGCGtGTCCAACATAGGAAcATTAAAGACCTTAAGCTGGCCTTCaGCGAGTTCTACCTCAGTCTCATCCTGCTGCAGAACGAACCAGAATTTTGCCACaAATATTCATATGGTGTGCGAGCCATTGTTCAATGTATTCCAGCTTGGCTTCGCTTCATCCAGTGCCTGCGCCGGTACCGTGACACGAGGAGGGCTTTTCCTCATCTAGTAAATGCTGGCAAATACTCcACAACTTTCTTCACcGTGACATTTGCAGCCCTTTACAGCACTCACAAACCTCCTCTTTCTCCAGAACAAAAtCACTCTGACACCgtGGTgTTCTTTTACCTGTCTATTACTGCTACAACGTTTAAGCCTCATGTTGGGGAC

>Ger-CB15-2

GAGAAGCTGGCAGAGGCTCAGCGCAGGTTTGCTACACTTCAGAATGAGCTTCAGTCATCGCTGGATGTGCAGAAAGAAAGCAGTGcTGTCACagCAtTGCGACAgCGCAGAAAGCCAGTGTTCCACCTGTCCCACGAGGAGCGtGTCCAACATAGGAAcATTAAAGACCTTAAGCTGGCCTTCaGCGAGTTCTACCTCAGTCTCATCCTGCTGCAGAACGAACCAGAATTTTGCCACaAATATTCATATGGTGTGCGAGCCATTGTTCAATGTATTCCAGCTTGGCTTCGCTTCATCCAGTGCCTGCGCCGGTACCGTGACACGAGGAGGGCTTTTCCTCATCTAGTAAATGCTGGCAAATACTCcACAACTTTCTTCACcGTGACATTTGCAGCCCTTTACAGCACTCACAAACCTCCTCTTTCTCCAGAACAAAAtCACTCTGACACCgtGGTgTTCTTTTACCTGTCTATTACTGCTACAACGTTTAAGCCTCATGTTGGGGAC

>Ger-CB18-1

GAGAAGCTGGCAGAGGCTCAGCGCAGGTTTGCTACACTTCAGAATGAGCTTCAGTCATCGCTGGATGTGCAGAAAGAAAGCAGTGcTGTCACagCAtTGCGACAgCGCAGAAAGCCAGTGTTCCACCTGTCCCACGAGGAGCGtGTCCAACATAGGAAcATTAAAGACCTTAAGCTGGCCTTCaGCGAGTTCTACCTCAGTCTCATCCTGCTGCAGAACGAACCAGAATTTTGCCACaAATATTCATATGGTGTGCGAGCCATTGTTCAATGTATTCCAGCTTGGCTTCGCTTCATCCAGTGCCTGCGCCGGTACCGTGACACGAGGAGGGCTTTTCCTCATCTAGTAAATGCTGGCAAATACTCcACAACTTTCTTCACcGTGACATTTGCAGCCCTTTACAGCACTCACAAACCTCCTCTTTCTCCAGAACAAAAtCACTCTGACACCgtGGTgTTCTTTTACCTGTCTATTACTGCTACAACGTTTAAGCCTCATGTTGGGGAC

>Ger-CB18-2

GAGAAGCTGGCAGAGGCTCAGCGCAGGTTTGCTACACTTCAGAATGAGCTTCAGTCATCGCTGGATGTGCAGAAAGAAAGCAGTGcTGTCACagCAtTGCGACAgCGCAGAAAGCCAGTGTTCCACCTGTCCCACGAGGAGCGtGTCCAACATAGGAAcATTAAAGACCTTAAGCTGGCCTTCaGCGAGTTCTACCTCAGTCTCATCCTGCTGCAGAACGAACCAGAATTTTGCCACaAATATTCATATGGTGTGCGAGCCATTGTTCAATGTATTCCAGCTTGGCTTCGCTTCATCCAGTGCCTGCGCCGGTACCGTGACACGAGGAGGGCTTTTCCTCATCTAGTAAATGCTGGCAAATACTCcACAACTTTCTTCACcGTGACATTTGCAGCCCTTTACAGCACTCACAAACCTCCTCTTTCTCCAGAACAAAAtCACTCTGACACCgtGGTgTTCTTTTACCTGTCTATTACTGCTACAACGTTTAAGCCTCATGTTGGGGAC

>Ger-CB20-1

GAGAAGCTGGCAGAGGCTCAGCGCAGGTTTGCTACACTTCAGAATGAGCTTCAGTCATCGCTGGATGTGCAGAAAGAAAGCAGTGcTGTCACagCAtTGCGACAgCGCAGAAAGCCAGTGTTCCACCTGTCCCACGAGGAGCGtGTCCAACATAGGAAcATTAAAGACCTTAAGCTGGCCTTCaGCGAGTTCTACCTCAGTCTCATCCTGCTGCAGAACGAACCAGAATTTTGCCACaAATATTCATATGGTGTGCGAGCCATTGTTCAATGTATTCCAGCTTGGCTTCGCTTCATCCAGTGCCTGCGCCGGTACCGTGACACGAGGAGGGCTTTTCCTCATCTAGTAAATGCTGGCAAATACTCcACAACTTTCTTCACcGTGACATTTGCAGCCCTTTACAGCACTCACAAACCTCCTCTTTCTCCAGAACAAAAtCACTCTGACACCgtGGTgTTCTTTTACCTGTCTATTACTGCTACAACGTTTAAGCCTCATGTTGGGGAC

>Ger-CB20-2

GAGAAGCTGGCAGAGGCTCAGCGCAGGTTTGCTACACTTCAGAATGAGCTTCAGTCATCGCTGGATGTGCAGAAAGAAAGCAGTGcTGTCACagCAtTGCGACAgCGCAGAAAGCCAGTGTTCCACCTGTCCCACGAGGAGCGtGTCCAACATAGGAAcATTAAAGACCTTAAGCTGGCCTTCaGCGAGTTCTACCTCAGTCTCATCCTGCTGCAGAACGAACCAGAATTTTGCCACaAATATTCATATGGTGTGCGAGCCATTGTTCAATGTATTCCAGCTTGGCTTCGCTTCATCCAGTGCCTGCGCCGGTACCGTGACACGAGGAGGGCTTTTCCTCATCTAGTAAATGCTGGCAAATACTCcACAACTTTCTTCACcGTGACATTTGCAGCCCTTTACAGCACTCACAAACCTCCTCTTTCTCCAGAACAAAAtCACTCTGACACCgtGGTgTTCTTTTACCTGTCTATTACTGCTACAACGTTTAAGCCTCATGTTGGGGAC

>Ger-CB01-1

GAGAAGCTGGCAGAGGCTCAGCGCAGGTTTGCTACACTTCAGAATGAGCTTCAGTCATCGCTGGATGTGCAGAAAGAAAGCAGTGcTGTCACagCAtTGCGACAgCGCAGAAAGCCAGTGTTCCACCTGTCCCACGAGGAGCGtGTCCAACATAGGAAcATTAAAGACCTTAAGCTGGCCTTCaGCGAGTTCTACCTCAGTCTCATCCTGCTGCAGAACGAACCAGAATTTTGCCACaAATATTCATATGGTGTGCGAGCCATTGTTCAATGTATTCCAGCTTGGCTTCGCTTCATCCAGTGCCTGCGCCGGTACCGTGACACGAGGAGGGCTTTTCCTCATCTAGTAAATGCTGGCAAATACTCcACAACTTTCTTCACcGTGACATTTGCAGCCCTTTACAGCACTCACAAACCTCCTCTTTCTCCAGAACAAAAtCACTCTGACACCgtGGTgTTCTTTTACCTGTCTATTACTGCTACAACGTTTAAGCCTCATGTTGGGGAC

>Ger-CB01-2

GAGAAGCTGGCAGAGGCTCAGCGCAGGTTTGCTACACTTCAGAATGAGCTTCAGTCATCGCTGGATGTGCAGAAAGAAAGCAGTGcTGTCACagCAtTGCGACAgCGCAGAAAGCCAGTGTTCCACCTGTCCCACGAGGAGCGtGTCCAACATAGGAAcATTAAAGACCTTAAGCTGGCCTTCaGCGAGTTCTACCTCAGTCTCATCCTGCTGCAGAACGAACCAGAATTTTGCCACaAATATTCATATGGTGTGCGAGCCATTGTTCAATGTATTCCAGCTTGGCTTCGCTTCATCCAGTGCCTGCGCCGGTACCGTGACACGAGGAGGGCTTTTCCTCATCTAGTAAATGCTGGCAAATACTCcACAACTTTCTTCACcGTGACATTTGCAGCCCTTTACAGCACTCACAAACCTCCTCTTTCTCCAGAACAAAAtCACTCTGACACCgtGGTgTTCTTTTACCTGTCTATTACTGCTACAACGTTTAAGCCTCATGTTGGGGAC

>Ger-CB09-1

GAGAAGCTGGCAGAGGCTCAGCGCAGGTTTGCTACACTTCAGAATGAGCTTCAGTCATCGCTGGATGTGCAGAAAGAAAGCAGTGcTGTCACagCAtTGCGACAgCGCAGAAAGCCAGTGTTCCACCTGTCCCACGAGGAGCGtGTCCAACATAGGAAcATTAAAGACCTTAAGCTGGCCTTCaGCGAGTTCTACCTCAGTCTCATCCTGCTGCAGAACGAACCAGAATTTTGCCACaAATATTCATATGGTGTGCGAGCCATTGTTCAATGTATTCCAGCTTGGCTTCGCTTCATCCAGTGCCTGCGCCGGTACCGTGACACGAGGAGGGCTTTTCCTCATCTAGTAAATGCTGGCAAATACTCcACAACTTTCTTCACcGTGACATTTGCAGCCCTTTACAGCACTCACAAACCTCCTCTTTCTCCAGAACAAAAtCACTCTGACACCgtGGTgTTCTTTTACCTGTCTATTACTGCTACAACGTTTAAGCCTCATGTTGGGGAC

>Ger-CB09-2

GAGAAGCTGGCAGAGGCTCAGCGCAGGTTTGCTACACTTCAGAATGAGCTTCAGTCATCGCTGGATGTGCAGAAAGAAAGCAGTGcTGTCACagCAtTGCGACAgCGCAGAAAGCCAGTGTTCCACCTGTCCCACGAGGAGCGtGTCCAACATAGGAAcATTAAAGACCTTAAGCTGGCCTTCaGCGAGTTCTACCTCAGTCTCATCCTGCTGCAGAACGAACCAGAATTTTGCCACaAATATTCATATGGTGTGCGAGCCATTGTTCAATGTATTCCAGCTTGGCTTCGCTTCATCCAGTGCCTGCGCCGGTACCGTGACACGAGGAGGGCTTTTCCTCATCTAGTAAATGCTGGCAAATACTCcACAACTTTCTTCACcGTGACATTTGCAGCCCTTTACAGCACTCACAAACCTCCTCTTTCTCCAGAACAAAAtCACTCTGACACCgtGGTgTTCTTTTACCTGTCTATTACTGCTACAACGTTTAAGCCTCATGTTGGGGAC

>Ger-CB05-1

GAGAAGCTGGCAGAGGCTCAGCGCAGGTTTGCTACACTTCAGAATGAGCTTCAGTCATCGCTGGATGTGCAGAAAGAAAGCAGTGcTGTCACagCAtTGCGACAgCGCAGAAAGCCAGTGTTCCACCTGTCCCACGAGGAGCGtGTCCAACATAGGAAcATTAAAGACCTTAAGCTGGCCTTCaGCGAGTTCTACCTCAGTCTCATCCTGCTGCAGAACGAACCAGAATTTTGCCACaAATATTCATATGGTGTGCGAGCCATTGTTCAATGTATTCCAGCTTGGCTTCGCTTCATCCAGTGCCTGCGCCGGTACCGTGACACGAGGAGGGCTTTTCCTCATCTAGTAAATGCTGGCAAATACTCcACAACTTTCTTCACcGTGACATTTGCAGCCCTTTACAGCACTCACAAACCTCCTCTTTCTCCAGAACAAAAtCACTCTGACACCgtGGTgTTCTTTTACCTGTCTATTACTGCTACAACGTTTAAGCCTCATGTTGGGGAC

>Ger-CB05-2

GAGAAGCTGGCAGAGGCTCAGCGCAGGTTTGCTACACTTCAGAATGAGCTTCAGTCATCGCTGGATGTGCAGAAAGAAAGCAGTGcTGTCACagCAtTGCGACAgCGCAGAAAGCCAGTGTTCCACCTGTCCCACGAGGAGCGtGTCCAACATAGGAAcATTAAAGACCTTAAGCTGGCCTTCaGCGAGTTCTACCTCAGTCTCATCCTGCTGCAGAACGAACCAGAATTTTGCCACaAATATTCATATGGTGTGCGAGCCATTGTTCAATGTATTCCAGCTTGGCTTCGCTTCATCCAGTGCCTGCGCCGGTACCGTGACACGAGGAGGGCTTTTCCTCATCTAGTAAATGCTGGCAAATACTCcACAACTTTCTTCACcGTGACATTTGCAGCCCTTTACAGCACTCACAAACCTCCTCTTTCTCCAGAACAAAAtCACTCTGACACCgtGGTgTTCTTTTACCTGTCTATTACTGCTACAACGTTTAAGCCTCATGTTGGGGAC

>Ger-CB13-1

GAGAAGCTGGCAGAGGCTCAGCGCAGGTTTGCTACACTTCAGAATGAGCTTCAGTCATCGCTGGATGTGCAGAAAGAAAGCAGTGcTGTCACagCAtTGCGACAgCGCAGAAAGCCAGTGTTCCACCTGTCCCACGAGGAGCGtGTCCAACATAGGAAcATTAAAGACCTTAAGCTGGCCTTCaGCGAGTTCTACCTCAGTCTCATCCTGCTGCAGAACGAACCAGAATTTTGCCACaAATATTCATATGGTGTGCGAGCCATTGTTCAATGTATTCCAGCTTGGCTTCGCTTCATCCAGTGCCTGCGCCGGTACCGTGACACGAGGAGGGCTTTTCCTCATCTAGTAAATGCTGGCAAATACTCcACAACTTTCTTCACcGTGACATTTGCAGCCCTTTACAGCACTCACAAACCTCCTCTTTCTCCAGAACAAAAtCACTCTGACACCgtGGTgTTCTTTTACCTGTCTATTACTGCTACAACGTTTAAGCCTCATGTTGGGGAC

>Ger-CB13-2

GAGAAGCTGGCAGAGGCTCAGCGCAGGTTTGCTACACTTCAGAATGAGCTTCAGTCATCGCTGGATGTGCAGAAAGAAAGCAGTGcTGTCACagCAtTGCGACAgCGCAGAAAGCCAGTGTTCCACCTGTCCCACGAGGAGCGtGTCCAACATAGGAAcATTAAAGACCTTAAGCTGGCCTTCaGCGAGTTCTACCTCAGTCTCATCCTGCTGCAGAACGAACCAGAATTTTGCCACaAATATTCATATGGTGTGCGAGCCATTGTTCAATGTATTCCAGCTTGGCTTCGCTTCATCCAGTGCCTGCGCCGGTACCGTGACACGAGGAGGGCTTTTCCTCATCTAGTAAATGCTGGCAAATACTCcACAACTTTCTTCACcGTGACATTTGCAGCCCTTTACAGCACTCACAAACCTCCTCTTTCTCCAGAACAAAAtCACTCTGACACCgtGGTgTTCTTTTACCTGTCTATTACTGCTACAACGTTTAAGCCTCATGTTGGGGAC

>Ger-CB03-1

GAGAAGCTGGCAGAGGCTCAGCGCAGGTTTGCTACACTTCAGAATGAGCTTCAGTCATCGCTGGATGTGCAGAAAGAAAGCAGTGcTGTCACagCAtTGCGACAgCGCAGAAAGCCAGTGTTCCACCTGTCCCACGAGGAGCGtGTCCAACATAGGAAcATTAAAGACCTTAAGCTGGCCTTCaGCGAGTTCTACCTCAGTCTCATCCTGCTGCAGAACGAACCAGAATTTTGCCACaAATATTCATATGGTGTGCGAGCCATTGTTCAATGTATTCCAGCTTGGCTTCGCTTCATCCAGTGCCTGCGCCGGTACCGTGACACGAGGAGGGCTTTTCCTCATCTAGTAAATGCTGGCAAATACTCcACAACTTTCTTCACcGTGACATTTGCAGCCCTTTACAGCACTCACAAACCTCCTCTTTCTCCAGAACAAAAtCACTCTGACACCgtGGTgTTCTTTTACCTGTCTATTACTGCTACAACGTTTAAGCCTCATGTTGGGGAC

>Ger-CB03-2

GAGAAGCTGGCAGAGGCTCAGCGCAGGTTTGCTACACTTCAGAATGAGCTTCAGTCATCGCTGGATGTGCAGAAAGAAAGCAGTGcTGTCACagCAtTGCGACAgCGCAGAAAGCCAGTGTTCCACCTGTCCCACGAGGAGCGtGTCCAACATAGGAAcATTAAAGACCTTAAGCTGGCCTTCaGCGAGTTCTACCTCAGTCTCATCCTGCTGCAGAACGAACCAGAATTTTGCCACaAATATTCATATGGTGTGCGAGCCATTGTTCAATGTATTCCAGCTTGGCTTCGCTTCATCCAGTGCCTGCGCCGGTACCGTGACACGAGGAGGGCTTTTCCTCATCTAGTAAATGCTGGCAAATACTCcACAACTTTCTTCACcGTGACATTTGCAGCCCTTTACAGCACTCACAAACCTCCTCTTTCTCCAGAACAAAAtCACTCTGACACCgtGGTgTTCTTTTACCTGTCTATTACTGCTACAACGTTTAAGCCTCATGTTGGGGAC

>Ger-CB21-1

GAGAAGCTGGCAGAGGCTCAGCGCAGGTTTGCTACACTTCAGAATGAGCTTCAGTCATCGCTGGATGTGCAGAAAGAAAGCAGTGcTGTCACagCAtTGCGACAgCGCAGAAAGCCAGTGTTCCACCTGTCCCACGAGGAGCGtGTCCAACATAGGAAcATTAAAGACCTTAAGCTGGCCTTCaGCGAGTTCTACCTCAGTCTCATCCTGCTGCAGAACGAACCAGAATTTTGCCACaAATATTCATATGGTGTGCGAGCCATTGTTCAATGTATTCCAGCTTGGCTTCGCTTCATCCAGTGCCTGCGCCGGTACCGTGACACGAGGAGGGCTTTTCCTCATCTAGTAAATGCTGGCAAATACTCcACAACTTTCTTCACcGTGACATTTGCAGCCCTTTACAGCACTCACAAACCTCCTCTTTCTCCAGAACAAAAtCACTCTGACACCgtGGTgTTCTTTTACCTGTCTATTACTGCTACAACGTTTAAGCCTCATGTTGGGGAC

>Ger-CB21-2

GAGAAGCTGGCAGAGGCTCAGCGCAGGTTTGCTACACTTCAGAATGAGCTTCAGTCATCGCTGGATGTGCAGAAAGAAAGCAGTGcTGTCACagCAtTGCGACAgCGCAGAAAGCCAGTGTTCCACCTGTCCCACGAGGAGCGtGTCCAACATAGGAAcATTAAAGACCTTAAGCTGGCCTTCaGCGAGTTCTACCTCAGTCTCATCCTGCTGCAGAACGAACCAGAATTTTGCCACaAATATTCATATGGTGTGCGAGCCATTGTTCAATGTATTCCAGCTTGGCTTCGCTTCATCCAGTGCCTGCGCCGGTACCGTGACACGAGGAGGGCTTTTCCTCATCTAGTAAATGCTGGCAAATACTCcACAACTTTCTTCACcGTGACATTTGCAGCCCTTTACAGCACTCACAAACCTCCTCTTTCTCCAGAACAAAAtCACTCTGACACCgtGGTgTTCTTTTACCTGTCTATTACTGCTACAACGTTTAAGCCTCATGTTGGGGAC

>Ger-CB10-1

GAGAAGCTGGCAGAGGCTCAGCGCAGGTTTGCTACACTTCAGAATGAGCTTCAGTCATCGCTGGATGTGCAGAAAGAAAGCAGTGgTGTCACaaCAcTGCGACAgCGCAGAAAGCCAGTGTTCCACCTGTCCCACGAGGAGCGtGTCCAACATAGGAAcATTAAAGACCTTAAGCTGGCCTTCaGCGAGTTCTACCTCAGTCTCATCCTGCTGCAGAACGAACCAGAATTTTGCCACaAATATTCATATGGTGTGCGAGCCATTGTTCAATGTATTCCAGCTTGGCTTCGCTTCATCCAGTGCCTGCGCCGGTACCGTGACACGAGGAGGGCTTTTCCTCATCTAGTAAATGCTGGCAAATACTCcACAACTTTCTTCACcGTGACATTTGCAGCCCTTTACAGCACTCACAAACCTCCTCTTTCTCCAGAACAAAAtCACTCTGACACCgtGGTgTTCTTTTACCTGTCTATTACTGCTACAACGTTTAAGCCTCATGTTGGGGAC

>Ger-CB10-2

GAGAAGCTGGCAGAGGCTCAGCGCAGGTTTGCTACACTTCAGAATGAGCTTCAGTCATCGCTGGATGTGCAGAAAGAAAGCAGTGcTGTCACagCAtTGCGACAgCGCAGAAAGCCAGTGTTCCACCTGTCCCACGAGGAGCGtGTCCAACATAGGAAcATTAAAGACCTTAAGCTGGCCTTCaGCGAGTTCTACCTCAGTCTCATCCTGCTGCAGAACGAACCAGAATTTTGCCACaAATATTCATATGGTGTGCGAGCCATTGTTCAATGTATTCCAGCTTGGCTTCGCTTCATCCAGTGCCTGCGCCGGTACCGTGACACGAGGAGGGCTTTTCCTCATCTAGTAAATGCTGGCAAATACTCcACAACTTTCTTCACcGTGACATTTGCAGCCCTTTACAGCACTCACAAACCTCCTCTTTCTCCAGAACAAAAtCACTCTGACACCgtGGTgTTCTTTTACCTGTCTATTACTGCTACAACGTTTAAGCCTCATGTTGGGGAC

>Fra-DB221-1

GAGAAGCTGGCAGAGGCTCAGCGCAGGTTTGCTACACTTCAGAATGAGCTTCAGTCATCGCTGGATGTGCAGAAAGAAAGCAGTGcTGTCACagCAtTGCGACAgCGCAGAAAGCCAGTGTTCCACCTGTCCCACGAGGAGCGtGTCCAACATAGGAAcATTAAAGACCTTAAGCTGGCCTTCaGCGAGTTCTACCTCAGTCTCATCCTGCTGCAGAACGAACCAGAATTTTGCCACaAATATTCATATGGTGTGCGAGCCATTGTTCAATGTATTCCAGCTTGGCTTCGCTTCATCCAGTGCCTGCGCCGGTACCGTGACACGAGGAGGGCTTTTCCTCATCTAGTAAATGCTGGCAAATACTCcACAACTTTCTTCACcGTGACATTTGCAGCCCTTTACAGCACTCACAAACCTCCTCTTTCTCCAGAACAAAAtCACTCTGACACCgtGGTgTTCTTTTACCTGTCTATTACTGCTACAACGTTTAAGCCTCATGTTGGGGAC

>Fra-DB221-2

GAGAAGCTGGCAGAGGCTCAGCGCAGGTTTGCTACACTTCAGAATGAGCTTCAGTCATCGCTGGATGTGCAGAAAGAAAGCAGTGcTGTCACagCAtTGCGACAgCGCAGAAAGCCAGTGTTCCACCTGTCCCACGAGGAGCGtGTCCAACATAGGAAcATTAAAGACCTTAAGCTGGCCTTCaGCGAGTTCTACCTCAGTCTCATCCTGCTGCAGAACGAACCAGAATTTTGCCACaAATATTCATATGGTGTGCGAGCCATTGTTCAATGTATTCCAGCTTGGCTTCGCTTCATCCAGTGCCTGCGCCGGTACCGTGACACGAGGAGGGCTTTTCCTCATCTAGTAAATGCTGGCAAATACTCcACAACTTTCTTCACcGTGACATTTGCAGCCCTTTACAGCACTCACAAACCTCCTCTTTCTCCAGAACAAAAtCACTCTGACACCgtGGTgTTCTTTTACCTGTCTATTACTGCTACAACGTTTAAGCCTCATGTTGGGGAC

>Fra-DB7-1

GAGAAGCTGGCAGAGGCTCAGCGCAGGTTTGCTACACTTCAGAATGAGCTTCAGTCATCGCTGGATGTGCAGAAAGAAAGCAGTGcTGTCACagCAtTGCGACAgCGCAGAAAGCCAGTGTTCCACCTGTCCCACGAGGAGCGtGTCCAACATAGGAAcATTAAAGACCTTAAGCTGGCCTTCaGCGAGTTCTACCTCAGTCTCATCCTGCTGCAGAACGAACCAGAATTTTGCCACaAATATTCATATGGTGTGCGAGCCATTGTTCAATGTATTCCAGCTTGGCTTCGCTTCATCCAGTGCCTGCGCCGGTACCGTGACACGAGGAGGGCTTTTCCTCATCTAGTAAATGCTGGCAAATACTCcACAACTTTCTTCACcGTGACATTTGCAGCCCTTTACAGCACTCACAAACCTCCTCTTTCTCCAGAACAAAAtCACTCTGACACCgtGGTgTTCTTTTACCTGTCTATTACTGCTACAACGTTTAAGCCTCATGTTGGGGAC

>Fra-DB7-2

GAGAAGCTGGCAGAGGCTCAGCGCAGGTTTGCTACACTTCAGAATGAGCTTCAGTCATCGCTGGATGTGCAGAAAGAAAGCAGTGcTGTCACagCAtTGCGACAgCGCAGAAAGCCAGTGTTCCACCTGTCCCACGAGGAGCGtGTCCAACATAGGAAcATTAAAGACCTTAAGCTGGCCTTCaGCGAGTTCTACCTCAGTCTCATCCTGCTGCAGAACGAACCAGAATTTTGCCACaAATATTCATATGGTGTGCGAGCCATTGTTCAATGTATTCCAGCTTGGCTTCGCTTCATCCAGTGCCTGCGCCGGTACCGTGACACGAGGAGGGCTTTTCCTCATCTAGTAAATGCTGGCAAATACTCcACAACTTTCTTCACcGTGACATTTGCAGCCCTTTACAGCACTCACAAACCTCCTCTTTCTCCAGAACAAAAtCACTCTGACACCgtGGTgTTCTTTTACCTGTCTATTACTGCTACAACGTTTAAGCCTCATGTTGGGGAC

>Fra-DB81-1

GAGAAGCTGGCAGAGGCTCAGCGCAGGTTTGCTACACTTCAGAATGAGCTTCAGTCATCGCTGGATGTGCAGAAAGAAAGCAGTGcTGTCACagCAtTGCGACAgCGCAGAAAGCCAGTGTTCCACCTGTCCCACGAGGAGCGtGTCCAACATAGGAAcATTAAAGACCTTAAGCTGGCCTTCaGCGAGTTCTACCTCAGTCTCATCCTGCTGCAGAACGAACCAGAATTTTGCCACaAATATTCATATGGTGTGCGAGCCATTGTTCAATGTATTCCAGCTTGGCTTCGCTTCATCCAGTGCCTGCGCCGGTACCGTGACACGAGGAGGGCTTTTCCTCATCTAGTAAATGCTGGCAAATACTCcACAACTTTCTTCACcGTGACATTTGCAGCCCTTTACAGCACTCACAAACCTCCTCTTTCTCCAGAACAAAAtCACTCTGACACCgtGGTgTTCTTTTACCTGTCTATTACTGCTACAACGTTTAAGCCTCATGTTGGGGAC

>Fra-DB81-2

GAGAAGCTGGCAGAGGCTCAGCGCAGGTTTGCTACACTTCAGAATGAGCTTCAGTCATCGCTGGATGTGCAGAAAGAAAGCAGTGcTGTCACagCAtTGCGACAgCGCAGAAAGCCAGTGTTCCACCTGTCCCACGAGGAGCGtGTCCAACATAGGAAcATTAAAGACCTTAAGCTGGCCTTCaGCGAGTTCTACCTCAGTCTCATCCTGCTGCAGAACGAACCAGAATTTTGCCACaAATATTCATATGGTGTGCGAGCCATTGTTCAATGTATTCCAGCTTGGCTTCGCTTCATCCAGTGCCTGCGCCGGTACCGTGACACGAGGAGGGCTTTTCCTCATCTAGTAAATGCTGGCAAATACTCcACAACTTTCTTCACcGTGACATTTGCAGCCCTTTACAGCACTCACAAACCTCCTCTTTCTCCAGAACAAAAtCACTCTGACACCgtGGTgTTCTTTTACCTGTCTATTACTGCTACAACGTTTAAGCCTCATGTTGGGGAC

>Fra-DB151-1

GAGAAGCTGGCAGAGGCTCAGCGCAGGTTTGCTACACTTCAGAATGAGCTTCAGTCATCGCTGGATGTGCAGAAAGAAAGCAGTGcTGTCACagCAtTGCGACAgCGCAGAAAGCCAGTGTTCCACCTGTCCCACGAGGAGCGtGTCCAACATAGGAAcATTAAAGACCTTAAGCTGGCCTTCaGCGAGTTCTACCTCAGTCTCATCCTGCTGCAGAACGAACCAGAATTTTGCCACaAATATTCATATGGTGTGCGAGCCATTGTTCAATGTATTCCAGCTTGGCTTCGCTTCATCCAGTGCCTGCGCCGGTACCGTGACACGAGGAGGGCTTTTCCTCATCTAGTAAATGCTGGCAAATACTCcACAACTTTCTTCACcGTGACATTTGCAGCCCTTTACAGCACTCACAAACCTCCTCTTTCTCCAGAACAAAAtCACTCTGACACCgtGGTgTTCTTTTACCTGTCTATTACTGCTACAACGTTTAAGCCTCATGTTGGGGAC

>Fra-DB151-2

GAGAAGCTGGCAGAGGCTCAGCGCAGGTTTGCTACACTTCAGAATGAGCTTCAGTCATCGCTGGATGTGCAGAAAGAAAGCAGTGcTGTCACagCAtTGCGACAgCGCAGAAAGCCAGTGTTCCACCTGTCCCACGAGGAGCGtGTCCAACATAGGAAcATTAAAGACCTTAAGCTGGCCTTCaGCGAGTTCTACCTCAGTCTCATCCTGCTGCAGAACGAACCAGAATTTTGCCACaAATATTCATATGGTGTGCGAGCCATTGTTCAATGTATTCCAGCTTGGCTTCGCTTCATCCAGTGCCTGCGCCGGTACCGTGACACGAGGAGGGCTTTTCCTCATCTAGTAAATGCTGGCAAATACTCcACAACTTTCTTCACcGTGACATTTGCAGCCCTTTACAGCACTCACAAACCTCCTCTTTCTCCAGAACAAAAtCACTCTGACACCgtGGTgTTCTTTTACCTGTCTATTACTGCTACAACGTTTAAGCCTCATGTTGGGGAC

>Fra-DB11-1

GAGAAGCTGGCAGAGGCTCAGCGCAGGTTTGCTACACTTCAGAATGAGCTTCAGTCATCGCTGGATGTGCAGAAAGAAAGCAGTGcTGTCACagCAtTGCGACAgCGCAGAAAGCCAGTGTTCCACCTGTCCCACGAGGAGCGtGTCCAACATAGGAAcATTAAAGACCTTAAGCTGGCCTTCaGCGAGTTCTACCTCAGTCTCATCCTGCTGCAGAACGAACCAGAATTTTGCCACaAATATTCATATGGTGTGCGAGCCATTGTTCAATGTATTCCAGCTTGGCTTCGCTTCATCCAGTGCCTGCGCCGGTACCGTGACACGAGGAGGGCTTTTCCTCATCTAGTAAATGCTGGCAAATACTCcACAACTTTCTTCACcGTGACATTTGCAGCCCTTTACAGCACTCACAAACCTCCTCTTTCTCCAGAACAAAAtCACTCTGACACCgtGGTgTTCTTTTACCTGTCTATTACTGCTACAACGTTTAAGCCTCATGTTGGGGAC

>Fra-DB11-2

GAGAAGCTGGCAGAGGCTCAGCGCAGGTTTGCTACACTTCAGAATGAGCTTCAGTCATCGCTGGATGTGCAGAAAGAAAGCAGTGcTGTCACagCAtTGCGACAgCGCAGAAAGCCAGTGTTCCACCTGTCCCACGAGGAGCGtGTCCAACATAGGAAcATTAAAGACCTTAAGCTGGCCTTCaGCGAGTTCTACCTCAGTCTCATCCTGCTGCAGAACGAACCAGAATTTTGCCACaAATATTCATATGGTGTGCGAGCCATTGTTCAATGTATTCCAGCTTGGCTTCGCTTCATCCAGTGCCTGCGCCGGTACCGTGACACGAGGAGGGCTTTTCCTCATCTAGTAAATGCTGGCAAATACTCcACAACTTTCTTCACcGTGACATTTGCAGCCCTTTACAGCACTCACAAACCTCCTCTTTCTCCAGAACAAAAtCACTCTGACACCgtGGTgTTCTTTTACCTGTCTATTACTGCTACAACGTTTAAGCCTCATGTTGGGGAC

>Fra-DB3-1

GAGAAGCTGGCAGAGGCTCAGCGCAGGTTTGCTACACTTCAGAATGAGCTTCAGTCATCGCTGGATGTGCAGAAAGAAAGCAGTGcTGTCACagCAtTGCGACAgCGCAGAAAGCCAGTGTTCCACCTGTCCCACGAGGAGCGtGTCCAACATAGGAAcATTAAAGACCTTAAGCTGGCCTTCaGCGAGTTCTACCTCAGTCTCATCCTGCTGCAGAACGAACCAGAATTTTGCCACaAATATTCATATGGTGTGCGAGCCATTGTTCAATGTATTCCAGCTTGGCTTCGCTTCATCCAGTGCCTGCGCCGGTACCGTGACACGAGGAGGGCTTTTCCTCATCTAGTAAATGCTGGCAAATACTCcACAACTTTCTTCACcGTGACATTTGCAGCCCTTTACAGCACTCACAAACCTCCTCTTTCTCCAGAACAAAAtCACTCTGACACCgtGGTgTTCTTTTACCTGTCTATTACTGCTACAACGTTTAAGCCTCATGTTGGGGAC

>Fra-DB3-2

GAGAAGCTGGCAGAGGCTCAGCGCAGGTTTGCTACACTTCAGAATGAGCTTCAGTCATCGCTGGATGTGCAGAAAGAAAGCAGTGcTGTCACagCAtTGCGACAgCGCAGAAAGCCAGTGTTCCACCTGTCCCACGAGGAGCGtGTCCAACATAGGAAcATTAAAGACCTTAAGCTGGCCTTCaGCGAGTTCTACCTCAGTCTCATCCTGCTGCAGAACGAACCAGAATTTTGCCACaAATATTCATATGGTGTGCGAGCCATTGTTCAATGTATTCCAGCTTGGCTTCGCTTCATCCAGTGCCTGCGCCGGTACCGTGACACGAGGAGGGCTTTTCCTCATCTAGTAAATGCTGGCAAATACTCcACAACTTTCTTCACcGTGACATTTGCAGCCCTTTACAGCACTCACAAACCTCCTCTTTCTCCAGAACAAAAtCACTCTGACACCgtGGTgTTCTTTTACCTGTCTATTACTGCTACAACGTTTAAGCCTCATGTTGGGGAC

>Fra-DB201-1

GAGAAGCTGGCAGAGGCTCAGCGCAGGTTTGCTACACTTCAGAATGAGCTTCAGTCATCGCTGGATGTGCAGAAAGAAAGCAGTGcTGTCACagCAtTGCGACAgCGCAGAAAGCCAGTGTTCCACCTGTCCCACGAGGAGCGtGTCCAACATAGGAAcATTAAAGACCTTAAGCTGGCCTTCaGCGAGTTCTACCTCAGTCTCATCCTGCTGCAGAACGAACCAGAATTTTGCCACaAATATTCATATGGTGTGCGAGCCATTGTTCAATGTATTCCAGCTTGGCTTCGCTTCATCCAGTGCCTGCGCCGGTACCGTGACACGAGGAGGGCTTTTCCTCATCTAGTAAATGCTGGCAAATACTCcACAACTTTCTTCACcGTGACATTTGCAGCCCTTTACAGCACTCACAAACCTCCTCTTTCTCCAGAACAAAAtCACTCTGACACCgtGGTgTTCTTTTACCTGTCTATTACTGCTACAACGTTTAAGCCTCATGTTGGGGAC

>Fra-DB201-2

GAGAAGCTGGCAGAGGCTCAGCGCAGGTTTGCTACACTTCAGAATGAGCTTCAGTCATCGCTGGATGTGCAGAAAGAAAGCAGTGcTGTCACagCAtTGCGACAgCGCAGAAAGCCAGTGTTCCACCTGTCCCACGAGGAGCGtGTCCAACATAGGAAcATTAAAGACCTTAAGCTGGCCTTCaGCGAGTTCTACCTCAGTCTCATCCTGCTGCAGAACGAACCAGAATTTTGCCACaAATATTCATATGGTGTGCGAGCCATTGTTCAATGTATTCCAGCTTGGCTTCGCTTCATCCAGTGCCTGCGCCGGTACCGTGACACGAGGAGGGCTTTTCCTCATCTAGTAAATGCTGGCAAATACTCcACAACTTTCTTCACcGTGACATTTGCAGCCCTTTACAGCACTCACAAACCTCCTCTTTCTCCAGAACAAAAtCACTCTGACACCgtGGTgTTCTTTTACCTGTCTATTACTGCTACAACGTTTAAGCCTCATGTTGGGGAC

>Fra-DB191-1

GAGAAGCTGGCAGAGGCTCAGCGCAGGTTTGCTACACTTCAGAATGAGCTTCAGTCATCGCTGGATGTGCAGAAAGAAAGCAGTGcTGTCACagCAtTGCGACAgCGCAGAAAGCCAGTGTTCCACCTGTCCCACGAGGAGCGtGTCCAACATAGGAAcATTAAAGACCTTAAGCTGGCCTTCaGCGAGTTCTACCTCAGTCTCATCCTGCTGCAGAACGAACCAGAATTTTGCCACaAATATTCATATGGTGTGCGAGCCATTGTTCAATGTATTCCAGCTTGGCTTCGCTTCATCCAGTGCCTGCGCCGGTACCGTGACACGAGGAGGGCTTTTCCTCATCTAGTAAATGCTGGCAAATACTCcACAACTTTCTTCACcGTGACATTTGCAGCCCTTTACAGCACTCACAAACCTCCTCTTTCTCCAGAACAAAAtCACTCTGACACCgtGGTgTTCTTTTACCTGTCTATTACTGCTACAACGTTTAAGCCTCATGTTGGGGAC

>Fra-DB191-2

GAGAAGCTGGCAGAGGCTCAGCGCAGGTTTGCTACACTTCAGAATGAGCTTCAGTCATCGCTGGATGTGCAGAAAGAAAGCAGTGgTGTCACaaCAcTGCGACAgCGCAGAAAGCCAGTGTTCCACCTGTCCCACGAGGAGCGtGTCCAACATAGGAAcATTAAAGACCTTAAGCTGGCCTTCaGCGAGTTCTACCTCAGTCTCATCCTGCTGCAGAACGAACCAGAATTTTGCCACaAATATTCATATGGTGTGCGAGCCATTGTTCAATGTATTCCAGCTTGGCTTCGCTTCATCCAGTGCCTGCGCCGGTACCGTGACACGAGGAGGGCTTTTCCTCATCTAGTAAATGCTGGCAAATACTCcACAACTTTCTTCACcGTGACATTTGCAGCCCTTTACAGCACTCACAAACCTCCTCTTTCTCCAGAACAAAAtCACTCTGACACCgtGGTgTTCTTTTACCTGTCTATTACTGCTACAACGTTTAAGCCTCATGTTGGGGAC

>Fra-DB22-1

GAGAAGCTGGCAGAGGCTCAGCGCAGGTTTGCTACACTTCAGAATGAGCTTCAGTCATCGCTGGATGTGCAGAAAGAAAGCAGTGcTGTCACagCAtTGCGACAgCGCAGAAAGCCAGTGTTCCACCTGTCCCACGAGGAGCGtGTCCAACATAGGAAcATTAAAGACCTTAAGCTGGCCTTCaGCGAGTTCTACCTCAGTCTCATCCTGCTGCAGAACGAACCAGAATTTTGCCACaAATATTCATATGGTGTGCGAGCCATTGTTCAATGTATTCCAGCTTGGCTTCGCTTCATCCAGTGCCTGCGCCGGTACCGTGACACGAGGAGGGCTTTTCCTCATCTAGTAAATGCTGGCAAATACTCcACAACTTTCTTCACcGTGACATTTGCAGCCCTTTACAGCACTCACAAACCTCCTCTTTCTCCAGAACAAAAtCACTCTGACACCgtGGTgTTCTTTTACCTGTCTATTACTGCTACAACGTTTAAGCCTCATGTTGGGGAC

>Fra-DB22-2

GAGAAGCTGGCAGAGGCTCAGCGCAGGTTTGCTACACTTCAGAATGAGCTTCAGTCATCGCTGGATGTGCAGAAAGAAAGCAGTGgTGTCACaaCAcTGCGACAgCGCAGAAAGCCAGTGTTCCACCTGTCCCACGAGGAGCGtGTCCAACATAGGAAcATTAAAGACCTTAAGCTGGCCTTCaGCGAGTTCTACCTCAGTCTCATCCTGCTGCAGAACGAACCAGAATTTTGCCACaAATATTCATATGGTGTGCGAGCCATTGTTCAATGTATTCCAGCTTGGCTTCGCTTCATCCAGTGCCTGCGCCGGTACCGTGACACGAGGAGGGCTTTTCCTCATCTAGTAAATGCTGGCAAATACTCcACAACTTTCTTCACcGTGACATTTGCAGCCCTTTACAGCACTCACAAACCTCCTCTTTCTCCAGAACAAAAtCACTCTGACACCgtGGTgTTCTTTTACCTGTCTATTACTGCTACAACGTTTAAGCCTCATGTTGGGGAC

>Fra-DB131-1

GAGAAGCTGGCAGAGGCTCAGCGCAGGTTTGCTACACTTCAGAATGAGCTTCAGTCATCGCTGGATGTGCAGAAAGAAAGCAGTGcTGTCACagCAtTGCGACAgCGCAGAAAGCCAGTGTTCCACCTGTCCCACGAGGAGCGtGTCCAACATAGGAAcATTAAAGACCTTAAGCTGGCCTTCaGCGAGTTCTACCTCAGTCTCATCCTGCTGCAGAACGAACCAGAATTTTGCCACaAATATTCATATGGTGTGCGAGCCATTGTTCAATGTATTCCAGCTTGGCTTCGCTTCATCCAGTGCCTGCGCCGGTACCGTGACACGAGGAGGGCTTTTCCTCATCTAGTAAATGCTGGCAAATACTCcACAACTTTCTTCACcGTGACATTTGCAGCCCTTTACAGCACTCACAAACCTCCTCTTTCTCCAGAACAAAAtCACTCTGACACCgtGGTgTTCTTTTACCTGTCTATTACTGCTACAACGTTTAAGCCTCATGTTGGGGAC

>Fra-DB131-2

GAGAAGCTGGCAGAGGCTCAGCGCAGGTTTGCTACACTTCAGAATGAGCTTCAGTCATCGCTGGATGTGCAGAAAGAAAGCAGTGgTGTCACaaCAcTGCGACAgCGCAGAAAGCCAGTGTTCCACCTGTCCCACGAGGAGCGtGTCCAACATAGGAAcATTAAAGACCTTAAGCTGGCCTTCaGCGAGTTCTACCTCAGTCTCATCCTGCTGCAGAACGAACCAGAATTTTGCCACaAATATTCATATGGTGTGCGAGCCATTGTTCAATGTATTCCAGCTTGGCTTCGCTTCATCCAGTGCCTGCGCCGGTACCGTGACACGAGGAGGGCTTTTCCTCATCTAGTAAATGCTGGCAAATACTCcACAACTTTCTTCACcGTGACATTTGCAGCCCTTTACAGCACTCACAAACCTCCTCTTTCTCCAGAACAAAAtCACTCTGACACCgtGGTgTTCTTTTACCTGTCTATTACTGCTACAACGTTTAAGCCTCATGTTGGGGAC

>Fra-DB14-1

GAGAAGCTGGCAGAGGCTCAGCGCAGGTTTGCTACACTTCAGAATGAGCTTCAGTCATCGCTGGATGTGCAGAAAGAAAGCAGTGcTGTCACagCAtTGCGACAgCGCAGAAAGCCAGTGTTCCACCTGTCCCACGAGGAGCGtGTCCAACATAGGAAcATTAAAGACCTTAAGCTGGCCTTCaGCGAGTTCTACCTCAGTCTCATCCTGCTGCAGAACGAACCAGAATTTTGCCACaAATATTCATATGGTGTGCGAGCCATTGTTCAATGTATTCCAGCTTGGCTTCGCTTCATCCAGTGCCTGCGCCGGTACCGTGACACGAGGAGGGCTTTTCCTCATCTAGTAAATGCTGGCAAATACTCcACAACTTTCTTCACcGTGACATTTGCAGCCCTTTACAGCACTCACAAACCTCCTCTTTCTCCAGAACAAAAtCACTCTGACACCgtGGTgTTCTTTTACCTGTCTATTACTGCTACAACGTTTAAGCCTCATGTTGGGGAC

>Fra-DB14-2

GAGAAGCTGGCAGAGGCTCAGCGCAGGTTTGCTACACTTCAGAATGAGCTTCAGTCATCGCTGGATGTGCAGAAAGAAAGCAGTGgTGTCACaaCAcTGCGACAgCGCAGAAAGCCAGTGTTCCACCTGTCCCACGAGGAGCGtGTCCAACATAGGAAcATTAAAGACCTTAAGCTGGCCTTCaGCGAGTTCTACCTCAGTCTCATCCTGCTGCAGAACGAACCAGAATTTTGCCACaAATATTCATATGGTGTGCGAGCCATTGTTCAATGTATTCCAGCTTGGCTTCGCTTCATCCAGTGCCTGCGCCGGTACCGTGACACGAGGAGGGCTTTTCCTCATCTAGTAAATGCTGGCAAATACTCcACAACTTTCTTCACcGTGACATTTGCAGCCCTTTACAGCACTCACAAACCTCCTCTTTCTCCAGAACAAAAtCACTCTGACACCgtGGTgTTCTTTTACCTGTCTATTACTGCTACAACGTTTAAGCCTCATGTTGGGGAC

>Fra-DB18-1

GAGAAGCTGGCAGAGGCTCAGCGCAGGTTTGCTACACTTCAGAATGAGCTTCAGTCATCGCTGGATGTGCAGAAAGAAAGCAGTGgTGTCACaaCAcTGCGACAgCGCAGAAAGCCAGTGTTCCACCTGTCCCACGAGGAGCGtGTCCAACATAGGAAcATTAAAGACCTTAAGCTGGCCTTCaGCGAGTTCTACCTCAGTCTCATCCTGCTGCAGAACGAACCAGAATTTTGCCACaAATATTCATATGGTGTGCGAGCCATTGTTCAATGTATTCCAGCTTGGCTTCGCTTCATCCAGTGCCTGCGCCGGTACCGTGACACGAGGAGGGCTTTTCCTCATCTAGTAAATGCTGGCAAATACTCcACAACTTTCTTCACcGTGACATTTGCAGCCCTTTACAGCACTCACAAACCTCCTCTTTCTCCAGAACAAAAtCACTCTGACACCgtGGTgTTCTTTTACCTGTCTATTACTGCTACAACGTTTAAGCCTCATGTTGGGGAC

>Fra-DB18-2

GAGAAGCTGGCAGAGGCTCAGCGCAGGTTTGCTACACTTCAGAATGAGCTTCAGTCATCGCTGGATGTGCAGAAAGAAAGCAGTGgTGTCACaaCAcTGCGACAgCGCAGAAAGCCAGTGTTCCACCTGTCCCACGAGGAGCGtGTCCAACATAGGAAcATTAAAGACCTTAAGCTGGCCTTCaGCGAGTTCTACCTCAGTCTCATCCTGCTGCAGAACGAACCAGAATTTTGCCACaAATATTCATATGGTGTGCGAGCCATTGTTCAATGTATTCCAGCTTGGCTTCGCTTCATCCAGTGCCTGCGCCGGTACCGTGACACGAGGAGGGCTTTTCCTCATCTAGTAAATGCTGGCAAATACTCcACAACTTTCTTCACcGTGACATTTGCAGCCCTTTACAGCACTCACAAACCTCCTCTTTCTCCAGAACAAAAtCACTCTGACACCgtGGTgTTCTTTTACCTGTCTATTACTGCTACAACGTTTAAGCCTCATGTTGGGGAC

>Fra-ES0201-1

GAGAAGCTGGCAGAGGCTCAGCGCAGGTTTGCTACACTTCAGAATGAGCTTCAGTCATCGCTGGATGTGCAGAAAGAAAGCAGTGgTGTCACaaCAcTGCGACAgCGCAGAAAGCCAGTGTTCCACCTGTCCCACGAGGAGCGtGTCCAACATAGGAAcATTAAAGACCTTAAGCTGGCCTTCaGCGAGTTCTACCTCAGTCTCATCCTGCTGCAGAACGAACCAGAATTTTGCCACaAATATTCATATGGTGTGCGAGCCATTGTTCAATGTATTCCAGCTTGGCTTCGCTTCATCCAGTGCCTGCGCCGGTACCGTGACACGAGGAGGGCTTTTCCTCATCTAGTAAATGCTGGCAAATACTCcACAACTTTCTTCACcGTGACATTTGCAGCCCTTTACAGCACTCACAAACCTCCTCTTTCTCCAGAACAAAAtCACTCTGACACCgtGGTgTTCTTTTACCTGTCTATTACTGCTACAACGTTTAAGCCTCATGTTGGGGAC

>Fra-ES0201-2

GAGAAGCTGGCAGAGGCTCAGCGCAGGTTTGCTACACTTCAGAATGAGCTTCAGTCATCGCTGGATGTGCAGAAAGAAAGCAGTGgTGTCACaaCAcTGCGACAgCGCAGAAAGCCAGTGTTCCACCTGTCCCACGAGGAGCGtGTCCAACATAGGAAcATTAAAGACCTTAAGCTGGCCTTCaGCGAGTTCTACCTCAGTCTCATCCTGCTGCAGAACGAACCAGAATTTTGCCACaAATATTCATATGGTGTGCGAGCCATTGTTCAATGTATTCCAGCTTGGCTTCGCTTCATCCAGTGCCTGCGCCGGTACCGTGACACGAGGAGGGCTTTTCCTCATCTAGTAAATGCTGGCAAATACTCcACAACTTTCTTCACcGTGACATTTGCAGCCCTTTACAGCACTCACAAACCTCCTCTTTCTCCAGAACAAAAtCACTCTGACACCgtGGTgTTCTTTTACCTGTCTATTACTGCTACAACGTTTAAGCCTCATGTTGGGGAC

>Fra-ES0501-1

GAGAAGCTGGCAGAGGCTCAGCGCAGGTTTGCTACACTTCAGAATGAGCTTCAGTCATCGCTGGATGTGCAGAAAGAAAGCAGTGgTGTCACaaCAcTGCGACAgCGCAGAAAGCCAGTGTTCCACCTGTCCCACGAGGAGCGtGTCCAACATAGGAAcATTAAAGACCTTAAGCTGGCCTTCaGCGAGTTCTACCTCAGTCTCATCCTGCTGCAGAACGAACCAGAATTTTGCCACaAATATTCATATGGTGTGCGAGCCATTGTTCAATGTATTCCAGCTTGGCTTCGCTTCATCCAGTGCCTGCGCCGGTACCGTGACACGAGGAGGGCTTTTCCTCATCTAGTAAATGCTGGCAAATACTCcACAACTTTCTTCACcGTGACATTTGCAGCCCTTTACAGCACTCACAAACCTCCTCTTTCTCCAGAACAAAAtCACTCTGACACCgtGGTgTTCTTTTACCTGTCTATTACTGCTACAACGTTTAAGCCTCATGTTGGGGAC

>Fra-ES0501-2

GAGAAGCTGGCAGAGGCTCAGCGCAGGTTTGCTACACTTCAGAATGAGCTTCAGTCATCGCTGGATGTGCAGAAAGAAAGCAGTGgTGTCACaaCAcTGCGACAgCGCAGAAAGCCAGTGTTCCACCTGTCCCACGAGGAGCGtGTCCAACATAGGAAcATTAAAGACCTTAAGCTGGCCTTCaGCGAGTTCTACCTCAGTCTCATCCTGCTGCAGAACGAACCAGAATTTTGCCACaAATATTCATATGGTGTGCGAGCCATTGTTCAATGTATTCCAGCTTGGCTTCGCTTCATCCAGTGCCTGCGCCGGTACCGTGACACGAGGAGGGCTTTTCCTCATCTAGTAAATGCTGGCAAATACTCcACAACTTTCTTCACcGTGACATTTGCAGCCCTTTACAGCACTCACAAACCTCCTCTTTCTCCAGAACAAAAtCACTCTGACACCgtGGTgTTCTTTTACCTGTCTATTACTGCTACAACGTTTAAGCCTCATGTTGGGGAC

>Fra-ES0901-1

GAGAAGCTGGCAGAGGCTCAGCGCAGGTTTGCTACACTTCAGAATGAGCTTCAGTCATCGCTGGATGTGCAGAAAGAAAGCAGTGgTGTCACaaCAcTGCGACAgCGCAGAAAGCCAGTGTTCCACCTGTCCCACGAGGAGCGtGTCCAACATAGGAAcATTAAAGACCTTAAGCTGGCCTTCaGCGAGTTCTACCTCAGTCTCATCCTGCTGCAGAACGAACCAGAATTTTGCCACaAATATTCATATGGTGTGCGAGCCATTGTTCAATGTATTCCAGCTTGGCTTCGCTTCATCCAGTGCCTGCGCCGGTACCGTGACACGAGGAGGGCTTTTCCTCATCTAGTAAATGCTGGCAAATACTCcACAACTTTCTTCACcGTGACATTTGCAGCCCTTTACAGCACTCACAAACCTCCTCTTTCTCCAGAACAAAAtCACTCTGACACCgtGGTgTTCTTTTACCTGTCTATTACTGCTACAACGTTTAAGCCTCATGTTGGGGAC

>Fra-ES0901-2

GAGAAGCTGGCAGAGGCTCAGCGCAGGTTTGCTACACTTCAGAATGAGCTTCAGTCATCGCTGGATGTGCAGAAAGAAAGCAGTGgTGTCACaaCAcTGCGACAgCGCAGAAAGCCAGTGTTCCACCTGTCCCACGAGGAGCGtGTCCAACATAGGAAcATTAAAGACCTTAAGCTGGCCTTCaGCGAGTTCTACCTCAGTCTCATCCTGCTGCAGAACGAACCAGAATTTTGCCACaAATATTCATATGGTGTGCGAGCCATTGTTCAATGTATTCCAGCTTGGCTTCGCTTCATCCAGTGCCTGCGCCGGTACCGTGACACGAGGAGGGCTTTTCCTCATCTAGTAAATGCTGGCAAATACTCcACAACTTTCTTCACcGTGACATTTGCAGCCCTTTACAGCACTCACAAACCTCCTCTTTCTCCAGAACAAAAtCACTCTGACACCgtGGTgTTCTTTTACCTGTCTATTACTGCTACAACGTTTAAGCCTCATGTTGGGGAC

>Fra-ES1101-1

GAGAAGCTGGCAGAGGCTCAGCGCAGGTTTGCTACACTTCAGAATGAGCTTCAGTCATCGCTGGATGTGCAGAAAGAAAGCAGTGgTGTCACaaCAcTGCGACAgCGCAGAAAGCCAGTGTTCCACCTGTCCCACGAGGAGCGtGTCCAACATAGGAAcATTAAAGACCTTAAGCTGGCCTTCaGCGAGTTCTACCTCAGTCTCATCCTGCTGCAGAACGAACCAGAATTTTGCCACaAATATTCATATGGTGTGCGAGCCATTGTTCAATGTATTCCAGCTTGGCTTCGCTTCATCCAGTGCCTGCGCCGGTACCGTGACACGAGGAGGGCTTTTCCTCATCTAGTAAATGCTGGCAAATACTCcACAACTTTCTTCACcGTGACATTTGCAGCCCTTTACAGCACTCACAAACCTCCTCTTTCTCCAGAACAAAAtCACTCTGACACCgtGGTgTTCTTTTACCTGTCTATTACTGCTACAACGTTTAAGCCTCATGTTGGGGAC

>Fra-ES1101-2

GAGAAGCTGGCAGAGGCTCAGCGCAGGTTTGCTACACTTCAGAATGAGCTTCAGTCATCGCTGGATGTGCAGAAAGAAAGCAGTGcTGTCACagCAtTGCGACAgCGCAGAAAGCCAGTGTTCCACCTGTCCCACGAGGAGCGtGTCCAACATAGGAAcATTAAAGACCTTAAGCTGGCCTTCaGCGAGTTCTACCTCAGTCTCATCCTGCTGCAGAACGAACCAGAATTTTGCCACaAATATTCATATGGTGTGCGAGCCATTGTTCAATGTATTCCAGCTTGGCTTCGCTTCATCCAGTGCCTGCGCCGGTACCGTGACACGAGGAGGGCTTTTCCTCATCTAGTAAATGCTGGCAAATACTCcACAACTTTCTTCACcGTGACATTTGCAGCCCTTTACAGCACTCACAAACCTCCTCTTTCTCCAGAACAAAAtCACTCTGACACCgtGGTgTTCTTTTACCTGTCTATTACTGCTACAACGTTTAAGCCTCATGTTGGGGAC

>Fra-ES1301-1

GAGAAGCTGGCAGAGGCTCAGCGCAGGTTTGCTACACTTCAGAATGAGCTTCAGTCATCGCTGGATGTGCAGAAAGAAAGCAGTGgTGTCACaaCAcTGCGACAgCGCAGAAAGCCAGTGTTCCACCTGTCCCACGAGGAGCGtGTCCAACATAGGAAcATTAAAGACCTTAAGCTGGCCTTCaGCGAGTTCTACCTCAGTCTCATCCTGCTGCAGAACGAACCAGAATTTTGCCACaAATATTCATATGGTGTGCGAGCCATTGTTCAATGTATTCCAGCTTGGCTTCGCTTCATCCAGTGCCTGCGCCGGTACCGTGACACGAGGAGGGCTTTTCCTCATCTAGTAAATGCTGGCAAATACTCcACAACTTTCTTCACcGTGACATTTGCAGCCCTTTACAGCACTCACAAACCTCCTCTTTCTCCAGAACAAAAtCACTCTGACACCgtGGTgTTCTTTTACCTGTCTATTACTGCTACAACGTTTAAGCCTCATGTTGGGGAC

>Fra-ES1301-2

GAGAAGCTGGCAGAGGCTCAGCGCAGGTTTGCTACACTTCAGAATGAGCTTCAGTCATCGCTGGATGTGCAGAAAGAAAGCAGTGcTGTCACagCAtTGCGACAgCGCAGAAAGCCAGTGTTCCACCTGTCCCACGAGGAGCGtGTCCAACATAGGAAcATTAAAGACCTTAAGCTGGCCTTCaGCGAGTTCTACCTCAGTCTCATCCTGCTGCAGAACGAACCAGAATTTTGCCACaAATATTCATATGGTGTGCGAGCCATTGTTCAATGTATTCCAGCTTGGCTTCGCTTCATCCAGTGCCTGCGCCGGTACCGTGACACGAGGAGGGCTTTTCCTCATCTAGTAAATGCTGGCAAATACTCcACAACTTTCTTCACcGTGACATTTGCAGCCCTTTACAGCACTCACAAACCTCCTCTTTCTCCAGAACAAAAtCACTCTGACACCgtGGTgTTCTTTTACCTGTCTATTACTGCTACAACGTTTAAGCCTCATGTTGGGGAC

>Fra-ES1501-1

GAGAAGCTGGCAGAGGCTCAGCGCAGGTTTGCTACACTTCAGAATGAGCTTCAGTCATCGCTGGATGTGCAGAAAGAAAGCAGTGgTGTCACaaCAcTGCGACAgCGCAGAAAGCCAGTGTTCCACCTGTCCCACGAGGAGCGtGTCCAACATAGGAAcATTAAAGACCTTAAGCTGGCCTTCaGCGAGTTCTACCTCAGTCTCATCCTGCTGCAGAACGAACCAGAATTTTGCCACaAATATTCATATGGTGTGCGAGCCATTGTTCAATGTATTCCAGCTTGGCTTCGCTTCATCCAGTGCCTGCGCCGGTACCGTGACACGAGGAGGGCTTTTCCTCATCTAGTAAATGCTGGCAAATACTCcACAACTTTCTTCACcGTGACATTTGCAGCCCTTTACAGCACTCACAAACCTCCTCTTTCTCCAGAACAAAAtCACTCTGACACCgtGGTgTTCTTTTACCTGTCTATTACTGCTACAACGTTTAAGCCTCATGTTGGGGAC

>Fra-ES1501-2

GAGAAGCTGGCAGAGGCTCAGCGCAGGTTTGCTACACTTCAGAATGAGCTTCAGTCATCGCTGGATGTGCAGAAAGAAAGCAGTGcTGTCACagCAtTGCGACAgCGCAGAAAGCCAGTGTTCCACCTGTCCCACGAGGAGCGtGTCCAACATAGGAAcATTAAAGACCTTAAGCTGGCCTTCaGCGAGTTCTACCTCAGTCTCATCCTGCTGCAGAACGAACCAGAATTTTGCCACaAATATTCATATGGTGTGCGAGCCATTGTTCAATGTATTCCAGCTTGGCTTCGCTTCATCCAGTGCCTGCGCCGGTACCGTGACACGAGGAGGGCTTTTCCTCATCTAGTAAATGCTGGCAAATACTCcACAACTTTCTTCACcGTGACATTTGCAGCCCTTTACAGCACTCACAAACCTCCTCTTTCTCCAGAACAAAAtCACTCTGACACCgtGGTgTTCTTTTACCTGTCTATTACTGCTACAACGTTTAAGCCTCATGTTGGGGAC

>Fra-ES1201-1

GAGAAGCTGGCAGAGGCTCAGCGCAGGTTTGCTACACTTCAGAATGAGCTTCAGTCATCGCTGGATGTGCAGAAAGAAAGCAGTGcTGTCACagCAtTGCGACAgCGCAGAAAGCCAGTGTTCCACCTGTCCCACGAGGAGCGtGTCCAACATAGGAAcATTAAAGACCTTAAGCTGGCCTTCaGCGAGTTCTACCTCAGTCTCATCCTGCTGCAGAACGAACCAGAATTTTGCCACaAATATTCATATGGTGTGCGAGCCATTGTTCAATGTATTCCAGCTTGGCTTCGCTTCATCCAGTGCCTGCGCCGGTACCGTGACACGAGGAGGGCTTTTCCTCATCTAGTAAATGCTGGCAAATACTCcACAACTTTCTTCACcGTGACATTTGCAGCCCTTTACAGCACTCACAAACCTCCTCTTTCTCCAGAACAAAAtCACTCTGACACCgtGGTgTTCTTTTACCTGTCTATTACTGCTACAACGTTTAAGCCTCATGTTGGGGAC

>Fra-ES1201-2

GAGAAGCTGGCAGAGGCTCAGCGCAGGTTTGCTACACTTCAGAATGAGCTTCAGTCATCGCTGGATGTGCAGAAAGAAAGCAGTGcTGTCACagCAtTGCGACAgCGCAGAAAGCCAGTGTTCCACCTGTCCCACGAGGAGCGtGTCCAACATAGGAAcATTAAAGACCTTAAGCTGGCCTTCaGCGAGTTCTACCTCAGTCTCATCCTGCTGCAGAACGAACCAGAATTTTGCCACaAATATTCATATGGTGTGCGAGCCATTGTTCAATGTATTCCAGCTTGGCTTCGCTTCATCCAGTGCCTGCGCCGGTACCGTGACACGAGGAGGGCTTTTCCTCATCTAGTAAATGCTGGCAAATACTCcACAACTTTCTTCACcGTGACATTTGCAGCCCTTTACAGCACTCACAAACCTCCTCTTTCTCCAGAACAAAAtCACTCTGACACCgtGGTgTTCTTTTACCTGTCTATTACTGCTACAACGTTTAAGCCTCATGTTGGGGAC

>Fra-ES1401-1

GAGAAGCTGGCAGAGGCTCAGCGCAGGTTTGCTACACTTCAGAATGAGCTTCAGTCATCGCTGGATGTGCAGAAAGAAAGCAGTGcTGTCACagCAtTGCGACAgCGCAGAAAGCCAGTGTTCCACCTGTCCCACGAGGAGCGtGTCCAACATAGGAAcATTAAAGACCTTAAGCTGGCCTTCaGCGAGTTCTACCTCAGTCTCATCCTGCTGCAGAACGAACCAGAATTTTGCCACaAATATTCATATGGTGTGCGAGCCATTGTTCAATGTATTCCAGCTTGGCTTCGCTTCATCCAGTGCCTGCGCCGGTACCGTGACACGAGGAGGGCTTTTCCTCATCTAGTAAATGCTGGCAAATACTCcACAACTTTCTTCACcGTGACATTTGCAGCCCTTTACAGCACTCACAAACCTCCTCTTTCTCCAGAACAAAAtCACTCTGACACCgtGGTgTTCTTTTACCTGTCTATTACTGCTACAACGTTTAAGCCTCATGTTGGGGAC

>Fra-ES1401-2

GAGAAGCTGGCAGAGGCTCAGCGCAGGTTTGCTACACTTCAGAATGAGCTTCAGTCATCGCTGGATGTGCAGAAAGAAAGCAGTGcTGTCACagCAtTGCGACAgCGCAGAAAGCCAGTGTTCCACCTGTCCCACGAGGAGCGtGTCCAACATAGGAAcATTAAAGACCTTAAGCTGGCCTTCaGCGAGTTCTACCTCAGTCTCATCCTGCTGCAGAACGAACCAGAATTTTGCCACaAATATTCATATGGTGTGCGAGCCATTGTTCAATGTATTCCAGCTTGGCTTCGCTTCATCCAGTGCCTGCGCCGGTACCGTGACACGAGGAGGGCTTTTCCTCATCTAGTAAATGCTGGCAAATACTCcACAACTTTCTTCACcGTGACATTTGCAGCCCTTTACAGCACTCACAAACCTCCTCTTTCTCCAGAACAAAAtCACTCTGACACCgtGGTgTTCTTTTACCTGTCTATTACTGCTACAACGTTTAAGCCTCATGTTGGGGAC

>Fra-ES0801-1

GAGAAGCTGGCAGAGGCTCAGCGCAGGTTTGCTACACTTCAGAATGAGCTTCAGTCATCGCTGGATGTGCAGAAAGAAAGCAGTGcTGTCACagCAtTGCGACAgCGCAGAAAGCCAGTGTTCCACCTGTCCCACGAGGAGCGtGTCCAACATAGGAAcATTAAAGACCTTAAGCTGGCCTTCaGCGAGTTCTACCTCAGTCTCATCCTGCTGCAGAACGAACCAGAATTTTGCCACaAATATTCATATGGTGTGCGAGCCATTGTTCAATGTATTCCAGCTTGGCTTCGCTTCATCCAGTGCCTGCGCCGGTACCGTGACACGAGGAGGGCTTTTCCTCATCTAGTAAATGCTGGCAAATACTCcACAACTTTCTTCACcGTGACATTTGCAGCCCTTTACAGCACTCACAAACCTCCTCTTTCTCCAGAACAAAAtCACTCTGACACCgtGGTgTTCTTTTACCTGTCTATTACTGCTACAACGTTTAAGCCTCATGTTGGGGAC

>Fra-ES0801-2

GAGAAGCTGGCAGAGGCTCAGCGCAGGTTTGCTACACTTCAGAATGAGCTTCAGTCATCGCTGGATGTGCAGAAAGAAAGCAGTGcTGTCACagCAtTGCGACAgCGCAGAAAGCCAGTGTTCCACCTGTCCCACGAGGAGCGtGTCCAACATAGGAAcATTAAAGACCTTAAGCTGGCCTTCaGCGAGTTCTACCTCAGTCTCATCCTGCTGCAGAACGAACCAGAATTTTGCCACaAATATTCATATGGTGTGCGAGCCATTGTTCAATGTATTCCAGCTTGGCTTCGCTTCATCCAGTGCCTGCGCCGGTACCGTGACACGAGGAGGGCTTTTCCTCATCTAGTAAATGCTGGCAAATACTCcACAACTTTCTTCACcGTGACATTTGCAGCCCTTTACAGCACTCACAAACCTCCTCTTTCTCCAGAACAAAAtCACTCTGACACCgtGGTgTTCTTTTACCTGTCTATTACTGCTACAACGTTTAAGCCTCATGTTGGGGAC

>Fra-ES0701-1

GAGAAGCTGGCAGAGGCTCAGCGCAGGTTTGCTACACTTCAGAATGAGCTTCAGTCATCGCTGGATGTGCAGAAAGAAAGCAGTGcTGTCACagCAtTGCGACAgCGCAGAAAGCCAGTGTTCCACCTGTCCCACGAGGAGCGtGTCCAACATAGGAAcATTAAAGACCTTAAGCTGGCCTTCaGCGAGTTCTACCTCAGTCTCATCCTGCTGCAGAACGAACCAGAATTTTGCCACaAATATTCATATGGTGTGCGAGCCATTGTTCAATGTATTCCAGCTTGGCTTCGCTTCATCCAGTGCCTGCGCCGGTACCGTGACACGAGGAGGGCTTTTCCTCATCTAGTAAATGCTGGCAAATACTCcACAACTTTCTTCACcGTGACATTTGCAGCCCTTTACAGCACTCACAAACCTCCTCTTTCTCCAGAACAAAAtCACTCTGACACCgtGGTgTTCTTTTACCTGTCTATTACTGCTACAACGTTTAAGCCTCATGTTGGGGAC

>Fra-ES0701-2

GAGAAGCTGGCAGAGGCTCAGCGCAGGTTTGCTACACTTCAGAATGAGCTTCAGTCATCGCTGGATGTGCAGAAAGAAAGCAGTGcTGTCACagCAtTGCGACAgCGCAGAAAGCCAGTGTTCCACCTGTCCCACGAGGAGCGtGTCCAACATAGGAAcATTAAAGACCTTAAGCTGGCCTTCaGCGAGTTCTACCTCAGTCTCATCCTGCTGCAGAACGAACCAGAATTTTGCCACaAATATTCATATGGTGTGCGAGCCATTGTTCAATGTATTCCAGCTTGGCTTCGCTTCATCCAGTGCCTGCGCCGGTACCGTGACACGAGGAGGGCTTTTCCTCATCTAGTAAATGCTGGCAAATACTCcACAACTTTCTTCACcGTGACATTTGCAGCCCTTTACAGCACTCACAAACCTCCTCTTTCTCCAGAACAAAAtCACTCTGACACCgtGGTgTTCTTTTACCTGTCTATTACTGCTACAACGTTTAAGCCTCATGTTGGGGAC

>Fra-ES0601-1

GAGAAGCTGGCAGAGGCTCAGCGCAGGTTTGCTACACTTCAGAATGAGCTTCAGTCATCGCTGGATGTGCAGAAAGAAAGCAGTGcTGTCACagCAtTGCGACAgCGCAGAAAGCCAGTGTTCCACCTGTCCCACGAGGAGCGtGTCCAACATAGGAAcATTAAAGACCTTAAGCTGGCCTTCaGCGAGTTCTACCTCAGTCTCATCCTGCTGCAGAACGAACCAGAATTTTGCCACaAATATTCATATGGTGTGCGAGCCATTGTTCAATGTATTCCAGCTTGGCTTCGCTTCATCCAGTGCCTGCGCCGGTACCGTGACACGAGGAGGGCTTTTCCTCATCTAGTAAATGCTGGCAAATACTCcACAACTTTCTTCACcGTGACATTTGCAGCCCTTTACAGCACTCACAAACCTCCTCTTTCTCCAGAACAAAAtCACTCTGACACCgtGGTgTTCTTTTACCTGTCTATTACTGCTACAACGTTTAAGCCTCATGTTGGGGAC

>Fra-ES0601-2

GAGAAGCTGGCAGAGGCTCAGCGCAGGTTTGCTACACTTCAGAATGAGCTTCAGTCATCGCTGGATGTGCAGAAAGAAAGCAGTGcTGTCACagCAtTGCGACAgCGCAGAAAGCCAGTGTTCCACCTGTCCCACGAGGAGCGtGTCCAACATAGGAAcATTAAAGACCTTAAGCTGGCCTTCaGCGAGTTCTACCTCAGTCTCATCCTGCTGCAGAACGAACCAGAATTTTGCCACaAATATTCATATGGTGTGCGAGCCATTGTTCAATGTATTCCAGCTTGGCTTCGCTTCATCCAGTGCCTGCGCCGGTACCGTGACACGAGGAGGGCTTTTCCTCATCTAGTAAATGCTGGCAAATACTCcACAACTTTCTTCACcGTGACATTTGCAGCCCTTTACAGCACTCACAAACCTCCTCTTTCTCCAGAACAAAAtCACTCTGACACCgtGGTgTTCTTTTACCTGTCTATTACTGCTACAACGTTTAAGCCTCATGTTGGGGAC

>Fra-ES0401-1

GAGAAGCTGGCAGAGGCTCAGCGCAGGTTTGCTACACTTCAGAATGAGCTTCAGTCATCGCTGGATGTGCAGAAAGAAAGCAGTGcTGTCACagCAtTGCGACAgCGCAGAAAGCCAGTGTTCCACCTGTCCCACGAGGAGCGtGTCCAACATAGGAAcATTAAAGACCTTAAGCTGGCCTTCaGCGAGTTCTACCTCAGTCTCATCCTGCTGCAGAACGAACCAGAATTTTGCCACaAATATTCATATGGTGTGCGAGCCATTGTTCAATGTATTCCAGCTTGGCTTCGCTTCATCCAGTGCCTGCGCCGGTACCGTGACACGAGGAGGGCTTTTCCTCATCTAGTAAATGCTGGCAAATACTCcACAACTTTCTTCACcGTGACATTTGCAGCCCTTTACAGCACTCACAAACCTCCTCTTTCTCCAGAACAAAAtCACTCTGACACCgtGGTgTTCTTTTACCTGTCTATTACTGCTACAACGTTTAAGCCTCATGTTGGGGAC

>Fra-ES0401-2

GAGAAGCTGGCAGAGGCTCAGCGCAGGTTTGCTACACTTCAGAATGAGCTTCAGTCATCGCTGGATGTGCAGAAAGAAAGCAGTGcTGTCACagCAtTGCGACAgCGCAGAAAGCCAGTGTTCCACCTGTCCCACGAGGAGCGtGTCCAACATAGGAAcATTAAAGACCTTAAGCTGGCCTTCaGCGAGTTCTACCTCAGTCTCATCCTGCTGCAGAACGAACCAGAATTTTGCCACaAATATTCATATGGTGTGCGAGCCATTGTTCAATGTATTCCAGCTTGGCTTCGCTTCATCCAGTGCCTGCGCCGGTACCGTGACACGAGGAGGGCTTTTCCTCATCTAGTAAATGCTGGCAAATACTCcACAACTTTCTTCACcGTGACATTTGCAGCCCTTTACAGCACTCACAAACCTCCTCTTTCTCCAGAACAAAAtCACTCTGACACCgtGGTgTTCTTTTACCTGTCTATTACTGCTACAACGTTTAAGCCTCATGTTGGGGAC

>Fra-LO101-1

GAGAAGCTGGCAGAGGCTCAGCGCAGGTTTGCTACACTTCAGAATGAGCTTCAGTCATCGCTGGATGTGCAGAAAGAAAGCAGTGgTGTCACaaCAcTGCGACAgCGCAGAAAGCCAGTGTTCCACCTGTCCCACGAGGAGCGtGTCCAACATAGGAAcATTAAAGACCTTAAGCTGGCCTTCaGCGAGTTCTACCTCAGTCTCATCCTGCTGCAGAACGAACCAGAATTTTGCCACaAATATTCATATGGTGTGCGAGCCATTGTTCAATGTATTCCAGCTTGGCTTCGCTTCATCCAGTGCCTGCGCCGGTACCGTGACACGAGGAGGGCTTTTCCTCATCTAGTAAATGCTGGCAAATACTCcACAACTTTCTTCACcGTGACATTTGCAGCCCTTTACAGCACTCACAAACCTCCTCTTTCTCCAGAACAAAAtCACTCTGACACCgtGGTgTTCTTTTACCTGTCTATTACTGCTACAACGTTTAAGCCTCATGTTGGGGAC

>Fra-LO101-2

GAGAAGCTGGCAGAGGCTCAGCGCAGGTTTGCTACACTTCAGAATGAGCTTCAGTCATCGCTGGATGTGCAGAAAGAAAGCAGTGgTGTCACaaCAcTGCGACAgCGCAGAAAGCCAGTGTTCCACCTGTCCCACGAGGAGCGtGTCCAACATAGGAAcATTAAAGACCTTAAGCTGGCCTTCaGCGAGTTCTACCTCAGTCTCATCCTGCTGCAGAACGAACCAGAATTTTGCCACaAATATTCATATGGTGTGCGAGCCATTGTTCAATGTATTCCAGCTTGGCTTCGCTTCATCCAGTGCCTGCGCCGGTACCGTGACACGAGGAGGGCTTTTCCTCATCTAGTAAATGCTGGCAAATACTCcACAACTTTCTTCACcGTGACATTTGCAGCCCTTTACAGCACTCACAAACCTCCTCTTTCTCCAGAACAAAAtCACTCTGACACCgtGGTgTTCTTTTACCTGTCTATTACTGCTACAACGTTTAAGCCTCATGTTGGGGAC

>Fra-LO13-1

GAGAAGCTGGCAGAGGCTCAGCGCAGGTTTGCTACACTTCAGAATGAGCTTCAGTCATCGCTGGATGTGCAGAAAGAAAGCAGTGgTGTCACaaCAcTGCGACAgCGCAGAAAGCCAGTGTTCCACCTGTCCCACGAGGAGCGtGTCCAACATAGGAAcATTAAAGACCTTAAGCTGGCCTTCaGCGAGTTCTACCTCAGTCTCATCCTGCTGCAGAACGAACCAGAATTTTGCCACaAATATTCATATGGTGTGCGAGCCATTGTTCAATGTATTCCAGCTTGGCTTCGCTTCATCCAGTGCCTGCGCCGGTACCGTGACACGAGGAGGGCTTTTCCTCATCTAGTAAATGCTGGCAAATACTCcACAACTTTCTTCACcGTGACATTTGCAGCCCTTTACAGCACTCACAAACCTCCTCTTTCTCCAGAACAAAAtCACTCTGACACCgtGGTgTTCTTTTACCTGTCTATTACTGCTACAACGTTTAAGCCTCATGTTGGGGAC

>Fra-LO13-2

GAGAAGCTGGCAGAGGCTCAGCGCAGGTTTGCTACACTTCAGAATGAGCTTCAGTCATCGCTGGATGTGCAGAAAGAAAGCAGTGgTGTCACaaCAcTGCGACAgCGCAGAAAGCCAGTGTTCCACCTGTCCCACGAGGAGCGtGTCCAACATAGGAAcATTAAAGACCTTAAGCTGGCCTTCaGCGAGTTCTACCTCAGTCTCATCCTGCTGCAGAACGAACCAGAATTTTGCCACaAATATTCATATGGTGTGCGAGCCATTGTTCAATGTATTCCAGCTTGGCTTCGCTTCATCCAGTGCCTGCGCCGGTACCGTGACACGAGGAGGGCTTTTCCTCATCTAGTAAATGCTGGCAAATACTCcACAACTTTCTTCACcGTGACATTTGCAGCCCTTTACAGCACTCACAAACCTCCTCTTTCTCCAGAACAAAAtCACTCTGACACCgtGGTgTTCTTTTACCTGTCTATTACTGCTACAACGTTTAAGCCTCATGTTGGGGAC

>Fra-LO21-1

GAGAAGCTGGCAGAGGCTCAGCGCAGGTTTGCTACACTTCAGAATGAGCTTCAGTCATCGCTGGATGTGCAGAAAGAAAGCAGTGgTGTCACaaCAcTGCGACAgCGCAGAAAGCCAGTGTTCCACCTGTCCCACGAGGAGCGtGTCCAACATAGGAAcATTAAAGACCTTAAGCTGGCCTTCaGCGAGTTCTACCTCAGTCTCATCCTGCTGCAGAACGAACCAGAATTTTGCCACaAATATTCATATGGTGTGCGAGCCATTGTTCAATGTATTCCAGCTTGGCTTCGCTTCATCCAGTGCCTGCGCCGGTACCGTGACACGAGGAGGGCTTTTCCTCATCTAGTAAATGCTGGCAAATACTCcACAACTTTCTTCACcGTGACATTTGCAGCCCTTTACAGCACTCACAAACCTCCTCTTTCTCCAGAACAAAAtCACTCTGACACCgtGGTgTTCTTTTACCTGTCTATTACTGCTACAACGTTTAAGCCTCATGTTGGGGAC

>Fra-LO21-2

GAGAAGCTGGCAGAGGCTCAGCGCAGGTTTGCTACACTTCAGAATGAGCTTCAGTCATCGCTGGATGTGCAGAAAGAAAGCAGTGgTGTCACaaCAcTGCGACAgCGCAGAAAGCCAGTGTTCCACCTGTCCCACGAGGAGCGtGTCCAACATAGGAAcATTAAAGACCTTAAGCTGGCCTTCaGCGAGTTCTACCTCAGTCTCATCCTGCTGCAGAACGAACCAGAATTTTGCCACaAATATTCATATGGTGTGCGAGCCATTGTTCAATGTATTCCAGCTTGGCTTCGCTTCATCCAGTGCCTGCGCCGGTACCGTGACACGAGGAGGGCTTTTCCTCATCTAGTAAATGCTGGCAAATACTCcACAACTTTCTTCACcGTGACATTTGCAGCCCTTTACAGCACTCACAAACCTCCTCTTTCTCCAGAACAAAAtCACTCTGACACCgtGGTgTTCTTTTACCTGTCTATTACTGCTACAACGTTTAAGCCTCATGTTGGGGAC

>Fra-LO31-1

GAGAAGCTGGCAGAGGCTCAGCGCAGGTTTGCTACACTTCAGAATGAGCTTCAGTCATCGCTGGATGTGCAGAAAGAAAGCAGTGgTGTCACaaCAcTGCGACAgCGCAGAAAGCCAGTGTTCCACCTGTCCCACGAGGAGCGtGTCCAACATAGGAAcATTAAAGACCTTAAGCTGGCCTTCaGCGAGTTCTACCTCAGTCTCATCCTGCTGCAGAACGAACCAGAATTTTGCCACaAATATTCATATGGTGTGCGAGCCATTGTTCAATGTATTCCAGCTTGGCTTCGCTTCATCCAGTGCCTGCGCCGGTACCGTGACACGAGGAGGGCTTTTCCTCATCTAGTAAATGCTGGCAAATACTCcACAACTTTCTTCACcGTGACATTTGCAGCCCTTTACAGCACTCACAAACCTCCTCTTTCTCCAGAACAAAAtCACTCTGACACCgtGGTgTTCTTTTACCTGTCTATTACTGCTACAACGTTTAAGCCTCATGTTGGGGAC

>Fra-LO31-2

GAGAAGCTGGCAGAGGCTCAGCGCAGGTTTGCTACACTTCAGAATGAGCTTCAGTCATCGCTGGATGTGCAGAAAGAAAGCAGTGgTGTCACaaCAcTGCGACAgCGCAGAAAGCCAGTGTTCCACCTGTCCCACGAGGAGCGtGTCCAACATAGGAAcATTAAAGACCTTAAGCTGGCCTTCaGCGAGTTCTACCTCAGTCTCATCCTGCTGCAGAACGAACCAGAATTTTGCCACaAATATTCATATGGTGTGCGAGCCATTGTTCAATGTATTCCAGCTTGGCTTCGCTTCATCCAGTGCCTGCGCCGGTACCGTGACACGAGGAGGGCTTTTCCTCATCTAGTAAATGCTGGCAAATACTCcACAACTTTCTTCACcGTGACATTTGCAGCCCTTTACAGCACTCACAAACCTCCTCTTTCTCCAGAACAAAAtCACTCTGACACCgtGGTgTTCTTTTACCTGTCTATTACTGCTACAACGTTTAAGCCTCATGTTGGGGAC

>Fra-LO141-1

GAGAAGCTGGCAGAGGCTCAGCGCAGGTTTGCTACACTTCAGAATGAGCTTCAGTCATCGCTGGATGTGCAGAAAGAAAGCAGTGgTGTCACaaCAcTGCGACAgCGCAGAAAGCCAGTGTTCCACCTGTCCCACGAGGAGCGtGTCCAACATAGGAAcATTAAAGACCTTAAGCTGGCCTTCaGCGAGTTCTACCTCAGTCTCATCCTGCTGCAGAACGAACCAGAATTTTGCCACaAATATTCATATGGTGTGCGAGCCATTGTTCAATGTATTCCAGCTTGGCTTCGCTTCATCCAGTGCCTGCGCCGGTACCGTGACACGAGGAGGGCTTTTCCTCATCTAGTAAATGCTGGCAAATACTCcACAACTTTCTTCACcGTGACATTTGCAGCCCTTTACAGCACTCACAAACCTCCTCTTTCTCCAGAACAAAAtCACTCTGACACCgtGGTgTTCTTTTACCTGTCTATTACTGCTACAACGTTTAAGCCTCATGTTGGGGAC

>Fra-LO141-2

GAGAAGCTGGCAGAGGCTCAGCGCAGGTTTGCTACACTTCAGAATGAGCTTCAGTCATCGCTGGATGTGCAGAAAGAAAGCAGTGcTGTCACagCAtTGCGACAgCGCAGAAAGCCAGTGTTCCACCTGTCCCACGAGGAGCGtGTCCAACATAGGAAcATTAAAGACCTTAAGCTGGCCTTCaGCGAGTTCTACCTCAGTCTCATCCTGCTGCAGAACGAACCAGAATTTTGCCACaAATATTCATATGGTGTGCGAGCCATTGTTCAATGTATTCCAGCTTGGCTTCGCTTCATCCAGTGCCTGCGCCGGTACCGTGACACGAGGAGGGCTTTTCCTCATCTAGTAAATGCTGGCAAATACTCcACAACTTTCTTCACcGTGACATTTGCAGCCCTTTACAGCACTCACAAACCTCCTCTTTCTCCAGAACAAAAtCACTCTGACACCgtGGTgTTCTTTTACCTGTCTATTACTGCTACAACGTTTAAGCCTCATGTTGGGGAC

>Fra-LO81-1

GAGAAGCTGGCAGAGGCTCAGCGCAGGTTTGCTACACTTCAGAATGAGCTTCAGTCATCGCTGGATGTGCAGAAAGAAAGCAGTGgTGTCACaaCAcTGCGACAgCGCAGAAAGCCAGTGTTCCACCTGTCCCACGAGGAGCGtGTCCAACATAGGAAcATTAAAGACCTTAAGCTGGCCTTCaGCGAGTTCTACCTCAGTCTCATCCTGCTGCAGAACGAACCAGAATTTTGCCACaAATATTCATATGGTGTGCGAGCCATTGTTCAATGTATTCCAGCTTGGCTTCGCTTCATCCAGTGCCTGCGCCGGTACCGTGACACGAGGAGGGCTTTTCCTCATCTAGTAAATGCTGGCAAATACTCcACAACTTTCTTCACcGTGACATTTGCAGCCCTTTACAGCACTCACAAACCTCCTCTTTCTCCAGAACAAAAtCACTCTGACACCgtGGTgTTCTTTTACCTGTCTATTACTGCTACAACGTTTAAGCCTCATGTTGGGGAC

>Fra-LO81-2

GAGAAGCTGGCAGAGGCTCAGCGCAGGTTTGCTACACTTCAGAATGAGCTTCAGTCATCGCTGGATGTGCAGAAAGAAAGCAGTGcTGTCACagCAtTGCGACAgCGCAGAAAGCCAGTGTTCCACCTGTCCCACGAGGAGCGtGTCCAACATAGGAAcATTAAAGACCTTAAGCTGGCCTTCaGCGAGTTCTACCTCAGTCTCATCCTGCTGCAGAACGAACCAGAATTTTGCCACaAATATTCATATGGTGTGCGAGCCATTGTTCAATGTATTCCAGCTTGGCTTCGCTTCATCCAGTGCCTGCGCCGGTACCGTGACACGAGGAGGGCTTTTCCTCATCTAGTAAATGCTGGCAAATACTCcACAACTTTCTTCACcGTGACATTTGCAGCCCTTTACAGCACTCACAAACCTCCTCTTTCTCCAGAACAAAAtCACTCTGACACCgtGGTgTTCTTTTACCTGTCTATTACTGCTACAACGTTTAAGCCTCATGTTGGGGAC

>Fra-LO91-1

GAGAAGCTGGCAGAGGCTCAGCGCAGGTTTGCTACACTTCAGAATGAGCTTCAGTCATCGCTGGATGTGCAGAAAGAAAGCAGTGgTGTCACaaCAcTGCGACAgCGCAGAAAGCCAGTGTTCCACCTGTCCCACGAGGAGCGtGTCCAACATAGGAAcATTAAAGACCTTAAGCTGGCCTTCaGCGAGTTCTACCTCAGTCTCATCCTGCTGCAGAACGAACCAGAATTTTGCCACaAATATTCATATGGTGTGCGAGCCATTGTTCAATGTATTCCAGCTTGGCTTCGCTTCATCCAGTGCCTGCGCCGGTACCGTGACACGAGGAGGGCTTTTCCTCATCTAGTAAATGCTGGCAAATACTCcACAACTTTCTTCACcGTGACATTTGCAGCCCTTTACAGCACTCACAAACCTCCTCTTTCTCCAGAACAAAAtCACTCTGACACCgtGGTgTTCTTTTACCTGTCTATTACTGCTACAACGTTTAAGCCTCATGTTGGGGAC

>Fra-LO91-2

GAGAAGCTGGCAGAGGCTCAGCGCAGGTTTGCTACACTTCAGAATGAGCTTCAGTCATCGCTGGATGTGCAGAAAGAAAGCAGTGcTGTCACagCAtTGCGACAgCGCAGAAAGCCAGTGTTCCACCTGTCCCACGAGGAGCGtGTCCAACATAGGAAcATTAAAGACCTTAAGCTGGCCTTCaGCGAGTTCTACCTCAGTCTCATCCTGCTGCAGAACGAACCAGAATTTTGCCACaAATATTCATATGGTGTGCGAGCCATTGTTCAATGTATTCCAGCTTGGCTTCGCTTCATCCAGTGCCTGCGCCGGTACCGTGACACGAGGAGGGCTTTTCCTCATCTAGTAAATGCTGGCAAATACTCcACAACTTTCTTCACcGTGACATTTGCAGCCCTTTACAGCACTCACAAACCTCCTCTTTCTCCAGAACAAAAtCACTCTGACACCgtGGTgTTCTTTTACCTGTCTATTACTGCTACAACGTTTAAGCCTCATGTTGGGGAC

>Fra-LO51-1

GAGAAGCTGGCAGAGGCTCAGCGCAGGTTTGCTACACTTCAGAATGAGCTTCAGTCATCGCTGGATGTGCAGAAAGAAAGCAGTGgTGTCACaaCAcTGCGACAgCGCAGAAAGCCAGTGTTCCACCTGTCCCACGAGGAGCGtGTCCAACATAGGAAcATTAAAGACCTTAAGCTGGCCTTCaGCGAGTTCTACCTCAGTCTCATCCTGCTGCAGAACGAACCAGAATTTTGCCACaAATATTCATATGGTGTGCGAGCCATTGTTCAATGTATTCCAGCTTGGCTTCGCTTCATCCAGTGCCTGCGCCGGTACCGTGACACGAGGAGGGCTTTTCCTCATCTAGTAAATGCTGGCAAATACTCcACAACTTTCTTCACcGTGACATTTGCAGCCCTTTACAGCACTCACAAACCTCCTCTTTCTCCAGAACAAAAtCACTCTGACACCgtGGTgTTCTTTTACCTGTCTATTACTGCTACAACGTTTAAGCCTCATGTTGGGGAC

>Fra-LO51-2

GAGAAGCTGGCAGAGGCTCAGCGCAGGTTTGCTACACTTCAGAATGAGCTTCAGTCATCGCTGGATGTGCAGAAAGAAAGCAGTGcTGTCACagCAtTGCGACAgCGCAGAAAGCCAGTGTTCCACCTGTCCCACGAGGAGCGtGTCCAACATAGGAAcATTAAAGACCTTAAGCTGGCCTTCaGCGAGTTCTACCTCAGTCTCATCCTGCTGCAGAACGAACCAGAATTTTGCCACaAATATTCATATGGTGTGCGAGCCATTGTTCAATGTATTCCAGCTTGGCTTCGCTTCATCCAGTGCCTGCGCCGGTACCGTGACACGAGGAGGGCTTTTCCTCATCTAGTAAATGCTGGCAAATACTCcACAACTTTCTTCACcGTGACATTTGCAGCCCTTTACAGCACTCACAAACCTCCTCTTTCTCCAGAACAAAAtCACTCTGACACCgtGGTgTTCTTTTACCTGTCTATTACTGCTACAACGTTTAAGCCTCATGTTGGGGAC

>Fra-LO41-1

GAGAAGCTGGCAGAGGCTCAGCGCAGGTTTGCTACACTTCAGAATGAGCTTCAGTCATCGCTGGATGTGCAGAAAGAAAGCAGTGgTGTCACaaCAcTGCGACAgCGCAGAAAGCCAGTGTTCCACCTGTCCCACGAGGAGCGtGTCCAACATAGGAAcATTAAAGACCTTAAGCTGGCCTTCaGCGAGTTCTACCTCAGTCTCATCCTGCTGCAGAACGAACCAGAATTTTGCCACaAATATTCATATGGTGTGCGAGCCATTGTTCAATGTATTCCAGCTTGGCTTCGCTTCATCCAGTGCCTGCGCCGGTACCGTGACACGAGGAGGGCTTTTCCTCATCTAGTAAATGCTGGCAAATACTCcACAACTTTCTTCACcGTGACATTTGCAGCCCTTTACAGCACTCACAAACCTCCTCTTTCTCCAGAACAAAAtCACTCTGACACCgtGGTgTTCTTTTACCTGTCTATTACTGCTACAACGTTTAAGCCTCATGTTGGGGAC

>Fra-LO41-2

GAGAAGCTGGCAGAGGCTCAGCGCAGGTTTGCTACACTTCAGAATGAGCTTCAGTCATCGCTGGATGTGCAGAAAGAAAGCAGTGcTGTCACagCAtTGCGACAgCGCAGAAAGCCAGTGTTCCACCTGTCCCACGAGGAGCGtGTCCAACATAGGAAcATTAAAGACCTTAAGCTGGCCTTCaGCGAGTTCTACCTCAGTCTCATCCTGCTGCAGAACGAACCAGAATTTTGCCACaAATATTCATATGGTGTGCGAGCCATTGTTCAATGTATTCCAGCTTGGCTTCGCTTCATCCAGTGCCTGCGCCGGTACCGTGACACGAGGAGGGCTTTTCCTCATCTAGTAAATGCTGGCAAATACTCcACAACTTTCTTCACcGTGACATTTGCAGCCCTTTACAGCACTCACAAACCTCCTCTTTCTCCAGAACAAAAtCACTCTGACACCgtGGTgTTCTTTTACCTGTCTATTACTGCTACAACGTTTAAGCCTCATGTTGGGGAC

>Fra-LO11-1

GAGAAGCTGGCAGAGGCTCAGCGCAGGTTTGCTACACTTCAGAATGAGCTTCAGTCATCGCTGGATGTGCAGAAAGAAAGCAGTGgTGTCACaaCAcTGCGACAgCGCAGAAAGCCAGTGTTCCACCTGTCCCACGAGGAGCGtGTCCAACATAGGAAcATTAAAGACCTTAAGCTGGCCTTCaGCGAGTTCTACCTCAGTCTCATCCTGCTGCAGAACGAACCAGAATTTTGCCACaAATATTCATATGGTGTGCGAGCCATTGTTCAATGTATTCCAGCTTGGCTTCGCTTCATCCAGTGCCTGCGCCGGTACCGTGACACGAGGAGGGCTTTTCCTCATCTAGTAAATGCTGGCAAATACTCcACAACTTTCTTCACcGTGACATTTGCAGCCCTTTACAGCACTCACAAACCTCCTCTTTCTCCAGAACAAAAtCACTCTGACACCgtGGTgTTCTTTTACCTGTCTATTACTGCTACAACGTTTAAGCCTCATGTTGGGGAC

>Fra-LO11-2

GAGAAGCTGGCAGAGGCTCAGCGCAGGTTTGCTACACTTCAGAATGAGCTTCAGTCATCGCTGGATGTGCAGAAAGAAAGCAGTGcTGTCACagCAtTGCGACAgCGCAGAAAGCCAGTGTTCCACCTGTCCCACGAGGAGCGtGTCCAACATAGGAAcATTAAAGACCTTAAGCTGGCCTTCaGCGAGTTCTACCTCAGTCTCATCCTGCTGCAGAACGAACCAGAATTTTGCCACaAATATTCATATGGTGTGCGAGCCATTGTTCAATGTATTCCAGCTTGGCTTCGCTTCATCCAGTGCCTGCGCCGGTACCGTGACACGAGGAGGGCTTTTCCTCATCTAGTAAATGCTGGCAAATACTCcACAACTTTCTTCACcGTGACATTTGCAGCCCTTTACAGCACTCACAAACCTCCTCTTTCTCCAGAACAAAAtCACTCTGACACCgtGGTgTTCTTTTACCTGTCTATTACTGCTACAACGTTTAAGCCTCATGTTGGGGAC

>Fra-LO61-1

GAGAAGCTGGCAGAGGCTCAGCGCAGGTTTGCTACACTTCAGAATGAGCTTCAGTCATCGCTGGATGTGCAGAAAGAAAGCAGTGcTGTCACagCAtTGCGACAgCGCAGAAAGCCAGTGTTCCACCTGTCCCACGAGGAGCGtGTCCAACATAGGAAcATTAAAGACCTTAAGCTGGCCTTCaGCGAGTTCTACCTCAGTCTCATCCTGCTGCAGAACGAACCAGAATTTTGCCACaAATATTCATATGGTGTGCGAGCCATTGTTCAATGTATTCCAGCTTGGCTTCGCTTCATCCAGTGCCTGCGCCGGTACCGTGACACGAGGAGGGCTTTTCCTCATCTAGTAAATGCTGGCAAATACTCcACAACTTTCTTCACcGTGACATTTGCAGCCCTTTACAGCACTCACAAACCTCCTCTTTCTCCAGAACAAAAtCACTCTGACACCgtGGTgTTCTTTTACCTGTCTATTACTGCTACAACGTTTAAGCCTCATGTTGGGGAC

>Fra-LO61-2

GAGAAGCTGGCAGAGGCTCAGCGCAGGTTTGCTACACTTCAGAATGAGCTTCAGTCATCGCTGGATGTGCAGAAAGAAAGCAGTGcTGTCACagCAtTGCGACAgCGCAGAAAGCCAGTGTTCCACCTGTCCCACGAGGAGCGtGTCCAACATAGGAAcATTAAAGACCTTAAGCTGGCCTTCaGCGAGTTCTACCTCAGTCTCATCCTGCTGCAGAACGAACCAGAATTTTGCCACaAATATTCATATGGTGTGCGAGCCATTGTTCAATGTATTCCAGCTTGGCTTCGCTTCATCCAGTGCCTGCGCCGGTACCGTGACACGAGGAGGGCTTTTCCTCATCTAGTAAATGCTGGCAAATACTCcACAACTTTCTTCACcGTGACATTTGCAGCCCTTTACAGCACTCACAAACCTCCTCTTTCTCCAGAACAAAAtCACTCTGACACCgtGGTgTTCTTTTACCTGTCTATTACTGCTACAACGTTTAAGCCTCATGTTGGGGAC

>Fra-LO71-1

GAGAAGCTGGCAGAGGCTCAGCGCAGGTTTGCTACACTTCAGAATGAGCTTCAGTCATCGCTGGATGTGCAGAAAGAAAGCAGTGcTGTCACagCAtTGCGACAgCGCAGAAAGCCAGTGTTCCACCTGTCCCACGAGGAGCGtGTCCAACATAGGAAcATTAAAGACCTTAAGCTGGCCTTCaGCGAGTTCTACCTCAGTCTCATCCTGCTGCAGAACGAACCAGAATTTTGCCACaAATATTCATATGGTGTGCGAGCCATTGTTCAATGTATTCCAGCTTGGCTTCGCTTCATCCAGTGCCTGCGCCGGTACCGTGACACGAGGAGGGCTTTTCCTCATCTAGTAAATGCTGGCAAATACTCcACAACTTTCTTCACcGTGACATTTGCAGCCCTTTACAGCACTCACAAACCTCCTCTTTCTCCAGAACAAAAtCACTCTGACACCgtGGTgTTCTTTTACCTGTCTATTACTGCTACAACGTTTAAGCCTCATGTTGGGGAC

>Fra-LO71-2

GAGAAGCTGGCAGAGGCTCAGCGCAGGTTTGCTACACTTCAGAATGAGCTTCAGTCATCGCTGGATGTGCAGAAAGAAAGCAGTGcTGTCACagCAtTGCGACAgCGCAGAAAGCCAGTGTTCCACCTGTCCCACGAGGAGCGtGTCCAACATAGGAAcATTAAAGACCTTAAGCTGGCCTTCaGCGAGTTCTACCTCAGTCTCATCCTGCTGCAGAACGAACCAGAATTTTGCCACaAATATTCATATGGTGTGCGAGCCATTGTTCAATGTATTCCAGCTTGGCTTCGCTTCATCCAGTGCCTGCGCCGGTACCGTGACACGAGGAGGGCTTTTCCTCATCTAGTAAATGCTGGCAAATACTCcACAACTTTCTTCACcGTGACATTTGCAGCCCTTTACAGCACTCACAAACCTCCTCTTTCTCCAGAACAAAAtCACTCTGACACCgtGGTgTTCTTTTACCTGTCTATTACTGCTACAACGTTTAAGCCTCATGTTGGGGAC

>Fra-MC1_27-1

GAGAAGCTGGCAGAGGCTCAGCGCAGGTTTGCTACACTTCAGAATGAGCTTCAGTCATCGCTGGATGTGCAGAAAGAAAGCAGTGgTGTCACgaCAtTGCGACAgCGCAGAAAGCCAGTGTTCCACCTGTCCCACGAGGAGCGtGTCCAACATAGGAAcATTAAAGACCTTAAGCTGGCCTTCaGCGAGTTCTACCTCAGTCTCATCCTGCTGCAGAACGAACCAGAATTTTGCCACaAATATTCATATGGTGTGCGAGCCATTGTTCAATGTATTCCAGCTTGGCTTCGCTTCATCCAGTGCCTGCGCCGGTACCGTGACACGAGGAGGGCTTTTCCTCATCTAGTAAATGCTGGCAAATACTCcACAACTTTCTTCACtGTGACATTTGCAGCCCTTTACAGCACTCACAAACCTCCTCTTTCTCCAGAACAAAAaCACTCTGACACCgtGGTgTTCTTTTACCTGTCTATTACTGCTACAACGTTTAAGCCTCATGTTGGGGAC

>Fra-MC1_27-2

GAGAAGCTGGCAGAGGCTCAGCGCAGGTTTGCTACACTTCAGAATGAGCTTCAGTCATCGCTGGATGTGCAGAAAGAAAGCAGTGcTGTCACagCAtTGCGACAgCGCAGAAAGCCAGTGTTCCACCTGTCCCACGAGGAGCGtGTCCAACATAGGAAcATTAAAGACCTTAAGCTGGCCTTCaGCGAGTTCTACCTCAGTCTCATCCTGCTGCAGAACGAACCAGAATTTTGCCACaAATATTCATATGGTGTGCGAGCCATTGTTCAATGTATTCCAGCTTGGCTTCGCTTCATCCAGTGCCTGCGCCGGTACCGTGACACGAGGAGGGCTTTTCCTCATCTAGTAAATGCTGGCAAATACTCcACAACTTTCTTCACcGTGACATTTGCAGCCCTTTACAGCACTCACAAACCTCCTCTTTCTCCAGAACAAAAtCACTCTGACACCgtGGTgTTCTTTTACCTGTCTATTACTGCTACAACGTTTAAGCCTCATGTTGGGGAC

>Fra-MC1_2-1

GAGAAGCTGGCAGAGGCTCAGCGCAGGTTTGCTACACTTCAGAATGAGCTTCAGTCATCGCTGGATGTGCAGAAAGAAAGCAGTGgTGTCACgaCAtTGCGACAgCGCAGAAAGCCAGTGTTCCACCTGTCCCACGAGGAGCGtGTCCAACATAGGAAcATTAAAGACCTTAAGCTGGCCTTCaGCGAGTTCTACCTCAGTCTCATCCTGCTGCAGAACGAACCAGAATTTTGCCACaAATATTCATATGGTGTGCGAGCCATTGTTCAATGTATTCCAGCTTGGCTTCGCTTCATCCAGTGCCTGCGCCGGTACCGTGACACGAGGAGGGCTTTTCCTCATCTAGTAAATGCTGGCAAATACTCcACAACTTTCTTCACcGTGACATTTGCAGCCCTTTACAGCACTCACAAACCTCCTCTTTCTCCAGAACAAAAaCACTCTGACACCgtGGTgTTCTTTTACCTGTCTATTACTGCTACAACGTTTAAGCCTCATGTTGGGGAC

>Fra-MC1_2-2

GAGAAGCTGGCAGAGGCTCAGCGCAGGTTTGCTACACTTCAGAATGAGCTTCAGTCATCGCTGGATGTGCAGAAAGAAAGCAGTGcTGTCACagCAtTGCGACAgCGCAGAAAGCCAGTGTTCCACCTGTCCCACGAGGAGCGtGTCCAACATAGGAAcATTAAAGACCTTAAGCTGGCCTTCaGCGAGTTCTACCTCAGTCTCATCCTGCTGCAGAACGAACCAGAATTTTGCCACaAATATTCATATGGTGTGCGAGCCATTGTTCAATGTATTCCAGCTTGGCTTCGCTTCATCCAGTGCCTGCGCCGGTACCGTGACACGAGGAGGGCTTTTCCTCATCTAGTAAATGCTGGCAAATACTCcACAACTTTCTTCACcGTGACATTTGCAGCCCTTTACAGCACTCACAAACCTCCTCTTTCTCCAGAACAAAAtCACTCTGACACCgtGGTgTTCTTTTACCTGTCTATTACTGCTACAACGTTTAAGCCTCATGTTGGGGAC

>Fra-MC1_3-1

GAGAAGCTGGCAGAGGCTCAGCGCAGGTTTGCTACACTTCAGAATGAGCTTCAGTCATCGCTGGATGTGCAGAAAGAAAGCAGTGgTGTCACgaCAtTGCGACAgCGCAGAAAGCCAGTGTTCCACCTGTCCCACGAGGAGCGtGTCCAACATAGGAAcATTAAAGACCTTAAGCTGGCCTTCaGCGAGTTCTACCTCAGTCTCATCCTGCTGCAGAACGAACCAGAATTTTGCCACaAATATTCATATGGTGTGCGAGCCATTGTTCAATGTATTCCAGCTTGGCTTCGCTTCATCCAGTGCCTGCGCCGGTACCGTGACACGAGGAGGGCTTTTCCTCATCTAGTAAATGCTGGCAAATACTCcACAACTTTCTTCACcGTGACATTTGCAGCCCTTTACAGCACTCACAAACCTCCTCTTTCTCCAGAACAAAAaCACTCTGACACCgtGGTgTTCTTTTACCTGTCTATTACTGCTACAACGTTTAAGCCTCATGTTGGGGAC

>Fra-MC1_3-2

GAGAAGCTGGCAGAGGCTCAGCGCAGGTTTGCTACACTTCAGAATGAGCTTCAGTCATCGCTGGATGTGCAGAAAGAAAGCAGTGcTGTCACagCAtTGCGACAgCGCAGAAAGCCAGTGTTCCACCTGTCCCACGAGGAGCGtGTCCAACATAGGAAcATTAAAGACCTTAAGCTGGCCTTCaGCGAGTTCTACCTCAGTCTCATCCTGCTGCAGAACGAACCAGAATTTTGCCACaAATATTCATATGGTGTGCGAGCCATTGTTCAATGTATTCCAGCTTGGCTTCGCTTCATCCAGTGCCTGCGCCGGTACCGTGACACGAGGAGGGCTTTTCCTCATCTAGTAAATGCTGGCAAATACTCcACAACTTTCTTCACcGTGACATTTGCAGCCCTTTACAGCACTCACAAACCTCCTCTTTCTCCAGAACAAAAtCACTCTGACACCgtGGTgTTCTTTTACCTGTCTATTACTGCTACAACGTTTAAGCCTCATGTTGGGGAC

>Fra-MC1_18-1

GAGAAGCTGGCAGAGGCTCAGCGCAGGTTTGCTACACTTCAGAATGAGCTTCAGTCATCGCTGGATGTGCAGAAAGAAAGCAGTGgTGTCACgaCAtTGCGACAgCGCAGAAAGCCAGTGTTCCACCTGTCCCACGAGGAGCGtGTCCAACATAGGAAcATTAAAGACCTTAAGCTGGCCTTCaGCGAGTTCTACCTCAGTCTCATCCTGCTGCAGAACGAACCAGAATTTTGCCACaAATATTCATATGGTGTGCGAGCCATTGTTCAATGTATTCCAGCTTGGCTTCGCTTCATCCAGTGCCTGCGCCGGTACCGTGACACGAGGAGGGCTTTTCCTCATCTAGTAAATGCTGGCAAATACTCcACAACTTTCTTCACcGTGACATTTGCAGCCCTTTACAGCACTCACAAACCTCCTCTTTCTCCAGAACAAAAaCACTCTGACACCgtGGTgTTCTTTTACCTGTCTATTACTGCTACAACGTTTAAGCCTCATGTTGGGGAC

>Fra-MC1_18-2

GAGAAGCTGGCAGAGGCTCAGCGCAGGTTTGCTACACTTCAGAATGAGCTTCAGTCATCGCTGGATGTGCAGAAAGAAAGCAGTGgTGTCACgaCAtTGCGACAgCGCAGAAAGCCAGTGTTCCACCTGTCCCACGAGGAGCGtGTCCAACATAGGAAcATTAAAGACCTTAAGCTGGCCTTCaGCGAGTTCTACCTCAGTCTCATCCTGCTGCAGAACGAACCAGAATTTTGCCACaAATATTCATATGGTGTGCGAGCCATTGTTCAATGTATTCCAGCTTGGCTTCGCTTCATCCAGTGCCTGCGCCGGTACCGTGACACGAGGAGGGCTTTTCCTCATCTAGTAAATGCTGGCAAATACTCcACAACTTTCTTCACcGTGACATTTGCAGCCCTTTACAGCACTCACAAACCTCCTCTTTCTCCAGAACAAAAaCACTCTGACACCgtGGTgTTCTTTTACCTGTCTATTACTGCTACAACGTTTAAGCCTCATGTTGGGGAC

>Fra-MC1_13-1

GAGAAGCTGGCAGAGGCTCAGCGCAGGTTTGCTACACTTCAGAATGAGCTTCAGTCATCGCTGGATGTGCAGAAAGAAAGCAGTGgTGTCACgaCAtTGCGACAgCGCAGAAAGCCAGTGTTCCACCTGTCCCACGAGGAGCGtGTCCAACATAGGAAcATTAAAGACCTTAAGCTGGCCTTCaGCGAGTTCTACCTCAGTCTCATCCTGCTGCAGAACGAACCAGAATTTTGCCACaAATATTCATATGGTGTGCGAGCCATTGTTCAATGTATTCCAGCTTGGCTTCGCTTCATCCAGTGCCTGCGCCGGTACCGTGACACGAGGAGGGCTTTTCCTCATCTAGTAAATGCTGGCAAATACTCcACAACTTTCTTCACcGTGACATTTGCAGCCCTTTACAGCACTCACAAACCTCCTCTTTCTCCAGAACAAAAaCACTCTGACACCgtGGTgTTCTTTTACCTGTCTATTACTGCTACAACGTTTAAGCCTCATGTTGGGGAC

>Fra-MC1_13-2

GAGAAGCTGGCAGAGGCTCAGCGCAGGTTTGCTACACTTCAGAATGAGCTTCAGTCATCGCTGGATGTGCAGAAAGAAAGCAGTGgTGTCACgaCAtTGCGACAgCGCAGAAAGCCAGTGTTCCACCTGTCCCACGAGGAGCGtGTCCAACATAGGAAcATTAAAGACCTTAAGCTGGCCTTCaGCGAGTTCTACCTCAGTCTCATCCTGCTGCAGAACGAACCAGAATTTTGCCACaAATATTCATATGGTGTGCGAGCCATTGTTCAATGTATTCCAGCTTGGCTTCGCTTCATCCAGTGCCTGCGCCGGTACCGTGACACGAGGAGGGCTTTTCCTCATCTAGTAAATGCTGGCAAATACTCcACAACTTTCTTCACtGTGACATTTGCAGCCCTTTACAGCACTCACAAACCTCCTCTTTCTCCAGAACAAAAaCACTCTGACACCgtGGTgTTCTTTTACCTGTCTATTACTGCTACAACGTTTAAGCCTCATGTTGGGGAC

>Fra-MC1_21-1

GAGAAGCTGGCAGAGGCTCAGCGCAGGTTTGCTACACTTCAGAATGAGCTTCAGTCATCGCTGGATGTGCAGAAAGAAAGCAGTGgTGTCACgaCAtTGCGACAgCGCAGAAAGCCAGTGTTCCACCTGTCCCACGAGGAGCGtGTCCAACATAGGAAcATTAAAGACCTTAAGCTGGCCTTCaGCGAGTTCTACCTCAGTCTCATCCTGCTGCAGAACGAACCAGAATTTTGCCACaAATATTCATATGGTGTGCGAGCCATTGTTCAATGTATTCCAGCTTGGCTTCGCTTCATCCAGTGCCTGCGCCGGTACCGTGACACGAGGAGGGCTTTTCCTCATCTAGTAAATGCTGGCAAATACTCcACAACTTTCTTCACcGTGACATTTGCAGCCCTTTACAGCACTCACAAACCTCCTCTTTCTCCAGAACAAAAaCACTCTGACACCgtGGTgTTCTTTTACCTGTCTATTACTGCTACAACGTTTAAGCCTCATGTTGGGGAC

>Fra-MC1_21-2

GAGAAGCTGGCAGAGGCTCAGCGCAGGTTTGCTACACTTCAGAATGAGCTTCAGTCATCGCTGGATGTGCAGAAAGAAAGCAGTGgTGTCACgaCAtTGCGACAgCGCAGAAAGCCAGTGTTCCACCTGTCCCACGAGGAGCGtGTCCAACATAGGAAcATTAAAGACCTTAAGCTGGCCTTCaGCGAGTTCTACCTCAGTCTCATCCTGCTGCAGAACGAACCAGAATTTTGCCACaAATATTCATATGGTGTGCGAGCCATTGTTCAATGTATTCCAGCTTGGCTTCGCTTCATCCAGTGCCTGCGCCGGTACCGTGACACGAGGAGGGCTTTTCCTCATCTAGTAAATGCTGGCAAATACTCcACAACTTTCTTCACcGTGACATTTGCAGCCCTTTACAGCACTCACAAACCTCCTCTTTCTCCAGAACAAAAaCACTCTGACACCgtGGTgTTCTTTTACCTGTCTATTACTGCTACAACGTTTAAGCCTCATGTTGGGGAC

>Fra-MC1_25-1

GAGAAGCTGGCAGAGGCTCAGCGCAGGTTTGCTACACTTCAGAATGAGCTTCAGTCATCGCTGGATGTGCAGAAAGAAAGCAGTGgTGTCACgaCAtTGCGACAgCGCAGAAAGCCAGTGTTCCACCTGTCCCACGAGGAGCGtGTCCAACATAGGAAcATTAAAGACCTTAAGCTGGCCTTCaGCGAGTTCTACCTCAGTCTCATCCTGCTGCAGAACGAACCAGAATTTTGCCACaAATATTCATATGGTGTGCGAGCCATTGTTCAATGTATTCCAGCTTGGCTTCGCTTCATCCAGTGCCTGCGCCGGTACCGTGACACGAGGAGGGCTTTTCCTCATCTAGTAAATGCTGGCAAATACTCcACAACTTTCTTCACcGTGACATTTGCAGCCCTTTACAGCACTCACAAACCTCCTCTTTCTCCAGAACAAAAaCACTCTGACACCgtGGTgTTCTTTTACCTGTCTATTACTGCTACAACGTTTAAGCCTCATGTTGGGGAC

>Fra-MC1_25-2

GAGAAGCTGGCAGAGGCTCAGCGCAGGTTTGCTACACTTCAGAATGAGCTTCAGTCATCGCTGGATGTGCAGAAAGAAAGCAGTGgTGTCACgaCAtTGCGACAgCGCAGAAAGCCAGTGTTCCACCTGTCCCACGAGGAGCGtGTCCAACATAGGAAcATTAAAGACCTTAAGCTGGCCTTCaGCGAGTTCTACCTCAGTCTCATCCTGCTGCAGAACGAACCAGAATTTTGCCACaAATATTCATATGGTGTGCGAGCCATTGTTCAATGTATTCCAGCTTGGCTTCGCTTCATCCAGTGCCTGCGCCGGTACCGTGACACGAGGAGGGCTTTTCCTCATCTAGTAAATGCTGGCAAATACTCcACAACTTTCTTCACcGTGACATTTGCAGCCCTTTACAGCACTCACAAACCTCCTCTTTCTCCAGAACAAAAaCACTCTGACACCgtGGTgTTCTTTTACCTGTCTATTACTGCTACAACGTTTAAGCCTCATGTTGGGGAC

>Fra-MC1_22-1

GAGAAGCTGGCAGAGGCTCAGCGCAGGTTTGCTACACTTCAGAATGAGCTTCAGTCATCGCTGGATGTGCAGAAAGAAAGCAGTGgTGTCACgaCAtTGCGACAgCGCAGAAAGCCAGTGTTCCACCTGTCCCACGAGGAGCGtGTCCAACATAGGAAcATTAAAGACCTTAAGCTGGCCTTCaGCGAGTTCTACCTCAGTCTCATCCTGCTGCAGAACGAACCAGAATTTTGCCACaAATATTCATATGGTGTGCGAGCCATTGTTCAATGTATTCCAGCTTGGCTTCGCTTCATCCAGTGCCTGCGCCGGTACCGTGACACGAGGAGGGCTTTTCCTCATCTAGTAAATGCTGGCAAATACTCcACAACTTTCTTCACcGTGACATTTGCAGCCCTTTACAGCACTCACAAACCTCCTCTTTCTCCAGAACAAAAaCACTCTGACACCgtGGTgTTCTTTTACCTGTCTATTACTGCTACAACGTTTAAGCCTCATGTTGGGGAC

>Fra-MC1_22-2

GAGAAGCTGGCAGAGGCTCAGCGCAGGTTTGCTACACTTCAGAATGAGCTTCAGTCATCGCTGGATGTGCAGAAAGAAAGCAGTGgTGTCACgaCAtTGCGACAgCGCAGAAAGCCAGTGTTCCACCTGTCCCACGAGGAGCGtGTCCAACATAGGAAcATTAAAGACCTTAAGCTGGCCTTCaGCGAGTTCTACCTCAGTCTCATCCTGCTGCAGAACGAACCAGAATTTTGCCACaAATATTCATATGGTGTGCGAGCCATTGTTCAATGTATTCCAGCTTGGCTTCGCTTCATCCAGTGCCTGCGCCGGTACCGTGACACGAGGAGGGCTTTTCCTCATCTAGTAAATGCTGGCAAATACTCcACAACTTTCTTCACcGTGACATTTGCAGCCCTTTACAGCACTCACAAACCTCCTCTTTCTCCAGAACAAAAaCACTCTGACACCgtGGTgTTCTTTTACCTGTCTATTACTGCTACAACGTTTAAGCCTCATGTTGGGGAC

>Fra-MC1_7-1

GAGAAGCTGGCAGAGGCTCAGCGCAGGTTTGCTACACTTCAGAATGAGCTTCAGTCATCGCTGGATGTGCAGAAAGAAAGCAGTGgTGTCACgaCAtTGCGACAgCGCAGAAAGCCAGTGTTCCACCTGTCCCACGAGGAGCGtGTCCAACATAGGAAcATTAAAGACCTTAAGCTGGCCTTCaGCGAGTTCTACCTCAGTCTCATCCTGCTGCAGAACGAACCAGAATTTTGCCACaAATATTCATATGGTGTGCGAGCCATTGTTCAATGTATTCCAGCTTGGCTTCGCTTCATCCAGTGCCTGCGCCGGTACCGTGACACGAGGAGGGCTTTTCCTCATCTAGTAAATGCTGGCAAATACTCcACAACTTTCTTCACtGTGACATTTGCAGCCCTTTACAGCACTCACAAACCTCCTCTTTCTCCAGAACAAAAaCACTCTGACACCgtGGTgTTCTTTTACCTGTCTATTACTGCTACAACGTTTAAGCCTCATGTTGGGGAC

>Fra-MC1_7-2

GAGAAGCTGGCAGAGGCTCAGCGCAGGTTTGCTACACTTCAGAATGAGCTTCAGTCATCGCTGGATGTGCAGAAAGAAAGCAGTGgTGTCACaaCAcTGCGACAgCGCAGAAAGCCAGTGTTCCACCTGTCCCACGAGGAGCGtGTCCAACATAGGAAcATTAAAGACCTTAAGCTGGCCTTCaGCGAGTTCTACCTCAGTCTCATCCTGCTGCAGAACGAACCAGAATTTTGCCACaAATATTCATATGGTGTGCGAGCCATTGTTCAATGTATTCCAGCTTGGCTTCGCTTCATCCAGTGCCTGCGCCGGTACCGTGACACGAGGAGGGCTTTTCCTCATCTAGTAAATGCTGGCAAATACTCcACAACTTTCTTCACcGTGACATTTGCAGCCCTTTACAGCACTCACAAACCTCCTCTTTCTCCAGAACAAAAtCACTCTGACACCgtGGTgTTCTTTTACCTGTCTATTACTGCTACAACGTTTAAGCCTCATGTTGGGGAC

>Fra-MC1_10-1

GAGAAGCTGGCAGAGGCTCAGCGCAGGTTTGCTACACTTCAGAATGAGCTTCAGTCATCGCTGGATGTGCAGAAAGAAAGCAGTGcTGTCACagCAtTGCGACAgCGCAGAAAGCCAGTGTTCCACCTGTCCCACGAGGAGCGtGTCCAACATAGGAAcATTAAAGACCTTAAGCTGGCCTTCaGCGAGTTCTACCTCAGTCTCATCCTGCTGCAGAACGAACCAGAATTTTGCCACaAATATTCATATGGTGTGCGAGCCATTGTTCAATGTATTCCAGCTTGGCTTCGCTTCATCCAGTGCCTGCGCCGGTACCGTGACACGAGGAGGGCTTTTCCTCATCTAGTAAATGCTGGCAAATACTCcACAACTTTCTTCACcGTGACATTTGCAGCCCTTTACAGCACTCACAAACCTCCTCTTTCTCCAGAACAAAAtCACTCTGACACCgtGGTgTTCTTTTACCTGTCTATTACTGCTACAACGTTTAAGCCTCATGTTGGGGAC

>Fra-MC1_10-2

GAGAAGCTGGCAGAGGCTCAGCGCAGGTTTGCTACACTTCAGAATGAGCTTCAGTCATCGCTGGATGTGCAGAAAGAAAGCAGTGcTGTCACagCAtTGCGACAgCGCAGAAAGCCAGTGTTCCACCTGTCCCACGAGGAGCGtGTCCAACATAGGAAcATTAAAGACCTTAAGCTGGCCTTCgGCGAGTTCTACCTCAGTCTCATCCTGCTGCAGAACGAACCAGAATTTTGCCACaAATATTCATATGGTGTGCGAGCCATTGTTCAATGTATTCCAGCTTGGCTTCGCTTCATCCAGTGCCTGCGCCGGTACCGTGACACGAGGAGGGCTTTTCCTCATCTAGTAAATGCTGGCAAATACTCcACAACTTTCTTCACcGTGACATTTGCAGCCCTTTACAGCACTCACAAACCTCCTCTTTCTCCAGAACAAAAtCACTCTGACACCgtGGTgTTCTTTTACCTGTCTATTACTGCTACAACGTTTAAGCCTCATGTTGGGGAC

>Fra-MC1_4-1

GAGAAGCTGGCAGAGGCTCAGCGCAGGTTTGCTACACTTCAGAATGAGCTTCAGTCATCGCTGGATGTGCAGAAAGAAAGCAGTGcTGTCACagCAtTGCGACAgCGCAGAAAGCCAGTGTTCCACCTGTCCCACGAGGAGCGtGTCCAACATAGGAAcATTAAAGACCTTAAGCTGGCCTTCaGCGAGTTCTACCTCAGTCTCATCCTGCTGCAGAACGAACCAGAATTTTGCCACaAATATTCATATGGTGTGCGAGCCATTGTTCAATGTATTCCAGCTTGGCTTCGCTTCATCCAGTGCCTGCGCCGGTACCGTGACACGAGGAGGGCTTTTCCTCATCTAGTAAATGCTGGCAAATACTCcACAACTTTCTTCACcGTGACATTTGCAGCCCTTTACAGCACTCACAAACCTCCTCTTTCTCCAGAACAAAAtCACTCTGACACCgtGGTgTTCTTTTACCTGTCTATTACTGCTACAACGTTTAAGCCTCATGTTGGGGAC

>Fra-MC1_4-2

GAGAAGCTGGCAGAGGCTCAGCGCAGGTTTGCTACACTTCAGAATGAGCTTCAGTCATCGCTGGATGTGCAGAAAGAAAGCAGTGcTGTCACagCAtTGCGACAgCGCAGAAAGCCAGTGTTCCACCTGTCCCACGAGGAGCGtGTCCAACATAGGAAcATTAAAGACCTTAAGCTGGCCTTCaGCGAGTTCTACCTCAGTCTCATCCTGCTGCAGAACGAACCAGAATTTTGCCACaAATATTCATATGGTGTGCGAGCCATTGTTCAATGTATTCCAGCTTGGCTTCGCTTCATCCAGTGCCTGCGCCGGTACCGTGACACGAGGAGGGCTTTTCCTCATCTAGTAAATGCTGGCAAATACTCcACAACTTTCTTCACcGTGACATTTGCAGCCCTTTACAGCACTCACAAACCTCCTCTTTCTCCAGAACAAAAtCACTCTGACACCgtGGTgTTCTTTTACCTGTCTATTACTGCTACAACGTTTAAGCCTCATGTTGGGGAC

>Fra-MC1_6-1

GAGAAGCTGGCAGAGGCTCAGCGCAGGTTTGCTACACTTCAGAATGAGCTTCAGTCATCGCTGGATGTGCAGAAAGAAAGCAGTGcTGTCACagCAtTGCGACAgCGCAGAAAGCCAGTGTTCCACCTGTCCCACGAGGAGCGtGTCCAACATAGGAAcATTAAAGACCTTAAGCTGGCCTTCaGCGAGTTCTACCTCAGTCTCATCCTGCTGCAGAACGAACCAGAATTTTGCCACaAATATTCATATGGTGTGCGAGCCATTGTTCAATGTATTCCAGCTTGGCTTCGCTTCATCCAGTGCCTGCGCCGGTACCGTGACACGAGGAGGGCTTTTCCTCATCTAGTAAATGCTGGCAAATACTCcACAACTTTCTTCACcGTGACATTTGCAGCCCTTTACAGCACTCACAAACCTCCTCTTTCTCCAGAACAAAAtCACTCTGACACCgtGGTgTTCTTTTACCTGTCTATTACTGCTACAACGTTTAAGCCTCATGTTGGGGAC

>Fra-MC1_6-2

GAGAAGCTGGCAGAGGCTCAGCGCAGGTTTGCTACACTTCAGAATGAGCTTCAGTCATCGCTGGATGTGCAGAAAGAAAGCAGTGcTGTCACagCAtTGCGACAgCGCAGAAAGCCAGTGTTCCACCTGTCCCACGAGGAGCGtGTCCAACATAGGAAcATTAAAGACCTTAAGCTGGCCTTCaGCGAGTTCTACCTCAGTCTCATCCTGCTGCAGAACGAACCAGAATTTTGCCACaAATATTCATATGGTGTGCGAGCCATTGTTCAATGTATTCCAGCTTGGCTTCGCTTCATCCAGTGCCTGCGCCGGTACCGTGACACGAGGAGGGCTTTTCCTCATCTAGTAAATGCTGGCAAATACTCcACAACTTTCTTCACcGTGACATTTGCAGCCCTTTACAGCACTCACAAACCTCCTCTTTCTCCAGAACAAAAtCACTCTGACACCgtGGTgTTCTTTTACCTGTCTATTACTGCTACAACGTTTAAGCCTCATGTTGGGGAC

>Fra-MC2_2101-1

GAGAAGCTGGCAGAGGCTCAGCGCAGGTTTGCTACACTTCAGAATGAGCTTCAGTCATCGCTGGATGTGCAGAAAGAAAGCAGTGgTGTCACgaCAtTGCGACAgCGCAGAAAGCCAGTGTTCCACCTGTCCCACGAGGAGCGtGTCCAACATAGGAAcATTAAAGACCTTAAGCTGGCCTTCaGCGAGTTCTACCTCAGTCTCATCCTGCTGCAGAACGAACCAGAATTTTGCCACaAATATTCATATGGTGTGCGAGCCATTGTTCAATGTATTCCAGCTTGGCTTCGCTTCATCCAGTGCCTGCGCCGGTACCGTGACACGAGGAGGGCTTTTCCTCATCTAGTAAATGCTGGCAAATACTCcACAACTTTCTTCACcGTGACATTTGCAGCCCTTTACAGCACTCACAAACCTCCTCTTTCTCCAGAACAAAAaCACTCTGACACCgtGGTgTTCTTTTACCTGTCTATTACTGCTACAACGTTTAAGCCTCATGTTGGGGAC

>Fra-MC2_2101-2

GAGAAGCTGGCAGAGGCTCAGCGCAGGTTTGCTACACTTCAGAATGAGCTTCAGTCATCGCTGGATGTGCAGAAAGAAAGCAGTGgTGTCACgaCAtTGCGACAgCGCAGAAAGCCAGTGTTCCACCTGTCCCACGAGGAGCGtGTCCAACATAGGAAcATTAAAGACCTTAAGCTGGCCTTCaGCGAGTTCTACCTCAGTCTCATCCTGCTGCAGAACGAACCAGAATTTTGCCACaAATATTCATATGGTGTGCGAGCCATTGTTCAATGTATTCCAGCTTGGCTTCGCTTCATCCAGTGCCTGCGCCGGTACCGTGACACGAGGAGGGCTTTTCCTCATCTAGTAAATGCTGGCAAATACTCcACAACTTTCTTCACcGTGACATTTGCAGCCCTTTACAGCACTCACAAACCTCCTCTTTCTCCAGAACAAAAaCACTCTGACACCgtGGTgTTCTTTTACCTGTCTATTACTGCTACAACGTTTAAGCCTCATGTTGGGGAC

>Fra-MC2_2301-1

GAGAAGCTGGCAGAGGCTCAGCGCAGGTTTGCTACACTTCAGAATGAGCTTCAGTCATCGCTGGATGTGCAGAAAGAAAGCAGTGgTGTCACgaCAtTGCGACAgCGCAGAAAGCCAGTGTTCCACCTGTCCCACGAGGAGCGtGTCCAACATAGGAAcATTAAAGACCTTAAGCTGGCCTTCaGCGAGTTCTACCTCAGTCTCATCCTGCTGCAGAACGAACCAGAATTTTGCCACaAATATTCATATGGTGTGCGAGCCATTGTTCAATGTATTCCAGCTTGGCTTCGCTTCATCCAGTGCCTGCGCCGGTACCGTGACACGAGGAGGGCTTTTCCTCATCTAGTAAATGCTGGCAAATACTCcACAACTTTCTTCACcGTGACATTTGCAGCCCTTTACAGCACTCACAAACCTCCTCTTTCTCCAGAACAAAAaCACTCTGACACCgtGGTgTTCTTTTACCTGTCTATTACTGCTACAACGTTTAAGCCTCATGTTGGGGAC

>Fra-MC2_2301-2

GAGAAGCTGGCAGAGGCTCAGCGCAGGTTTGCTACACTTCAGAATGAGCTTCAGTCATCGCTGGATGTGCAGAAAGAAAGCAGTGgTGTCACgaCAtTGCGACAgCGCAGAAAGCCAGTGTTCCACCTGTCCCACGAGGAGCGtGTCCAACATAGGAAcATTAAAGACCTTAAGCTGGCCTTCaGCGAGTTCTACCTCAGTCTCATCCTGCTGCAGAACGAACCAGAATTTTGCCACaAATATTCATATGGTGTGCGAGCCATTGTTCAATGTATTCCAGCTTGGCTTCGCTTCATCCAGTGCCTGCGCCGGTACCGTGACACGAGGAGGGCTTTTCCTCATCTAGTAAATGCTGGCAAATACTCcACAACTTTCTTCACcGTGACATTTGCAGCCCTTTACAGCACTCACAAACCTCCTCTTTCTCCAGAACAAAAaCACTCTGACACCgtGGTgTTCTTTTACCTGTCTATTACTGCTACAACGTTTAAGCCTCATGTTGGGGAC

>Fra-MC2_1501-1

GAGAAGCTGGCAGAGGCTCAGCGCAGGTTTGCTACACTTCAGAATGAGCTTCAGTCATCGCTGGATGTGCAGAAAGAAAGCAGTGgTGTCACgaCAtTGCGACAgCGCAGAAAGCCAGTGTTCCACCTGTCCCACGAGGAGCGtGTCCAACATAGGAAcATTAAAGACCTTAAGCTGGCCTTCaGCGAGTTCTACCTCAGTCTCATCCTGCTGCAGAACGAACCAGAATTTTGCCACaAATATTCATATGGTGTGCGAGCCATTGTTCAATGTATTCCAGCTTGGCTTCGCTTCATCCAGTGCCTGCGCCGGTACCGTGACACGAGGAGGGCTTTTCCTCATCTAGTAAATGCTGGCAAATACTCcACAACTTTCTTCACcGTGACATTTGCAGCCCTTTACAGCACTCACAAACCTCCTCTTTCTCCAGAACAAAAaCACTCTGACACCgtGGTgTTCTTTTACCTGTCTATTACTGCTACAACGTTTAAGCCTCATGTTGGGGAC

>Fra-MC2_1501-2

GAGAAGCTGGCAGAGGCTCAGCGCAGGTTTGCTACACTTCAGAATGAGCTTCAGTCATCGCTGGATGTGCAGAAAGAAAGCAGTGgTGTCACgaCAtTGCGACAgCGCAGAAAGCCAGTGTTCCACCTGTCCCACGAGGAGCGtGTCCAACATAGGAAcATTAAAGACCTTAAGCTGGCCTTCaGCGAGTTCTACCTCAGTCTCATCCTGCTGCAGAACGAACCAGAATTTTGCCACaAATATTCATATGGTGTGCGAGCCATTGTTCAATGTATTCCAGCTTGGCTTCGCTTCATCCAGTGCCTGCGCCGGTACCGTGACACGAGGAGGGCTTTTCCTCATCTAGTAAATGCTGGCAAATACTCcACAACTTTCTTCACcGTGACATTTGCAGCCCTTTACAGCACTCACAAACCTCCTCTTTCTCCAGAACAAAAaCACTCTGACACCgtGGTgTTCTTTTACCTGTCTATTACTGCTACAACGTTTAAGCCTCATGTTGGGGAC

>Fra-MC2_1201-1

GAGAAGCTGGCAGAGGCTCAGCGCAGGTTTGCTACACTTCAGAATGAGCTTCAGTCATCGCTGGATGTGCAGAAAGAAAGCAGTGgTGTCACgaCAtTGCGACAgCGCAGAAAGCCAGTGTTCCACCTGTCCCACGAGGAGCGtGTCCAACATAGGAAcATTAAAGACCTTAAGCTGGCCTTCaGCGAGTTCTACCTCAGTCTCATCCTGCTGCAGAACGAACCAGAATTTTGCCACaAATATTCATATGGTGTGCGAGCCATTGTTCAATGTATTCCAGCTTGGCTTCGCTTCATCCAGTGCCTGCGCCGGTACCGTGACACGAGGAGGGCTTTTCCTCATCTAGTAAATGCTGGCAAATACTCcACAACTTTCTTCACtGTGACATTTGCAGCCCTTTACAGCACTCACAAACCTCCTCTTTCTCCAGAACAAAAaCACTCTGACACCgtGGTgTTCTTTTACCTGTCTATTACTGCTACAACGTTTAAGCCTCATGTTGGGGAC

>Fra-MC2_1201-2

GAGAAGCTGGCAGAGGCTCAGCGCAGGTTTGCTACACTTCAGAATGAGCTTCAGTCATCGCTGGATGTGCAGAAAGAAAGCAGTGgTGTCACgaCAtTGCGACAgCGCAGAAAGCCAGTGTTCCACCTGTCCCACGAGGAGCGtGTCCAACATAGGAAcATTAAAGACCTTAAGCTGGCCTTCaGCGAGTTCTACCTCAGTCTCATCCTGCTGCAGAACGAACCAGAATTTTGCCACaAATATTCATATGGTGTGCGAGCCATTGTTCAATGTATTCCAGCTTGGCTTCGCTTCATCCAGTGCCTGCGCCGGTACCGTGACACGAGGAGGGCTTTTCCTCATCTAGTAAATGCTGGCAAATACTCcACAACTTTCTTCACcGTGACATTTGCAGCCCTTTACAGCACTCACAAACCTCCTCTTTCTCCAGAACAAAAaCACTCTGACACCgtGGTgTTCTTTTACCTGTCTATTACTGCTACAACGTTTAAGCCTCATGTTGGGGAC

>Fra-MC2_0301-1

GAGAAGCTGGCAGAGGCTCAGCGCAGGTTTGCTACACTTCAGAATGAGCTTCAGTCATCGCTGGATGTGCAGAAAGAAAGCAGTGgTGTCACgaCAtTGCGACAgCGCAGAAAGCCAGTGTTCCACCTGTCCCACGAGGAGCGtGTCCAACATAGGAAcATTAAAGACCTTAAGCTGGCCTTCaGCGAGTTCTACCTCAGTCTCATCCTGCTGCAGAACGAACCAGAATTTTGCCACaAATATTCATATGGTGTGCGAGCCATTGTTCAATGTATTCCAGCTTGGCTTCGCTTCATCCAGTGCCTGCGCCGGTACCGTGACACGAGGAGGGCTTTTCCTCATCTAGTAAATGCTGGCAAATACTCcACAACTTTCTTCACcGTGACATTTGCAGCCCTTTACAGCACTCACAAACCTCCTCTTTCTCCAGAACAAAAaCACTCTGACACCgtGGTgTTCTTTTACCTGTCTATTACTGCTACAACGTTTAAGCCTCATGTTGGGGAC

>Fra-MC2_0301-2

GAGAAGCTGGCAGAGGCTCAGCGCAGGTTTGCTACACTTCAGAATGAGCTTCAGTCATCGCTGGATGTGCAGAAAGAAAGCAGTGgTGTCACgaCAtTGCGACAgCGCAGAAAGCCAGTGTTCCACCTGTCCCACGAGGAGCGtGTCCAACATAGGAAcATTAAAGACCTTAAGCTGGCCTTCaGCGAGTTCTACCTCAGTCTCATCCTGCTGCAGAACGAACCAGAATTTTGCCACaAATATTCATATGGTGTGCGAGCCATTGTTCAATGTATTCCAGCTTGGCTTCGCTTCATCCAGTGCCTGCGCCGGTACCGTGACACGAGGAGGGCTTTTCCTCATCTAGTAAATGCTGGCAAATACTCcACAACTTTCTTCACcGTGACATTTGCAGCCCTTTACAGCACTCACAAACCTCCTCTTTCTCCAGAACAAAAaCACTCTGACACCgtGGTgTTCTTTTACCTGTCTATTACTGCTACAACGTTTAAGCCTCATGTTGGGGAC

>Fra-MC2_1301-1

GAGAAGCTGGCAGAGGCTCAGCGCAGGTTTGCTACACTTCAGAATGAGCTTCAGTCATCGCTGGATGTGCAGAAAGAAAGCAGTGgTGTCACaaCAcTGCGACAgCGCAGAAAGCCAGTGTTCCACCTGTCCCACGAGGAGCGtGTCCAACATAGGAAcATTAAAGACCTTAAGCTGGCCTTCaGCGAGTTCTACCTCAGTCTCATCCTGCTGCAGAACGAACCAGAATTTTGCCACaAATATTCATATGGTGTGCGAGCCATTGTTCAATGTATTCCAGCTTGGCTTCGCTTCATCCAGTGCCTGCGCCGGTACCGTGACACGAGGAGGGCTTTTCCTCATCTAGTAAATGCTGGCAAATACTCcACAACTTTCTTCACcGTGACATTTGCAGCCCTTTACAGCACTCACAAACCTCCTCTTTCTCCAGAACAAAAtCACTCTGACACCgtGGTgTTCTTTTACCTGTCTATTACTGCTACAACGTTTAAGCCTCATGTTGGGGAC

>Fra-MC2_1301-2

GAGAAGCTGGCAGAGGCTCAGCGCAGGTTTGCTACACTTCAGAATGAGCTTCAGTCATCGCTGGATGTGCAGAAAGAAAGCAGTGgTGTCACgaCAtTGCGACAgCGCAGAAAGCCAGTGTTCCACCTGTCCCACGAGGAGCGtGTCCAACATAGGAAcATTAAAGACCTTAAGCTGGCCTTCaGCGAGTTCTACCTCAGTCTCATCCTGCTGCAGAACGAACCAGAATTTTGCCACaAATATTCATATGGTGTGCGAGCCATTGTTCAATGTATTCCAGCTTGGCTTCGCTTCATCCAGTGCCTGCGCCGGTACCGTGACACGAGGAGGGCTTTTCCTCATCTAGTAAATGCTGGCAAATACTCcACAACTTTCTTCACcGTGACATTTGCAGCCCTTTACAGCACTCACAAACCTCCTCTTTCTCCAGAACAAAAaCACTCTGACACCgtGGTgTTCTTTTACCTGTCTATTACTGCTACAACGTTTAAGCCTCATGTTGGGGAC

>Fra-MC2_2201-1

GAGAAGCTGGCAGAGGCTCAGCGCAGGTTTGCTACACTTCAGAATGAGCTTCAGTCATCGCTGGATGTGCAGAAAGAAAGCAGTGgTGTCACaaCAcTGCGACAgCGCAGAAAGCCAGTGTTCCACCTGTCCCACGAGGAGCGtGTCCAACATAGGAAcATTAAAGACCTTAAGCTGGCCTTCaGCGAGTTCTACCTCAGTCTCATCCTGCTGCAGAACGAACCAGAATTTTGCCACaAATATTCATATGGTGTGCGAGCCATTGTTCAATGTATTCCAGCTTGGCTTCGCTTCATCCAGTGCCTGCGCCGGTACCGTGACACGAGGAGGGCTTTTCCTCATCTAGTAAATGCTGGCAAATACTCcACAACTTTCTTCACcGTGACATTTGCAGCCCTTTACAGCACTCACAAACCTCCTCTTTCTCCAGAACAAAAtCACTCTGACACCgtGGTgTTCTTTTACCTGTCTATTACTGCTACAACGTTTAAGCCTCATGTTGGGGAC

>Fra-MC2_2201-2

GAGAAGCTGGCAGAGGCTCAGCGCAGGTTTGCTACACTTCAGAATGAGCTTCAGTCATCGCTGGATGTGCAGAAAGAAAGCAGTGgTGTCACgaCAtTGCGACAgCGCAGAAAGCCAGTGTTCCACCTGTCCCACGAGGAGCGtGTCCAACATAGGAAcATTAAAGACCTTAAGCTGGCCTTCaGCGAGTTCTACCTCAGTCTCATCCTGCTGCAGAACGAACCAGAATTTTGCCACaAATATTCATATGGTGTGCGAGCCATTGTTCAATGTATTCCAGCTTGGCTTCGCTTCATCCAGTGCCTGCGCCGGTACCGTGACACGAGGAGGGCTTTTCCTCATCTAGTAAATGCTGGCAAATACTCcACAACTTTCTTCACcGTGACATTTGCAGCCCTTTACAGCACTCACAAACCTCCTCTTTCTCCAGAACAAAAaCACTCTGACACCgtGGTgTTCTTTTACCTGTCTATTACTGCTACAACGTTTAAGCCTCATGTTGGGGAC

>Fra-MC2_0201-1

GAGAAGCTGGCAGAGGCTCAGCGCAGGTTTGCTACACTTCAGAATGAGCTTCAGTCATCGCTGGATGTGCAGAAAGAAAGCAGTGgTGTCACgaCAtTGCGACAgCGCAGAAAGCCAGTGTTCCACCTGTCCCACGAGGAGCGtGTCCAACATAGGAAcATTAAAGACCTTAAGCTGGCCTTCaGCGAGTTCTACCTCAGTCTCATCCTGCTGCAGAACGAACCAGAATTTTGCCACaAATATTCATATGGTGTGCGAGCCATTGTTCAATGTATTCCAGCTTGGCTTCGCTTCATCCAGTGCCTGCGCCGGTACCGTGACACGAGGAGGGCTTTTCCTCATCTAGTAAATGCTGGCAAATACTCcACAACTTTCTTCACcGTGACATTTGCAGCCCTTTACAGCACTCACAAACCTCCTCTTTCTCCAGAACAAAAtCACTCTGACACCgtGGTgTTCTTTTACCTGTCTATTACTGCTACAACGTTTAAGCCTCATGTTGGGGAC

>Fra-MC2_0201-2

GAGAAGCTGGCAGAGGCTCAGCGCAGGTTTGCTACACTTCAGAATGAGCTTCAGTCATCGCTGGATGTGCAGAAAGAAAGCAGTGcTGTCACagCAtTGCGACAgCGCAGAAAGCCAGTGTTCCACCTGTCCCACGAGGAGCGtGTCCAACATAGGAAcATTAAAGACCTTAAGCTGGCCTTCaGCGAGTTCTACCTCAGTCTCATCCTGCTGCAGAACGAACCAGAATTTTGCCACaAATATTCATATGGTGTGCGAGCCATTGTTCAATGTATTCCAGCTTGGCTTCGCTTCATCCAGTGCCTGCGCCGGTACCGTGACACGAGGAGGGCTTTTCCTCATCTAGTAAATGCTGGCAAATACTCcACAACTTTCTTCACcGTGACATTTGCAGCCCTTTACAGCACTCACAAACCTCCTCTTTCTCCAGAACAAAAtCACTCTGACACCgtGGTgTTCTTTTACCTGTCTATTACTGCTACAACGTTTAAGCCTCATGTTGGGGAC

>Fra-MC2_2001-1

GAGAAGCTGGCAGAGGCTCAGCGCAGGTTTGCTACACTTCAGAATGAGCTTCAGTCATCGCTGGATGTGCAGAAAGAAAGCAGTGgTGTCACgaCAtTGCGACAgCGCAGAAAGCCAGTGTTCCACCTGTCCCACGAGGAGCGtGTCCAACATAGGAAcATTAAAGACCTTAAGCTGGCCTTCaGCGAGTTCTACCTCAGTCTCATCCTGCTGCAGAACGAACCAGAATTTTGCCACaAATATTCATATGGTGTGCGAGCCATTGTTCAATGTATTCCAGCTTGGCTTCGCTTCATCCAGTGCCTGCGCCGGTACCGTGACACGAGGAGGGCTTTTCCTCATCTAGTAAATGCTGGCAAATACTCcACAACTTTCTTCACcGTGACATTTGCAGCCCTTTACAGCACTCACAAACCTCCTCTTTCTCCAGAACAAAAaCACTCTGACACCgtGGTgTTCTTTTACCTGTCTATTACTGCTACAACGTTTAAGCCTCATGTTGGGGAC

>Fra-MC2_2001-2

GAGAAGCTGGCAGAGGCTCAGCGCAGGTTTGCTACACTTCAGAATGAGCTTCAGTCATCGCTGGATGTGCAGAAAGAAAGCAGTGcTGTCACagCAtTGCGACAgCGCAGAAAGCCAGTGTTCCACCTGTCCCACGAGGAGCGtGTCCAACATAGGAAcATTAAAGACCTTAAGCTGGCCTTCaGCGAGTTCTACCTCAGTCTCATCCTGCTGCAGAACGAACCAGAATTTTGCCACaAATATTCATATGGTGTGCGAGCCATTGTTCAATGTATTCCAGCTTGGCTTCGCTTCATCCAGTGCCTGCGCCGGTACCGTGACACGAGGAGGGCTTTTCCTCATCTAGTAAATGCTGGCAAATACTCcACAACTTTCTTCACcGTGACATTTGCAGCCCTTTACAGCACTCACAAACCTCCTCTTTCTCCAGAACAAAAaCACTCTGACACCgtGGTgTTCTTTTACCTGTCTATTACTGCTACAACGTTTAAGCCTCATGTTGGGGAC

>Fra-MC2_0601-1

GAGAAGCTGGCAGAGGCTCAGCGCAGGTTTGCTACACTTCAGAATGAGCTTCAGTCATCGCTGGATGTGCAGAAAGAAAGCAGTGgTGTCACaaCAcTGCGACAgCGCAGAAAGCCAGTGTTCCACCTGTCCCACGAGGAGCGtGTCCAACATAGGAAcATTAAAGACCTTAAGCTGGCCTTCaGCGAGTTCTACCTCAGTCTCATCCTGCTGCAGAACGAACCAGAATTTTGCCACaAATATTCATATGGTGTGCGAGCCATTGTTCAATGTATTCCAGCTTGGCTTCGCTTCATCCAGTGCCTGCGCCGGTACCGTGACACGAGGAGGGCTTTTCCTCATCTAGTAAATGCTGGCAAATACTCcACAACTTTCTTCACcGTGACATTTGCAGCCCTTTACAGCACTCACAAACCTCCTCTTTCTCCAGAACAAAAtCACTCTGACACCgtGGTgTTCTTTTACCTGTCTATTACTGCTACAACGTTTAAGCCTCATGTTGGGGAC

>Fra-MC2_0601-2

GAGAAGCTGGCAGAGGCTCAGCGCAGGTTTGCTACACTTCAGAATGAGCTTCAGTCATCGCTGGATGTGCAGAAAGAAAGCAGTGcTGTCACagCAtTGCGACAgCGCAGAAAGCCAGTGTTCCACCTGTCCCACGAGGAGCGtGTCCAACATAGGAAcATTAAAGACCTTAAGCTGGCCTTCaGCGAGTTCTACCTCAGTCTCATCCTGCTGCAGAACGAACCAGAATTTTGCCACaAATATTCATATGGTGTGCGAGCCATTGTTCAATGTATTCCAGCTTGGCTTCGCTTCATCCAGTGCCTGCGCCGGTACCGTGACACGAGGAGGGCTTTTCCTCATCTAGTAAATGCTGGCAAATACTCcACAACTTTCTTCACcGTGACATTTGCAGCCCTTTACAGCACTCACAAACCTCCTCTTTCTCCAGAACAAAAtCACTCTGACACCgtGGTgTTCTTTTACCTGTCTATTACTGCTACAACGTTTAAGCCTCATGTTGGGGAC

>Fra-MC2_1101-1

GAGAAGCTGGCAGAGGCTCAGCGCAGGTTTGCTACACTTCAGAATGAGCTTCAGTCATCGCTGGATGTGCAGAAAGAAAGCAGTGgTGTCACaaCAcTGCGACAgCGCAGAAAGCCAGTGTTCCACCTGTCCCACGAGGAGCGtGTCCAACATAGGAAcATTAAAGACCTTAAGCTGGCCTTCaGCGAGTTCTACCTCAGTCTCATCCTGCTGCAGAACGAACCAGAATTTTGCCACaAATATTCATATGGTGTGCGAGCCATTGTTCAATGTATTCCAGCTTGGCTTCGCTTCATCCAGTGCCTGCGCCGGTACCGTGACACGAGGAGGGCTTTTCCTCATCTAGTAAATGCTGGCAAATACTCcACAACTTTCTTCACcGTGACATTTGCAGCCCTTTACAGCACTCACAAACCTCCTCTTTCTCCAGAACAAAAtCACTCTGACACCgtGGTgTTCTTTTACCTGTCTATTACTGCTACAACGTTTAAGCCTCATGTTGGGGAC

>Fra-MC2_1101-2

GAGAAGCTGGCAGAGGCTCAGCGCAGGTTTGCTACACTTCAGAATGAGCTTCAGTCATCGCTGGATGTGCAGAAAGAAAGCAGTGcTGTCACagCAtTGCGACAgCGCAGAAAGCCAGTGTTCCACCTGTCCCACGAGGAGCGtGTCCAACATAGGAAcATTAAAGACCTTAAGCTGGCCTTCaGCGAGTTCTACCTCAGTCTCATCCTGCTGCAGAACGAACCAGAATTTTGCCACaAATATTCATATGGTGTGCGAGCCATTGTTCAATGTATTCCAGCTTGGCTTCGCTTCATCCAGTGCCTGCGCCGGTACCGTGACACGAGGAGGGCTTTTCCTCATCTAGTAAATGCTGGCAAATACTCcACAACTTTCTTCACcGTGACATTTGCAGCCCTTTACAGCACTCACAAACCTCCTCTTTCTCCAGAACAAAAtCACTCTGACACCgtGGTgTTCTTTTACCTGTCTATTACTGCTACAACGTTTAAGCCTCATGTTGGGGAC

>Fra-NA141-1

GAGAAGCTGGCAGAGGCTCAGCGCAGGTTTGCTACACTTCAGAATGAGCTTCAGTCATCGCTGGATGTGCAGAAAGAAAGCAGTGcTGTCACagCAtTGCGACAgCGCAGAAAGCCAGTGTTCCACCTGTCCCACGAGGAGCGtGTCCAACATAGGAAcATTAAAGACCTTAAGCTGGCCTTCaGCGAGTTCTACCTCAGTCTCATCCTGCTGCAGAACGAACCAGAATTTTGCCACaAATATTCATATGGTGTGCGAGCCATTGTTCAATGTATTCCAGCTTGGCTTCGCTTCATCCAGTGCCTGCGCCGGTACCGTGACACGAGGAGGGCTTTTCCTCATCTAGTAAATGCTGGCAAATACTCcACAACTTTCTTCACcGTGACATTTGCAGCCCTTTACAGCACTCACAAACCTCCTCTTTCTCCAGAACAAAAtCACTCTGACACCgtGGTgTTCTTTTACCTGTCTATTACTGCTACAACGTTTAAGCCTCATGTTGGGGAC

>Fra-NA141-2

GAGAAGCTGGCAGAGGCTCAGCGCAGGTTTGCTACACTTCAGAATGAGCTTCAGTCATCGCTGGATGTGCAGAAAGAAAGCAGTGcTGTCACagCAtTGCGACAgCGCAGAAAGCCAGTGTTCCACCTGTCCCACGAGGAGCGtGTCCAACATAGGAAcATTAAAGACCTTAAGCTGGCCTTCaGCGAGTTCTACCTCAGTCTCATCCTGCTGCAGAACGAACCAGAATTTTGCCACaAATATTCATATGGTGTGCGAGCCATTGTTCAATGTATTCCAGCTTGGCTTCGCTTCATCCAGTGCCTGCGCCGGTACCGTGACACGAGGAGGGCTTTTCCTCATCTAGTAAATGCTGGCAAATACTCcACAACTTTCTTCACcGTGACATTTGCAGCCCTTTACAGCACTCACAAACCTCCTCTTTCTCCAGAACAAAAtCACTCTGACACCgtGGTgTTCTTTTACCTGTCTATTACTGCTACAACGTTTAAGCCTCATGTTGGGGAC

>Fra-NA31-1

GAGAAGCTGGCAGAGGCTCAGCGCAGGTTTGCTACACTTCAGAATGAGCTTCAGTCATCGCTGGATGTGCAGAAAGAAAGCAGTGcTGTCACagCAtTGCGACAgCGCAGAAAGCCAGTGTTCCACCTGTCCCACGAGGAGCGtGTCCAACATAGGAAcATTAAAGACCTTAAGCTGGCCTTCaGCGAGTTCTACCTCAGTCTCATCCTGCTGCAGAACGAACCAGAATTTTGCCACaAATATTCATATGGTGTGCGAGCCATTGTTCAATGTATTCCAGCTTGGCTTCGCTTCATCCAGTGCCTGCGCCGGTACCGTGACACGAGGAGGGCTTTTCCTCATCTAGTAAATGCTGGCAAATACTCcACAACTTTCTTCACcGTGACATTTGCAGCCCTTTACAGCACTCACAAACCTCCTCTTTCTCCAGAACAAAAtCACTCTGACACCgtGGTgTTCTTTTACCTGTCTATTACTGCTACAACGTTTAAGCCTCATGTTGGGGAC

>Fra-NA31-2

GAGAAGCTGGCAGAGGCTCAGCGCAGGTTTGCTACACTTCAGAATGAGCTTCAGTCATCGCTGGATGTGCAGAAAGAAAGCAGTGcTGTCACagCAtTGCGACAgCGCAGAAAGCCAGTGTTCCACCTGTCCCACGAGGAGCGtGTCCAACATAGGAAcATTAAAGACCTTAAGCTGGCCTTCaGCGAGTTCTACCTCAGTCTCATCCTGCTGCAGAACGAACCAGAATTTTGCCACaAATATTCATATGGTGTGCGAGCCATTGTTCAATGTATTCCAGCTTGGCTTCGCTTCATCCAGTGCCTGCGCCGGTACCGTGACACGAGGAGGGCTTTTCCTCATCTAGTAAATGCTGGCAAATACTCcACAACTTTCTTCACcGTGACATTTGCAGCCCTTTACAGCACTCACAAACCTCCTCTTTCTCCAGAACAAAAtCACTCTGACACCgtGGTgTTCTTTTACCTGTCTATTACTGCTACAACGTTTAAGCCTCATGTTGGGGAC

>Fra-NA61-1

GAGAAGCTGGCAGAGGCTCAGCGCAGGTTTGCTACACTTCAGAATGAGCTTCAGTCATCGCTGGATGTGCAGAAAGAAAGCAGTGcTGTCACagCAtTGCGACAgCGCAGAAAGCCAGTGTTCCACCTGTCCCACGAGGAGCGtGTCCAACATAGGAAcATTAAAGACCTTAAGCTGGCCTTCaGCGAGTTCTACCTCAGTCTCATCCTGCTGCAGAACGAACCAGAATTTTGCCACaAATATTCATATGGTGTGCGAGCCATTGTTCAATGTATTCCAGCTTGGCTTCGCTTCATCCAGTGCCTGCGCCGGTACCGTGACACGAGGAGGGCTTTTCCTCATCTAGTAAATGCTGGCAAATACTCcACAACTTTCTTCACcGTGACATTTGCAGCCCTTTACAGCACTCACAAACCTCCTCTTTCTCCAGAACAAAAtCACTCTGACACCgtGGTgTTCTTTTACCTGTCTATTACTGCTACAACGTTTAAGCCTCATGTTGGGGAC

>Fra-NA61-2

GAGAAGCTGGCAGAGGCTCAGCGCAGGTTTGCTACACTTCAGAATGAGCTTCAGTCATCGCTGGATGTGCAGAAAGAAAGCAGTGcTGTCACagCAtTGCGACAgCGCAGAAAGCCAGTGTTCCACCTGTCCCACGAGGAGCGtGTCCAACATAGGAAcATTAAAGACCTTAAGCTGGCCTTCaGCGAGTTCTACCTCAGTCTCATCCTGCTGCAGAACGAACCAGAATTTTGCCACaAATATTCATATGGTGTGCGAGCCATTGTTCAATGTATTCCAGCTTGGCTTCGCTTCATCCAGTGCCTGCGCCGGTACCGTGACACGAGGAGGGCTTTTCCTCATCTAGTAAATGCTGGCAAATACTCcACAACTTTCTTCACcGTGACATTTGCAGCCCTTTACAGCACTCACAAACCTCCTCTTTCTCCAGAACAAAAtCACTCTGACACCgtGGTgTTCTTTTACCTGTCTATTACTGCTACAACGTTTAAGCCTCATGTTGGGGAC

>Fra-NA21-1

GAGAAGCTGGCAGAGGCTCAGCGCAGGTTTGCTACACTTCAGAATGAGCTTCAGTCATCGCTGGATGTGCAGAAAGAAAGCAGTGcTGTCACagCAtTGCGACAgCGCAGAAAGCCAGTGTTCCACCTGTCCCACGAGGAGCGtGTCCAACATAGGAAcATTAAAGACCTTAAGCTGGCCTTCaGCGAGTTCTACCTCAGTCTCATCCTGCTGCAGAACGAACCAGAATTTTGCCACaAATATTCATATGGTGTGCGAGCCATTGTTCAATGTATTCCAGCTTGGCTTCGCTTCATCCAGTGCCTGCGCCGGTACCGTGACACGAGGAGGGCTTTTCCTCATCTAGTAAATGCTGGCAAATACTCcACAACTTTCTTCACcGTGACATTTGCAGCCCTTTACAGCACTCACAAACCTCCTCTTTCTCCAGAACAAAAtCACTCTGACACCgtGGTgTTCTTTTACCTGTCTATTACTGCTACAACGTTTAAGCCTCATGTTGGGGAC

>Fra-NA21-2

GAGAAGCTGGCAGAGGCTCAGCGCAGGTTTGCTACACTTCAGAATGAGCTTCAGTCATCGCTGGATGTGCAGAAAGAAAGCAGTGcTGTCACagCAtTGCGACAgCGCAGAAAGCCAGTGTTCCACCTGTCCCACGAGGAGCGtGTCCAACATAGGAAcATTAAAGACCTTAAGCTGGCCTTCaGCGAGTTCTACCTCAGTCTCATCCTGCTGCAGAACGAACCAGAATTTTGCCACaAATATTCATATGGTGTGCGAGCCATTGTTCAATGTATTCCAGCTTGGCTTCGCTTCATCCAGTGCCTGCGCCGGTACCGTGACACGAGGAGGGCTTTTCCTCATCTAGTAAATGCTGGCAAATACTCcACAACTTTCTTCACcGTGACATTTGCAGCCCTTTACAGCACTCACAAACCTCCTCTTTCTCCAGAACAAAAtCACTCTGACACCgtGGTgTTCTTTTACCTGTCTATTACTGCTACAACGTTTAAGCCTCATGTTGGGGAC

>Fra-NA131-1

GAGAAGCTGGCAGAGGCTCAGCGCAGGTTTGCTACACTTCAGAATGAGCTTCAGTCATCGCTGGATGTGCAGAAAGAAAGCAGTGcTGTCACagCAtTGCGACAgCGCAGAAAGCCAGTGTTCCACCTGTCCCACGAGGAGCGtGTCCAACATAGGAAcATTAAAGACCTTAAGCTGGCCTTCaGCGAGTTCTACCTCAGTCTCATCCTGCTGCAGAACGAACCAGAATTTTGCCACaAATATTCATATGGTGTGCGAGCCATTGTTCAATGTATTCCAGCTTGGCTTCGCTTCATCCAGTGCCTGCGCCGGTACCGTGACACGAGGAGGGCTTTTCCTCATCTAGTAAATGCTGGCAAATACTCcACAACTTTCTTCACcGTGACATTTGCAGCCCTTTACAGCACTCACAAACCTCCTCTTTCTCCAGAACAAAAtCACTCTGACACCgtGGTgTTCTTTTACCTGTCTATTACTGCTACAACGTTTAAGCCTCATGTTGGGGAC

>Fra-NA131-2

GAGAAGCTGGCAGAGGCTCAGCGCAGGTTTGCTACACTTCAGAATGAGCTTCAGTCATCGCTGGATGTGCAGAAAGAAAGCAGTGcTGTCACagCAtTGCGACAgCGCAGAAAGCCAGTGTTCCACCTGTCCCACGAGGAGCGtGTCCAACATAGGAAcATTAAAGACCTTAAGCTGGCCTTCaGCGAGTTCTACCTCAGTCTCATCCTGCTGCAGAACGAACCAGAATTTTGCCACaAATATTCATATGGTGTGCGAGCCATTGTTCAATGTATTCCAGCTTGGCTTCGCTTCATCCAGTGCCTGCGCCGGTACCGTGACACGAGGAGGGCTTTTCCTCATCTAGTAAATGCTGGCAAATACTCcACAACTTTCTTCACcGTGACATTTGCAGCCCTTTACAGCACTCACAAACCTCCTCTTTCTCCAGAACAAAAtCACTCTGACACCgtGGTgTTCTTTTACCTGTCTATTACTGCTACAACGTTTAAGCCTCATGTTGGGGAC

>Fra-NA101-1

GAGAAGCTGGCAGAGGCTCAGCGCAGGTTTGCTACACTTCAGAATGAGCTTCAGTCATCGCTGGATGTGCAGAAAGAAAGCAGTGcTGTCACagCAtTGCGACAgCGCAGAAAGCCAGTGTTCCACCTGTCCCACGAGGAGCGtGTCCAACATAGGAAcATTAAAGACCTTAAGCTGGCCTTCaGCGAGTTCTACCTCAGTCTCATCCTGCTGCAGAACGAACCAGAATTTTGCCACaAATATTCATATGGTGTGCGAGCCATTGTTCAATGTATTCCAGCTTGGCTTCGCTTCATCCAGTGCCTGCGCCGGTACCGTGACACGAGGAGGGCTTTTCCTCATCTAGTAAATGCTGGCAAATACTCcACAACTTTCTTCACcGTGACATTTGCAGCCCTTTACAGCACTCACAAACCTCCTCTTTCTCCAGAACAAAAtCACTCTGACACCgtGGTgTTCTTTTACCTGTCTATTACTGCTACAACGTTTAAGCCTCATGTTGGGGAC

>Fra-NA101-2

GAGAAGCTGGCAGAGGCTCAGCGCAGGTTTGCTACACTTCAGAATGAGCTTCAGTCATCGCTGGATGTGCAGAAAGAAAGCAGTGcTGTCACagCAtTGCGACAgCGCAGAAAGCCAGTGTTCCACCTGTCCCACGAGGAGCGtGTCCAACATAGGAAcATTAAAGACCTTAAGCTGGCCTTCaGCGAGTTCTACCTCAGTCTCATCCTGCTGCAGAACGAACCAGAATTTTGCCACaAATATTCATATGGTGTGCGAGCCATTGTTCAATGTATTCCAGCTTGGCTTCGCTTCATCCAGTGCCTGCGCCGGTACCGTGACACGAGGAGGGCTTTTCCTCATCTAGTAAATGCTGGCAAATACTCcACAACTTTCTTCACcGTGACATTTGCAGCCCTTTACAGCACTCACAAACCTCCTCTTTCTCCAGAACAAAAtCACTCTGACACCgtGGTgTTCTTTTACCTGTCTATTACTGCTACAACGTTTAAGCCTCATGTTGGGGAC

>Fra-NA111-1

GAGAAGCTGGCAGAGGCTCAGCGCAGGTTTGCTACACTTCAGAATGAGCTTCAGTCATCGCTGGATGTGCAGAAAGAAAGCAGTGcTGTCACagCAtTGCGACAgCGCAGAAAGCCAGTGTTCCACCTGTCCCACGAGGAGCGtGTCCAACATAGGAAcATTAAAGACCTTAAGCTGGCCTTCaGCGAGTTCTACCTCAGTCTCATCCTGCTGCAGAACGAACCAGAATTTTGCCACaAATATTCATATGGTGTGCGAGCCATTGTTCAATGTATTCCAGCTTGGCTTCGCTTCATCCAGTGCCTGCGCCGGTACCGTGACACGAGGAGGGCTTTTCCTCATCTAGTAAATGCTGGCAAATACTCcACAACTTTCTTCACcGTGACATTTGCAGCCCTTTACAGCACTCACAAACCTCCTCTTTCTCCAGAACAAAAtCACTCTGACACCgtGGTgTTCTTTTACCTGTCTATTACTGCTACAACGTTTAAGCCTCATGTTGGGGAC

>Fra-NA111-2

GAGAAGCTGGCAGAGGCTCAGCGCAGGTTTGCTACACTTCAGAATGAGCTTCAGTCATCGCTGGATGTGCAGAAAGAAAGCAGTGgTGTCACaaCAcTGCGACAgCGCAGAAAGCCAGTGTTCCACCTGTCCCACGAGGAGCGtGTCCAACATAGGAAcATTAAAGACCTTAAGCTGGCCTTCaGCGAGTTCTACCTCAGTCTCATCCTGCTGCAGAACGAACCAGAATTTTGCCACaAATATTCATATGGTGTGCGAGCCATTGTTCAATGTATTCCAGCTTGGCTTCGCTTCATCCAGTGCCTGCGCCGGTACCGTGACACGAGGAGGGCTTTTCCTCATCTAGTAAATGCTGGCAAATACTCcACAACTTTCTTCACcGTGACATTTGCAGCCCTTTACAGCACTCACAAACCTCCTCTTTCTCCAGAACAAAAtCACTCTGACACCgtGGTgTTCTTTTACCTGTCTATTACTGCTACAACGTTTAAGCCTCATGTTGGGGAC

>Fra-NA121-1

GAGAAGCTGGCAGAGGCTCAGCGCAGGTTTGCTACACTTCAGAATGAGCTTCAGTCATCGCTGGATGTGCAGAAAGAAAGCAGTGcTGTCACagCAtTGCGACAgCGCAGAAAGCCAGTGTTCCACCTGTCCCACGAGGAGCGtGTCCAACATAGGAAcATTAAAGACCTTAAGCTGGCCTTCaGCGAGTTCTACCTCAGTCTCATCCTGCTGCAGAACGAACCAGAATTTTGCCACaAATATTCATATGGTGTGCGAGCCATTGTTCAATGTATTCCAGCTTGGCTTCGCTTCATCCAGTGCCTGCGCCGGTACCGTGACACGAGGAGGGCTTTTCCTCATCTAGTAAATGCTGGCAAATACTCcACAACTTTCTTCACcGTGACATTTGCAGCCCTTTACAGCACTCACAAACCTCCTCTTTCTCCAGAACAAAAtCACTCTGACACCgtGGTgTTCTTTTACCTGTCTATTACTGCTACAACGTTTAAGCCTCATGTTGGGGAC

>Fra-NA121-2

GAGAAGCTGGCAGAGGCTCAGCGCAGGTTTGCTACACTTCAGAATGAGCTTCAGTCATCGCTGGATGTGCAGAAAGAAAGCAGTGgTGTCACaaCAcTGCGACAgCGCAGAAAGCCAGTGTTCCACCTGTCCCACGAGGAGCGtGTCCAACATAGGAAcATTAAAGACCTTAAGCTGGCCTTCaGCGAGTTCTACCTCAGTCTCATCCTGCTGCAGAACGAACCAGAATTTTGCCACaAATATTCATATGGTGTGCGAGCCATTGTTCAATGTATTCCAGCTTGGCTTCGCTTCATCCAGTGCCTGCGCCGGTACCGTGACACGAGGAGGGCTTTTCCTCATCTAGTAAATGCTGGCAAATACTCcACAACTTTCTTCACcGTGACATTTGCAGCCCTTTACAGCACTCACAAACCTCCTCTTTCTCCAGAACAAAAtCACTCTGACACCgtGGTgTTCTTTTACCTGTCTATTACTGCTACAACGTTTAAGCCTCATGTTGGGGAC

>Fra-NA41-1

GAGAAGCTGGCAGAGGCTCAGCGCAGGTTTGCTACACTTCAGAATGAGCTTCAGTCATCGCTGGATGTGCAGAAAGAAAGCAGTGcTGTCACagCAtTGCGACAgCGCAGAAAGCCAGTGTTCCACCTGTCCCACGAGGAGCGtGTCCAACATAGGAAcATTAAAGACCTTAAGCTGGCCTTCaGCGAGTTCTACCTCAGTCTCATCCTGCTGCAGAACGAACCAGAATTTTGCCACaAATATTCATATGGTGTGCGAGCCATTGTTCAATGTATTCCAGCTTGGCTTCGCTTCATCCAGTGCCTGCGCCGGTACCGTGACACGAGGAGGGCTTTTCCTCATCTAGTAAATGCTGGCAAATACTCcACAACTTTCTTCACcGTGACATTTGCAGCCCTTTACAGCACTCACAAACCTCCTCTTTCTCCAGAACAAAAtCACTCTGACACCgtGGTgTTCTTTTACCTGTCTATTACTGCTACAACGTTTAAGCCTCATGTTGGGGAC

>Fra-NA41-2

GAGAAGCTGGCAGAGGCTCAGCGCAGGTTTGCTACACTTCAGAATGAGCTTCAGTCATCGCTGGATGTGCAGAAAGAAAGCAGTGgTGTCACaaCAcTGCGACAgCGCAGAAAGCCAGTGTTCCACCTGTCCCACGAGGAGCGtGTCCAACATAGGAAcATTAAAGACCTTAAGCTGGCCTTCaGCGAGTTCTACCTCAGTCTCATCCTGCTGCAGAACGAACCAGAATTTTGCCACaAATATTCATATGGTGTGCGAGCCATTGTTCAATGTATTCCAGCTTGGCTTCGCTTCATCCAGTGCCTGCGCCGGTACCGTGACACGAGGAGGGCTTTTCCTCATCTAGTAAATGCTGGCAAATACTCcACAACTTTCTTCACcGTGACATTTGCAGCCCTTTACAGCACTCACAAACCTCCTCTTTCTCCAGAACAAAAtCACTCTGACACCgtGGTgTTCTTTTACCTGTCTATTACTGCTACAACGTTTAAGCCTCATGTTGGGGAC

>Fra-NA71-1

GAGAAGCTGGCAGAGGCTCAGCGCAGGTTTGCTACACTTCAGAATGAGCTTCAGTCATCGCTGGATGTGCAGAAAGAAAGCAGTGcTGTCACagCAtTGCGACAgCGCAGAAAGCCAGTGTTCCACCTGTCCCACGAGGAGCGtGTCCAACATAGGAAcATTAAAGACCTTAAGCTGGCCTTCaGCGAGTTCTACCTCAGTCTCATCCTGCTGCAGAACGAACCAGAATTTTGCCACaAATATTCATATGGTGTGCGAGCCATTGTTCAATGTATTCCAGCTTGGCTTCGCTTCATCCAGTGCCTGCGCCGGTACCGTGACACGAGGAGGGCTTTTCCTCATCTAGTAAATGCTGGCAAATACTCcACAACTTTCTTCACcGTGACATTTGCAGCCCTTTACAGCACTCACAAACCTCCTCTTTCTCCAGAACAAAAtCACTCTGACACCgtGGTgTTCTTTTACCTGTCTATTACTGCTACAACGTTTAAGCCTCATGTTGGGGAC

>Fra-NA71-2

GAGAAGCTGGCAGAGGCTCAGCGCAGGTTTGCTACACTTCAGAATGAGCTTCAGTCATCGCTGGATGTGCAGAAAGAAAGCAGTGgTGTCACaaCAcTGCGACAgCGCAGAAAGCCAGTGTTCCACCTGTCCCACGAGGAGCGtGTCCAACATAGGAAcATTAAAGACCTTAAGCTGGCCTTCaGCGAGTTCTACCTCAGTCTCATCCTGCTGCAGAACGAACCAGAATTTTGCCACaAATATTCATATGGTGTGCGAGCCATTGTTCAATGTATTCCAGCTTGGCTTCGCTTCATCCAGTGCCTGCGCCGGTACCGTGACACGAGGAGGGCTTTTCCTCATCTAGTAAATGCTGGCAAATACTCcACAACTTTCTTCACcGTGACATTTGCAGCCCTTTACAGCACTCACAAACCTCCTCTTTCTCCAGAACAAAAtCACTCTGACACCgtGGTgTTCTTTTACCTGTCTATTACTGCTACAACGTTTAAGCCTCATGTTGGGGAC

>Fra-NA92-1

GAGAAGCTGGCAGAGGCTCAGCGCAGGTTTGCTACACTTCAGAATGAGCTTCAGTCATCGCTGGATGTGCAGAAAGAAAGCAGTGcTGTCACagCAtTGCGACAgCGCAGAAAGCCAGTGTTCCACCTGTCCCACGAGGAGCGtGTCCAACATAGGAAcATTAAAGACCTTAAGCTGGCCTTCaGCGAGTTCTACCTCAGTCTCATCCTGCTGCAGAACGAACCAGAATTTTGCCACaAATATTCATATGGTGTGCGAGCCATTGTTCAATGTATTCCAGCTTGGCTTCGCTTCATCCAGTGCCTGCGCCGGTACCGTGACACGAGGAGGGCTTTTCCTCATCTAGTAAATGCTGGCAAATACTCcACAACTTTCTTCACcGTGACATTTGCAGCCCTTTACAGCACTCACAAACCTCCTCTTTCTCCAGAACAAAAtCACTCTGACACCgtGGTgTTCTTTTACCTGTCTATTACTGCTACAACGTTTAAGCCTCATGTTGGGGAC

>Fra-NA92-2

GAGAAGCTGGCAGAGGCTCAGCGCAGGTTTGCTACACTTCAGAATGAGCTTCAGTCATCGCTGGATGTGCAGAAAGAAAGCAGTGgTGTCACaaCAcTGCGACAgCGCAGAAAGCCAGTGTTCCACCTGTCCCACGAGGAGCGtGTCCAACATAGGAAcATTAAAGACCTTAAGCTGGCCTTCaGCGAGTTCTACCTCAGTCTCATCCTGCTGCAGAACGAACCAGAATTTTGCCACaAATATTCATATGGTGTGCGAGCCATTGTTCAATGTATTCCAGCTTGGCTTCGCTTCATCCAGTGCCTGCGCCGGTACCGTGACACGAGGAGGGCTTTTCCTCATCTAGTAAATGCTGGCAAATACTCcACAACTTTCTTCACcGTGACATTTGCAGCCCTTTACAGCACTCACAAACCTCCTCTTTCTCCAGAACAAAAtCACTCTGACACCgtGGTgTTCTTTTACCTGTCTATTACTGCTACAACGTTTAAGCCTCATGTTGGGGAC

>Fra-NA81-1

GAGAAGCTGGCAGAGGCTCAGCGCAGGTTTGCTACACTTCAGAATGAGCTTCAGTCATCGCTGGATGTGCAGAAAGAAAGCAGTGgTGTCACaaCAcTGCGACAgCGCAGAAAGCCAGTGTTCCACCTGTCCCACGAGGAGCGtGTCCAACATAGGAAcATTAAAGACCTTAAGCTGGCCTTCaGCGAGTTCTACCTCAGTCTCATCCTGCTGCAGAACGAACCAGAATTTTGCCACaAATATTCATATGGTGTGCGAGCCATTGTTCAATGTATTCCAGCTTGGCTTCGCTTCATCCAGTGCCTGCGCCGGTACCGTGACACGAGGAGGGCTTTTCCTCATCTAGTAAATGCTGGCAAATACTCcACAACTTTCTTCACcGTGACATTTGCAGCCCTTTACAGCACTCACAAACCTCCTCTTTCTCCAGAACAAAAtCACTCTGACACCgtGGTgTTCTTTTACCTGTCTATTACTGCTACAACGTTTAAGCCTCATGTTGGGGAC

>Fra-NA81-2

GAGAAGCTGGCAGAGGCTCAGCGCAGGTTTGCTACACTTCAGAATGAGCTTCAGTCATCGCTGGATGTGCAGAAAGAAAGCAGTGgTGTCACaaCAcTGCGACAgCGCAGAAAGCCAGTGTTCCACCTGTCCCACGAGGAGCGtGTCCAACATAGGAAcATTAAAGACCTTAAGCTGGCCTTCaGCGAGTTCTACCTCAGTCTCATCCTGCTGCAGAACGAACCAGAATTTTGCCACaAATATTCATATGGTGTGCGAGCCATTGTTCAATGTATTCCAGCTTGGCTTCGCTTCATCCAGTGCCTGCGCCGGTACCGTGACACGAGGAGGGCTTTTCCTCATCTAGTAAATGCTGGCAAATACTCcACAACTTTCTTCACcGTGACATTTGCAGCCCTTTACAGCACTCACAAACCTCCTCTTTCTCCAGAACAAAAtCACTCTGACACCgtGGTgTTCTTTTACCTGTCTATTACTGCTACAACGTTTAAGCCTCATGTTGGGGAC

>Ger-SL11-1

GAGAAGCTGGCAGAGGCTCAGCGCAGGTTTGCTACACTTCAGAATGAGCTTCAGTCATCGCTGGATGTGCAGAAAGAAAGCAGTGcTGTCACagCAtTGCGACAgCGCAGAAAGCCAGTGTTCCACCTGTCCCACGAGGAGCGtGTCCAACATAGGAAcATTAAAGACCTTAAGCTGGCCTTCaGCGAGTTCTACCTCAGTCTCATCCTGCTGCAGAACGAACCAGAATTTTGCCACaAATATTCATATGGTGTGCGAGCCATTGTTCAATGTATTCCAGCTTGGCTTCGCTTCATCCAGTGCCTGCGCCGGTACCGTGACACGAGGAGGGCTTTTCCTCATCTAGTAAATGCTGGCAAATACTCcACAACTTTCTTCACcGTGACATTTGCAGCCCTTTACAGCACTCACAAACCTCCTCTTTCTCCAGAACAAAAtCACTCTGACACCgtGGTgTTCTTTTACCTGTCTATTACTGCTACAACGTTTAAGCCTCATGTTGGGGAC

>Ger-SL11-2

GAGAAGCTGGCAGAGGCTCAGCGCAGGTTTGCTACACTTCAGAATGAGCTTCAGTCATCGCTGGATGTGCAGAAAGAAAGCAGTGcTGTCACagCAtTGCGACAgCGCAGAAAGCCAGTGTTCCACCTGTCCCACGAGGAGCGtGTCCAACATAGGAAcATTAAAGACCTTAAGCTGGCCTTCaGCGAGTTCTACCTCAGTCTCATCCTGCTGCAGAACGAACCAGAATTTTGCCACaAATATTCATATGGTGTGCGAGCCATTGTTCAATGTATTCCAGCTTGGCTTCGCTTCATCCAGTGCCTGCGCCGGTACCGTGACACGAGGAGGGCTTTTCCTCATCTAGTAAATGCTGGCAAATACTCcACAACTTTCTTCACcGTGACATTTGCAGCCCTTTACAGCACTCACAAACCTCCTCTTTCTCCAGAACAAAAtCACTCTGACACCgtGGTgTTCTTTTACCTGTCTATTACTGCTACAACGTTTAAGCCTCATGTTGGGGAC

>Ger-SL3-1

GAGAAGCTGGCAGAGGCTCAGCGCAGGTTTGCTACACTTCAGAATGAGCTTCAGTCATCGCTGGATGTGCAGAAAGAAAGCAGTGcTGTCACagCAtTGCGACAgCGCAGAAAGCCAGTGTTCCACCTGTCCCACGAGGAGCGtGTCCAACATAGGAAcATTAAAGACCTTAAGCTGGCCTTCaGCGAGTTCTACCTCAGTCTCATCCTGCTGCAGAACGAACCAGAATTTTGCCACaAATATTCATATGGTGTGCGAGCCATTGTTCAATGTATTCCAGCTTGGCTTCGCTTCATCCAGTGCCTGCGCCGGTACCGTGACACGAGGAGGGCTTTTCCTCATCTAGTAAATGCTGGCAAATACTCcACAACTTTCTTCACcGTGACATTTGCAGCCCTTTACAGCACTCACAAACCTCCTCTTTCTCCAGAACAAAAtCACTCTGACACCgtGGTgTTCTTTTACCTGTCTATTACTGCTACAACGTTTAAGCCTCATGTTGGGGAC

>Ger-SL3-2

GAGAAGCTGGCAGAGGCTCAGCGCAGGTTTGCTACACTTCAGAATGAGCTTCAGTCATCGCTGGATGTGCAGAAAGAAAGCAGTGcTGTCACagCAtTGCGACAgCGCAGAAAGCCAGTGTTCCACCTGTCCCACGAGGAGCGtGTCCAACATAGGAAcATTAAAGACCTTAAGCTGGCCTTCaGCGAGTTCTACCTCAGTCTCATCCTGCTGCAGAACGAACCAGAATTTTGCCACaAATATTCATATGGTGTGCGAGCCATTGTTCAATGTATTCCAGCTTGGCTTCGCTTCATCCAGTGCCTGCGCCGGTACCGTGACACGAGGAGGGCTTTTCCTCATCTAGTAAATGCTGGCAAATACTCcACAACTTTCTTCACcGTGACATTTGCAGCCCTTTACAGCACTCACAAACCTCCTCTTTCTCCAGAACAAAAtCACTCTGACACCgtGGTgTTCTTTTACCTGTCTATTACTGCTACAACGTTTAAGCCTCATGTTGGGGAC

>Ger-SL91-1

GAGAAGCTGGCAGAGGCTCAGCGCAGGTTTGCTACACTTCAGAATGAGCTTCAGTCATCGCTGGATGTGCAGAAAGAAAGCAGTGcTGTCACagCAtTGCGACAgCGCAGAAAGCCAGTGTTCCACCTGTCCCACGAGGAGCGtGTCCAACATAGGAAcATTAAAGACCTTAAGCTGGCCTTCaGCGAGTTCTACCTCAGTCTCATCCTGCTGCAGAACGAACCAGAATTTTGCCACaAATATTCATATGGTGTGCGAGCCATTGTTCAATGTATTCCAGCTTGGCTTCGCTTCATCCAGTGCCTGCGCCGGTACCGTGACACGAGGAGGGCTTTTCCTCATCTAGTAAATGCTGGCAAATACTCcACAACTTTCTTCACcGTGACATTTGCAGCCCTTTACAGCACTCACAAACCTCCTCTTTCTCCAGAACAAAAtCACTCTGACACCgtGGTgTTCTTTTACCTGTCTATTACTGCTACAACGTTTAAGCCTCATGTTGGGGAC

>Ger-SL91-2

GAGAAGCTGGCAGAGGCTCAGCGCAGGTTTGCTACACTTCAGAATGAGCTTCAGTCATCGCTGGATGTGCAGAAAGAAAGCAGTGcTGTCACagCAtTGCGACAgCGCAGAAAGCCAGTGTTCCACCTGTCCCACGAGGAGCGtGTCCAACATAGGAAcATTAAAGACCTTAAGCTGGCCTTCaGCGAGTTCTACCTCAGTCTCATCCTGCTGCAGAACGAACCAGAATTTTGCCACaAATATTCATATGGTGTGCGAGCCATTGTTCAATGTATTCCAGCTTGGCTTCGCTTCATCCAGTGCCTGCGCCGGTACCGTGACACGAGGAGGGCTTTTCCTCATCTAGTAAATGCTGGCAAATACTCcACAACTTTCTTCACcGTGACATTTGCAGCCCTTTACAGCACTCACAAACCTCCTCTTTCTCCAGAACAAAAtCACTCTGACACCgtGGTgTTCTTTTACCTGTCTATTACTGCTACAACGTTTAAGCCTCATGTTGGGGAC

>Ger-SL181-1

GAGAAGCTGGCAGAGGCTCAGCGCAGGTTTGCTACACTTCAGAATGAGCTTCAGTCATCGCTGGATGTGCAGAAAGAAAGCAGTGcTGTCACagCAtTGCGACAgCGCAGAAAGCCAGTGTTCCACCTGTCCCACGAGGAGCGtGTCCAACATAGGAAcATTAAAGACCTTAAGCTGGCCTTCaGCGAGTTCTACCTCAGTCTCATCCTGCTGCAGAACGAACCAGAATTTTGCCACaAATATTCATATGGTGTGCGAGCCATTGTTCAATGTATTCCAGCTTGGCTTCGCTTCATCCAGTGCCTGCGCCGGTACCGTGACACGAGGAGGGCTTTTCCTCATCTAGTAAATGCTGGCAAATACTCcACAACTTTCTTCACcGTGACATTTGCAGCCCTTTACAGCACTCACAAACCTCCTCTTTCTCCAGAACAAAAtCACTCTGACACCgtGGTgTTCTTTTACCTGTCTATTACTGCTACAACGTTTAAGCCTCATGTTGGGGAC

>Ger-SL181-2

GAGAAGCTGGCAGAGGCTCAGCGCAGGTTTGCTACACTTCAGAATGAGCTTCAGTCATCGCTGGATGTGCAGAAAGAAAGCAGTGcTGTCACagCAtTGCGACAgCGCAGAAAGCCAGTGTTCCACCTGTCCCACGAGGAGCGtGTCCAACATAGGAAcATTAAAGACCTTAAGCTGGCCTTCaGCGAGTTCTACCTCAGTCTCATCCTGCTGCAGAACGAACCAGAATTTTGCCACaAATATTCATATGGTGTGCGAGCCATTGTTCAATGTATTCCAGCTTGGCTTCGCTTCATCCAGTGCCTGCGCCGGTACCGTGACACGAGGAGGGCTTTTCCTCATCTAGTAAATGCTGGCAAATACTCcACAACTTTCTTCACcGTGACATTTGCAGCCCTTTACAGCACTCACAAACCTCCTCTTTCTCCAGAACAAAAtCACTCTGACACCgtGGTgTTCTTTTACCTGTCTATTACTGCTACAACGTTTAAGCCTCATGTTGGGGAC

>Ger-SL51-1

GAGAAGCTGGCAGAGGCTCAGCGCAGGTTTGCTACACTTCAGAATGAGCTTCAGTCATCGCTGGATGTGCAGAAAGAAAGCAGTGcTGTCACagCAtTGCGACAgCGCAGAAAGCCAGTGTTCCACCTGTCCCACGAGGAGCGtGTCCAACATAGGAAcATTAAAGACCTTAAGCTGGCCTTCaGCGAGTTCTACCTCAGTCTCATCCTGCTGCAGAACGAACCAGAATTTTGCCACaAATATTCATATGGTGTGCGAGCCATTGTTCAATGTATTCCAGCTTGGCTTCGCTTCATCCAGTGCCTGCGCCGGTACCGTGACACGAGGAGGGCTTTTCCTCATCTAGTAAATGCTGGCAAATACTCcACAACTTTCTTCACcGTGACATTTGCAGCCCTTTACAGCACTCACAAACCTCCTCTTTCTCCAGAACAAAAtCACTCTGACACCgtGGTgTTCTTTTACCTGTCTATTACTGCTACAACGTTTAAGCCTCATGTTGGGGAC

>Ger-SL51-2

GAGAAGCTGGCAGAGGCTCAGCGCAGGTTTGCTACACTTCAGAATGAGCTTCAGTCATCGCTGGATGTGCAGAAAGAAAGCAGTGcTGTCACagCAtTGCGACAgCGCAGAAAGCCAGTGTTCCACCTGTCCCACGAGGAGCGtGTCCAACATAGGAAcATTAAAGACCTTAAGCTGGCCTTCaGCGAGTTCTACCTCAGTCTCATCCTGCTGCAGAACGAACCAGAATTTTGCCACaAATATTCATATGGTGTGCGAGCCATTGTTCAATGTATTCCAGCTTGGCTTCGCTTCATCCAGTGCCTGCGCCGGTACCGTGACACGAGGAGGGCTTTTCCTCATCTAGTAAATGCTGGCAAATACTCcACAACTTTCTTCACcGTGACATTTGCAGCCCTTTACAGCACTCACAAACCTCCTCTTTCTCCAGAACAAAAtCACTCTGACACCgtGGTgTTCTTTTACCTGTCTATTACTGCTACAACGTTTAAGCCTCATGTTGGGGAC

>Ger-SL41-1

GAGAAGCTGGCAGAGGCTCAGCGCAGGTTTGCTACACTTCAGAATGAGCTTCAGTCATCGCTGGATGTGCAGAAAGAAAGCAGTGcTGTCACagCAtTGCGACAgCGCAGAAAGCCAGTGTTCCACCTGTCCCACGAGGAGCGtGTCCAACATAGGAAcATTAAAGACCTTAAGCTGGCCTTCaGCGAGTTCTACCTCAGTCTCATCCTGCTGCAGAACGAACCAGAATTTTGCCACaAATATTCATATGGTGTGCGAGCCATTGTTCAATGTATTCCAGCTTGGCTTCGCTTCATCCAGTGCCTGCGCCGGTACCGTGACACGAGGAGGGCTTTTCCTCATCTAGTAAATGCTGGCAAATACTCcACAACTTTCTTCACcGTGACATTTGCAGCCCTTTACAGCACTCACAAACCTCCTCTTTCTCCAGAACAAAAtCACTCTGACACCgtGGTgTTCTTTTACCTGTCTATTACTGCTACAACGTTTAAGCCTCATGTTGGGGAC

>Ger-SL41-2

GAGAAGCTGGCAGAGGCTCAGCGCAGGTTTGCTACACTTCAGAATGAGCTTCAGTCATCGCTGGATGTGCAGAAAGAAAGCAGTGcTGTCACagCAtTGCGACAgCGCAGAAAGCCAGTGTTCCACCTGTCCCACGAGGAGCGtGTCCAACATAGGAAcATTAAAGACCTTAAGCTGGCCTTCaGCGAGTTCTACCTCAGTCTCATCCTGCTGCAGAACGAACCAGAATTTTGCCACaAATATTCATATGGTGTGCGAGCCATTGTTCAATGTATTCCAGCTTGGCTTCGCTTCATCCAGTGCCTGCGCCGGTACCGTGACACGAGGAGGGCTTTTCCTCATCTAGTAAATGCTGGCAAATACTCcACAACTTTCTTCACcGTGACATTTGCAGCCCTTTACAGCACTCACAAACCTCCTCTTTCTCCAGAACAAAAtCACTCTGACACCgtGGTgTTCTTTTACCTGTCTATTACTGCTACAACGTTTAAGCCTCATGTTGGGGAC

>Ger-SL171-1

GAGAAGCTGGCAGAGGCTCAGCGCAGGTTTGCTACACTTCAGAATGAGCTTCAGTCATCGCTGGATGTGCAGAAAGAAAGCAGTGcTGTCACagCAtTGCGACAgCGCAGAAAGCCAGTGTTCCACCTGTCCCACGAGGAGCGtGTCCAACATAGGAAcATTAAAGACCTTAAGCTGGCCTTCaGCGAGTTCTACCTCAGTCTCATCCTGCTGCAGAACGAACCAGAATTTTGCCACaAATATTCATATGGTGTGCGAGCCATTGTTCAATGTATTCCAGCTTGGCTTCGCTTCATCCAGTGCCTGCGCCGGTACCGTGACACGAGGAGGGCTTTTCCTCATCTAGTAAATGCTGGCAAATACTCcACAACTTTCTTCACcGTGACATTTGCAGCCCTTTACAGCACTCACAAACCTCCTCTTTCTCCAGAACAAAAtCACTCTGACACCgtGGTgTTCTTTTACCTGTCTATTACTGCTACAACGTTTAAGCCTCATGTTGGGGAC

>Ger-SL171-2

GAGAAGCTGGCAGAGGCTCAGCGCAGGTTTGCTACACTTCAGAATGAGCTTCAGTCATCGCTGGATGTGCAGAAAGAAAGCAGTGcTGTCACagCAtTGCGACAgCGCAGAAAGCCAGTGTTCCACCTGTCCCACGAGGAGCGtGTCCAACATAGGAAcATTAAAGACCTTAAGCTGGCCTTCaGCGAGTTCTACCTCAGTCTCATCCTGCTGCAGAACGAACCAGAATTTTGCCACaAATATTCATATGGTGTGCGAGCCATTGTTCAATGTATTCCAGCTTGGCTTCGCTTCATCCAGTGCCTGCGCCGGTACCGTGACACGAGGAGGGCTTTTCCTCATCTAGTAAATGCTGGCAAATACTCcACAACTTTCTTCACcGTGACATTTGCAGCCCTTTACAGCACTCACAAACCTCCTCTTTCTCCAGAACAAAAtCACTCTGACACCgtGGTgTTCTTTTACCTGTCTATTACTGCTACAACGTTTAAGCCTCATGTTGGGGAC

>Ger-SL151-1

GAGAAGCTGGCAGAGGCTCAGCGCAGGTTTGCTACACTTCAGAATGAGCTTCAGTCATCGCTGGATGTGCAGAAAGAAAGCAGTGcTGTCACagCAtTGCGACAgCGCAGAAAGCCAGTGTTCCACCTGTCCCACGAGGAGCGtGTCCAACATAGGAAcATTAAAGACCTTAAGCTGGCCTTCaGCGAGTTCTACCTCAGTCTCATCCTGCTGCAGAACGAACCAGAATTTTGCCACaAATATTCATATGGTGTGCGAGCCATTGTTCAATGTATTCCAGCTTGGCTTCGCTTCATCCAGTGCCTGCGCCGGTACCGTGACACGAGGAGGGCTTTTCCTCATCTAGTAAATGCTGGCAAATACTCcACAACTTTCTTCACcGTGACATTTGCAGCCCTTTACAGCACTCACAAACCTCCTCTTTCTCCAGAACAAAAtCACTCTGACACCgtGGTgTTCTTTTACCTGTCTATTACTGCTACAACGTTTAAGCCTCATGTTGGGGAC

>Ger-SL151-2

GAGAAGCTGGCAGAGGCTCAGCGCAGGTTTGCTACACTTCAGAATGAGCTTCAGTCATCGCTGGATGTGCAGAAAGAAAGCAGTGcTGTCACagCAtTGCGACAgCGCAGAAAGCCAGTGTTCCACCTGTCCCACGAGGAGCGtGTCCAACATAGGAAcATTAAAGACCTTAAGCTGGCCTTCaGCGAGTTCTACCTCAGTCTCATCCTGCTGCAGAACGAACCAGAATTTTGCCACaAATATTCATATGGTGTGCGAGCCATTGTTCAATGTATTCCAGCTTGGCTTCGCTTCATCCAGTGCCTGCGCCGGTACCGTGACACGAGGAGGGCTTTTCCTCATCTAGTAAATGCTGGCAAATACTCcACAACTTTCTTCACcGTGACATTTGCAGCCCTTTACAGCACTCACAAACCTCCTCTTTCTCCAGAACAAAAtCACTCTGACACCgtGGTgTTCTTTTACCTGTCTATTACTGCTACAACGTTTAAGCCTCATGTTGGGGAC

>Ger-SL131-1

GAGAAGCTGGCAGAGGCTCAGCGCAGGTTTGCTACACTTCAGAATGAGCTTCAGTCATCGCTGGATGTGCAGAAAGAAAGCAGTGcTGTCACagCAtTGCGACAgCGCAGAAAGCCAGTGTTCCACCTGTCCCACGAGGAGCGtGTCCAACATAGGAAcATTAAAGACCTTAAGCTGGCCTTCaGCGAGTTCTACCTCAGTCTCATCCTGCTGCAGAACGAACCAGAATTTTGCCACaAATATTCATATGGTGTGCGAGCCATTGTTCAATGTATTCCAGCTTGGCTTCGCTTCATCCAGTGCCTGCGCCGGTACCGTGACACGAGGAGGGCTTTTCCTCATCTAGTAAATGCTGGCAAATACTCcACAACTTTCTTCACcGTGACATTTGCAGCCCTTTACAGCACTCACAAACCTCCTCTTTCTCCAGAACAAAAtCACTCTGACACCgtGGTgTTCTTTTACCTGTCTATTACTGCTACAACGTTTAAGCCTCATGTTGGGGAC

>Ger-SL131-2

GAGAAGCTGGCAGAGGCTCAGCGCAGGTTTGCTACACTTCAGAATGAGCTTCAGTCATCGCTGGATGTGCAGAAAGAAAGCAGTGcTGTCACagCAtTGCGACAgCGCAGAAAGCCAGTGTTCCACCTGTCCCACGAGGAGCGtGTCCAACATAGGAAcATTAAAGACCTTAAGCTGGCCTTCaGCGAGTTCTACCTCAGTCTCATCCTGCTGCAGAACGAACCAGAATTTTGCCACaAATATTCATATGGTGTGCGAGCCATTGTTCAATGTATTCCAGCTTGGCTTCGCTTCATCCAGTGCCTGCGCCGGTACCGTGACACGAGGAGGGCTTTTCCTCATCTAGTAAATGCTGGCAAATACTCcACAACTTTCTTCACcGTGACATTTGCAGCCCTTTACAGCACTCACAAACCTCCTCTTTCTCCAGAACAAAAtCACTCTGACACCgtGGTgTTCTTTTACCTGTCTATTACTGCTACAACGTTTAAGCCTCATGTTGGGGAC

>Ger-SL141-1

GAGAAGCTGGCAGAGGCTCAGCGCAGGTTTGCTACACTTCAGAATGAGCTTCAGTCATCGCTGGATGTGCAGAAAGAAAGCAGTGcTGTCACagCAtTGCGACAgCGCAGAAAGCCAGTGTTCCACCTGTCCCACGAGGAGCGtGTCCAACATAGGAAcATTAAAGACCTTAAGCTGGCCTTCaGCGAGTTCTACCTCAGTCTCATCCTGCTGCAGAACGAACCAGAATTTTGCCACaAATATTCATATGGTGTGCGAGCCATTGTTCAATGTATTCCAGCTTGGCTTCGCTTCATCCAGTGCCTGCGCCGGTACCGTGACACGAGGAGGGCTTTTCCTCATCTAGTAAATGCTGGCAAATACTCcACAACTTTCTTCACcGTGACATTTGCAGCCCTTTACAGCACTCACAAACCTCCTCTTTCTCCAGAACAAAAtCACTCTGACACCgtGGTgTTCTTTTACCTGTCTATTACTGCTACAACGTTTAAGCCTCATGTTGGGGAC

>Ger-SL141-2

GAGAAGCTGGCAGAGGCTCAGCGCAGGTTTGCTACACTTCAGAATGAGCTTCAGTCATCGCTGGATGTGCAGAAAGAAAGCAGTGgTGTCACaaCAcTGCGACAgCGCAGAAAGCCAGTGTTCCACCTGTCCCACGAGGAGCGtGTCCAACATAGGAAcATTAAAGACCTTAAGCTGGCCTTCaGCGAGTTCTACCTCAGTCTCATCCTGCTGCAGAACGAACCAGAATTTTGCCACaAATATTCATATGGTGTGCGAGCCATTGTTCAATGTATTCCAGCTTGGCTTCGCTTCATCCAGTGCCTGCGCCGGTACCGTGACACGAGGAGGGCTTTTCCTCATCTAGTAAATGCTGGCAAATACTCcACAACTTTCTTCACcGTGACATTTGCAGCCCTTTACAGCACTCACAAACCTCCTCTTTCTCCAGAACAAAAtCACTCTGACACCgtGGTgTTCTTTTACCTGTCTATTACTGCTACAACGTTTAAGCCTCATGTTGGGGAC

>Ger-SL83-1

GAGAAGCTGGCAGAGGCTCAGCGCAGGTTTGCTACACTTCAGAATGAGCTTCAGTCATCGCTGGATGTGCAGAAAGAAAGCAGTGcTGTCACagCAtTGCGACAgCGCAGAAAGCCAGTGTTCCACCTGTCCCACGAGGAGCGtGTCCAACATAGGAAcATTAAAGACCTTAAGCTGGCCTTCaGCGAGTTCTACCTCAGTCTCATCCTGCTGCAGAACGAACCAGAATTTTGCCACaAATATTCATATGGTGTGCGAGCCATTGTTCAATGTATTCCAGCTTGGCTTCGCTTCATCCAGTGCCTGCGCCGGTACCGTGACACGAGGAGGGCTTTTCCTCATCTAGTAAATGCTGGCAAATACTCcACAACTTTCTTCACcGTGACATTTGCAGCCCTTTACAGCACTCACAAACCTCCTCTTTCTCCAGAACAAAAtCACTCTGACACCgtGGTgTTCTTTTACCTGTCTATTACTGCTACAACGTTTAAGCCTCATGTTGGGGAC

>Ger-SL83-2

GAGAAGCTGGCAGAGGCTCAGCGCAGGTTTGCTACACTTCAGAATGAGCTTCAGTCATCGCTGGATGTGCAGAAAGAAAGCAGTGgTGTCACaaCAcTGCGACAgCGCAGAAAGCCAGTGTTCCACCTGTCCCACGAGGAGCGtGTCCAACATAGGAAcATTAAAGACCTTAAGCTGGCCTTCaGCGAGTTCTACCTCAGTCTCATCCTGCTGCAGAACGAACCAGAATTTTGCCACaAATATTCATATGGTGTGCGAGCCATTGTTCAATGTATTCCAGCTTGGCTTCGCTTCATCCAGTGCCTGCGCCGGTACCGTGACACGAGGAGGGCTTTTCCTCATCTAGTAAATGCTGGCAAATACTCcACAACTTTCTTCACcGTGACATTTGCAGCCCTTTACAGCACTCACAAACCTCCTCTTTCTCCAGAACAAAAtCACTCTGACACCgtGGTgTTCTTTTACCTGTCTATTACTGCTACAACGTTTAAGCCTCATGTTGGGGAC

>Ger-SL16-1

GAGAAGCTGGCAGAGGCTCAGCGCAGGTTTGCTACACTTCAGAATGAGCTTCAGTCATCGCTGGATGTGCAGAAAGAAAGCAGTGgTGTCACaaCAcTGCGACAgCGCAGAAAGCCAGTGTTCCACCTGTCCCACGAGGAGCGtGTCCAACATAGGAAcATTAAAGACCTTAAGCTGGCCTTCaGCGAGTTCTACCTCAGTCTCATCCTGCTGCAGAACGAACCAGAATTTTGCCACaAATATTCATATGGTGTGCGAGCCATTGTTCAATGTATTCCAGCTTGGCTTCGCTTCATCCAGTGCCTGCGCCGGTACCGTGACACGAGGAGGGCTTTTCCTCATCTAGTAAATGCTGGCAAATACTCcACAACTTTCTTCACcGTGACATTTGCAGCCCTTTACAGCACTCACAAACCTCCTCTTTCTCCAGAACAAAAtCACTCTGACACCgtGGTgTTCTTTTACCTGTCTATTACTGCTACAACGTTTAAGCCTCATGTTGGGGAC

>Ger-SL16-2

GAGAAGCTGGCAGAGGCTCAGCGCAGGTTTGCTACACTTCAGAATGAGCTTCAGTCATCGCTGGATGTGCAGAAAGAAAGCAGTGgTGTCACaaCAcTGCGACAgCGCAGAAAGCCAGTGTTCCACCTGTCCCACGAGGAGCGtGTCCAACATAGGAAcATTAAAGACCTTAAGCTGGCCTTCaGCGAGTTCTACCTCAGTCTCATCCTGCTGCAGAACGAACCAGAATTTTGCCACaAATATTCATATGGTGTGCGAGCCATTGTTCAATGTATTCCAGCTTGGCTTCGCTTCATCCAGTGCCTGCGCCGGTACCGTGACACGAGGAGGGCTTTTCCTCATCTAGTAAATGCTGGCAAATACTCcACAACTTTCTTCACcGTGACATTTGCAGCCCTTTACAGCACTCACAAACCTCCTCTTTCTCCAGAACAAAAtCACTCTGACACCgtGGTgTTCTTTTACCTGTCTATTACTGCTACAACGTTTAAGCCTCATGTTGGGGAC

>Dom-IR21-1

GAGAAGCTGGCAGAGGCTCAGCGCAGGTTTGCTACACTTCAGAATGAGCTTCAGTCATCGCTGGATGTGCAGAAAGAAAGCAGTGgTGTCACgaCAtTGCGACAgCGCAGAAAGCCAGTGTTCCACCTGTCCCACGAGGAGCGtGTCCAACATAGGAAcATTAAAGACCTTAAGCTGGCCTTCaGCGAGTTCTACCTCAGTCTCATCCTGCTGCAGAACGAACCAGAATTTTGCCACaAATATTCATATGGTGTGCGAGCCATTGTTCAATGTATTCCAGCTTGGCTTCGCTTCATCCAGTGCCTGCGCCGGTACCGTGACACGAGGAGGGCTTTTCCTCATCTAGTAAATGCTGGCAAATACTCcACAACTTTCTTCACcGTGACATTTGCAGCCCTTTACAGCACTCACAAACCTCCTCTTTCTCCAGAACAAAAaCACTCTGACACCgtGGTgTTCTTTTACCTGTCTATTACTGCTACAACGTTTAAGCCTCATGTTGGGGAC

>Dom-IR21-2

GAGAAGCTGGCAGAGGCTCAGCGCAGGTTTGCTACACTTCAGAATGAGCTTCAGTCATCGCTGGATGTGCAGAAAGAAAGCAGTGcTGTCACagCAtTGCGACAgCGCAGAAAGCCAGTGTTCCACCTGTCCCACGAGGAGCGtGTCCAACATAGGAAcATTAAAGACCTTAAGCTGGCCTTCaGCGAGTTCTACCTCAGTCTCATCCTGCTGCAGAACGAACCAGAATTTTGCCACaAATATTCATATGGTGTGCGAGCCATTGTTCAATGTATTCCAGCTTGGCTTCGCTTCATCCAGTGCCTGCGCCGGTACCGTGACACGAGGAGGGCTTTTCCTCATCTAGTAAATGCTGGCAAATACTCcACAACTTTCTTCACcGTGACATTTGCAGCCCTTTACAGCACTCACAAACCTCCTCTTTCTCCAGAACAAAAaCACTCTGACACCgtGGTgTTCTTTTACCTGTCTATTACTGCTACAACGTTTAAGCCTCATGTTGGGGAC

>Dom-IR63-1

GAGAAGCTGGCAGAGGCTCAGCGCAGGTTTGCTACACTTCAGAATGAGCTTCAGTCATCGCTGGATGTGCAGAAAGAAAGCAGTGgTGTCACgaCAtTGCGACAgCGCAGAAAGCCAGTGTTCCACCTGTCCCACGAGGAGCGtGTCCAACATAGGAAcATTAAAGACCTTAAGCTGGCCTTCaGCGAGTTCTACCTCAGTCTCATCCTGCTGCAGAACGAACCAGAATTTTGCCACaAATATTCATATGGTGTGCGAGCCATTGTTCAATGTATTCCAGCTTGGCTTCGCTTCATCCAGTGCCTGCGCCGGTACCGTGACACGAGGAGGGCTTTTCCTCATCTAGTAAATGCTGGCAAATACTCcACAACTTTCTTCACcGTGACATTTGCAGCCCTTTACAGCACTCACAAACCTCCTCTTTCTCCAGAACAAAAaCACTCTGACACCgtGGTgTTCTTTTACCTGTCTATTACTGCTACAACGTTTAAGCCTCATGTTGGGGAC

>Dom-IR63-2

GAGAAGCTGGCAGAGGCTCAGCGCAGGTTTGCTACACTTCAGAATGAGCTTCAGTCATCGCTGGATGTGCAGAAAGAAAGCAGTGgTGTCACgaCAtTGCGACAgCGCAGAAAGCCAGTGTTCCACCTGTCCCACGAGGAGCGtGTCCAACATAGGAAcATTAAAGACCTTAAGCTGGCCTTCaGCGAGTTCTACCTCAGTCTCATCCTGCTGCAGAACGAACCAGAATTTTGCCACaAATATTCATATGGTGTGCGAGCCATTGTTCAATGTATTCCAGCTTGGCTTCGCTTCATCCAGTGCCTGCGCCGGTACCGTGACACGAGGAGGGCTTTTCCTCATCTAGTAAATGCTGGCAAATACTCcACAACTTTCTTCACcGTGACATTTGCAGCCCTTTACAGCACTCACAAACCTCCTCTTTCTCCAGAACAAAAaCACTCTGACACCgtGGTgTTCTTTTACCTGTCTATTACTGCTACAACGTTTAAGCCTCATGTTGGGGAC

>Dom-IR64-1

GAGAAGCTGGCAGAGGCTCAGCGCAGGTTTGCTACACTTCAGAATGAGCTTCAGTCATCGCTGGATGTGCAGAAAGAAAGCAGTGgTGTCACgaCAtTGCGACAgCGCAGAAAGCCAGTGTTCCACCTGTCCCACGAGGAGCGtGTCCAACATAGGAAcATTAAAGACCTTAAGCTGGCCTTCaGCGAGTTCTACCTCAGTCTCATCCTGCTGCAGAACGAACCAGAATTTTGCCACaAATATTCATATGGTGTGCGAGCCATTGTTCAATGTATTCCAGCTTGGCTTCGCTTCATCCAGTGCCTGCGCCGGTACCGTGACACGAGGAGGGCTTTTCCTCATCTAGTAAATGCTGGCAAATACTCcACAACTTTCTTCACcGTGACATTTGCAGCCCTTTACAGCACTCACAAACCTCCTCTTTCTCCAGAACAAAAaCACTCTGACACCgtGGTgTTCTTTTACCTGTCTATTACTGCTACAACGTTTAAGCCTCATGTTGGGGAC

>Dom-IR64-2

GAGAAGCTGGCAGAGGCTCAGCGCAGGTTTGCTACACTTCAGAATGAGCTTCAGTCATCGCTGGATGTGCAGAAAGAAAGCAGTGgTGTCACgaCAtTGCGACAgCGCAGAAAGCCAGTGTTCCACCTGTCCCACGAGGAGCGtGTCCAACATAGGAAcATTAAAGACCTTAAGCTGGCCTTCaGCGAGTTCTACCTCAGTCTCATCCTGCTGCAGAACGAACCAGAATTTTGCCACaAATATTCATATGGTGTGCGAGCCATTGTTCAATGTATTCCAGCTTGGCTTCGCTTCATCCAGTGCCTGCGCCGGTACCGTGACACGAGGAGGGCTTTTCCTCATCTAGTAAATGCTGGCAAATACTCcACAACTTTCTTCACcGTGACATTTGCAGCCCTTTACAGCACTCACAAACCTCCTCTTTCTCCAGAACAAAAaCACTCTGACACCgtGGTgTTCTTTTACCTGTCTATTACTGCTACAACGTTTAAGCCTCATGTTGGGGAC

>Dom-IR61-1

GAGAAGCTGGCAGAGGCTCAGCGCAGGTTTGCTACACTTCAGAATGAGCTTCAGTCATCGCTGGATGTGCAGAAAGAAAGCAGTGgTGTCACgaCAtTGCGACAgCGCAGAAAGCCAGTGTTCCACCTGTCCCACGAGGAGCGtGTCCAACATAGGAAcATTAAAGACCTTAAGCTGGCCTTCaGCGAGTTCTACCTCAGTCTCATCCTGCTGCAGAACGAACCAGAATTTTGCCACaAATATTCATATGGTGTGCGAGCCATTGTTCAATGTATTCCAGCTTGGCTTCGCTTCATCCAGTGCCTGCGCCGGTACCGTGACACGAGGAGGGCTTTTCCTCATCTAGTAAATGCTGGCAAATACTCcACAACTTTCTTCACcGTGACATTTGCAGCCCTTTACAGCACTCACAAACCTCCTCTTTCTCCAGAACAAAAaCACTCTGACACCgtGGTgTTCTTTTACCTGTCTATTACTGCTACAACGTTTAAGCCTCATGTTGGGGAC

>Dom-IR61-2

GAGAAGCTGGCAGAGGCTCAGCGCAGGTTTGCTACACTTCAGAATGAGCTTCAGTCATCGCTGGATGTGCAGAAAGAAAGCAGTGgTGTCACgaCAtTGCGACAgCGCAGAAAGCCAGTGTTCCACCTGTCCCACGAGGAGCGtGTCCAACATAGGAAcATTAAAGACCTTAAGCTGGCCTTCaGCGAGTTCTACCTCAGTCTCATCCTGCTGCAGAACGAACCAGAATTTTGCCACaAATATTCATATGGTGTGCGAGCCATTGTTCAATGTATTCCAGCTTGGCTTCGCTTCATCCAGTGCCTGCGCCGGTACCGTGACACGAGGAGGGCTTTTCCTCATCTAGTAAATGCTGGCAAATACTCcACAACTTTCTTCACcGTGACATTTGCAGCCCTTTACAGCACTCACAAACCTCCTCTTTCTCCAGAACAAAAaCACTCTGACACCgtGGTgTTCTTTTACCTGTCTATTACTGCTACAACGTTTAAGCCTCATGTTGGGGAC

>Dom-IR52-1

GAGAAGCTGGCAGAGGCTCAGCGCAGGTTTGCTACACTTCAGAATGAGCTTCAGTCATCGCTGGATGTGCAGAAAGAAAGCAGTGgTGTCACgaCAtTGCGACAgCGCAGAAAGCCAGTGTTCCACCTGTCCCACGAGGAGCGtGTCCAACATAGGAAcATTAAAGACCTTAAGCTGGCCTTCaGCGAGTTCTACCTCAGTCTCATCCTGCTGCAGAACGAACCAGAATTTTGCCACaAATATTCATATGGTGTGCGAGCCATTGTTCAATGTATTCCAGCTTGGCTTCGCTTCATCCAGTGCCTGCGCCGGTACCGTGACACGAGGAGGGCTTTTCCTCATCTAGTAAATGCTGGCAAATACTCcACAACTTTCTTCACcGTGACATTTGCAGCCCTTTACAGCACTCACAAACCTCCTCTTTCTCCAGAACAAAAaCACTCTGACACCgtGGTgTTCTTTTACCTGTCTATTACTGCTACAACGTTTAAGCCTCATGTTGGGGAC

>Dom-IR52-2

GAGAAGCTGGCAGAGGCTCAGCGCAGGTTTGCTACACTTCAGAATGAGCTTCAGTCATCGCTGGATGTGCAGAAAGAAAGCAGTGgTGTCACgaCAtTGCGACAgCGCAGAAAGCCAGTGTTCCACCTGTCCCACGAGGAGCGtGTCCAACATAGGAAcATTAAAGACCTTAAGCTGGCCTTCaGCGAGTTCTACCTCAGTCTCATCCTGCTGCAGAACGAACCAGAATTTTGCCACaAATATTCATATGGTGTGCGAGCCATTGTTCAATGTATTCCAGCTTGGCTTCGCTTCATCCAGTGCCTGCGCCGGTACCGTGACACGAGGAGGGCTTTTCCTCATCTAGTAAATGCTGGCAAATACTCcACAACTTTCTTCACcGTGACATTTGCAGCCCTTTACAGCACTCACAAACCTCCTCTTTCTCCAGAACAAAAaCACTCTGACACCgtGGTgTTCTTTTACCTGTCTATTACTGCTACAACGTTTAAGCCTCATGTTGGGGAC

>Dom-IR51-1

GAGAAGCTGGCAGAGGCTCAGCGCAGGTTTGCTACACTTCAGAATGAGCTTCAGTCATCGCTGGATGTGCAGAAAGAAAGCAGTGgTGTCACgaCAtTGCGACAgCGCAGAAAGCCAGTGTTCCACCTGTCCCACGAGGAGCGtGTCCAACATAGGAAcATTAAAGACCTTAAGCTGGCCTTCaGCGAGTTCTACCTCAGTCTCATCCTGCTGCAGAACGAACCAGAATTTTGCCACaAATATTCATATGGTGTGCGAGCCATTGTTCAATGTATTCCAGCTTGGCTTCGCTTCATCCAGTGCCTGCGCCGGTACCGTGACACGAGGAGGGCTTTTCCTCATCTAGTAAATGCTGGCAAATACTCcACAACTTTCTTCACcGTGACATTTGCAGCCCTTTACAGCACTCACAAACCTCCTCTTTCTCCAGAACAAAAaCACTCTGACACCgtGGTgTTCTTTTACCTGTCTATTACTGCTACAACGTTTAAGCCTCATGTTGGGGAC

>Dom-IR51-2

GAGAAGCTGGCAGAGGCTCAGCGCAGGTTTGCTACACTTCAGAATGAGCTTCAGTCATCGCTGGATGTGCAGAAAGAAAGCAGTGgTGTCACgaCAtTGCGACAgCGCAGAAAGCCAGTGTTCCACCTGTCCCACGAGGAGCGtGTCCAACATAGGAAcATTAAAGACCTTAAGCTGGCCTTCaGCGAGTTCTACCTCAGTCTCATCCTGCTGCAGAACGAACCAGAATTTTGCCACaAATATTCATATGGTGTGCGAGCCATTGTTCAATGTATTCCAGCTTGGCTTCGCTTCATCCAGTGCCTGCGCCGGTACCGTGACACGAGGAGGGCTTTTCCTCATCTAGTAAATGCTGGCAAATACTCcACAACTTTCTTCACcGTGACATTTGCAGCCCTTTACAGCACTCACAAACCTCCTCTTTCTCCAGAACAAAAaCACTCTGACACCgtGGTgTTCTTTTACCTGTCTATTACTGCTACAACGTTTAAGCCTCATGTTGGGGAC

>Dom-IR31-1

GAGAAGCTGGCAGAGGCTCAGCGCAGGTTTGCTACACTTCAGAATGAGCTTCAGTCATCGCTGGATGTGCAGAAAGAAAGCAGTGgTGTCACgaCAtTGCGACAgCGCAGAAAGCCAGTGTTCCACCTGTCCCACGAGGAGCGtGTCCAACATAGGAAcATTAAAGACCTTAAGCTGGCCTTCaGCGAGTTCTACCTCAGTCTCATCCTGCTGCAGAACGAACCAGAATTTTGCCACaAATATTCATATGGTGTGCGAGCCATTGTTCAATGTATTCCAGCTTGGCTTCGCTTCATCCAGTGCCTGCGCCGGTACCGTGACACGAGGAGGGCTTTTCCTCATCTAGTAAATGCTGGCAAATACTCcACAACTTTCTTCACcGTGACATTTGCAGCCCTTTACAGCACTCACAAACCTCCTCTTTCTCCAGAACAAAAaCACTCTGACACCgtGGTgTTCTTTTACCTGTCTATTACTGCTACAACGTTTAAGCCTCATGTTGGGGAC

>Dom-IR31-2

GAGAAGCTGGCAGAGGCTCAGCGCAGGTTTGCTACACTTCAGAATGAGCTTCAGTCATCGCTGGATGTGCAGAAAGAAAGCAGTGgTGTCACgaCAtTGCGACAgCGCAGAAAGCCAGTGTTCCACCTGTCCCACGAGGAGCGtGTCCAACATAGGAAcATTAAAGACCTTAAGCTGGCCTTCaGCGAGTTCTACCTCAGTCTCATCCTGCTGCAGAACGAACCAGAATTTTGCCACaAATATTCATATGGTGTGCGAGCCATTGTTCAATGTATTCCAGCTTGGCTTCGCTTCATCCAGTGCCTGCGCCGGTACCGTGACACGAGGAGGGCTTTTCCTCATCTAGTAAATGCTGGCAAATACTCcACAACTTTCTTCACcGTGACATTTGCAGCCCTTTACAGCACTCACAAACCTCCTCTTTCTCCAGAACAAAAaCACTCTGACACCgtGGTgTTCTTTTACCTGTCTATTACTGCTACAACGTTTAAGCCTCATGTTGGGGAC

>Dom-IR29-1

GAGAAGCTGGCAGAGGCTCAGCGCAGGTTTGCTACACTTCAGAATGAGCTTCAGTCATCGCTGGATGTGCAGAAAGAAAGCAGTGgTGTCACgaCAtTGCGACAgCGCAGAAAGCCAGTGTTCCACCTGTCCCACGAGGAGCGtGTCCAACATAGGAAcATTAAAGACCTTAAGCTGGCCTTCaGCGAGTTCTACCTCAGTCTCATCCTGCTGCAGAACGAACCAGAATTTTGCCACaAATATTCATATGGTGTGCGAGCCATTGTTCAATGTATTCCAGCTTGGCTTCGCTTCATCCAGTGCCTGCGCCGGTACCGTGACACGAGGAGGGCTTTTCCTCATCTAGTAAATGCTGGCAAATACTCcACAACTTTCTTCACcGTGACATTTGCAGCCCTTTACAGCACTCACAAACCTCCTCTTTCTCCAGAACAAAAaCACTCTGACACCgtGGTgTTCTTTTACCTGTCTATTACTGCTACAACGTTTAAGCCTCATGTTGGGGAC

>Dom-IR29-2

GAGAAGCTGGCAGAGGCTCAGCGCAGGTTTGCTACACTTCAGAATGAGCTTCAGTCATCGCTGGATGTGCAGAAAGAAAGCAGTGgTGTCACgaCAtTGCGACAgCGCAGAAAGCCAGTGTTCCACCTGTCCCACGAGGAGCGtGTCCAACATAGGAAcATTAAAGACCTTAAGCTGGCCTTCaGCGAGTTCTACCTCAGTCTCATCCTGCTGCAGAACGAACCAGAATTTTGCCACaAATATTCATATGGTGTGCGAGCCATTGTTCAATGTATTCCAGCTTGGCTTCGCTTCATCCAGTGCCTGCGCCGGTACCGTGACACGAGGAGGGCTTTTCCTCATCTAGTAAATGCTGGCAAATACTCcACAACTTTCTTCACcGTGACATTTGCAGCCCTTTACAGCACTCACAAACCTCCTCTTTCTCCAGAACAAAAaCACTCTGACACCgtGGTgTTCTTTTACCTGTCTATTACTGCTACAACGTTTAAGCCTCATGTTGGGGAC

>Dom-IR27-1

GAGAAGCTGGCAGAGGCTCAGCGCAGGTTTGCTACACTTCAGAATGAGCTTCAGTCATCGCTGGATGTGCAGAAAGAAAGCAGTGgTGTCACgaCAtTGCGACAgCGCAGAAAGCCAGTGTTCCACCTGTCCCACGAGGAGCGtGTCCAACATAGGAAcATTAAAGACCTTAAGCTGGCCTTCaGCGAGTTCTACCTCAGTCTCATCCTGCTGCAGAACGAACCAGAATTTTGCCACaAATATTCATATGGTGTGCGAGCCATTGTTCAATGTATTCCAGCTTGGCTTCGCTTCATCCAGTGCCTGCGCCGGTACCGTGACACGAGGAGGGCTTTTCCTCATCTAGTAAATGCTGGCAAATACTCcACAACTTTCTTCACcGTGACATTTGCAGCCCTTTACAGCACTCACAAACCTCCTCTTTCTCCAGAACAAAAaCACTCTGACACCgtGGTgTTCTTTTACCTGTCTATTACTGCTACAACGTTTAAGCCTCATGTTGGGGAC

>Dom-IR27-2

GAGAAGCTGGCAGAGGCTCAGCGCAGGTTTGCTACACTTCAGAATGAGCTTCAGTCATCGCTGGATGTGCAGAAAGAAAGCAGTGgTGTCACgaCAtTGCGACAgCGCAGAAAGCCAGTGTTCCACCTGTCCCACGAGGAGCGtGTCCAACATAGGAAcATTAAAGACCTTAAGCTGGCCTTCaGCGAGTTCTACCTCAGTCTCATCCTGCTGCAGAACGAACCAGAATTTTGCCACaAATATTCATATGGTGTGCGAGCCATTGTTCAATGTATTCCAGCTTGGCTTCGCTTCATCCAGTGCCTGCGCCGGTACCGTGACACGAGGAGGGCTTTTCCTCATCTAGTAAATGCTGGCAAATACTCcACAACTTTCTTCACcGTGACATTTGCAGCCCTTTACAGCACTCACAAACCTCCTCTTTCTCCAGAACAAAAaCACTCTGACACCgtGGTgTTCTTTTACCTGTCTATTACTGCTACAACGTTTAAGCCTCATGTTGGGGAC

>Dom-IR25-1

GAGAAGCTGGCAGAGGCTCAGCGCAGGTTTGCTACACTTCAGAATGAGCTTCAGTCATCGCTGGATGTGCAGAAAGAAAGCAGTGgTGTCACgaCAtTGCGACAgCGCAGAAAGCCAGTGTTCCACCTGTCCCACGAGGAGCGtGTCCAACATAGGAAcATTAAAGACCTTAAGCTGGCCTTCaGCGAGTTCTACCTCAGTCTCATCCTGCTGCAGAACGAACCAGAATTTTGCCACaAATATTCATATGGTGTGCGAGCCATTGTTCAATGTATTCCAGCTTGGCTTCGCTTCATCCAGTGCCTGCGCCGGTACCGTGACACGAGGAGGGCTTTTCCTCATCTAGTAAATGCTGGCAAATACTCcACAACTTTCTTCACcGTGACATTTGCAGCCCTTTACAGCACTCACAAACCTCCTCTTTCTCCAGAACAAAAaCACTCTGACACCgtGGTgTTCTTTTACCTGTCTATTACTGCTACAACGTTTAAGCCTCATGTTGGGGAC

>Dom-IR25-2

GAGAAGCTGGCAGAGGCTCAGCGCAGGTTTGCTACACTTCAGAATGAGCTTCAGTCATCGCTGGATGTGCAGAAAGAAAGCAGTGgTGTCACgaCAtTGCGACAgCGCAGAAAGCCAGTGTTCCACCTGTCCCACGAGGAGCGtGTCCAACATAGGAAcATTAAAGACCTTAAGCTGGCCTTCaGCGAGTTCTACCTCAGTCTCATCCTGCTGCAGAACGAACCAGAATTTTGCCACaAATATTCATATGGTGTGCGAGCCATTGTTCAATGTATTCCAGCTTGGCTTCGCTTCATCCAGTGCCTGCGCCGGTACCGTGACACGAGGAGGGCTTTTCCTCATCTAGTAAATGCTGGCAAATACTCcACAACTTTCTTCACcGTGACATTTGCAGCCCTTTACAGCACTCACAAACCTCCTCTTTCTCCAGAACAAAAaCACTCTGACACCgtGGTgTTCTTTTACCTGTCTATTACTGCTACAACGTTTAAGCCTCATGTTGGGGAC

>Dom-IR23-1

GAGAAGCTGGCAGAGGCTCAGCGCAGGTTTGCTACACTTCAGAATGAGCTTCAGTCATCGCTGGATGTGCAGAAAGAAAGCAGTGgTGTCACgaCAtTGCGACAgCGCAGAAAGCCAGTGTTCCACCTGTCCCACGAGGAGCGtGTCCAACATAGGAAcATTAAAGACCTTAAGCTGGCCTTCaGCGAGTTCTACCTCAGTCTCATCCTGCTGCAGAACGAACCAGAATTTTGCCACaAATATTCATATGGTGTGCGAGCCATTGTTCAATGTATTCCAGCTTGGCTTCGCTTCATCCAGTGCCTGCGCCGGTACCGTGACACGAGGAGGGCTTTTCCTCATCTAGTAAATGCTGGCAAATACTCcACAACTTTCTTCACcGTGACATTTGCAGCCCTTTACAGCACTCACAAACCTCCTCTTTCTCCAGAACAAAAaCACTCTGACACCgtGGTgTTCTTTTACCTGTCTATTACTGCTACAACGTTTAAGCCTCATGTTGGGGAC

>Dom-IR23-2

GAGAAGCTGGCAGAGGCTCAGCGCAGGTTTGCTACACTTCAGAATGAGCTTCAGTCATCGCTGGATGTGCAGAAAGAAAGCAGTGgTGTCACgaCAtTGCGACAgCGCAGAAAGCCAGTGTTCCACCTGTCCCACGAGGAGCGtGTCCAACATAGGAAcATTAAAGACCTTAAGCTGGCCTTCaGCGAGTTCTACCTCAGTCTCATCCTGCTGCAGAACGAACCAGAATTTTGCCACaAATATTCATATGGTGTGCGAGCCATTGTTCAATGTATTCCAGCTTGGCTTCGCTTCATCCAGTGCCTGCGCCGGTACCGTGACACGAGGAGGGCTTTTCCTCATCTAGTAAATGCTGGCAAATACTCcACAACTTTCTTCACcGTGACATTTGCAGCCCTTTACAGCACTCACAAACCTCCTCTTTCTCCAGAACAAAAaCACTCTGACACCgtGGTgTTCTTTTACCTGTCTATTACTGCTACAACGTTTAAGCCTCATGTTGGGGAC

>Dom-IR22-1

GAGAAGCTGGCAGAGGCTCAGCGCAGGTTTGCTACACTTCAGAATGAGCTTCAGTCATCGCTGGATGTGCAGAAAGAAAGCAGTGgTGTCACgaCAtTGCGACAgCGCAGAAAGCCAGTGTTCCACCTGTCCCACGAGGAGCGtGTCCAACATAGGAAcATTAAAGACCTTAAGCTGGCCTTCaGCGAGTTCTACCTCAGTCTCATCCTGCTGCAGAACGAACCAGAATTTTGCCACaAATATTCATATGGTGTGCGAGCCATTGTTCAATGTATTCCAGCTTGGCTTCGCTTCATCCAGTGCCTGCGCCGGTACCGTGACACGAGGAGGGCTTTTCCTCATCTAGTAAATGCTGGCAAATACTCcACAACTTTCTTCACcGTGACATTTGCAGCCCTTTACAGCACTCACAAACCTCCTCTTTCTCCAGAACAAAAaCACTCTGACACCgtGGTgTTCTTTTACCTGTCTATTACTGCTACAACGTTTAAGCCTCATGTTGGGGAC

>Dom-IR22-2

GAGAAGCTGGCAGAGGCTCAGCGCAGGTTTGCTACACTTCAGAATGAGCTTCAGTCATCGCTGGATGTGCAGAAAGAAAGCAGTGgTGTCACgaCAtTGCGACAgCGCAGAAAGCCAGTGTTCCACCTGTCCCACGAGGAGCGtGTCCAACATAGGAAcATTAAAGACCTTAAGCTGGCCTTCaGCGAGTTCTACCTCAGTCTCATCCTGCTGCAGAACGAACCAGAATTTTGCCACaAATATTCATATGGTGTGCGAGCCATTGTTCAATGTATTCCAGCTTGGCTTCGCTTCATCCAGTGCCTGCGCCGGTACCGTGACACGAGGAGGGCTTTTCCTCATCTAGTAAATGCTGGCAAATACTCcACAACTTTCTTCACcGTGACATTTGCAGCCCTTTACAGCACTCACAAACCTCCTCTTTCTCCAGAACAAAAaCACTCTGACACCgtGGTgTTCTTTTACCTGTCTATTACTGCTACAACGTTTAAGCCTCATGTTGGGGAC

>Musc-CR1-1

GAGAAGCTGGCAGAGGCTCAGCGCAGGTTTGCTACACTTCAGAATGAGCTTCAGTCATCGCTGGATGTGCAGAAAGAAAGCAGTGgTGTCACgaCAtTGCGACAgCGCAGAAAGCCAGTGTTCCACCTGTCCCACGAGGAGCGtGTCCAACATAGGAAcATTAAAGACCTTAAGCTGGCCTTCaGCGAGTTCTACCTCAGTCTCATCCTGCTGCAGAACGAACCAGAATTTTGCCACcAATATTCATATGGTGTGCGAGCCATTGTTCAATGTATTCCAGCTTGGCTTCGCTTCATCCAGTGCCTGCGCCGGTACCGTGACACGAGGAGGGCTTTTCCTCATCTAGTAAATGCTGGCAAATACTCcACAACTTTCTTCACcGTGACATTTGCAGCCCTTTACAGCACTCACAAACCTCCTCTTTCTCCAGAACAAAAtCACTCTGACACCgtGGTgTTCTTTTACCTGTCT---ACTGCTACAAaGTTTAAGCCTCATGTTGGGGAC

>Musc-CR1-2

GAGAAGCTGGCAGAGGCTCAGCGCAGGTTTGCTACACTTCAGAATGAGCTTCAGTCATCGCTGGATGTGCAGAAAGAAAGCAGTGgTGTCACgaCAtTGCGACAgCGCAGAAAGCCAGTGTTCCACCTGTCCCACGAGGAGCGtGTCCAACATAGGAAcATTAAAGACCTTAAGCTGGCCTTCaGCGAGTTCTACCTCAGTCTCATCCTGCTGCAGAACGAACCAGAATTTTGCCACcAATATTCATATGGTGTGCGAGCCATTGTTCAATGTATTCCAGCTTGGCTTCGCTTCATCCAGTGCCTGCGCCGGTACCGTGACACGAGGAGGGCTTTTCCTCATCTAGTAAATGCTGGCAAATACTCcACAACTTTCTTCACcGTGACATTTGCAGCCCTTTACAGCACTCACAAACCTCCTCTTTCTCCAGAACAAAAtCACTCTGACACCgtGGTgTTCTTTTACCTGTCT---ACTGCTACAAaGTTTAAGCCTCATGTTGGGGAC

>Musc-CR18-1

GAGAAGCTGGCAGAGGCTCAGCGCAGGTTTGCTACACTTCAGAATGAGCTTCAGTCATCGCTGGATGTGCAGAAAGAAAGCAGTGgTGTCACgaCAtTGCGACAgCGCAGAAAGCCAGTGTTCCACCTGTCCCACGAGGAGCGtGTCCAACATAGGAAcATTAAAGACCTTAAGCTGGCCTTCaGCGAGTTCTACCTCAGTCTCATCCTGCTGCAGAACGAACCAGAATTTTGCCACcAATATTCATATGGTGTGCGAGCCATTGTTCAATGTATTCCAGCTTGGCTTCGCTTCATCCAGTGCCTGCGCCGGTACCGTGACACGAGGAGGGCTTTTCCTCATCTAGTAAATGCTGGCAAATACTCcACAACTTTCTTCACcGTGACATTTGCAGCCCTTTACAGCACTCACAAACCTCCTCTTTCTCCAGAACAAAAtCACTCTGACACCgtGGTgTTCTTTTACCTGTCT---ACTGCTACAAaGTTTAAGCCTCATGTTGGGGAC

>Musc-CR18-2

GAGAAGCTGGCAGAGGCTCAGCGCAGGTTTGCTACACTTCAGAATGAGCTTCAGTCATCGCTGGATGTGCAGAAAGAAAGCAGTGgTGTCACgaCAtTGCGACAgCGCAGAAAGCCAGTGTTCCACCTGTCCCACGAGGAGCGtGTCCAACATAGGAAcATTAAAGACCTTAAGCTGGCCTTCaGCGAGTTCTACCTCAGTCTCATCCTGCTGCAGAACGAACCAGAATTTTGCCACcAATATTCATATGGTGTGCGAGCCATTGTTCAATGTATTCCAGCTTGGCTTCGCTTCATCCAGTGCCTGCGCCGGTACCGTGACACGAGGAGGGCTTTTCCTCATCTAGTAAATGCTGGCAAATACTCcACAACTTTCTTCACcGTGACATTTGCAGCCCTTTACAGCACTCACAAACCTCCTCTTTCTCCAGAACAAAAtCACTCTGACACCgtGGTgTTCTTTTACCTGTCT---ACTGCTACAAaGTTTAAGCCTCATGTTGGGGAC

>Musc-CR10-1

GAGAAGCTGGCAGAGGCTCAGCGCAGGTTTGCTACACTTCAGAATGAGCTTCAGTCATCGCTGGATGTGCAGAAAGAAAGCAGTGgTGTCACgaCAtTGCGACAgCGCAGAAAGCCAGTGTTCCACCTGTCCCACGAGGAGCGtGTCCAACATAGGAAcATTAAAGACCTTAAGCTGGCCTTCaGCGAGTTCTACCTCAGTCTCATCCTGCTGCAGAACGAACCAGAATTTTGCCACcAATATTCATATGGTGTGCGAGCCATTGTTCAATGTATTCCAGCTTGGCTTCGCTTCATCCAGTGCCTGCGCCGGTACCGTGACACGAGGAGGGCTTTTCCTCATCTAGTAAATGCTGGCAAATACTCcACAACTTTCTTCACcGTGACATTTGCAGCCCTTTACAGCACTCACAAACCTCCTCTTTCTCCAGAACAAAAtCACTCTGACACCgtGGTgTTCTTTTACCTGTCT---ACTGCTACAAaGTTTAAGCCTCATGTTGGGGAC

>Musc-CR10-2

GAGAAGCTGGCAGAGGCTCAGCGCAGGTTTGCTACACTTCAGAATGAGCTTCAGTCATCGCTGGATGTGCAGAAAGAAAGCAGTGgTGTCACgaCAtTGCGACAgCGCAGAAAGCCAGTGTTCCACCTGTCCCACGAGGAGCGtGTCCAACATAGGAAcATTAAAGACCTTAAGCTGGCCTTCaGCGAGTTCTACCTCAGTCTCATCCTGCTGCAGAACGAACCAGAATTTTGCCACcAATATTCATATGGTGTGCGAGCCATTGTTCAATGTATTCCAGCTTGGCTTCGCTTCATCCAGTGCCTGCGCCGGTACCGTGACACGAGGAGGGCTTTTCCTCATCTAGTAAATGCTGGCAAATACTCcACAACTTTCTTCACcGTGACATTTGCAGCCCTTTACAGCACTCACAAACCTCCTCTTTCTCCAGAACAAAAtCACTCTGACACCgtGGTgTTCTTTTACCTGTCT---ACTGCTACAAaGTTTAAGCCTCATGTTGGGGAC

>Musc-CR12-1

GAGAAGCTGGCAGAGGCTCAGCGCAGGTTTGCTACACTTCAGAATGAGCTTCAGTCATCGCTGGATGTGCAGAAAGAAAGCAGTGgTGTCACgaCAtTGCGACAgCGCAGAAAGCCAGTGTTCCACCTGTCCCACGAGGAGCGtGTCCAACATAGGAAcATTAAAGACCTTAAGCTGGCCTTCaGCGAGTTCTACCTCAGTCTCATCCTGCTGCAGAACGAACCAGAATTTTGCCACcAATATTCATATGGTGTGCGAGCCATTGTTCAATGTATTCCAGCTTGGCTTCGCTTCATCCAGTGCCTGCGCCGGTACCGTGACACGAGGAGGGCTTTTCCTCATCTAGTAAATGCTGGCAAATACTCcACAACTTTCTTCACcGTGACATTTGCAGCCCTTTACAGCACTCACAAACCTCCTCTTTCTCCAGAACAAAAtCACTCTGACACCgtGGTgTTCTTTTACCTGTCT---ACTGCTACAAaGTTTAAGCCTCATGTTGGGGAC

>Musc-CR12-2

GAGAAGCTGGCAGAGGCTCAGCGCAGGTTTGCTACACTTCAGAATGAGCTTCAGTCATCGCTGGATGTGCAGAAAGAAAGCAGTGgTGTCACgaCAtTGCGACAgCGCAGAAAGCCAGTGTTCCACCTGTCCCACGAGGAGCGtGTCCAACATAGGAAcATTAAAGACCTTAAGCTGGCCTTCaGCGAGTTCTACCTCAGTCTCATCCTGCTGCAGAACGAACCAGAATTTTGCCACcAATATTCATATGGTGTGCGAGCCATTGTTCAATGTATTCCAGCTTGGCTTCGCTTCATCCAGTGCCTGCGCCGGTACCGTGACACGAGGAGGGCTTTTCCTCATCTAGTAAATGCTGGCAAATACTCcACAACTTTCTTCACcGTGACATTTGCAGCCCTTTACAGCACTCACAAACCTCCTCTTTCTCCAGAACAAAAtCACTCTGACACCgtGGTgTTCTTTTACCTGTCT---ACTGCTACAAaGTTTAAGCCTCATGTTGGGGAC

>Musc-CR13-1

GAGAAGCTGGCAGAGGCTCAGCGCAGGTTTGCTACACTTCAGAATGAGCTTCAGTCATCGCTGGATGTGCAGAAAGAAAGCAGTGgTGTCACgaCAtTGCGACAgCGCAGAAAGCCAGTGTTCCACCTGTCCCACGAGGAGCGtGTCCAACATAGGAAcATTAAAGACCTTAAGCTGGCCTTCaGCGAGTTCTACCTCAGTCTCATCCTGCTGCAGAACGAACCAGAATTTTGCCACcAATATTCATATGGTGTGCGAGCCATTGTTCAATGTATTCCAGCTTGGCTTCGCTTCATCCAGTGCCTGCGCCGGTACCGTGACACGAGGAGGGCTTTTCCTCATCTAGTAAATGCTGGCAAATACTCcACAACTTTCTTCACcGTGACATTTGCAGCCCTTTACAGCACTCACAAACCTCCTCTTTCTCCAGAACAAAAtCACTCTGACACCgtGGTgTTCTTTTACCTGTCT---ACTGCTACAAaGTTTAAGCCTCATGTTGGGGAC

>Musc-CR13-2

GAGAAGCTGGCAGAGGCTCAGCGCAGGTTTGCTACACTTCAGAATGAGCTTCAGTCATCGCTGGATGTGCAGAAAGAAAGCAGTGgTGTCACgaCAtTGCGACAgCGCAGAAAGCCAGTGTTCCACCTGTCCCACGAGGAGCGtGTCCAACATAGGAAcATTAAAGACCTTAAGCTGGCCTTCaGCGAGTTCTACCTCAGTCTCATCCTGCTGCAGAACGAACCAGAATTTTGCCACcAATATTCATATGGTGTGCGAGCCATTGTTCAATGTATTCCAGCTTGGCTTCGCTTCATCCAGTGCCTGCGCCGGTACCGTGACACGAGGAGGGCTTTTCCTCATCTAGTAAATGCTGGCAAATACTCcACAACTTTCTTCACcGTGACATTTGCAGCCCTTTACAGCACTCACAAACCTCCTCTTTCTCCAGAACAAAAtCACTCTGACACCgtGGTgTTCTTTTACCTGTCT---ACTGCTACAAaGTTTAAGCCTCATGTTGGGGAC

>Musc-CR14-1

GAGAAGCTGGCAGAGGCTCAGCGCAGGTTTGCTACACTTCAGAATGAGCTTCAGTCATCGCTGGATGTGCAGAAAGAAAGCAGTGgTGTCACgaCAtTGCGACAgCGCAGAAAGCCAGTGTTCCACCTGTCCCACGAGGAGCGtGTCCAACATAGGAAcATTAAAGACCTTAAGCTGGCCTTCaGCGAGTTCTACCTCAGTCTCATCCTGCTGCAGAACGAACCAGAATTTTGCCACcAATATTCATATGGTGTGCGAGCCATTGTTCAATGTATTCCAGCTTGGCTTCGCTTCATCCAGTGCCTGCGCCGGTACCGTGACACGAGGAGGGCTTTTCCTCATCTAGTAAATGCTGGCAAATACTCcACAACTTTCTTCACcGTGACATTTGCAGCCCTTTACAGCACTCACAAACCTCCTCTTTCTCCAGAACAAAAtCACTCTGACACCgtGGTgTTCTTTTACCTGTCT---ACTGCTACAAaGTTTAAGCCTCATGTTGGGGAC

>Musc-CR14-2

GAGAAGCTGGCAGAGGCTCAGCGCAGGTTTGCTACACTTCAGAATGAGCTTCAGTCATCGCTGGATGTGCAGAAAGAAAGCAGTGgTGTCACgaCAtTGCGACAgCGCAGAAAGCCAGTGTTCCACCTGTCCCACGAGGAGCGtGTCCAACATAGGAAcATTAAAGACCTTAAGCTGGCCTTCaGCGAGTTCTACCTCAGTCTCATCCTGCTGCAGAACGAACCAGAATTTTGCCACcAATATTCATATGGTGTGCGAGCCATTGTTCAATGTATTCCAGCTTGGCTTCGCTTCATCCAGTGCCTGCGCCGGTACCGTGACACGAGGAGGGCTTTTCCTCATCTAGTAAATGCTGGCAAATACTCcACAACTTTCTTCACcGTGACATTTGCAGCCCTTTACAGCACTCACAAACCTCCTCTTTCTCCAGAACAAAAtCACTCTGACACCgtGGTgTTCTTTTACCTGTCT---ACTGCTACAAaGTTTAAGCCTCATGTTGGGGAC

>Musc-CR16-1

GAGAAGCTGGCAGAGGCTCAGCGCAGGTTTGCTACACTTCAGAATGAGCTTCAGTCATCGCTGGATGTGCAGAAAGAAAGCAGTGgTGTCACgaCAtTGCGACAgCGCAGAAAGCCAGTGTTCCACCTGTCCCACGAGGAGCGtGTCCAACATAGGAAcATTAAAGACCTTAAGCTGGCCTTCaGCGAGTTCTACCTCAGTCTCATCCTGCTGCAGAACGAACCAGAATTTTGCCACcAATATTCATATGGTGTGCGAGCCATTGTTCAATGTATTCCAGCTTGGCTTCGCTTCATCCAGTGCCTGCGCCGGTACCGTGACACGAGGAGGGCTTTTCCTCATCTAGTAAATGCTGGCAAATACTCcACAACTTTCTTCACcGTGACATTTGCAGCCCTTTACAGCACTCACAAACCTCCTCTTTCTCCAGAACAAAAtCACTCTGACACCgtGGTgTTCTTTTACCTGTCT---ACTGCTACAAaGTTTAAGCCTCATGTTGGGGAC

>Musc-CR16-2

GAGAAGCTGGCAGAGGCTCAGCGCAGGTTTGCTACACTTCAGAATGAGCTTCAGTCATCGCTGGATGTGCAGAAAGAAAGCAGTGgTGTCACgaCAtTGCGACAgCGCAGAAAGCCAGTGTTCCACCTGTCCCACGAGGAGCGtGTCCAACATAGGAAcATTAAAGACCTTAAGCTGGCCTTCaGCGAGTTCTACCTCAGTCTCATCCTGCTGCAGAACGAACCAGAATTTTGCCACcAATATTCATATGGTGTGCGAGCCATTGTTCAATGTATTCCAGCTTGGCTTCGCTTCATCCAGTGCCTGCGCCGGTACCGTGACACGAGGAGGGCTTTTCCTCATCTAGTAAATGCTGGCAAATACTCcACAACTTTCTTCACcGTGACATTTGCAGCCCTTTACAGCACTCACAAACCTCCTCTTTCTCCAGAACAAAAtCACTCTGACACCgtGGTgTTCTTTTACCTGTCT---ACTGCTACAAaGTTTAAGCCTCATGTTGGGGAC

>Musc-CR17-1

GAGAAGCTGGCAGAGGCTCAGCGCAGGTTTGCTACACTTCAGAATGAGCTTCAGTCATCGCTGGATGTGCAGAAAGAAAGCAGTGgTGTCACgaCAtTGCGACAgCGCAGAAAGCCAGTGTTCCACCTGTCCCACGAGGAGCGtGTCCAACATAGGAAcATTAAAGACCTTAAGCTGGCCTTCaGCGAGTTCTACCTCAGTCTCATCCTGCTGCAGAACGAACCAGAATTTTGCCACcAATATTCATATGGTGTGCGAGCCATTGTTCAATGTATTCCAGCTTGGCTTCGCTTCATCCAGTGCCTGCGCCGGTACCGTGACACGAGGAGGGCTTTTCCTCATCTAGTAAATGCTGGCAAATACTCcACAACTTTCTTCACcGTGACATTTGCAGCCCTTTACAGCACTCACAAACCTCCTCTTTCTCCAGAACAAAAtCACTCTGACACCgtGGTgTTCTTTTACCTGTCT---ACTGCTACAAaGTTTAAGCCTCATGTTGGGGAC

>Musc-CR17-2

GAGAAGCTGGCAGAGGCTCAGCGCAGGTTTGCTACACTTCAGAATGAGCTTCAGTCATCGCTGGATGTGCAGAAAGAAAGCAGTGgTGTCACgaCAtTGCGACAgCGCAGAAAGCCAGTGTTCCACCTGTCCCACGAGGAGCGtGTCCAACATAGGAAcATTAAAGACCTTAAGCTGGCCTTCaGCGAGTTCTACCTCAGTCTCATCCTGCTGCAGAACGAACCAGAATTTTGCCACcAATATTCATATGGTGTGCGAGCCATTGTTCAATGTATTCCAGCTTGGCTTCGCTTCATCCAGTGCCTGCGCCGGTACCGTGACACGAGGAGGGCTTTTCCTCATCTAGTAAATGCTGGCAAATACTCcACAACTTTCTTCACcGTGACATTTGCAGCCCTTTACAGCACTCACAAACCTCCTCTTTCTCCAGAACAAAAtCACTCTGACACCgtGGTgTTCTTTTACCTGTCT---ACTGCTACAAaGTTTAAGCCTCATGTTGGGGAC

>Musc-CR2-1

GAGAAGCTGGCAGAGGCTCAGCGCAGGTTTGCTACACTTCAGAATGAGCTTCAGTCATCGCTGGATGTGCAGAAAGAAAGCAGTGgTGTCACgaCAtTGCGACAgCGCAGAAAGCCAGTGTTCCACCTGTCCCACGAGGAGCGtGTCCAACATAGGAAcATTAAAGACCTTAAGCTGGCCTTCaGCGAGTTCTACCTCAGTCTCATCCTGCTGCAGAACGAACCAGAATTTTGCCACcAATATTCATATGGTGTGCGAGCCATTGTTCAATGTATTCCAGCTTGGCTTCGCTTCATCCAGTGCCTGCGCCGGTACCGTGACACGAGGAGGGCTTTTCCTCATCTAGTAAATGCTGGCAAATACTCcACAACTTTCTTCACcGTGACATTTGCAGCCCTTTACAGCACTCACAAACCTCCTCTTTCTCCAGAACAAAAtCACTCTGACACCgtGGTgTTCTTTTACCTGTCT---ACTGCTACAAaGTTTAAGCCTCATGTTGGGGAC

>Musc-CR2-2

GAGAAGCTGGCAGAGGCTCAGCGCAGGTTTGCTACACTTCAGAATGAGCTTCAGTCATCGCTGGATGTGCAGAAAGAAAGCAGTGgTGTCACgaCAtTGCGACAgCGCAGAAAGCCAGTGTTCCACCTGTCCCACGAGGAGCGtGTCCAACATAGGAAcATTAAAGACCTTAAGCTGGCCTTCaGCGAGTTCTACCTCAGTCTCATCCTGCTGCAGAACGAACCAGAATTTTGCCACcAATATTCATATGGTGTGCGAGCCATTGTTCAATGTATTCCAGCTTGGCTTCGCTTCATCCAGTGCCTGCGCCGGTACCGTGACACGAGGAGGGCTTTTCCTCATCTAGTAAATGCTGGCAAATACTCcACAACTTTCTTCACcGTGACATTTGCAGCCCTTTACAGCACTCACAAACCTCCTCTTTCTCCAGAACAAAAtCACTCTGACACCgtGGTgTTCTTTTACCTGTCT---ACTGCTACAAaGTTTAAGCCTCATGTTGGGGAC

>Musc-CR3-1

GAGAAGCTGGCAGAGGCTCAGCGCAGGTTTGCTACACTTCAGAATGAGCTTCAGTCATCGCTGGATGTGCAGAAAGAAAGCAGTGgTGTCACgaCAtTGCGACAgCGCAGAAAGCCAGTGTTCCACCTGTCCCACGAGGAGCGtGTCCAACATAGGAAcATTAAAGACCTTAAGCTGGCCTTCaGCGAGTTCTACCTCAGTCTCATCCTGCTGCAGAACGAACCAGAATTTTGCCACcAATATTCATATGGTGTGCGAGCCATTGTTCAATGTATTCCAGCTTGGCTTCGCTTCATCCAGTGCCTGCGCCGGTACCGTGACACGAGGAGGGCTTTTCCTCATCTAGTAAATGCTGGCAAATACTCcACAACTTTCTTCACcGTGACATTTGCAGCCCTTTACAGCACTCACAAACCTCCTCTTTCTCCAGAACAAAAtCACTCTGACACCgtGGTgTTCTTTTACCTGTCT---ACTGCTACAAaGTTTAAGCCTCATGTTGGGGAC

>Musc-CR3-2

GAGAAGCTGGCAGAGGCTCAGCGCAGGTTTGCTACACTTCAGAATGAGCTTCAGTCATCGCTGGATGTGCAGAAAGAAAGCAGTGgTGTCACgaCAtTGCGACAgCGCAGAAAGCCAGTGTTCCACCTGTCCCACGAGGAGCGtGTCCAACATAGGAAcATTAAAGACCTTAAGCTGGCCTTCaGCGAGTTCTACCTCAGTCTCATCCTGCTGCAGAACGAACCAGAATTTTGCCACcAATATTCATATGGTGTGCGAGCCATTGTTCAATGTATTCCAGCTTGGCTTCGCTTCATCCAGTGCCTGCGCCGGTACCGTGACACGAGGAGGGCTTTTCCTCATCTAGTAAATGCTGGCAAATACTCcACAACTTTCTTCACcGTGACATTTGCAGCCCTTTACAGCACTCACAAACCTCCTCTTTCTCCAGAACAAAAtCACTCTGACACCgtGGTgTTCTTTTACCTGTCT---ACTGCTACAAaGTTTAAGCCTCATGTTGGGGAC

>Musc-CR6-1

GAGAAGCTGGCAGAGGCTCAGCGCAGGTTTGCTACACTTCAGAATGAGCTTCAGTCATCGCTGGATGTGCAGAAAGAAAGCAGTGgTGTCACgaCAtTGCGACAgCGCAGAAAGCCAGTGTTCCACCTGTCCCACGAGGAGCGtGTCCAACATAGGAAcATTAAAGACCTTAAGCTGGCCTTCaGCGAGTTCTACCTCAGTCTCATCCTGCTGCAGAACGAACCAGAATTTTGCCACcAATATTCATATGGTGTGCGAGCCATTGTTCAATGTATTCCAGCTTGGCTTCGCTTCATCCAGTGCCTGCGCCGGTACCGTGACACGAGGAGGGCTTTTCCTCATCTAGTAAATGCTGGCAAATACTCcACAACTTTCTTCACcGTGACATTTGCAGCCCTTTACAGCACTCACAAACCTCCTCTTTCTCCAGAACAAAAtCACTCTGACACCgtGGTgTTCTTTTACCTGTCT---ACTGCTACAAaGTTTAAGCCTCATGTTGGGGAC

>Musc-CR6-2

GAGAAGCTGGCAGAGGCTCAGCGCAGGTTTGCTACACTTCAGAATGAGCTTCAGTCATCGCTGGATGTGCAGAAAGAAAGCAGTGgTGTCACgaCAtTGCGACAgCGCAGAAAGCCAGTGTTCCACCTGTCCCACGAGGAGCGtGTCCAACATAGGAAcATTAAAGACCTTAAGCTGGCCTTCaGCGAGTTCTACCTCAGTCTCATCCTGCTGCAGAACGAACCAGAATTTTGCCACcAATATTCATATGGTGTGCGAGCCATTGTTCAATGTATTCCAGCTTGGCTTCGCTTCATCCAGTGCCTGCGCCGGTACCGTGACACGAGGAGGGCTTTTCCTCATCTAGTAAATGCTGGCAAATACTCcACAACTTTCTTCACcGTGACATTTGCAGCCCTTTACAGCACTCACAAACCTCCTCTTTCTCCAGAACAAAAtCACTCTGACACCgtGGTgTTCTTTTACCTGTCT---ACTGCTACAAaGTTTAAGCCTCATGTTGGGGAC

>Musc-CR5-1

GAGAAGCTGGCAGAGGCTCAGCGCAGGTTTGCTACACTTCAGAATGAGCTTCAGTCATCGCTGGATGTGCAGAAAGAAAGCAGTGgTGTCACgaCAtTGCGACAgCGCAGAAAGCCAGTGTTCCACCTGTCCCACGAGGAGCGtGTCCAACATAGGAAcATTAAAGACCTTAAGCTGGCCTTCaGCGAGTTCTACCTCAGTCTCATCCTGCTGCAGAACGAACCAGAATTTTGCCACcAATATTCATATGGTGTGCGAGCCATTGTTCAATGTATTCCAGCTTGGCTTCGCTTCATCCAGTGCCTGCGCCGGTACCGTGACACGAGGAGGGCTTTTCCTCATCTAGTAAATGCTGGCAAATACTCcACAACTTTCTTCACcGTGACATTTGCAGCCCTTTACAGCACTCACAAACCTCCTCTTTCTCCAGAACAAAAtCACTCTGACACCgtGGTgTTCTTTTACCTGTCT---ACTGCTACAAaGTTTAAGCCTCATGTTGGGGAC

>Musc-CR5-2

GAGAAGCTGGCAGAGGCTCAGCGCAGGTTTGCTACACTTCAGAATGAGCTTCAGTCATCGCTGGATGTGCAGAAAGAAAGCAGTGgTGTCACgaCAtTGCGACAgCGCAGAAAGCCAGTGTTCCACCTGTCCCACGAGGAGCGtGTCCAACATAGGAAcATTAAAGACCTTAAGCTGGCCTTCaGCGAGTTCTACCTCAGTCTCATCCTGCTGCAGAACGAACCAGAATTTTGCCACcAATATTCATATGGTGTGCGAGCCATTGTTCAATGTATTCCAGCTTGGCTTCGCTTCATCCAGTGCCTGCGCCGGTACCGTGACACGAGGAGGGCTTTTCCTCATCTAGTAAATGCTGGCAAATACTCcACAACTTTCTTCACcGTGACATTTGCAGCCCTTTACAGCACTCACAAACCTCCTCTTTCTCCAGAACAAAAtCACTCTGACACCgtGGTgTTCTTTTACCTGTCT---ACTGCTACAAaGTTTAAGCCTCATGTTGGGGAC

>Musc-AL11-1

GAGAAGCTGGCAGAGGCTCAGCGCAGGTTTGCTACACTTCAGAATGAGCTTCAGTCATCGCTGGATGTGCAGAAAGAAAGCAGTGgTGTCACgaCAtTGCGACAgCGCAGAAAGCCAGTGTTCCACCTGTCCCACGAGGAGCGtGTCCAACATAGGAAcATTAAAGACCTTAAGCTGGCCTTCaGCGAGTTCTACCTCAGTCTCATCCTGCTGCAGAACGAACCAGAATTTTGCCACcAATATTCATATGGTGTGCGAGCCATTGTTCAATGTATTCCAGCTTGGCTTCGCTTCATCCAGTGCCTGCGCCGGTACCGTGACACGAGGAGGGCTTTTCCTCATCTAGTAAATGCTGGCAAATACTCcACAACTTTCTTCACcGTGACATTTGCAGCCCTTTACAGCACTCACAAACCTCCTCTTTCTCCAGAACAAAAtCACTCTGACACCatGGTgTTCTTTTACCTGTCT---ACTGCTACAAaGTTTAAGCCTCATGTTGGGGAC

>Musc-AL11-2

GAGAAGCTGGCAGAGGCTCAGCGCAGGTTTGCTACACTTCAGAATGAGCTTCAGTCATCGCTGGATGTGCAGAAAGAAAGCAGTGgTGTCACgaCAtTGCGACAgCGCAGAAAGCCAGTGTTCCACCTGTCCCACGAGGAGCGtGTCCAACATAGGAAcATTAAAGACCTTAAGCTGGCCTTCaGCGAGTTCTACCTCAGTCTCATCCTGCTGCAGAACGAACCAGAATTTTGCCACcAATATTCATATGGTGTGCGAGCCATTGTTCAATGTATTCCAGCTTGGCTTCGCTTCATCCAGTGCCTGCGCCGGTACCGTGACACGAGGAGGGCTTTTCCTCATCTAGTAAATGCTGGCAAATACTCcACAACTTTCTTCACcGTGACATTTGCAGCCCTTTACAGCACTCACAAACCTCCTCTTTCTCCAGAACAAAAtCACTCTGACACCatGGTgTTCTTTTACCTGTCT---ACTGCTACAAaGTTTAAGCCTCATGTTGGGGAC

>Musc-AL1-1

GAGAAGCTGGCAGAGGCTCAGCGCAGGTTTGCTACACTTCAGAATGAGCTTCAGTCATCGCTGGATGTGCAGAAAGAAAGCAGTGgTGTCACgaCAtTGCGACAgCGCAGAAAGCCAGTGTTCCACCTGTCCCACGAGGAGCGtGTCCAACATAGGAAcATTAAAGACCTTAAGCTGGCCTTCaGCGAGTTCTACCTCAGTCTCATCCTGCTGCAGAACGAACCAGAATTTTGCCACcAATATTCATATGGTGTGCGAGCCATTGTTCAATGTATTCCAGCTTGGCTTCGCTTCATCCAGTGCCTGCGCCGGTACCGTGACACGAGGAGGGCTTTTCCTCATCTAGTAAATGCTGGCAAATACTCcACAACTTTCTTCACcGTGACATTTGCAGCCCTTTACAGCACTCACAAACCTCCTCTTTCTCCAGAACAAAAtCACTCTGACACCatGGTgTTCTTTTACCTGTCT---ACTGCTACAAaGTTTAAGCCTCATGTTGGGGAC

>Musc-AL1-2

GAGAAGCTGGCAGAGGCTCAGCGCAGGTTTGCTACACTTCAGAATGAGCTTCAGTCATCGCTGGATGTGCAGAAAGAAAGCAGTGgTGTCACgaCAtTGCGACAgCGCAGAAAGCCAGTGTTCCACCTGTCCCACGAGGAGCGtGTCCAACATAGGAAcATTAAAGACCTTAAGCTGGCCTTCaGCGAGTTCTACCTCAGTCTCATCCTGCTGCAGAACGAACCAGAATTTTGCCACcAATATTCATATGGTGTGCGAGCCATTGTTCAATGTATTCCAGCTTGGCTTCGCTTCATCCAGTGCCTGCGCCGGTACCGTGACACGAGGAGGGCTTTTCCTCATCTAGTAAATGCTGGCAAATACTCcACAACTTTCTTCACcGTGACATTTGCAGCCCTTTACAGCACTCACAAACCTCCTCTTTCTCCAGAACAAAAtCACTCTGACACCatGGTgTTCTTTTACCTGTCT---ACTGCTACAAaGTTTAAGCCTCATGTTGGGGAC

>Musc-AL6-1

GAGAAGCTGGCAGAGGCTCAGCGCAGGTTTGCTACACTTCAGAATGAGCTTCAGTCATCGCTGGATGTGCAGAAAGAAAGCAGTGgTGTCACgaCAtTGCGACAgCGCAGAAAGCCAGTGTTCCACCTGTCCCACGAGGAGCGtGTCCAACATAGGAAcATTAAAGACCTTAAGCTGGCCTTCaGCGAGTTCTACCTCAGTCTCATCCTGCTGCAGAACGAACCAGAATTTTGCCACcAATATTCATATGGTGTGCGAGCCATTGTTCAATGTATTCCAGCTTGGCTTCGCTTCATCCAGTGCCTGCGCCGGTACCGTGACACGAGGAGGGCTTTTCCTCATCTAGTAAATGCTGGCAAATACTCcACAACTTTCTTCACcGTGACATTTGCAGCCCTTTACAGCACTCACAAACCTCCTCTTTCTCCAGAACAAAAtCACTCTGACACCatGGTgTTCTTTTACCTGTCT---ACTGCTACAAaGTTTAAGCCTCATGTTGGGGAC

>Musc-AL6-2

GAGAAGCTGGCAGAGGCTCAGCGCAGGTTTGCTACACTTCAGAATGAGCTTCAGTCATCGCTGGATGTGCAGAAAGAAAGCAGTGgTGTCACgaCAtTGCGACAgCGCAGAAAGCCAGTGTTCCACCTGTCCCACGAGGAGCGtGTCCAACATAGGAAcATTAAAGACCTTAAGCTGGCCTTCaGCGAGTTCTACCTCAGTCTCATCCTGCTGCAGAACGAACCAGAATTTTGCCACcAATATTCATATGGTGTGCGAGCCATTGTTCAATGTATTCCAGCTTGGCTTCGCTTCATCCAGTGCCTGCGCCGGTACCGTGACACGAGGAGGGCTTTTCCTCATCTAGTAAATGCTGGCAAATACTCcACAACTTTCTTCACcGTGACATTTGCAGCCCTTTACAGCACTCACAAACCTCCTCTTTCTCCAGAACAAAAtCACTCTGACACCatGGTgTTCTTTTACCTGTCT---ACTGCTACAAaGTTTAAGCCTCATGTTGGGGAC

>Musc-AL15-1

GAGAAGCTGGCAGAGGCTCAGCGCAGGTTTGCTACACTTCAGAATGAGCTTCAGTCATCGCTGGATGTGCAGAAAGAAAGCAGTGgTGTCACgaCAtTGCGACAgCGCAGAAAGCCAGTGTTCCACCTGTCCCACGAGGAGCGtGTCCAACATAGGAAcATTAAAGACCTTAAGCTGGCCTTCaGCGAGTTCTACCTCAGTCTCATCCTGCTGCAGAACGAACCAGAATTTTGCCACcAATATTCATATGGTGTGCGAGCCATTGTTCAATGTATTCCAGCTTGGCTTCGCTTCATCCAGTGCCTGCGCCGGTACCGTGACACGAGGAGGGCTTTTCCTCATCTAGTAAATGCTGGCAAATACTCcACAACTTTCTTCACcGTGACATTTGCAGCCCTTTACAGCACTCACAAACCTCCTCTTTCTCCAGAACAAAAtCACTCTGACACCatGGTgTTCTTTTACCTGTCT---ACTGCTACAAaGTTTAAGCCTCATGTTGGGGAC

>Musc-AL15-2

GAGAAGCTGGCAGAGGCTCAGCGCAGGTTTGCTACACTTCAGAATGAGCTTCAGTCATCGCTGGATGTGCAGAAAGAAAGCAGTGgTGTCACgaCAtTGCGACAgCGCAGAAAGCCAGTGTTCCACCTGTCCCACGAGGAGCGtGTCCAACATAGGAAcATTAAAGACCTTAAGCTGGCCTTCaGCGAGTTCTACCTCAGTCTCATCCTGCTGCAGAACGAACCAGAATTTTGCCACcAATATTCATATGGTGTGCGAGCCATTGTTCAATGTATTCCAGCTTGGCTTCGCTTCATCCAGTGCCTGCGCCGGTACCGTGACACGAGGAGGGCTTTTCCTCATCTAGTAAATGCTGGCAAATACTCcACAACTTTCTTCACcGTGACATTTGCAGCCCTTTACAGCACTCACAAACCTCCTCTTTCTCCAGAACAAAAtCACTCTGACACCatGGTgTTCTTTTACCTGTCT---ACTGCTACAAaGTTTAAGCCTCATGTTGGGGAC

>Musc-AL13-1

GAGAAGCTGGCAGAGGCTCAGCGCAAGTTTGCTACACTTCAGAATGAGCTTCAGTCATCGCTGGATGTGCAGAAAGAAAGCAGTGgTGTCACgaCAtTGCGACAgCGCAGAAAGCCAGTGTTCCACCTGTCCCACGAGGAGCGtGTCCAACATAGGAAcATTAAAGACCTTAAGCTGGCCTTCaGCGAGTTCTACCTCAGTCTCATCCTGCTGCAGAACGAACCAGAATTTTGCCACcAATATTCATATGGTGTGCGAGCCATTGTTCAATGTATTCCAGCTTGGCTTCGCTTCATCCAGTGCCTGCGCCGGTACCGTGACACGAGGAGGGCTTTTCCTCATCTAGTAAATGCTGGCAAATACTCcACAACTTTCTTCACcGTGACATTTGCAGCCCTTTACAGCACTCACAAACCTCCTCTTTCTCCAGAACAAAAtCACTCTGACACCatGGTgTTCTTTTACCTGTCT---ACTGCTACAAaGTTTAAGCCTCATGTTGGGGAC

>Musc-AL13-2

GAGAAGCTGGCAGAGGCTCAGCGCAGGTTTGCTACACTTCAGAATGAGCTTCAGTCATCGCTGGATGTGCAGAAAGAAAGCAGTGgTGTCACgaCAtTGCGACAgCGCAGAAAGCCAGTGTTCCACCTGTCCCACGAGGAGCGtGTCCAACATAGGAAcATTAAAGACCTTAAGCTGGCCTTCaGCGAGTTCTACCTCAGTCTCATCCTGCTGCAGAACGAACCAGAATTTTGCCACcAATATTCATATGGTGTGCGAGCCATTGTTCAATGTATTCCAGCTTGGCTTCGCTTCATCCAGTGCCTGCGCCGGTACCGTGACACGAGGAGGGCTTTTCCTCATCTAGTAAATGCTGGCAAATACTCcACAACTTTCTTCACcGTGACATTTGCAGCCCTTTACAGCACTCACAAACCTCCTCTTTCTCCAGAACAAAAtCACTCTGACACCatGGTgTTCTTTTACCTGTCT---ACTGCTACAAaGTTTAAGCCTCATGTTGGGGAC

>Musc-AL2-1

GAGAAGCTGGCAGAGGCTCAGCGCAGGTTTGCTACACTTCAGAATGAGCTTCAGTCATCGCTGGATGTGCAGAAAGAAAGCAGTGgTGTCACgaCAtTGCGACAgCGCAGAAAGCCAGTGTTCCACCTGTCCCACGAGGAGCGtGTCCAACATAGGAAcATTAAAGACCTTAAGCTGGCCTTCaGCGAGTTCTACCTCAGTCTCATCCTGCTGCAGAACGAACCAGAATTTTGCCACcAATATTCATATGGTGTGCGAGCCATTGTTCAATGTATTCCAGCTTGGCTTCGCTTCATCCAGTGCCTGCGCCGGTACCGTGACACGAGGAGGGCTTTTCCTCATCTAGTAAATGCTGGCAAATACTCcACAACTTTCTTCACcGTGACATTTGCAGCCCTTTACAGCACTCACAAACCTCCTCTTTCTCCAGAACAAAAtCACTCTGACACCatGGTgTTCTTTTACCTGTCT---ACTGCTACAAaGTTTAAGCCTCATGTTGGGGAC

>Musc-AL2-2

GAGAAGCTGGCAGAGGCTCAGCGCAGGTTTGCTACACTTCAGAATGAGCTTCAGTCATCGCTGGATGTGCAGAAAGAAAGCAGTGgTGTCACgaCAtTGCGACAgCGCAGAAAGCCAGTGTTCCACCTGTCCCACGAGGAGCGtGTCCAACATAGGAAcATTAAAGACCTTAAGCTGGCCTTCaGCGAGTTCTACCTCAGTCTCATCCTGCTGCAGAACGAACCAGAATTTTGCCACcAATATTCATATGGTGTGCGAGCCATTGTTCAATGTATTCCAGCTTGGCTTCGCTTCATCCAGTGCCTGCGCCGGTACCGTGACACGAGGAGGGCTTTTCCTCATCTAGTAAATGCTGGCAAATACTCcACAACTTTCTTCACcGTGACATTTGCAGCCCTTTACAGCACTCACAAACCTCCTCTTTCTCCAGAACAAAAtCACTCTGACACCgtGGTgTTCTTTTACCTGTCT---ACTGCTACAAaGTTTAAGCCTCATGTTGGGGAC

>Musc-AL3-1

GAGAAGCTGGCAGAGGCTCAGCGCAGGTTTGCTACACTTCAGAATGAGCTTCAGTCATCGCTGGATGTGCAGAAAGAAAGCAGTGgTGTCACgaCAtTGCGACAgCGCAGAAAGCCAGTGTTCCACCTGTCCCACGAGGAGCGtGTCCAACATAGGAAcATTAAAGACCTTAAGCTGGCCTTCaGCGAGTTCTACCTCAGTCTCATCCTGCTGCAGAACGAACCAGAATTTTGCCACcAATATTCATATGGTGTGCGAGCCATTGTTCAATGTATTCCAGCTTGGCTTCGCTTCATCCAGTGCCTGCGCCGGTACCGTGACACGAGGAGGGCTTTTCCTCATCTAGTAAATGCTGGCAAATACTCcACAACTTTCTTCACcGTGACATTTGCAGCCCTTTACAGCACTCACAAACCTCCTCTTTCTCCAGAACAAAAtCACTCTGACACCatGGTgTTCTTTTACCTGTCT---ACTGCTACAAaGTTTAAGCCTCATGTTGGGGAC

>Musc-AL3-2

GAGAAGCTGGCAGAGGCTCAGCGCAGGTTTGCTACACTTCAGAATGAGCTTCAGTCATCGCTGGATGTGCAGAAAGAAAGCAGTGgTGTCACgaCAtTGCGACAgCGCAGAAAGCCAGTGTTCCACCTGTCCCACGAGGAGCGtGTCCAACATAGGAAcATTAAAGACCTTAAGCTGGCCTTCaGCGAGTTCTACCTCAGTCTCATCCTGCTGCAGAACGAACCAGAATTTTGCCACcAATATTCATATGGTGTGCGAGCCATTGTTCAATGTATTCCAGCTTGGCTTCGCTTCATCCAGTGCCTGCGCCGGTACCGTGACACGAGGAGGGCTTTTCCTCATCTAGTAAATGCTGGCAAATACTCcACAACTTTCTTCACcGTGACATTTGCAGCCCTTTACAGCACTCACAAACCTCCTCTTTCTCCAGAACAAAAtCACTCTGACACCatGGTgTTCTTTTACCTGTCT---ACTGCTACAAaGTTTAAGCCTCATGTTGGGGAC

>Musc-AL4-1

GAGAAGCTGGCAGAGGCTCAGCGCAGGTTTGCTACACTTCAGAATGAGCTTCAGTCATCGCTGGATGTGCAGAAAGAAAGCAGTGgTGTCACgaCAtTGCGACAgCGCAGAAAGCCAGTGTTCCACCTGTCCCACGAGGAGCGtGTCCAACATAGGAAcATTAAAGACCTTAAGCTGGCCTTCaGCGAGTTCTACCTCAGTCTCATCCTGCTGCAGAACGAACCAGAATTTTGCCACcAATATTCATATGGTGTGCGAGCCATTGTTCAATGTATTCCAGCTTGGCTTCGCTTCATCCAGTGCCTGCGCCGGTACCGTGACACGAGGAGGGCTTTTCCTCATCTAGTAAATGCTGGCAAATACTCcACAACTTTCTTCACcGTGACATTTGCAGCCCTTTACAGCACTCACAAACCTCCTCTTTCTCCAGAACAAAAtCACTCTGACACCatGGTgTTCTTTTACCTGTCT---ACTGCTACAAaGTTTAAGCCTCATGTTGGGGAC

>Musc-AL4-2

GAGAAGCTGGCAGAGGCTCAGCGCAGGTTTGCTACACTTCAGAATGAGCTTCAGTCATCGCTGGATGTGCAGAAAGAAAGCAGTGgTGTCACgaCAtTGCGACAgCGCAGAAAGCCAGTGTTCCACCTGTCCCACGAGGAGCGtGTCCAACATAGGAAcATTAAAGACCTTAAGCTGGCCTTCaGCGAGTTCTACCTCAGTCTCATCCTGCTGCAGAACGAACCAGAATTTTGCCACcAATATTCATATGGTGTGCGAGCCATTGTTCAATGTATTCCAGCTTGGCTTCGCTTCATCCAGTGCCTGCGCCGGTACCGTGACACGAGGAGGGCTTTTCCTCATCTAGTAAATGCTGGCAAATACTCcACAACTTTCTTCACcGTGACATTTGCAGCCCTTTACAGCACTCACAAACCTCCTCTTTCTCCAGAACAAAAtCACTCTGACACCatGGTgTTCTTTTACCTGTCT---ACTGCTACAAaGTTTAAGCCTCATGTTGGGGAC

>Musc-AL9-1

GAGAAGCTGGCAGAGGCTCAGCGCAGGTTTGCTACACTTCAGAATGAGCTTCAGTCATCGCTGGATGTGCAGAAAGAAAGCAGTGgTGTCACgaCAtTGCGACAgCGCAGAAAGCCAGTGTTCCACCTGTCCCACGAGGAGCGtGTCCAACATAGGAAcATTAAAGACCTTAAGCTGGCCTTCaGCGAGTTCTACCTCAGTCTCATCCTGCTGCAGAACGAACCAGAATTTTGCCACcAATATTCATATGGTGTGCGAGCCATTGTTCAATGTATTCCAGCTTGGCTTCGCTTCATCCAGTGCCTGCGCCGGTACCGTGACACGAGGAGGGCTTTTCCTCATCTAGTAAATGCTGGCAAATACTCcACAACTTTCTTCACcGTGACATTTGCAGCCCTTTACAGCACTCACAAACCTCCTCTTTCTCCAGAACAAAAtCACTCTGACACCatGGTgTTCTTTTACCTGTCT---ACTGCTACAAaGTTTAAGCCTCATGTTGGGGAC

>Musc-AL9-2

GAGAAGCTGGCAGAGGCTCAGCGCAGGTTTGCTACACTTCAGAATGAGCTTCAGTCATCGCTGGATGTGCAGAAAGAAAGCAGTGgTGTCACgaCAtTGCGACAgCGCAGAAAGCCAGTGTTCCACCTGTCCCACGAGGAGCGtGTCCAACATAGGAAcATTAAAGACCTTAAGCTGGCCTTCaGCGAGTTCTACCTCAGTCTCATCCTGCTGCAGAACGAACCAGAATTTTGCCACcAATATTCATATGGTGTGCGAGCCATTGTTCAATGTATTCCAGCTTGGCTTCGCTTCATCCAGTGCCTGCGCCGGTACCGTGACACGAGGAGGGCTTTTCCTCATCTAGTAAATGCTGGCAAATACTCcACAACTTTCTTCACcGTGACATTTGCAGCCCTTTACAGCACTCACAAACCTCCTCTTTCTCCAGAACAAAAtCACTCTGACACCatGGTgTTCTTTTACCTGTCT---ACTGCTACAAaGTTTAAGCCTCATGTTGGGGAC

>Musc-AL14-1

GAGAAGCTGGCAGAGGCTCAGCGCAGGTTTGCTACACTTCAGAATGAGCTTCAGTCATCGCTGGATGTGCAGAAAGAAAGCAGTGgTGTCACgaCAtTGCGACAgCGCAGAAAGCCAGTGTTCCACCTGTCCCACGAGGAGCGtGTCCAACATAGGAAcATTAAAGACCTTAAGCTGGCCTTCaGCGAGTTCTACCTCAGTCTCATCCTGCTGCAGAACGAACCAGAATTTTGCCACcAATATTCATATGGTGTGCGAGCCATTGTTCAATGTATTCCAGCTTGGCTTCGCTTCATCCAGTGCCTGCGCCGGTACCGTGACACGAGGAGGGCTTTTCCTCATCTAGTAAATGCTGGCAAATACTCcACAACTTTCTTCACcGTGACATTTGCAGCCCTTTACAGCACTCACAAACCTCCTCTTTCTCCAGAACAAAAtCACTCTGACACCatGGTgTTCTTTTACCTGTCT---ACTGCTACAAaGTTTAAGCCTCATGTTGGGGAC

>Musc-AL14-2

GAGAAGCTGGCAGAGGCTCAGCGCAGGTTTGCTACACTTCAGAATGAGCTTCAGTCATCGCTGGATGTGCAGAAAGAAAGCAGTGgTGTCACgaCAtTGCGACAgCGCAGAAAGCCAGTGTTCCACCTGTCCCACGAGGAGCGtGTCCAACATAGGAAcATTAAAGACCTTAAGCTGGCCTTCaGCGAGTTCTACCTCAGTCTCATCCTGCTGCAGAACGAACCAGAATTTTGCCACcAATATTCATATGGTGTGCGAGCCATTGTTCAATGTATTCCAGCTTGGCTTCGCTTCATCCAGTGCCTGCGCCGGTACCGTGACACGAGGAGGGCTTTTCCTCATCTAGTAAATGCTGGCAAATACTCcACAACTTTCTTCACcGTGACATTTGCAGCCCTTTACAGCACTCACAAACCTCCTCTTTCTCCAGAACAAAAtCACTCTGACACCatGGTgTTCTTTTACCTGTCT---ACTGCTACAAaGTTTAAGCCTCATGTTGGGGAC

>Musc-AL10-1

GAGAAGCTGGCAGAGGCTCAGCGCAGGTTTGCTACACTTCAGAATGAGCTTCAGTCATCGCTGGATGTGCAGAAAGAAAGCAGTGgTGTCACgaCAtTGCGACAgCGCAGAAAGCCAGTGTTCCACCTGTCCCACGAGGAGCGtGTCCAACATAGGAAcATTAAAGACCTTAAGCTGGCCTTCaGCGAGTTCTACCTCAGTCTCATCCTGCTGCAGAACGAACCAGAATTTTGCCACcAATATTCATATGGTGTGCGAGCCATTGTTCAATGTATTCCAGCTTGGCTTCGCTTCATCCAGTGCCTGCGCCGGTACCGTGACACGAGGAGGGCTTTTCCTCATCTAGTAAATGCTGGCAAATACTCcACAACTTTCTTCACcGTGACATTTGCAGCCCTTTACAGCACTCACAAACCTCCTCTTTCTCCAGAACAAAAtCACTCTGACACCatGGTgTTCTTTTACCTGTCT---ACTGCTACAAaGTTTAAGCCTCATGTTGGGGAC

>Musc-AL10-2

GAGAAGCTGGCAGAGGCTCAGCGCAGGTTTGCTACACTTCAGAATGAGCTTCAGTCATCGCTGGATGTGCAGAAAGAAAGCAGTGgTGTCACgaCAtTGCGACAgCGCAGAAAGCCAGTGTTCCACCTGTCCCACGAGGAGCGtGTCCAACATAGGAAcATTAAAGACCTTAAGCTGGCCTTCaGCGAGTTCTACCTCAGTCTCATCCTGCTGCAGAACGAACCAGAATTTTGCCACcAATATTCATATGGTGTGCGAGCCATTGTTCAATGTATTCCAGCTTGGCTTCGCTTCATCCAGTGCCTGCGCCGGTACCGTGACACGAGGAGGGCTTTTCCTCATCTAGTAAATGCTGGCAAATACTCcACAACTTTCTTCACcGTGACATTTGCAGCCCTTTACAGCACTCACAAACCTCCTCTTTCTCCAGAACAAAAtCACTCTGACACCgtGGTgTTCTTTTACCTGTCT---ACTGCTACAAaGTTTAAGCCTCATGTTGGGGAC

>Musc-AL7-1

GAGAAGCTGGCAGAGGCTCAGCGCAGGTTTGCTACACTTCAGAATGAGCTTCAGTCATCGCTGGATGTGCAGAAAGAAAGCAGTGgTGTCACgaCAtTGCGACAgCGCAGAAAGCCAGTGTTCCACCTGTCCCACGAGGAGCGtGTCCAACATAGGAAcATTAAAGACCTTAAGCTGGCCTTCaGCGAGTTCTACCTCAGTCTCATCCTGCTGCAGAACGAACCAGAATTTTGCCACcAATATTCATATGGTGTGCGAGCCATTGTTCAATGTATTCCAGCTTGGCTTCGCTTCATCCAGTGCCTGCGCCGGTACCGTGACACGAGGAGGGCTTTTCCTCATCTAGTAAATGCTGGCAAATACTCcACAACTTTCTTCACcGTGACATTTGCAGCCCTTTACAGCACTCACAAACCTCCTCTTTCTCCAGAACAAAAtCACTCTGACACCatGGTgTTCTTTTACCTGTCT---ACTGCTACAAaGTTTAAGCCTCATGTTGGGGAC

>Musc-AL7-2

GAGAAGCTGGCAGAGGCTCAGCGCAGGTTTGCTACACTTCAGAATGAGCTTCAGTCATCGCTGGATGTGCAGAAAGAAAGCAGTGgTGTCACgaCAtTGCGACAgCGCAGAAAGCCAGTGTTCCACCTGTCCCACGAGGAGCGtGTCCAACATAGGAAcATTAAAGACCTTAAGCTGGCCTTCaGCGAGTTCTACCTCAGTCTCATCCTGCTGCAGAACGAACCAGAATTTTGCCACcAATATTCATATGGTGTGCGAGCCATTGTTCAATGTATTCCAGCTTGGCTTCGCTTCATCCAGTGCCTGCGCCGGTACCGTGACACGAGGAGGGCTTTTCCTCATCTAGTAAATGCTGGCAAATACTCcACAACTTTCTTCACcGTGACATTTGCAGCCCTTTACAGCACTCACAAACCTCCTCTTTCTCCAGAACAAAAtCACTCTGACACCatGGTgTTCTTTTACCTGTCT---ACTGCTACAAaGTTTAAGCCTCATGTTGGGGAC

>Cast-TAI2967-1

GAGAAGCTGGCAGAGGCTCAGCGCAGGTTTGCTACACTTCAGAATGAGCTTCAGTCATCGCTGGATGTGCAGAAAGAAAGCAGTGgTGTCACagCAtTGCGACAgCGCAGAAAGCCAGTGTTCCACCTGTCCCACGAGGAGCGcGTCCAACATAGGAAcATTAAAGACCTTAAGCTGGCCTTCaGCGAGTTCTACCTCAGTCTCATCCTGCTGCAGAACGAACCAGAATTTTGCCACaAATATTCATATGGTGTGCGAGCCATTGTTCAATGTATTCCAGCTTGGCTTCGCTTCATCCAGTGCCTGCGCCGGTACCGTGACACGAGGAGGGCTTTTCCTCATCTAGTAAATGCTGGCAAATACTCcACAACTTTCTTCACcGTGACATTTGCAGCCCTTTACAGCACTCACAAACCTCCTCTTTCTCCAGAACAAAAtCACTCTGACACCgtGGTgTTCTTTTACCTGTCTATTACTGCT---------------CATGTTGGGGAC

>Cast-TAI2967-2

GAGAAGCTGGCAGAGGCTCAGCGCAGGTTTGCTACACTTCAGAATGAGCTTCAGTCATCGCTGGATGTGCAGAAAGAAAGCAGTGgTGTCACagCAtTGCGACAgCGCAGAAAGCCAGTGTTCCACCTGTCCCACGAGGAGCGcGTCCAACATAGGAAcATTAAAGACCTTAAGCTGGCCTTCaGCGAGTTCTACCTCAGTCTCATCCTGCTGCAGAACGAACCAGAATTTTGCCACaAATATTCATATGGTGTGCGAGCCATTGTTCAATGTATTCCAGCTTGGCTTCGCTTCATCCAGTGCCTGCGCCGGTACCGTGACACGAGGAGGGCTTTTCCTCATCTAGTAAATGCTGGCAAATACTCcACAACTTTCTTCACcGTGACATTTGCAGCCCTTTACAGCACTCACAAACCTCCTCTTTCTCCAGAACAAAAtCACTCTGACACCgtGGTgTTCTTTTACCTGTCTATTACTGCT---------------CATGTTGGGGAC

>Cast-TAI2971-1

GAGAAGCTGGCAGAGGCTCAGCGCAGGTTTGCTACACTTCAGAATGAGCTTCAGTCATCGCTGGATGTGCAGAAAGAAAGCAGTGgTGTCACagCAtTGCGACAgCGCAGAAAGCCAGTGTTCCACCTGTCCCACGAGGAGCGcGTCCAACATAGGAAcATTAAAGACCTTAAGCTGGCCTTCaGCGAGTTCTACCTCAGTCTCATCCTGCTGCAGAACGAACCAGAATTTTGCCACaAATATTCATATGGTGTGCGAGCCATTGTTCAATGTATTCCAGCTTGGCTTCGCTTCATCCAGTGCCTGCGCCGGTACCGTGACACGAGGAGGGCTTTTCCTCATCTAGTAAATGCTGGCAAATACTCcACAACTTTCTTCACcGTGACATTTGCAGCCCTTTACAGCACTCACAAACCTCCTCTTTCTCCAGAACAAAAtCACTCTGACACCgtGGTgTTCTTTTACCTGTCTATTACTGCT---------------CATGTTGGGGAC

>Cast-TAI2971-2

GAGAAGCTGGCAGAGGCTCAGCGCAGGTTTGCTACACTTCAGAATGAGCTTCAGTCATCGCTGGATGTGCAGAAAGAAAGCAGTGgTGTCACagCAtTGCGACAgCGCAGAAAGCCAGTGTTCCACCTGTCCCACGAGGAGCGcGTCCAACATAGGAAcATTAAAGACCTTAAGCTGGCCTTCaGCGAGTTCTACCTCAGTCTCATCCTGCTGCAGAACGAACCAGAATTTTGCCACaAATATTCATATGGTGTGCGAGCCATTGTTCAATGTATTCCAGCTTGGCTTCGCTTCATCCAGTGCCTGCGCCGGTACCGTGACACGAGGAGGGCTTTTCCTCATCTAGTAAATGCTGGCAAATACTCcACAACTTTCTTCACcGTGACATTTGCAGCCCTTTACAGCACTCACAAACCTCCTCTTTCTCCAGAACAAAAtCACTCTGACACCgtGGTgTTCTTTTACCTGTCTATTACTGCT---------------CATGTTGGGGAC

>Scl100-1

GAGAAGCTGGCAGAGGCTCAGCGCAGGTTTGCTACACTTCAGAATGAGCTTCAGTCATCGCTGGATGTGCAGAAAGAAAGCAGTGgTGTCACaaCAtTGCGACAaCGCAGAAAGCCAGTGTTCCACCTGTCCCACGAGGAGCGtGTCCAACATAGGAAtATTAAAGACCTTAAGCTGGCCTTCaGCGAGTTCTACCTCAGTCTCATCCTGCTGCAGAACGAACCAGAATTTTGCCACaAATATTCATATGGTGTGCGAGCCATTGTTCAATGTATTCCAGCTTGGCTTCGCTTCATCCAGTGCCTGCGCCGGTACCGTGACACGAGGAGGGCTTTTCCTCATCTAGTAAATGCTGGCAAATACTCcACAACTTTCTTCACcGTGACATTTGCAGCCCTTTACAGCACTCACAAACCTCCTCTTTCTCCAGAACAAAAtCACTCTGACACCgcGGTgTTCTTTTACCTGTCTATTACTGCTACAACGTTTAAGCCTCATGTTGGGGAC

>Scl100-2

GAGAAGCTGGCAGAGGCTCAGCGCAGGTTTGCTACACTTCAGAATGAGCTTCAGTCATCGCTGGATGTGCAGAAAGAAAGCAGTGgTGTCACaaCAtTGCGACAaCGCAGAAAGCCAGTGTTCCACCTGTCCCACGAGGAGCGtGTCCAACATAGGAAtATTAAAGACCTTAAGCTGGCCTTCaGCGAGTTCTACCTCAGTCTCATCCTGCTGCAGAACGAACCAGAATTTTGCCACaAATATTCATATGGTGTGCGAGCCATTGTTCAATGTATTCCAGCTTGGCTTCGCTTCATCCAGTGCCTGCGCCGGTACCGTGACACGAGGAGGGCTTTTCCTCATCTAGTAAATGCTGGCAAATACTCcACAACTTTCTTCACcGTGACATTTGCAGCCCTTTACAGCACTCACAAACCTCCTCTTTCTCCAGAACAAAAtCACTCTGACACCgcGGTgTTCTTTTACCTGTCTATTACTGCTACAACGTTTAAGCCTCATGTTGGGGAC

>Scl103-1

GAGAAGCTGGCAGAGGCTCAGCGCAGGTTTGCTACACTTCAGAATGAGCTTCAGTCATCGCTGGATGTGCAGAAAGAAAGCAGTGgTGTCACaaCAtTGCGACAaCGCAGAAAGCCAGTGTTCCACCTGTCCCACGAGGAGCGtGTCCAACATAGGAAtATTAAAGACCTTAAGCTGGCCTTCaGCGAGTTCTACCTCAGTCTCATCCTGCTGCAGAACGAACCAGAATTTTGCCACaAATATTCATATGGTGTGCGAGCCATTGTTCAATGTATTCCAGCTTGGCTTCGCTTCATCCAGTGCCTGCGCCGGTACCGTGACACGAGGAGGGCTTTTCCTCATCTAGTAAATGCTGGCAAATACTCcACAACTTTCTTCACcGTGACATTTGCAGCCCTTTACAGCACTCACAAACCTCCTCTTTCTCCAGAACAAAAtCACTCTGACACCgcGGTgTTCTTTTACCTGTCTATTACTGCTACAACGTTTAAGCCTCATGTTGGGGAC

>Scl103-2

GAGAAGCTGGCAGAGGCTCAGCGCAGGTTTGCTACACTTCAGAATGAGCTTCAGTCATCGCTGGATGTGCAGAAAGAAAGCAGTGgTGTCACaaCAtTGCGACAaCGCAGAAAGCCAGTGTTCCACCTGTCCCACGAGGAGCGtGTCCAACATAGGAAtATTAAAGACCTTAAGCTGGCCTTCaGCGAGTTCTACCTCAGTCTCATCCTGCTGCAGAACGAACCAGAATTTTGCCACaAATATTCATATGGTGTGCGAGCCATTGTTCAATGTATTCCAGCTTGGCTTCGCTTCATCCAGTGCCTGCGCCGGTACCGTGACACGAGGAGGGCTTTTCCTCATCTAGTAAATGCTGGCAAATACTCcACAACTTTCTTCACcGTGACATTTGCAGCCCTTTACAGCACTCACAAACCTCCTCTTTCTCCAGAACAAAAtCACTCTGACACCgcGGTgTTCTTTTACCTGTCTATTACTGCTACAACGTTTAAGCCTCATGTTGGGGAC

>SP2723-1

GAGAAGCTGGCAGAGGCTCAGCGCAGGTTTGCTACACTTCAGAATGAGCTTCAGTCATCGCTGGATGTGCAGAAAGAAAGCAGTGgTGTCACaaCAtTGCGACAaCGCAGAAAGCCAGTGTTCCACCTGTCCCACGAGGAGCGtGTCCAACATAGGAAtATTAAAGACCTTAAGCTGGCCTTCaGCGAGTTCTACCTCAGTCTCATCCTGCTGCAGAACGAACCAGAATTTTGCCACaAATATTCATATGGTGTGCGAGCCATTGTTCAATGTATTCCAGCTTGGCTTCGCTTCATCCAGTGCCTGCGCCGGTACCGTGACACGAGGAGGGCTTTTCCTCATCTAGTAAATGCTGGCAAATACTCtACAACTTTCTTCACcGTGACATTTGCAGCCCTTTACAGCACTCACAAACCTCCTCTTTCTCCAGAACAAAAtCACTCTGACACCgtGGTaTTCTTTTACCTGTCTATTACTGCTACAACGTTTAAGCCTCATGTTGGGGAC

>SP2723-2

GAGAAGCTGGCAGAGGCTCAGCGCAGGTTTGCTACACTTCAGAATGAGCTTCAGTCATCGCTGGATGTGCAGAAAGAAAGCAGTGgTGTCACaaCAtTGCGACAaCGCAGAAAGCCAGTGTTCCACCTGTCCCACGAGGAGCGtGTCCAACATAGGAAtATTAAAGACCTTAAGCTGGCCTTCaGCGAGTTCTACCTCAGTCTCATCCTGCTGCAGAACGAACCAGAATTTTGCCACaAATATTCATATGGTGTGCGAGCCATTGTTCAATGTATTCCAGCTTGGCTTCGCTTCATCCAGTGCCTGCGCCGGTACCGTGACACGAGGAGGGCTTTTCCTCATCTAGTAAATGCTGGCAAATACTCtACAACTTTCTTCACcGTGACATTTGCAGCCCTTTACAGCACTCACAAACCTCCTCTTTCTCCAGAACAAAAtCACTCTGACACCgtGGTaTTCTTTTACCTGTCTATTACTGCTACAACGTTTAAGCCTCATGTTGGGGAC

>SP2729-1

GAGAAGCTGGCAGAGGCTCAGCGCAGGTTTGCTACACTTCAGAATGAGCTTCAGTCATCGCTGGATGTGCAGAAAGAAAGCAGTGgTGTCACaaCAtTGCGACAaCGCAGAAAGCCAGTGTTCCACCTGTCCCACGAGGAGCGtGTCCAACATAGGAAtATTAAAGACCTTAAGCTGGCCTTCaGCGAGTTCTACCTCAGTCTCATCCTGCTGCAGAACGAACCAGAATTTTGCCACaAATATTCATATGGTGTGCGAGCCATTGTTCAATGTATTCCAGCTTGGCTTCGCTTCATCCAGTGCCTGCGCCGGTACCGTGACACGAGGAGGGCTTTTCCTCATCTAGTAAATGCTGGCAAATACTCtACAACTTTCTTCACcGTGACATTTGCAGCCCTTTACAGCACTCACAAACCTCCTCTTTCTCCAGAACAAAAtCACTCTGACACCgtGGTaTTCTTTTACCTGTCTATTACTGCTACAACGTTTAAGCCTCATGTTGGGGAC

>SP2729-2

GAGAAGCTGGCAGAGGCTCAGCGCAGGTTTGCTACACTTCAGAATGAGCTTCAGTCATCGCTGGATGTGCAGAAAGAAAGCAGTGgTGTCACaaCAtTGCGACAaCGCAGAAAGCCAGTGTTCCACCTGTCCCACGAGGAGCGtGTCCAACATAGGAAtATTAAAGACCTTAAGCTGGCCTTCaGCGAGTTCTACCTCAGTCTCATCCTGCTGCAGAACGAACCAGAATTTTGCCACaAATATTCATATGGTGTGCGAGCCATTGTTCAATGTATTCCAGCTTGGCTTCGCTTCATCCAGTGCCTGCGCCGGTACCGTGACACGAGGAGGGCTTTTCCTCATCTAGTAAATGCTGGCAAATACTCtACAACTTTCTTCACcGTGACATTTGCAGCCCTTTACAGCACTCACAAACCTCCTCTTTCTCCAGAACAAAAtCACTCTGACACCgtGGTaTTCTTTTACCTGTCTATTACTGCTACAACGTTTAAGCCTCATGTTGGGGAC

**RBD Sequences of P-MLV variants from the population samples**

>10007037_IraAH

ACATGACAGCCCTCATCAGGTCTTCAATGTTACTTGGAGAGTTACCAACTTAATGACAGGACAAACAGCTAATGCTACCTCCCTCCTGGGGACAATGACCGATGCCTTTCCTAAACTGTACTTTGACTTGTGCGATTTAATAGGGGACGACTGGGATGAGACTGGACTCGGGTGTCGCACTCCCGGGGGAAGAAAAAGGKCAAGAAYATTTGACTTCTATGTTTGCCCCGGGCATACTGTACCAACAGGGTGTGGAGGGCCGAGAGAGGGCTACTGTGGCAAATGGGGCTGTGAGACCACTGGACAGGCATACTGRAAGCCATCATCATCATGGGACCTAATTTCCCTTAAGCGAGGAAACACCCCTCGGAATCAGGGCCCCTGTTATGATTCCTCAGCGGTCTCCAGTGACATCCAGGGCGCCACACCGGGGGGTCGATGCAATCCCCTAGTCCTAGAATTCACTGACGCGGGCAAAAAGGCCAGCTGGGATGGCCCCAAAGTATGGGGACTAAGACTGTACCGACCCACAGGGACCGACCCGGTGACCCGGTTCTCTTTGACCCGCCAGG

>10007040_IraAH

ACATGACAGCCCTCATCAGGTCTTCAATGTTACTTGGAGAGTTACCAACTTAATGACAGGACAAACAGCTAATGCTACCTCCCTCCTGGGGACAATGACCGATGCCTTTCCTAAACTGTACTTTGACTTGTGCGATTTAATAGGGGACGACTGRGATGAGACTGGACTCGGGTGTCGCACTCCCGGGGGAAGAAAAAGGKCAAGAATATTTGACTTCTATGTTTGCCCCGGGCATACTGTACCAACAGGGTGTGGAGGGCCGAGAGAGGGCTACTGTGGCAAATGGGGCTGTGAGACCACTGGACAGGCATACTGGAAGCCATCATCATCATGGGACCTAATTTCCCTTAAGCGAGGAAACACCCCTCRGAATCAGGGCCCCTGYTATGATTCCTCAGCGGTCTCCAGTGACATCCAGGGCGCCACACCGGGGGGTCGATGCAATCCCCTAGTCCTARAATTCACTGACGCGGGCAAAAAGGCCAGCTGGGATGGCCCCAAAGTATGGGGACTAAGACTGTACCGACCCACAGGGACCGACCCGGTGACCCGGTTCTCTTTGACCCGCCAGG

>10007049_IraAH

ACATGACAGCCCTCATCAGGTCTTCAATGTTACTTGGARAGTTACCAACTTAATGACAGGACAAACAGCTAATGCTACCTCCCTCCTGGGGACAATGACCGATGCCTTTCCTAAACTGTACTTTGACTTGTGCGATTTAATAGGGGACGACTGGGATGAGACTGGACTCGGGTGTCGCACTCCCGGGGGAAGAAAAAGGGCAAGAAYATTTGACTTCTATGTTTGCCCCGGGCATACTGTACCAACAGGGTGTGGAGGGCCGAGAGAGGGCTACTGTGGCAAATGGGGCTGTGAGACCACTGGACAGGCATACTGGAAGCCATCATCATCATGGGACCTAATTTCCCTTAAGCGAGGAAACACCCCTCGGAATCAGGGCCCCTGTTATGATTCCTCAGCGGTCTCCAGTGACATCCAGGGCGCCACACCGGGGGGTCGATGCAATCCCCTAGTCCTAGAATTCACTGACGCGGGCAAAAAGGCCAGCTGGGATGGCCCCAAAGTATGGGGACTAAGACTGTACCGAYCCACAGRGACCGACCCGGTGACCCGGTTCTCTTTGACCCGCCAGG

>5001003_IraAH

ACATGACAGCCCTCATCAGGTCTTCAATGTTACTTGGARAGTTACCAACTTAATGACAGGACAAACAGCTAATGCTACCTCCCTCCTGGGGACAATGACCGATGCCTTTCCTAAACTGTACTTTGACTTGTGCGATTTAATAGGGGACGACTGGGATGAGACTGGACTCGGGTGTCGCACTCCCGGGGGAAGAAAAAGGGCAAGAATATTTGACTTCTATGTTTGCCCCGGGCATACTGTACCAACAGGGTGTGGAGGGCCGAGAGAGGGCTACTGTGGCAAATGGGGCTGTGAGACCACTGGACAGGCATACTGGAAGCCATCATCATCATGGGACCTAATTTCCCTTAAGCGAGGAAACACCCCTCGGAATCAGGGCCCCTGYTATGATTCCTCAGCGGTCTCCAGTGACATCCAGGGCGCCACACCGGGGGGTCGATGCAATCCCCTAGTCCTAGAATTCACTGACGCGGGCAAAAAGGCCAGCTGGGATGGCCCCAAAGTATGGGGACTAAGACTGTACCGAYCCACAGGGACCGACCCGGTGACCCGGTTCTCTTTGACCCGCCAGG

>50010191_IraAH

ACATGACAGCCCTCATCAGGTCTTCAATGTTACTTGGAGAGTTACCAACTTAATGACAGGACAAACAGCTAATGCTACCTCCCTCCTGGGGACAATGACCGATGCCTTTCCTAAACTGTACTTTGACTTGTGCGATTTAATAGGGGACGACTGGGATGAGACTGGACTCGGGTGTCGCACTCCCGGGGGAAGAAAAAGGGCAAGAAYATTTGACTTCTATGTTTGCCCCGGGCATACTGTACCAACAGGGTGTGGAGGGCCGAGAGAGGGCTACTGTGGCAAATGGGGCTGTGAGACCACTGGACAGGCATACTGGAAGCCATCATCATCATGGGACCTAATTTCCCTTAAGCGAGGAAACACCCCTCGGAATCAGGGCCCCTGTTATGATTCCTCAGCGGTCTCCAGTGACATCCAGGGCGCCACACCGGGGGGTCGATGCAATCCCCTAGTCCTAGAATTCACTGACGCGGGCAAAAAGGCCAGCTGGGATGGCCCCAAAGTATGGGGACTAAGACTGTACCGAYCCACAGGGACCGACCCGGTGACCCGGTTCTCTTTGACCCGCCAGG

>50010262_IraAH

ACATGACAGCCCTCATCAGGTCTTCAATGTTACTTGGAGAGTTACCAACTTAATGACAGGACAAACAGCTAATGCTACCTCCCTCCTGGGGACAATGACCGATGCCTTTCCTAAACTGTACTTTGACTTGTGCGATTTAATAGGGGACGACTGGGATGAGACTGGACTCGGGTGTCGCACTCCCGGGGGAAGAAAAAGGGCAAGAATATTTGACTTCTATGTTTGCCCCGGGCATACTGTACCAACAGGGTGTGGAGGGCCGAGAGAGGGCTACTGTGGCAAATGGGGCTGTGAGACCACTGGACAGGCATACTGGAAGCCATCATCATCATGGGACCTAATTTCCCTTAAGCGAGGAAACACCCCTCGGAATCAGGGCCCCTGTTATGATTCCTCAGCGGTCTCCAGTGACATCCAGGGCGCCACACCGGGGGGTCGATGCAATCCCCTAGTCCTAGAATTCACTGACGCGGGCAAAAAGGCCAGCTGGGATGGCCCCAAAGTATGGGGACTAAGACTGTACCGACCCACAGGGACCGACCCGGTGACCCGGTTCTCTTTGACCCGCCAGG

>10006704_GerCB

ACATGACAGCCCTCATCAGGTCTTCAATGTTACTTGGAGAGTTACCAACTTAATGACAGGACAAACAGCTAAYGCTACCTCCCTCCTGGGGACAATGACMGATGCCTTTCCYAWRCTGTACTTYGACTTGTGCGATTTAATAGGGGACGACTGGGATGAGACTGGACTYGGGTGTCGCACTCCCGGGGGAAGAAAAAGGGCAAGAACATTTGACTTCTATGTTTGCCCCGGGCATACTGTACCAACAGGGTGTGGAGGSCCGAGAGAGGGCTACTGTGGCAAATGGGGCTGTGAGACCACTGGACAGGCATACTGGAAGCCATCATCATCATGGGACCTAATTTCCCTTAAGCGAGGAAACACCCCTCGGAATCAGGGCCCCTGTTATGATTCCTCAGCGGTCTCCAGTGGCATCCAGGGTGCCACACMGGGGGGTCGATGCAATCCCCTAGTCCTARAATTCACTGACGCGGGTAAAAAGGCCAGCTGRGATGGCCCCAAAGTATGGGGACTAAGACTGTACCGATCCACAGGAACCGACCCGGTGACCCGGTTCTCTTTGACCCGCCAGG

>10006710_GerCB

ACATGACAGCCCTCATCAGGTCTTCAATGTTACTTGGAGAGTTACCAACTTAATGACAGGACAAACAGCTAAYGCTACCTCCCTCCTGGGGACAATGACMGATGCCTTTCCYAWRCTGTACYTTGACTTGTGCGATTTAATAGGGGACGACTGGGATGAGACTGGACTYGGGTGTCGCACTCCCGGGGGAAGAAAAAGGGCAAGAACATTTGACTTCTATGTTTGCCCCGGGCATACTGTACCAACAGGGTGTGGAGGCCCGAGAGAGGGCTACTGTGGCAAATGGGGCTGTGAGACCACTGGACAGGCATACTGGAAGCCATCATCATCATGGGACCTAATTTCCCTTAAGCGAGGAAACACCCCTCGGAATCAGGGCCCCTGTTATGATTCCTCAGCGGTCTCCAGTGGCATCCAGGGTGCCACACCGGGGGGTCGATGCAATCCCCTAGTCCTAGAATTCACTGACGCGGGTAAAAAGGCCAGCTGGGATGGCCCCAAAGTATGGGGACTAAGACTGTACCGATCCACAGGAACCGACCCGGTGACCCGGTTCTCTTTGACCCGCCAGG

>10006714_GerCB

ACATGACAGCCCTCATCAGGTCTTCAATGTTACTTGGAGAGTTACCAACTTAATGACAGGACAAACAGCTAATGCTACCTCCCTCCTGGGGACAATGACCGATGCCTTTCCYAAACTGTACTTTGACTTGTGCGATTTAATAGGGGACGACTGGGATGAGACTGGACTCGGGTGTCGCACTCCCGGGGGAAGAAAAAGGGCAAGAACATTTGACTTCTATGTTTGCCCCGGGCATACTGTACCAACAGGGTGTGGAGGSCCGAGAGAGGGCTACTGTGGCAAATGGGGCTGTGAGACCACTGGACAGGCATACTGRAAGCCATCATCATCATGGGACCTAATTTCCCTTAAGCGAGGAAACACCCCTCGGAATCAGGGCCCCTGTTATGATTCCTCAGCGGTCTCCAGTGRCATCMAGGGYGCCACACCGGGGGGTCGATGCAATCCCCTAGTCCTRGAATTCACTGACGCGGGYAAAAAGGCCAGCTGGGATGGCCCCAAAGTATGGGGACTAAGACTGTACCGATCCACAGGRACCGACCCGGTGACCCGGTTCTCTTTGACCCGCCAGG

>10006760_GerCB

ACATGACAGCCCTCATCAGGTCTTCAATGTTACTTGGAGAGTTACCAACTTAATGACAGGACAAACAGCTAATGCTACCTCCCTCCTGGGGACARTGACCGATGCCTTTCCCAAACTGTACTTTGACTTGTGCGATTTAATAGGGGACGACTGGGATGAGACTGGACTCGGGTGTCGCACTCCCGGGGGAAGAAAAAGGGCAARAACATTTGACTTCTATGTTTGCCCCGGGCATACTGTACCAACAGGGTGTGGAGGCCCGAGAGAGGGCTACTGTGGCAAATGGGGCTGTGAGACCACTGGACAGGCATACTGGAAGCCATCATCATCATGGGACCTAATTTCCCTTAAGCGAGGAAACACCCCTCGGAATCAGGGCCCCTGTTATGATTCCTCAGCGGTCTCCAGTGGCATCCAGGGTGCCACACMGGGGGGTCGATGCAATCCCCTAGTCCTARAATTCACTGACGCGGGTAAAAAGGCCAGCTGGGATGGCCCCAAAGTATGGGGACTAAGACTGTACCGATCCACAGGAACCGACCCGGTGACCCGGTTCTCTTTGACCCGCCAGG

>10006876_GerCB

ACATGACAGCCCTCATCAGGTCTTCAATGTTACTTGGAGAGTTACCAACTTAATGACAGGACAAACAGCTAAYGCTACCTCCCTCCTGGGGACAATGACMGATGCCTTTCCYAWRCTGTACTTYGACTTGTGCGATTTAATAGGGGACGACTGGGATGAGACTGGACTCGGGTGTCGCACTCCCGGGGGAAGAAAAAGGGCAARAACATTTGACTTCTATGTTTGCCCCGGGCATACTGTACCAACAGGGTGTGGAGGSCCGAGAGAGGGCTACTGTGGCAAATGGGGCTGTGAGACCACTGGACAGGCATACTGGAAGCCATCATCATCATGGGACCTAATTTCCCTTAAGCGARGAAACACCCCTCRGAATCAGGGCCCCTGTTATGATTCCTCAGCGGTCTCCAGTGRCATCMAGGGYGCCACACCGGGGGGTCGATGCAATCCCCTAGTCCTRRAATTCACTGACGCGGGYAAAAAGGCCAGCTGGGATGGCCCCAAAGTATGGGGACTAAGACTGTACCGATCCACAGGAACCGACCCGGTGACCCGGTTCTCTTTGACCCGCCAGG

>10006926_GerCB

ACATGACAGCCCTCATCAGGTCTTCAATGTTACTTRGAGAGTTACCAACTTAATGACAGGACAAACAGCTAATGCTACCTCCCTCCTGGGGACAATGACCGATGCCTTTCCYAAACTGTACTTTGACTTGTGCGATTTAATAGGGGACGACTGGGATGAGACTGGACTCGGGTGTCGCACTCCCGGGGGAAGAAAAAGGGCAAGAACATTTGACTTCTATGTTTGCCCCGGGCATACTGTACCAACAGGGTGTGGAGGSCCGAGAGAGGGCTACTGTGGCAAATGGGGCTGTGAGACCACTGGACAGGCATACTGGAAGCCATCATCATCATGGGACCTAATTTCCCTTAAGCGARGAAACACCCCTCRGAATCAGGGCCCCTGTTATGATTCCTCAGCGGTCTCCAGTGRCATCMAGGGYGCCACACMGGGGGGTCGATGCAATCCCCTAGTCCTRGAATTCACTGACGCGGGYAAAAAGGCCAGCTGGGATGGCCCCAAAGTATGGGGACTAAGACTGTACCGATCCACAGGRACCGACCCGGTGACCCGGTTCTCTTTGACCCGCCAGG

>10006336_FraMC

ACATGACAGCCCTCAYCAGGTCTTCAATGTTACTTGGAGAGTTACCAACTTAATGACAGGACAAACAGCTAAYGCTACCTCCCTCCTGGGGACAATGACMGATGCCTTTCCYAWRCTGTACTTYGACTTGTGCGATTTAATAGGGGACGAYTGGGATGAGACTGGACTYGGGTGTCGCACTCCCGGGGGAAGAAAAAGGGCAAGAACATTTGACTTCTATGTTTGCCCCGGGCATACTGTACCAACAGGGTGTGGAGGSCCGAGAGAGGGCTACTGTGGCAAATGGGGCTGTGAGACCACTGGACAGGCATACTGGAAGCCATCATCATCATGGGACCTAATTTCCCTTAAGCGAGGAAACACCCCTCGGAATCAGGGCCCCTGTTATGATTCCTCAGYGGTCTCCAGTGGCATCCAGGGTGCCACACCGGGGGGTCGATGCAATCCCCTAGTCCTAGAATTCACTGACGCGGGTAAAAAGGCCAGCTGGGATGGCCCCAAAGTATGGGGACTAAGACTGTACCGATCCACAGGRAYCGACCCGGTGACCCGGTTCTCTTTGACCCGCCAGG

>10006355_FraMC

ACATGACAGCCCTCATCAGGTCTTCAATGTTACTTGGAGAGTTACCAACTTAATGACAGGACAAACAGCTAATGCTACCTCCCTCCTGGGGACAATGACCGATGCCTTTCCCAAACTGTACTTTGACTTGTGCGATTTAATAGGGGACGACTGGGATGAGACTGGACTCGGGTGTCGCACTCCCGGGGGAAGAAAAAGGGCAAGAACATTTGACTTCTATGTTTGCCCCGGGCATACTGTACCAACAGGGTGTGGAGGCCCGAGAGAGGGCTACTGTGGCAAATGGGGCTGTGAGACCACTGGACAGGCATACTGGAAGCCATCATCATCATGGGACCTAATTTCCCTTAAGCGAGGAAACACCCCTCGGAATCAGGGCCCCTGTTATGATTCCTCAGCGGTCTCCAGTGGCATCCAGGGTGCCACACCGGGGGGTCGATGCAATCCCCTAGTCCTAGAATTCACTGACGCGGGTAAAAAGGCCAGCTGGGATGGCCCCAAAGTATGGGGACTAAGACTGTACCGATCCACAGGAACCGACCCGGTGACCCGGTTCTCTTTGACCCGCCAGG

>10006398_FraMC

ACATGACAGCCCTCAYCAGGTCTTCAATGTTACTTGGAGAGTTACCAACTTAATGACAGGACAAACAGCTAAYGCTACCTCCCTCCTGGGGACAATGACMGATGCCTTTCCYAWRCTGTACTTYGACTTGTGCGATTTAATAGGGGACGAYTGGGATGAGACTGGACTYGGGTGTCGCACTCCCGGGGGAAGAAAAAGGGCAAGAACATTTGACTTCTATGTTTGCCCCGGGCATACTGTACCAACAGGGTGTGGAGGGCCGAGAGAGGGCTACTGTGGCAAATGGGGCTGTGAGACCACTGGACAGGCATACTGGAAGCCATCATCATCATGGGACCTAATTTCCCTTAAGCGAGGAAACACCCCTCGGAATCAGGGCCCCTGTTATGATTCCTCAGYGGTCTCCAGTGRCATCMAGGGYGCCACACCGGGGGGTCGATGCAATCCCCTAGTCCTRGAATTCACTGACGCGGGTAAAAAGGCCAGCTGGGATGGCCCCAAAGTATGGGGACTAAGACTGTACCGATCCACAGGRAYCGACCCGGTGACCCGGTTCTCTTTGACCCGCCAGG

>10006403_FraMC

ACATGACAGCCCTCACCAGGTCTTCAATGTTACTTGGAGAGTTACCAACTTAATGACAGGACAAACAGCTAAYGCTACCTCCCTCCTGGGGACAATGACMGATGCCTTTCCYAWRCTGTACTTYGACTTGTGCGATTTAATAGGGGACGATTGGGATGAGACTGGACTYGGGTGTCGCACTCCCGGGGGAAGAAAAAGGGCAAGAACATTTGACTTCTATGTTTGCCCCGGGCATACTGTACCAACAGGGTGTGGAGGSCCGAGAGAGGGCTACTGTGGCAAATGGGGCTGTGAGACCACTGGACAGGCATACTGGAAGCCATCATCATCATGGGACCTAATTTCCCTTAAGCGAGGAAACACCCCTCGGAATCAGGGCCCCTGTTATGATTCCTCAGYGGTCTCCAGTGGCATCCAGGGTGCCACACCGGGGGGTCGATGCAATCCCCTAGTCCTRGAATTCACTGACGCGGGTAAAAAGGCCAGCTGGGATGGCCCCAAAGTATGGGGACTAAGACTGTACCGATCCACAGGRAYCGACCCGGTGACCCGGTTCTCTTTGACCCGCCAGG

>10006437_FraMC

ACATGACAGCCCTCAYCAGGTCTTCAATGTTACTTGGAGAGTTACCAACTTAATGACAGGACAAACAGCTAAYGCTACCTCCCTCCTGGGGACAATGACMGATGCCTTTCCYAWRCTGTACTTYGACTTGTGCGATTTAATAGGGGACGAYTGGGATGAGACTGGACTYGGGTGTCGCACTCCCGGGGGAAGAAAAAGGGCAAGAACATTTGACTTCTATGTTTGCCCCGGGCATACTGTACCAACAGGGTGTGGAGGSCCGAGAGAGGGCTACTGTGGCAAATGGGGCTGTGAGACCACTGGACAGGCATACTGGAAGCCATCATCATCATGGGACCTAATTTCCCTTAAGCGAGGAAACACCCCTCGGAATCAGGGCCCCTGTTATGATTCCTCAGYRGTCTCCAGTGGCATCCAGGGTGCCACACCGGGGGGTCGATGCAATCCCCTAGTCCTAGAATTCACTGACGCGGGTAAAAAGGCCAGCTGGGATGGCCCCAAAGTATGGGGACTAAGACTGTACCGATCCACAGGRAYCGACCCGGTGACCCGGTTCTCTTTGACCCGCCAGG

>10006466_FraMC

ACATGACAGCCCTCAYCAGGTCTTCAATGTTACTTGGAGAGTTACCAACTTAATGACAGGACAAACAGCTAAYGCTACCTCCCTCCTGGGGACAATGACMGATGCCTTTCCTAWRCTGTACTTYGACTTGTGCGATTTAATAGGGGACGAYTGGGATGAGACTGGACTYGGGTGTCGCACTCCCGGGGGAAGAAAAAGGGCAAGAACATTTGACTTCTATGTTTGCCCCGGGCATACTGTACCAACAGGGTGTGGAGGGCCGAGAGAGGGCTACTGTGGCAAATGGGGCTGTGAGACCACTGGACAGGCATACTGGAAGCCATCATCATCATGGGACCTAATTTCCCTTAAGCGAGGAAACACCCCTCGGAATCAGGGCCCCTGTTATGATTCCTCAGYGGTCTCCAGTGRCATCCAGGGYGCCACACCGGGGGGTCGATGCAATCCCCTAGTCCTRGAATTCACTGACGCGGGTAAAAAGGCCAGCTGGGATGGCCCCAAAGTATGGGGACTAAGACTGTACCGATCCACAGGRAYCGACCCGGTGACCCGGTTCTCTTTGACCCGCCAGG

>10006471_FraMC

ACATGACAGCCCTCAYCAGGTCTTCAATGTTACTTGGAGAGTTACCAACTTAATGACAGGACAAACAGCTAAYGCTACCTCCCTCCTGGGGACAATGACMGATGCCTTTCCYAWRCTGTACTTYGACTTGTGCGATTTAATAGGGGACGAYTGGGATGAGACTGGACTYGGGTGTCGCACTCCCGGGGGAAGAAAAAGGGCAAGAACATTTGACTTCTATGTTTGCCCCGGGCATACTGTACCAACAGGGTGTGGAGGSCCGAGAGAGGGCTACTGTGGCAAATGGGGCTGTGAGACCACTGGACAGGCATACTGGAAGCCATCATCATCATGGGACCTAATTTCCCTTAAGCGAGGAAACACCCCTCGGAATCAGGGCCCCTGTTATGATTCCTCAGYGGTCTCCAGTGGCATCCAGGGTGCCACACCGGGGGGTCGATGCAATCCCCTAGTCCTRGAATTCACTGACGCGGGTAAAAAGGCCAGCTGGGATGGCCCCAAAGTATGGGGACTAAGACTGTACCGATCCACAGGRAYCGACCCGGTGACCCGGTTCTCTTTGACCCGCCAGG

>10006472_FraMC

ACATGACAGCCCTCAYCAGGTCTTCAATGTTACTTGGAGAGTTACCAACTTAATGACAGGACAAACAGCTAAYGCTACCTCCCTCCTGGGGACAATGACMGATGCCTTTCCYAWRCTGTACTTYGACTTGTGCGATTTAATAGGGGACGAYTGGGATGAGACTGGACTYGGGTGTCGCACTCCCGGGGGAAGAAAAAGGGCAAGAACATTTGACTTCTATGTTTGCCCCGGGCATACTGTACCAACAGGGTGTGGAGGSCCGAGAGAGGGCTACTGTGGCAAATGGGGCTGTGAGACCACTGGACAGGCATACTGGAAGCCATCATCATCATGGGACCTAATTTCCCTTAAGCGAGGAAACACCCCTCGGAATCAGGGCCCCTGTTATGATTCCTCAGYRGTCTCCAGTGRCATCCAGGGYGCCACACCGGGGGGTCGATGCAATCCCCTAGTCCTRGAATTCACTGACGCGGGYAAAAAGGCCAGCTGGGATGGCCCCAAAGTATGGGGACTAAGACTGTACCGATCCACAGGRAYCGACCCGGTGACCCGGTTCTCTTTGACCCGCCAGG

>10006473_FraMC

ACATGACAGCCCTCAYCAGGTCTTCAATGTTACTTGGAGAGTTACCAACTTAATGACAGGACAAACAGCTAAYGCTACCTCCCTCCTGGGGACAATGACMGATGCCTTTCCYAWRCTGTACTTYGACTTGTGCGATTTAATAGGGGACGAYTGGGATGAGACTGGACTYGGGTGTCGCACTCCCGGGGGAAGAAAAAGGGCAAGAACATTTGACTTCTATGTTTGCCCCGGGCATACTGTACCAACAGGGTGTGGAGGSCCGAGAGAGGGCTACTGTGGCAAATGGGGCTGTGAGACCACTGGACAGGCATACTGGAAGCCATCATCATCATGGGACCTAATTTCCCTTAAGCGAGGAAACACCCCTCGGAATCAGGGCCCCTGTTATGATTCCTCAGYRGTCTCCAGTGGCATCCAGGGTGCCACACCGGGGGGTCGATGCAATCCCCTAGTCCTAGAATTCACTGACGCGGGTAAAAAGGCCAGCTGGGATGGCCCCAAAGTATGGGGACTAAGACTGTACCGATCCACAGGRAYCGACCCGGTGACCCGGTTCTCTTTGACCCGCCAGG

>10006571_FraMC

ACATGACAGCCCTCAYCAGGTCTTCAATGTTACTTGGAGAGTTACCAACTTAATGACAGGACAAACAGCTAAYGCTACCTCCCTCCTGGGGACAATGACMGATGCCTTTCCYAWRCTGTACTTYGACTTGTGCGATTTAATAGGGGACGAYTGGGATGAGACTGGACTYGGGTGTCGCACTCCCGGGGGAAGAAAAAGGGCAAGAACATTTGACTTCTATGTTTGCCCCGGGCATACTGTACCAACAGGGTGTGGAGGSCCGAGAGAGGGCTACTGTGGCAAATGGGGCTGTGAGACCACTGGACAGGCATACTGGAAGCCATCATCATCATGGGACCTAATTTCCCTTAAGCGAGGAAACACCCCTCGGAATCAGGGCCCCTGTTATGATTCCTCAGYRGTCTCCAGTGGCATCCAGGGTGCCACACCGGGGGGTCGATGCAATCCCCTAGTCCTAGAATTCACTGACGCGGGTAAAAAGGCCAGCTGGGATGGCCCCAAAGTATGGGGACTAAGACTGTACCGATCCACAGGRAYCGACCCGGTGACCCGGTTCTCTTTGACCCGCCAGG

>10006984_FraMC

ACATGACAGCCCTCAYCAGGTCTTCAATGTTACTTGGAGAGTTACCAACTTAATGACAGGACAAACAGCTAAYGCTACCTCCCTCCTGGGGACAATGACMGATGCCTTTCCYAWRCTGTACTTYGACTTGTGCGATTTAATAGGGGACGAYTGGGATGAGACTGGACTYGGGTGTCGCACTCCCGGGGGAAGAAAAAGGGCAAGAACATTTGACTTCTATGTTTGCCCCGGGCATACTGTACCAACAGGGTGTGGAGGSCCGAGAGAGGGCTACTGTGGCAAATGGGGCTGTGAGACCACTGGACAGGCATACTGGAAGCCATCATCATCATGGGACCTAATTTCCCTTAAGCGAGGAAACACCCCTCGGAATCAGGGCCCCTGTTATGATTCCTCAGYGGTCTCCAGTGGCATCCAGGGTGCCACACCGGGGGGTCGATGCAATCCCCTAGTCCTAGAATTCACTGACGCGGGTAAAAAGGCCAGCTGGGATGGCCCCAAAGTATGGGGACTAAGACTGTACCGATCCACAGGRAYCGACCCGGTGACCCGGTTCTCTTTGACCCGCCAGG

>10007045_FraMC

ACATGACAGCCCTCAYCAGGTCTTCAATGTTACTTGGAGAGTTACCAACTTAATGACAGGACAAACAGCTAAYGCTACCTCCCTCCTGGGGACAATGACMGATGCCTTTCCTAWRCTGTACTTYGACTTGTGCGATTTAATAGGGGACGAYTGGGATGAGACTGGACTYGGGTGTCGCACTCCCGGGGGAAGAAAAAGGGCAAGAACATTTGACTTCTATGTTTGCCCCGGGCATACTGTACCAACAGGGTGTGGAGGGCCGAGAGAGGGCTACTGTGGCAAATGGGGCTGTGAGACCACTGGACAGGCATACTGGAAGCCATCATCATCATGGGACCTAATTTCCCTTAAGCGAGGAAACACCCCTCGGAATCAGGGCCCCTGTTATGATTCCTCAGCRGTCTCCAGTGRCATCCAGGGYGCCACACCGGGGGGTCGATGCAATCCCCTAGTCCTRGAATTCACTGACGCGGGYAAAAAGGCCAGCTGGGATGGCCCCAAAGTATGGGGACTAAGACTGTACCGATCCACAGGGAYCGACCCGGTGACCCGGTTCTCTTTGACCCGCCAGG

**RBD Sequences of P-MLV variants from the reciprocal crossing experiment**

>MLV101_GerCB_M_before

ACATGACAGCCCTCATCAGGTCTTCAATGTTACTTGGAGAGTTACCAACTTAATGACAGGACAAACAGCTAATGCTACCTCCCTCCTGGGGACAATGACCGATGCCTTTCCYAAACTGTACTTTGACTTGTGCGATTTAATAGGGGACGACTGGGATGAGACTGGACTCGGGTGTCGCACTCCCGGGGGAAGAAAAAGGGCAAGAACATTTGACTTCTATGTTTGCCCCGGGCATACTGTACCAACAGGGTGTGGAGGSCCGAGAGAGGGCTACTGTGGCAAATGGGGCTGTGAGACCACTGGACAGGCATACTGGAAGCCATCATCATCATGGGACCTAATTTCCCTTAAGCGAGGAAACACCCCTCGGAATCAGGGCCCCTGTTATGATTCCTCAGCGGTCTCCAGTGRCATCMAGGGYGCCACACCGGGGGGTCGATGCAATCCCCTAGTCCTRGAATTCACTGACGCGGGYAAAAAGGCCAGCTGGGATGGCCCCAAAGTATGGGGACTAAGACTGTACCGATCCACAGGRACCGACCCGGTGACCCGGTTCTCTTTGACCCGCCAGG

>MLV101_GerCB_M_after

ACATGACAGCCCTCATCAGGTCTTCAATGTTACTTGGAGAGTTACCAACTTAATGACAGGACAAACAGCTAATGCTACCTCCCTCCTGGGGACAATGACCGATGCCTTTCCYAAACTGTACTTTGACTTGTGCGATTTAATAGGGGACGACTGGGATGAGACTGGACTCGGGTGTCGCACTCCCGGGGGAAGAAAAAGGGCAAGAACATTTGACTTCTATGTTTGCCCCGGGCATACTGTACCAACAGGGTGTGGAGGSCCGAGAGAGGGCTACTGTGGCAAATGGGGCTGTGAGACCACTGGACAGGCATACTGGAAGCCATCATCATCATGGGACCTAATTTCCCTTAAGCGAGGAAACACCCCTCGGAATCAGGGCCCCTGTTATGATTCCTCAGCGGTCTCCAGTGRCATCMAGGGYGCCACACCGGGGGGTCGATGCAATCCCCTAGTCCTRGAATTCACTGACGCGGGYAAAAAGGCCAGCTGGGATGGCCCCAAAGTATGGGGACTAAGACTGTACCGATCCACAGGRACCGACCCGGTGACCCGGTTCTCTTTGACCCGCCAGG

>MLV101_IraAH_F_before

ACATGACAGCCCTCATCAGGTCTTCAATGTTACTTGGAGAGTTACCAACTTAATGACAGGACAAACAGCTAATGCTACCTCCCTCCTGGGGACAATGACCGATGCCTTTCCTAAACTGTACTTTGACTTGTGCGATTTAATAGGGGACGACTGRRATGAGACTGGACTCGGGTGTCGCACTCCCGGGGGAAGAAAAAGGGCAAGAATATTTGACTTCTATGTTTGCCCCGGGCATACTGTACCAACAGGGTGTGGAGGGCCGAGAGAGGGCTACTGTGGCAAATGGGGCTGTGAGACCACTGGACAGGCATACTGGAAGCCATCATCATCATGGGACCTAATTTCCCTTAAGCGAGGAAACACCCCTCRGAATCAGGGCCCCTGYTATGATTCCTCAGCGGTCTCCAGTGACATCCAGGGCGCCACACCGGGGGGTCGATGCAATCCCCTAGTCCTARAATTCACTGACGCGGGCAAAAAGGCCAGCTGGGATGGCCCCAAAGTATGGGGACTAARACTGTACCGACCCACAGGGACCGACCCGGTGACCCGGTTCTCTTTGACCCGCCAGG

>MLV101_IraAH_F_after

ACATGACAGCCCTCATCAGGTCTTCAATGTTACTTGGAGAGTTACCAACTTAATGACAGGACAAACAGCTAATGCTACCTCCCTCCTGGGGACAATGACCGATGCCTTTCCTAAACTGTACTTTGACTTGTGCGATTTAATAGGGGACGACTGRRATGAGACTGGACTCGGGTGTCGCACTCCCGGGGGAAGAAAAAGGGCAAGAATATTTGACTTCTATGTTTGCCCCGGGCATACTGTACCAACAGGGTGTGGAGGGCCGAGAGAGGGCTACTGTGGCAAATGGGGCTGTGAGACCACTGGACAGGCATACTGGAAGCCATCATCATCATGGGACCTAATTTCCCTTAAGCGAGGAAACACCCCTCRGAATCAGGGCCCCTGYTATGATTCCTCAGCGGTCTCCAGTGACATCCAGGGCGCCACACCGGGGGGTCGATGCAATCCCCTAGTCCTARAATTCACTGACGCGGGCAAAAAGGCCAGCTGGGATGGCCCCAAAGTATGGGGACTAARACTGTACCGACCCACAGGGACCGACCCGGTGACCCGGTTCTCTTTGACCCGCCAGG

>MLV101_1_GerCBxIraAH

ACATGACAGCCCTCATCAGGTCTTCAATGTTACTTGGAGAGTTACCAACTTAATGACAGGACAAACAGCTAATGCTACCTCCCTCCTGGGGACAATGACCGATGCCTTTCCYAAACTGTACTTTGACTTGTGCGATTTAATAGGGGACGACTGGGATGAGACTGGACTCGGGTGTCGCACTCCCGGGGGAAGAAAAAGGGCAAGAAYATTTGACTTCTATGTTTGCCCCGGGCATACTGTACCAACAGGGTGTGGAGGGCCGAGAGAGGGCTACTGTGGCAAATGGGGCTGTGAGACCACTGGACAGGCATACTGGAAGCCATCATCATCATGGGACCTAATTTCCCTTAAGCGAGGAAACACCCCTCGGAATCAGGGCCCCTGTTATGATTCCTCAGCGGTCTCCAGTGACATCCAGGGCGCCACACCGGGGGGTCGATGCAATCCCCTAGTCCTAGAATTCACTGACGCGGGCAAAAAGGCCAGCTGGGATGGCCCCAAAGTATGGGGACTAAGACTGTACCGAYCCACAGGGACCGACCCGGTGACCCGGTTCTCTTTGACCCGCCAGG

>MLV101_2_GerCBxIraAH

ACATGACAGCCCTCATCAGGTCTTCAATGTTACTTGGAGAGTTACCAACTTAATGACAGGACAAACAGCTAATGCTACCTCCCTCCTGGGGACAATGACCGATGCCTTTCCYAAACTGTACTTTGACTTGTGCGATTTAATAGGGGACGACTGGGATGAGACTGGACTCGGGTGTCGCACTCCCGGGGGAAGAAAAAGGGCAAGAAYATTTGACTTCTATGTTTGCCCCGGGCATACTGTACCAACAGGGTGTGGAGGGCCGAGAGAGGGCTACTGTGGCAAATGGGGCTGTGAGACCACTGGACAGGCATACTGGAAGCCATCATCATCATGGGACCTAATTTCCCTTAAGCGAGGAAACACCCCTCRGAATCAGGGCCCCTGYTATGATTCCTCAGCGGTCTCCAGTGACATCCAGGGCGCCACACCGGGGGGTCGATGCAATCCCCTAGTCCTAGAATTCACTGACGCGGGCAAAAAGGCCAGCTGGGATGGCCCCAAAGTATGGGGACTAAGACTGTACCGAYCCACAGGGACCGACCCGGTGACCCGGTTCTCTTTGACCCGCCAGG

>MLV101_3_GerCBxIraAH

ACATGACAGCCCTCATCAGGTCTTCAATGTTACTTGGAGAGTTACCAACTTAATGACAGGACAAACAGCTAATGCTACCTCCCTCCTGGGGACAATGACCGATGCCTTTCCYAAACTGTACTTTGACTTGTGCGATTTAATAGGGGACGACTGGGATGAGACTGGACTCGGGTGTCGCACTCCCGGGGGAAGAAAAAGGGCAAGAAYATTTGACTTCTATGTTTGCCCCGGGCATACTGTACCAACAGGGTGTGGAGGGCCGAGAGAGGGCTACTGTGGCAAATGGGGCTGTGAGACCACTGGACAGGCATACTGGAAGCCATCATCATCATGGGACCTAATTTCCCTTAAGCGAGGAAACACCCCTCRGAATCAGGGCCCCTGYTATGATTCCTCAGCGGTCTCCAGTGACATCCAGGGCGCCACACCGGGGGGTCGATGCAATCCCCTAGTCCTARAATTCACTGACGCGGGCAAAAAGGCCAGCTGGGATGGCCCCAAAGTATGGGGACTAAGACTGTACCGAYCCACAGGGACCGACCCGGTGACCCGGTTCTCTTTGACCCGCCAGG

>MLV101_4_GerCBxIraAH

ACATGACAGCCCTCATCAGGTCTTCAATGTTACTTGGAGAGTTACCAACTTAATGACAGGACAAACAGCTAATGCTACCTCCCTCCTGGGGACAATGACCGATGCCTTTCCTAAACTGTACTTTGACTTGTGCGATTTAATAGGGGACGACTGRRATGAGACTGGACTCGGGTGTCGCACTCCCGGGGGAAGAAAAAGGGCAAGAAYATTTGACTTCTATGTTTGCCCCGGGCATACTGTACCAACAGGGTGTGGAGGGCCGAGAGAGGGCTACTGTGGCAAATGGGGCTGTGAGACCACTGGACAGGCATACTGGAAGCCATCATCATCATGGGACCTAATTTCCCTTAAGCGAGGAAACACCCCTCRGAATCAGGGCCCCTGYTATGATTCCTCAGCGGTCTCCAGTGACATCCAGGGCGCCACACCGGGGGGTCGATGCAATCCCCTAGTCCTARAATTCACTGACGCGGGCAAAAAGGCCAGCTGGGATGGCCCCAAAGTATGGGGACTAAGACTGTACCGAYCCACAGGGACCGACCCGGTGACCCGGTTCTCTTTGACCCGCCAGG

>MLV101_5_GerCBxIraAH

ACATGACAGCCCTCATCAGGTCTTCAATGTTACTTGGAGAGTTACCAACTTAATGACAGGACAAACAGCTAATGCTACCTCCCTCCTGGGGACAATGACCGATGCCTTTCCTAAACTGTACTTTGACTTGTGCGATTTAATAGGGGACGACTGGGATGAGACTGGACTCGGGTGTCGCACTCCCGGGGGAAGAAAAAGGGCAAGAAYATTTGACTTCTATGTTTGCCCCGGGCATACTGTACCAACAGGGTGTGGAGGGCCGAGAGAGGGCTACTGTGGCAAATGGGGCTGTGAGACCACTGGACAGGCATACTGGAAGCCATCATCATCATGGGACCTAATTTCCCTTAAGCGAGGAAACACCCCTCGGAATCAGGGCCCCTGTTATGATTCCTCAGCGGTCTCCAGTGACATCCAGGGYGCCACACCGGGGGGTCGATGCAATCCCCTAGTCCTAGAATTCACTGACGCGGGCAAAAAGGCCAGCTGGGATGGCCCCAAAGTATGGGGACTAAGACTGTACCGAYCCACAGGGACCGACCCGGTGACCCGGTTCTCTTTGACCCGCCAGG

>MLV101_6_GerCBxIraAH

ACATGACAGCCCTCATCAGGTCTTCAATGTTACTTGGAGAGTTACCAACTTAATGACAGGACAAACAGCTAATGCTACCTCCCTCCTGGGGACAATGACCGATGCCTTTCCTAAACTGTACTTTGACTTGTGCGATTTAATAGGGGACGACTGGRATGAGACTGGACTCGGGTGTCGCACTCCCGGGGGAAGAAAAAGGGCAAGAAYATTTGACTTCTATGTTTGCCCCGGGCATACTGTACCAACAGGGTGTGGAGGGCCGAGAGAGGGCTACTGTGGCAAATGGGGCTGTGAGACCACTGGACAGGCATACTGGAAGCCATCATCATCATGGGACCTAATTTCCCTTAAGCGAGGAAACACCCCTCGGAATCAGGGCCCCTGTTATGATTCCTCAGCGGTCTCCAGTGACATCCAGGGCGCCACACCGGGGGGTCGATGCAATCCCCTAGTCCTAGAATTCACTGACGCGGGCAAAAAGGCCAGCTGGGATGGCCCCAAAGTATGGGGACTAAGACTGTACCGAYCCACAGGGACCGACCCGGTGACCCGGTTCTCTTTGACCCGCCAGG

>MLV102_IraAH_M_before

ACATGACAGCCCTCATCAGGTCTTCAATGTTACTTGGARAGTTACCAACTTAATGACAGGACAAACAGCTAATGCTACCTCCCTCCTGGGGACAATGACCGATGCCTTTCCTAAACTGTACTTTGACTTGTGCGATTTAATAGGGGACGACTGGGATGAGACTGGACTCGGGTGTCGCACTCCCGGGGGAAGAAAAAGGGCAAGAAYATTTGACTTCTATGTTTGCCCCGGGCATACTGTACCAACAGGGTGTGGAGGGCCGAGAGAGGGCTACTGTGGCAAATGGGGCTGTGAGACCACTGGACAGGCATACTGGAAGCCATCATCATCATGGGACCTAATTTCCCTTAAGCGAGGAAACACCCCTCGGAATCAGGGCCCCTGTTATGATTCCTCAGCGGTCTCCAGTGACATCCAGGGCGCCACACCGGGGGGTCGATGCAATCCCCTAGTCCTAGAATTCACTGACGCGGGCAAAAAGGCCAGCTGGGATGGCCCCAAAGTATGGGGACTAAGACTGTACCGAYCCACAGGGACCGACCCGGTGACCCGGTTCTCTTTGACCCGCCAGG

>MLV102_IraAH_M_after

ACATGACAGCCCTCATCAGGTCTTCAATGTTACTTGGARAGTTACCAACTTAATGACAGGACAAACAGCTAATGCTACCTCCCTCCTGGGGACAATGACCGATGCCTTTCCTAAACTGTACTTTGACTTGTGCGATTTAATAGGGGACGACTGGGATGAGACTGGACTCGGGTGTCGCACTCCCGGGGGAAGAAAAAGGGCAAGAAYATTTGACTTCTATGTTTGCCCCGGGCATACTGTACCAACAGGGTGTGGAGGGCCGAGAGAGGGCTACTGTGGCAAATGGGGCTGTGAGACCACTGGACAGGCATACTGGAAGCCATCATCATCATGGGACCTAATTTCCCTTAAGCGAGGAAACACCCCTCGGAATCAGGGCCCCTGTTATGATTCCTCAGCGGTCTCCAGTGACATCCAGGGCGCCACACCGGGGGGTCGATGCAATCCCCTAGTCCTAGAATTCACTGACGCGGGCAAAAAGGCCAGCTGGGATGGCCCCAAAGTATGGGGACTAAGACTGTACCGAYCCACAGGGACCGACCCGGTGACCCGGTTCTCTTTGACCCGCCAGG

>MLV102_GerCB_F_before

ACATGACAGCCCTCATCAGGTCTTCAATGTTACTTGGAGAGTTACCAACTTAATGACAGGACAAACAGCTAATGCTACCTCCCTCCTGGGGACAATGACCGATGCCTTTCCYAAACTGTACTTTGACTTGTGCGATTTAATAGGGGACGACTGGGATGAGACTGGACTCGGGTGTCGCACTCCCGGGGGAAGAAAAAGGGCAAGAACATTTGACTTCTATGTTTGCCCCGGGCATACTGTACCAACAGGGTGTGGAGGSCCGAGAGAGGGCTACTGTGGCAAATGGGGCTGTGAGACCACTGGACAGGCATACTGGAAGCCATCATCATCATGGGACCTAATTTCCCTTAAGCGAGGAAACACCCCTCGGAATCAGGGCCCCTGTTATGATTCCTCAGCGGTCTCCAGTGRCATCMAGGGYRCCACACCGGGGGGTCGATGCAATCCCCTAGTCCTRGAATTCACTGACGCGGGYAAAAAGGCCAGCTGGGATGGCCCCAAAGTATGGGGACTAAGACTGTACCGATCCACAGGRACCGACCCGGTGACCCGGTTCTCTTTGACCCGCCAGG

>MLV102_GerCB_F_after

ACATGACAGCCCTCATCAGGTCTTCAATGTTACTTGGAGAGTTACCAACTTAATGACAGGACAAACAGCTAATGCTACCTCCCTCCTGGGGACAATGACCGATGCCTTTCCYAAACTGTACTTTGACTTGTGCGATTTAATAGGGGACGACTGGGATGAGACTGGACTCGGGTGTCGCACTCCCGGGGGAAGAAAAAGGGCAAGAACATTTGACTTCTATGTTTGCCCCGGGCATACTGTACCAACAGGGTGTGGAGGSCCGAGAGAGGGCTACTGTGGCAAATGGGGCTGTGAGACCACTGGACAGGCATACTGGAAGCCATCATCATCATGGGACCTAATTTCCCTTAAGCGAGGAAACACCCCTCGGAATCAGGGCCCCTGTTATGATTCCTCAGCGGTCTCCAGTGRCATCMAGGGYRCCACACCGGGGGGTCGATGCAATCCCCTAGTCCTRGAATTCACTGACGCGGGYAAAAAGGCCAGCTGGGATGGCCCCAAAGTATGGGGACTAAGACTGTACCGATCCACAGGRACCGACCCGGTGACCCGGTTCTCTTTGACCCGCCAGG

>MLV102_1_IraAHxGerCB

ACATGACAGCCCTCATCAGGTCTTCAATGTTACTTGGAGAGTTACCAACTTAATGACAGGACAAACAGCTAATGCTACCTCCCTCCTGGGGACAATGACCGATGCCTTTCCTAAACTGTACTTTGACTTGTGCGATTTAATAGGGGACGACTGGGATGAGACTGGACTCGGGTGTCGCACTCCCGGGGGAAGAAAAAGGGCAAGAAYATTTGACTTCTATGTTTGCCCCGGGCATACTGTACCAACAGGGTGTGGAGGGCCGAGAGAGGGCTACTGTGGCAAATGGGGCTGTGAGACCACTGGACAGGCATACTGGAAGCCATCATCATCATGGGACCTAATTTCCCTTAAGCGAGGAAACACCCCTCGGAATCAGGGCCCCTGTTATGATTCCTCAGCGGTCTCCAGTGACATCCAGGGCGCCACACCGGGGGGTCGATGCAATCCCCTAGTCCTAGAATTCACTGACGCGGGCAAAAAGGCCAGCTGGGATGGCCCCAAAGTATGGGGACTAAGACTGTACCGAYCCACAGGGACCGACCCGGTGACCCGGTTCTCTTTGACCCGCCAGG

>MLV102_2_IraAHxGerCB

ACATGACAGCCCTCATCAGGTCTTCAATGTTACTTGGAGAGTTACCAACTTAATGACAGGACAAACAGCTAATGCTACCTCCCTCCTGGGGACAATGACCGATGCCTTTCCTAAACTGTACTTTGACTTGTGCGATTTAATAGGGGACGACTGGGATGAGACTGGACTCGGGTGTCGCACTCCCGGGGGAAGAAAAAGGGCAAGAAYATTTGACTTCTATGTTTGCCCCGGGCATACTGTACCAACAGGGTGTGGAGGGCCGAGAGAGGGCTACTGTGGCAAATGGGGCTGTGAGACCACTGGACAGGCATACTGGAAGCCATCATCATCATGGGACCTAATTTCCCTTAAGCGAGGAAACACCCCTCGGAATCAGGGCCCCTGTTATGATTCCTCAGCGGTCTCCAGTGACATCCAGGGCGCCACACCGGGGGGTCGATGCAATCCCCTAGTCCTAGAATTCACTGACGCGGGCAAAAAGGCCAGCTGGGATGGCCCCAAAGTATGGGGACTAAGACTGTACCGAYCCACAGGRACCGACCCGGTGACCCGGTTCTCTTTGACCCGCCAGG

>MLV102_3_IraAHxGerCB

ACATGACAGCCCTCATCAGGTCTTCAATGTTACTTGGARAGTTACCAACTTAATGACAGGACAAACAGCTAATGCTACCTCCCTCCTGGGGACAATGACCGATGCCTTTCCYAAACTGTACTTTGACTTGTGCGATTTAATAGGGGACGACTGGGATGAGACTGGACTCGGGTGTCGCACTCCCGGGGGAAGAAAAAGGGCAAGAAYATTTGACTTCTATGTTTGCCCCGGGCATACTGTACCAACAGGGTGTGGAGGSCCGAGAGAGGGCTACTGTGGCAAATGGGGCTGTGAGACCACTGGACAGGCATACTGGAAGCCATCATCATCATGGGACCTAATTTCCCTTAAGCGAGGAAACACCCCTCGGAATCAGGGCCCCTGTTATGATTCCTCAGCGGTCTCCAGTGRCATCCAGGGYGCCACACCGGGGGGTCGATGCAATCCCCTAGTCCTAGAATTCACTGACGCGGGYAAAAAGGCCAGCTGGGATGGCCCCAAAGTATGGGGACTAAGACTGTACCGATCCACAGGRACCGACCCGGTGACCCGGTTCTCTTTGACCCGCCAGG

>MLV102_4_IraAHxGerCB

ACATGACAGCCCTCATCAGGTCTTCAATGTTACTTGGAGAGTTACCAACTTAATGACAGGACAAACAGCTAATGCTACCTCCCTCCTGGGGACAATGACCGATGCCTTTCCTAAACTGTACTTTGACTTGTGCGATTTAATAGGGGACGACTGGGATGAGACTGGACTCGGGTGTCGCACTCCCGGGGGAAGAAAAAGGGCAAGAAYATTTGACTTCTATGTTTGCCCCGGGCATACTGTACCAACAGGGTGTGGAGGGCCGAGAGAGGGCTACTGTGGCAAATGGGGCTGTGAGACCACTGGACAGGCATACTGGAAGCCATCATCATCATGGGACCTAATTTCCCTTAAGCGAGGAAACACCCCTCGGAATCAGGGCCCCTGTTATGATTCCTCAGCGGTCTCCAGTGACATCCAGGGCGCCACACCGGGGGGTCGATGCAATCCCCTAGTCCTAGAATTCACTGACGCGGGCAAAAAGGCCAGCTGGGATGGCCCCAAAGTATGGGGACTAAGACTGTACCGAYCCACAGGGACCGACCCGGTGACCCGGTTCTCTTTGACCCGCCAGG

>MLV102_5_IraAHxGerCB

ACATGACAGCCCTCATCAGGTCTTCAATGTTACTTGGAGAGTTACCAACTTAATGACAGGACAAACAGCTAATGCTACCTCCCTCCTGGGGACAATGACCGATGCCTTTCCYAAACTGTACTTTGACTTGTGCGATTTAATAGGGGACGACTGGGATGAGACTGGACTCGGGTGTCGCACTCCCGGGGGAAGAAAAAGGGCAAGAAYATTTGACTTCTATGTTTGCCCCGGGCATACTGTACCAACAGGGTGTGGAGGSCCGAGAGAGGGCTACTGTGGCAAATGGGGCTGTGAGACCACTGGACAGGCATACTGGAAGCCATCATCATCATGGGACCTAATTTCCCTTAAGCGAGGAAACACCCCTCGGAATCAGGGCCCCTGTTATGATTCCTCAGCGGTCTCCAGTGACATCCAGGGCGCCACACCGGGGGGTCGATGCAATCCCCTAGTCCTAGAATTCACTGACGCGGGCAAAAAGGCCAGCTGGGATGGCCCCAAAGTATGGGGACTAAGACTGTACCGAYCCACAGGGACCGACCCGGTGACCCGGTTCTCTTTGACCCGCCAGG
